# Supplementary material for: YOLOv8s-CGF: a lightweight model for wheat ear Fusarium head blight detection
Source: PeerJ Comput Sci. 2024 Mar 27;10:e1948. doi: 10.7717/peerj-cs.1948 (PMC11041926; doi:10.7717/peerj-cs.1948)
Supplement: Supplemental Information 1 [file peerj-cs-10-1948-s001.zip › ultralytics-main/docs/overrides/partials/source-file.html]

{% import "partials/language.html" as lang with context %}
  

{% if page.meta.git\_revision\_date\_localized %}
📅 {{ lang.t("source.file.date.updated") }}:
{{ page.meta.git\_revision\_date\_localized }}
{% if page.meta.git\_creation\_date\_localized %}
