# Supplementary figures and images for: YOLOv8s-CGF: a lightweight model for wheat ear Fusarium head blight detection (part 2 of 3)
Source: PeerJ Comput Sci. 2024 Mar 27;10:e1948. doi: 10.7717/peerj-cs.1948 (PMC11041926; doi:10.7717/peerj-cs.1948)

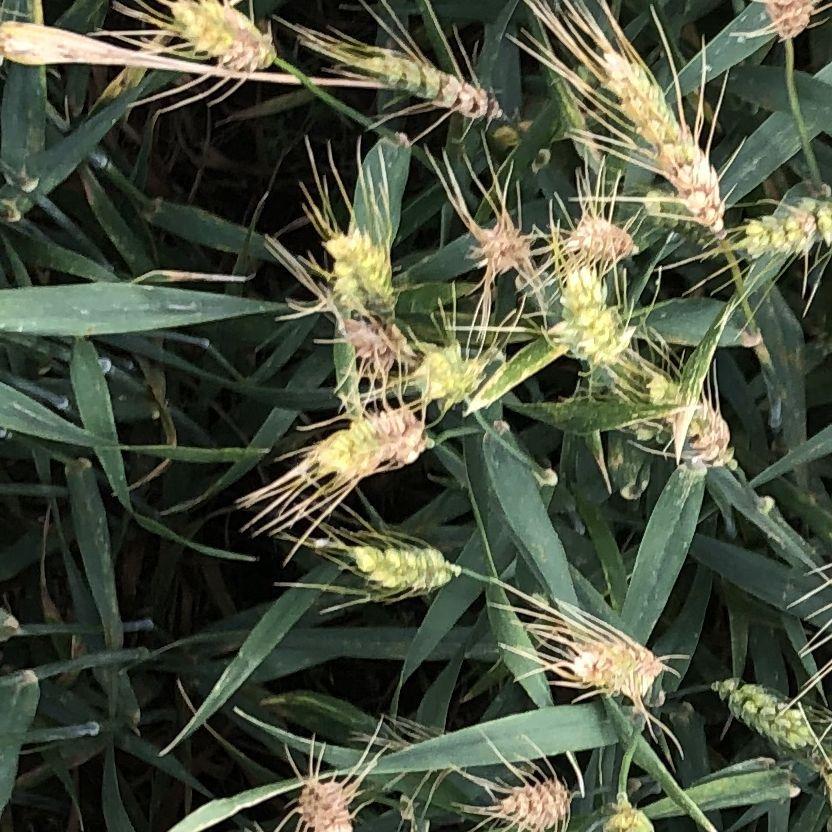

Supplement: Supplemental Information 2 [file peerj-cs-10-1948-s002.zip › data1/image0144.jpg]

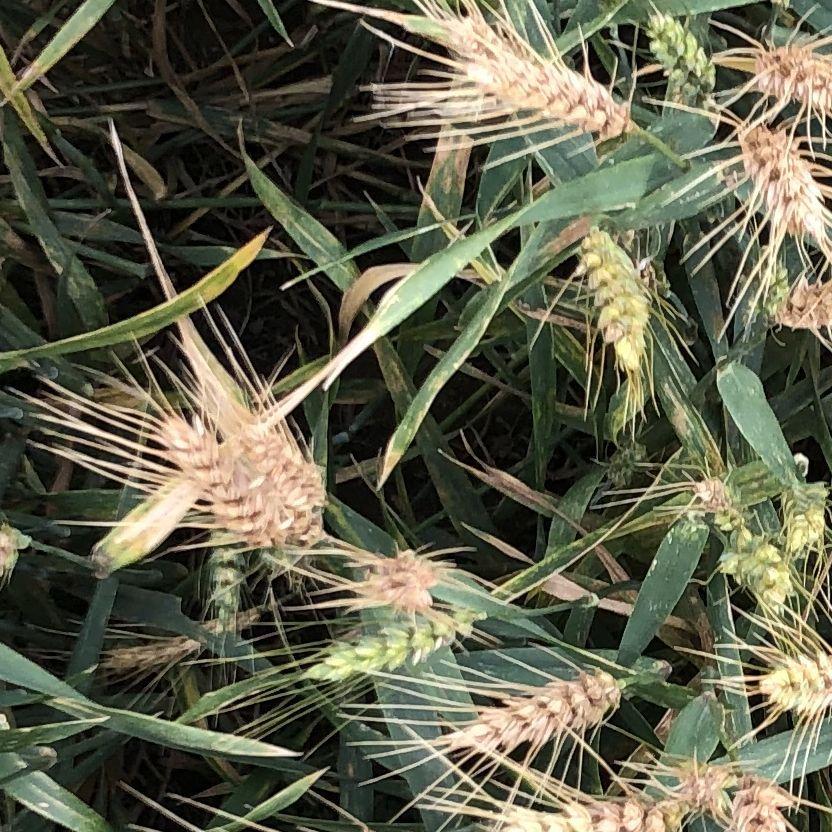

Supplement: Supplemental Information 2 [file peerj-cs-10-1948-s002.zip › data1/image0145.jpg]

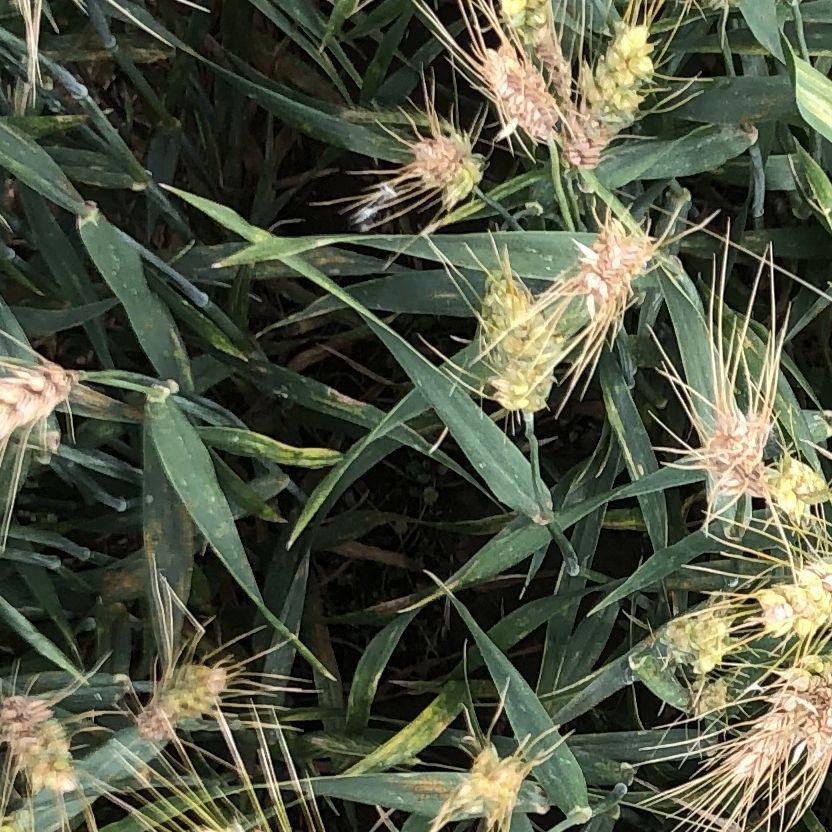

Supplement: Supplemental Information 2 [file peerj-cs-10-1948-s002.zip › data1/image0146.jpg]

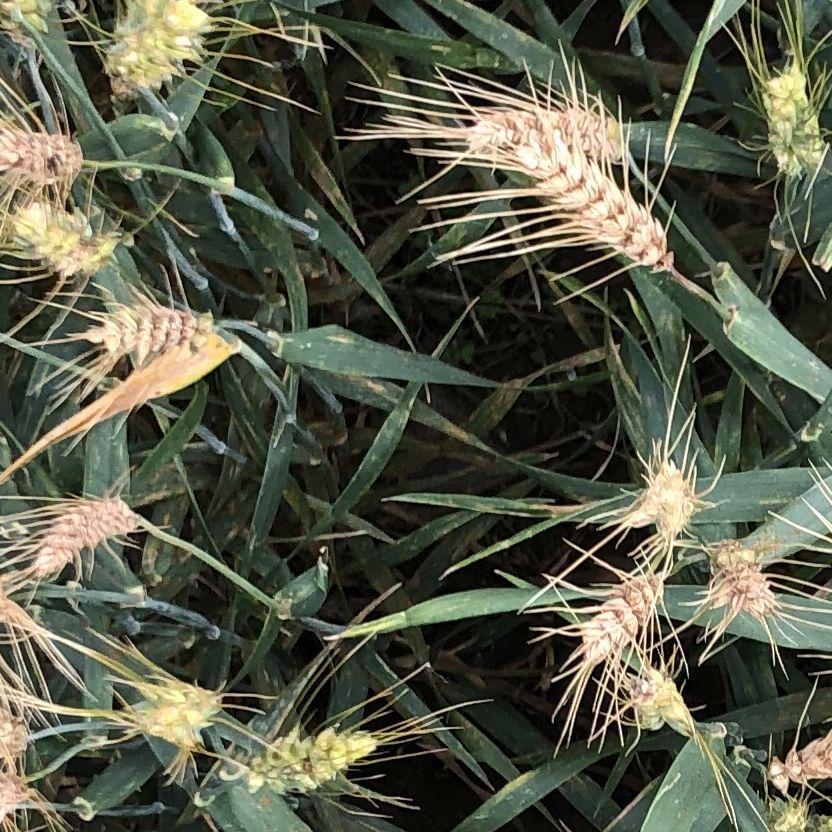

Supplement: Supplemental Information 2 [file peerj-cs-10-1948-s002.zip › data1/image0147.jpg]

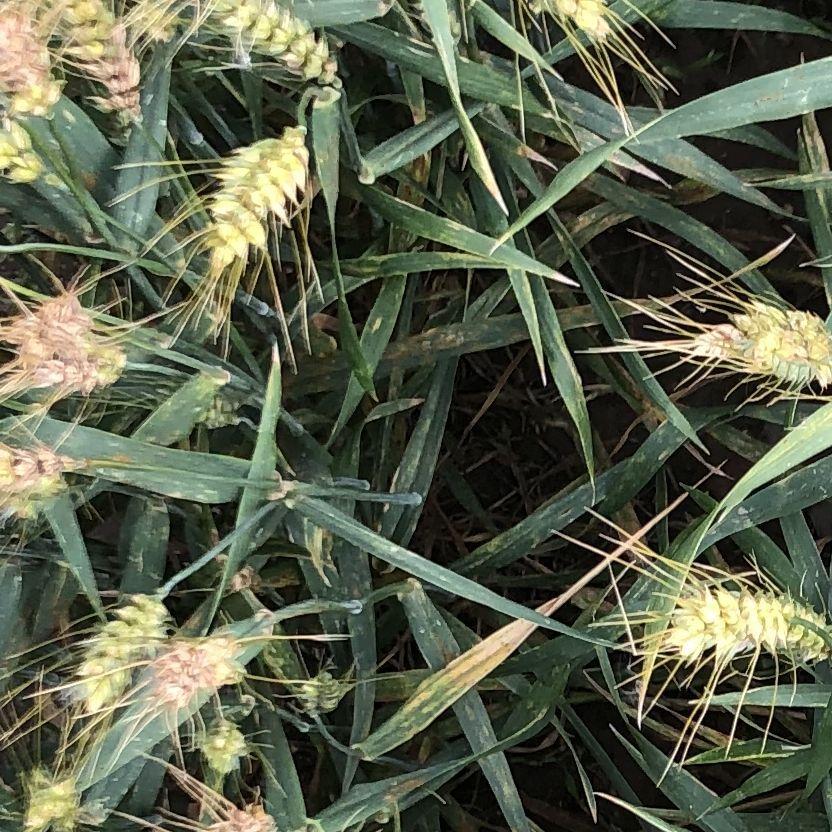

Supplement: Supplemental Information 2 [file peerj-cs-10-1948-s002.zip › data1/image0148.jpg]

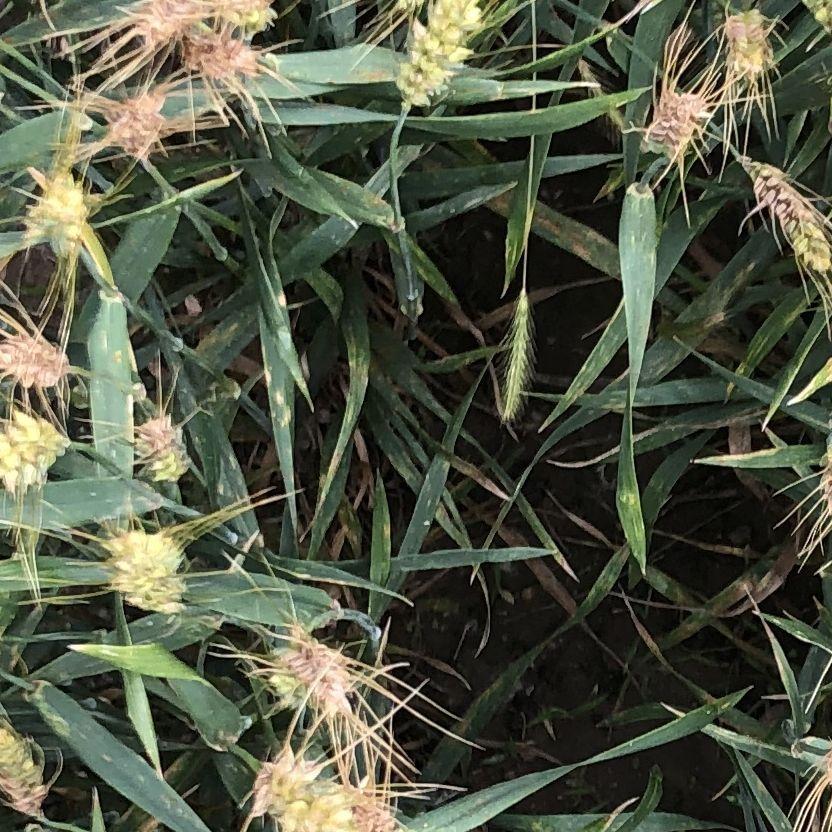

Supplement: Supplemental Information 2 [file peerj-cs-10-1948-s002.zip › data1/image0149.jpg]

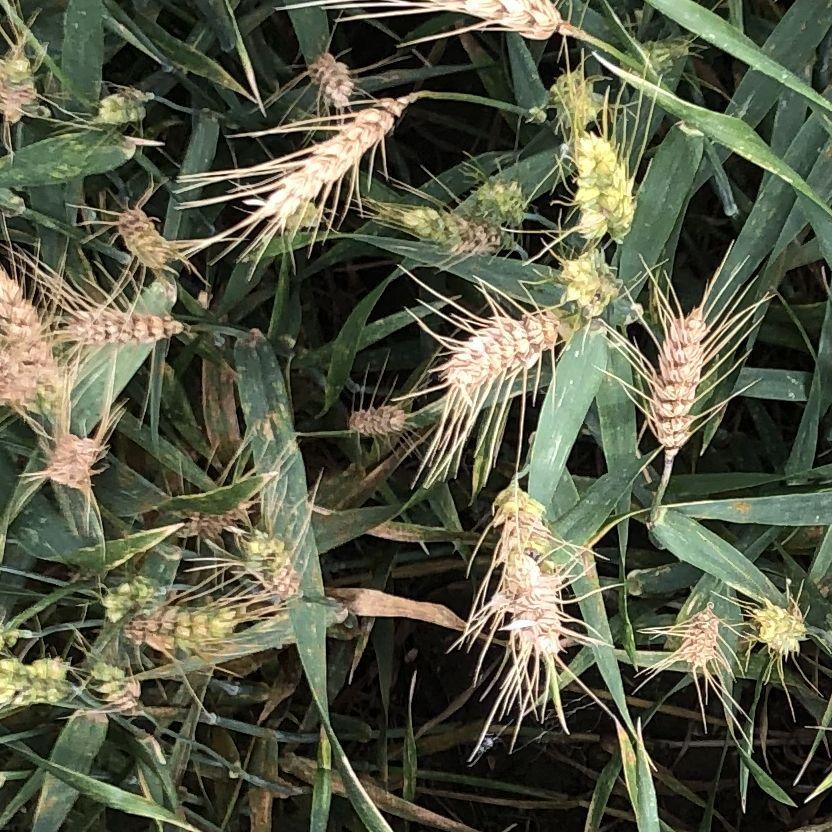

Supplement: Supplemental Information 2 [file peerj-cs-10-1948-s002.zip › data1/image0150.jpg]

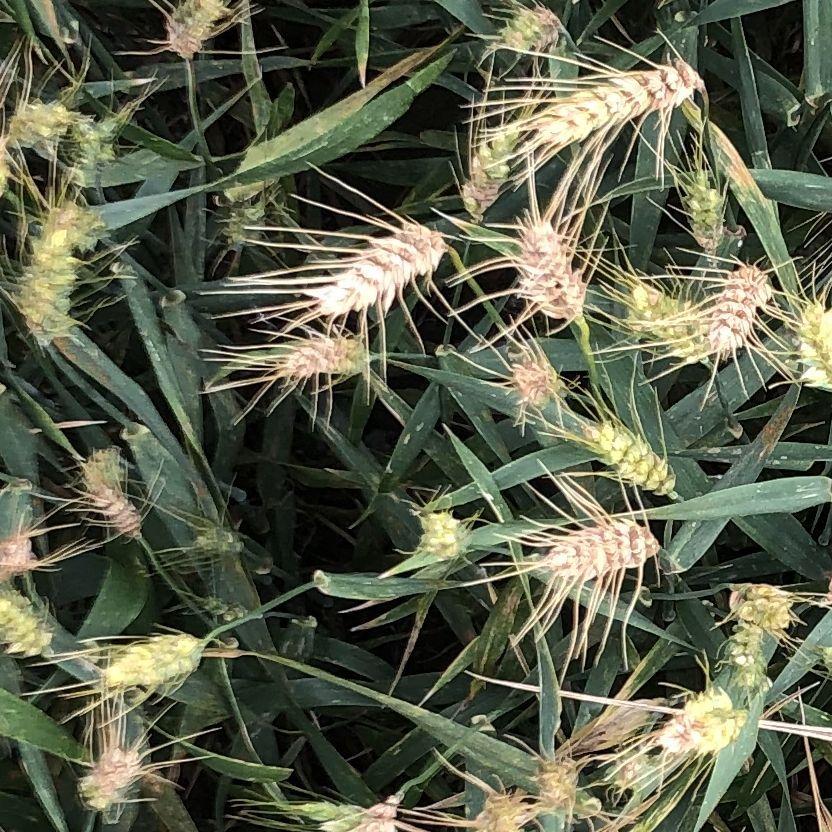

Supplement: Supplemental Information 2 [file peerj-cs-10-1948-s002.zip › data1/image0151.jpg]

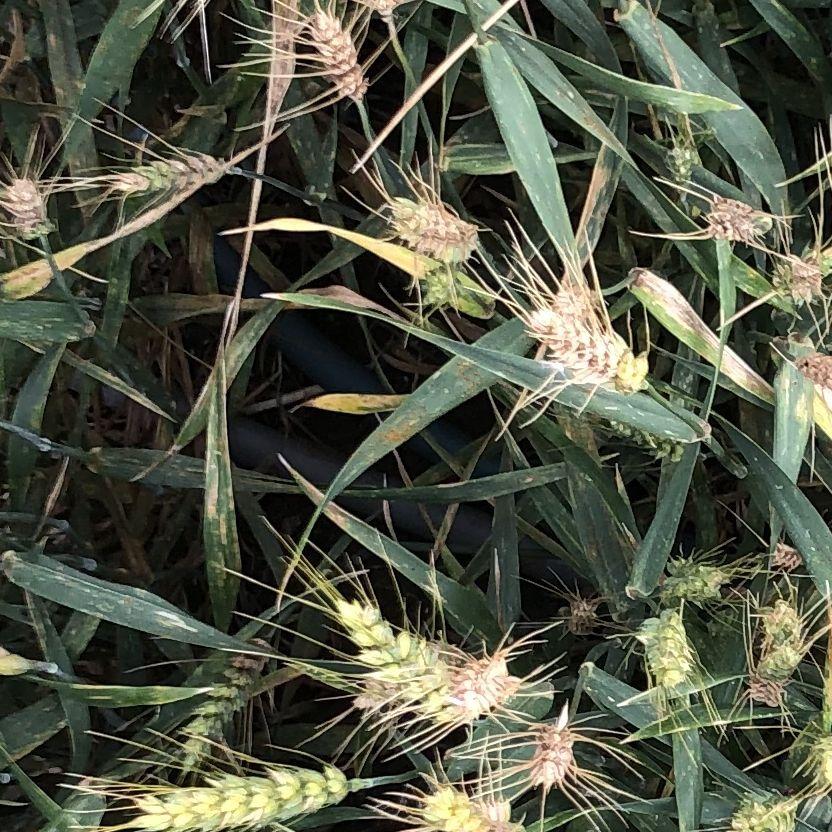

Supplement: Supplemental Information 2 [file peerj-cs-10-1948-s002.zip › data1/image0152.jpg]

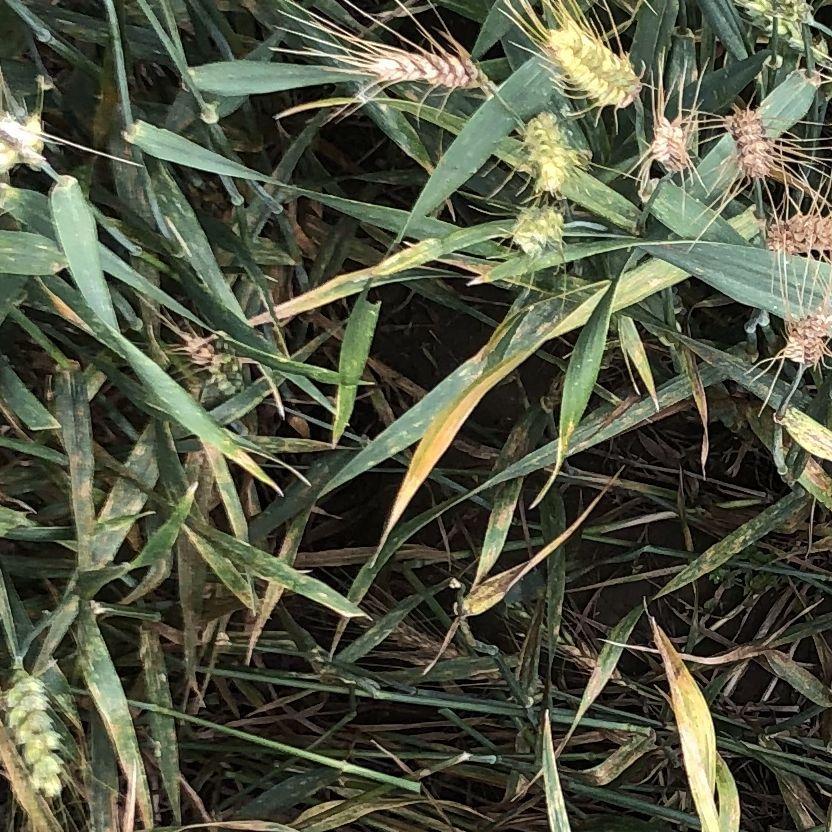

Supplement: Supplemental Information 2 [file peerj-cs-10-1948-s002.zip › data1/image0154.jpg]

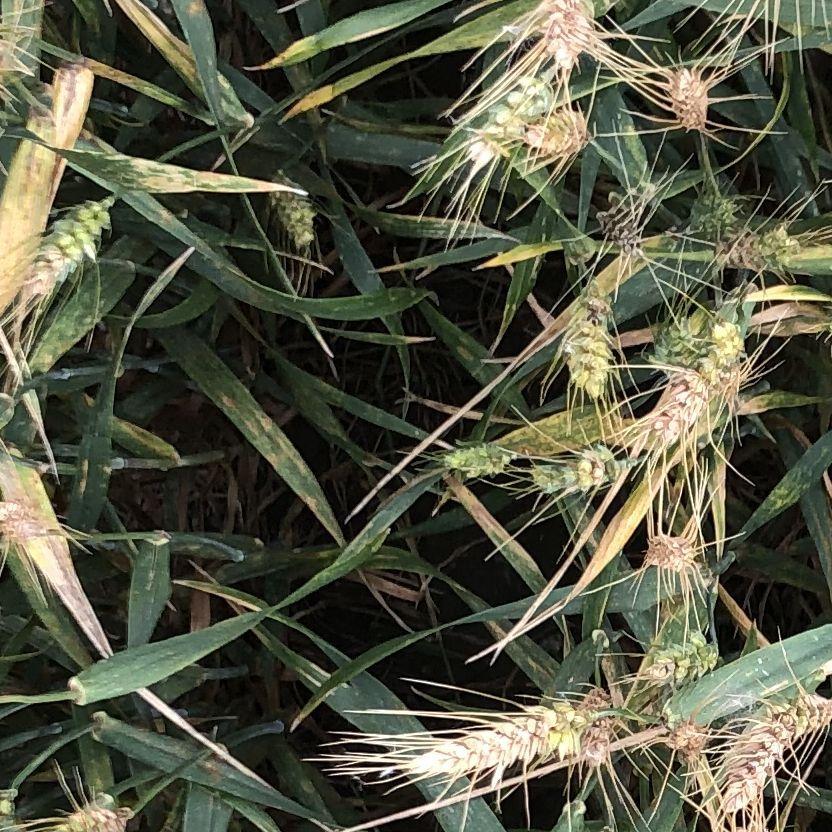

Supplement: Supplemental Information 2 [file peerj-cs-10-1948-s002.zip › data1/image0155.jpg]

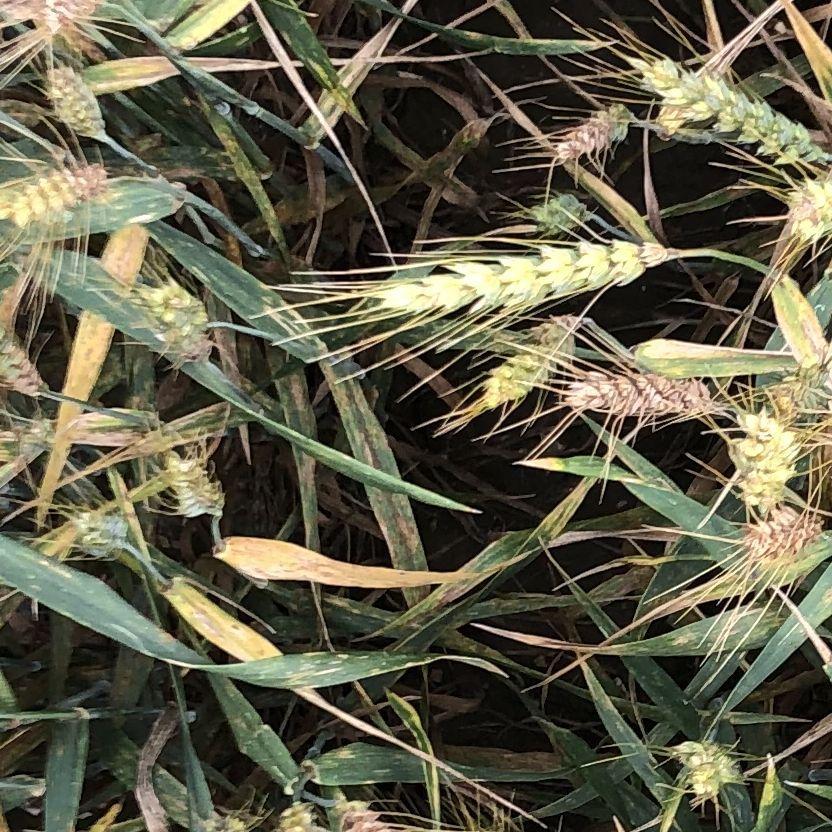

Supplement: Supplemental Information 2 [file peerj-cs-10-1948-s002.zip › data1/image0156.jpg]

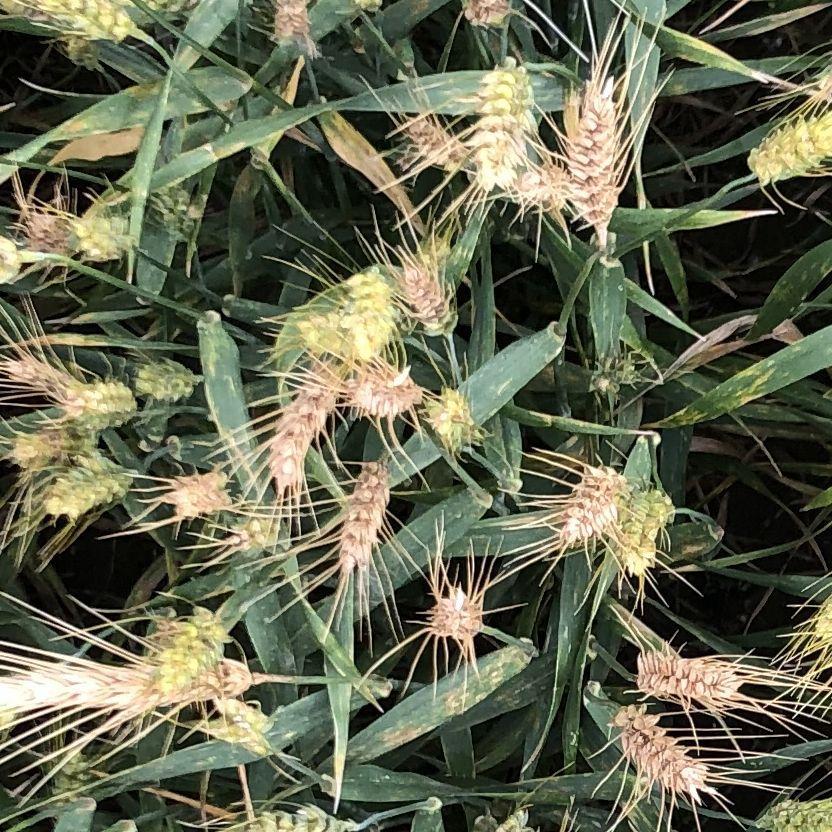

Supplement: Supplemental Information 2 [file peerj-cs-10-1948-s002.zip › data1/image0157.jpg]

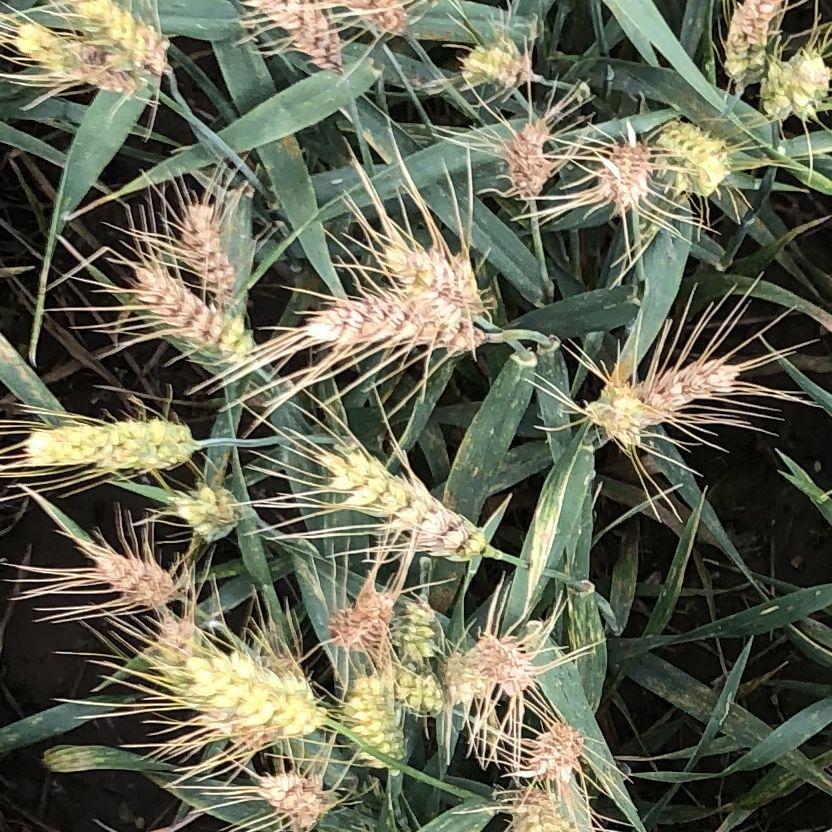

Supplement: Supplemental Information 2 [file peerj-cs-10-1948-s002.zip › data1/image0158.jpg]

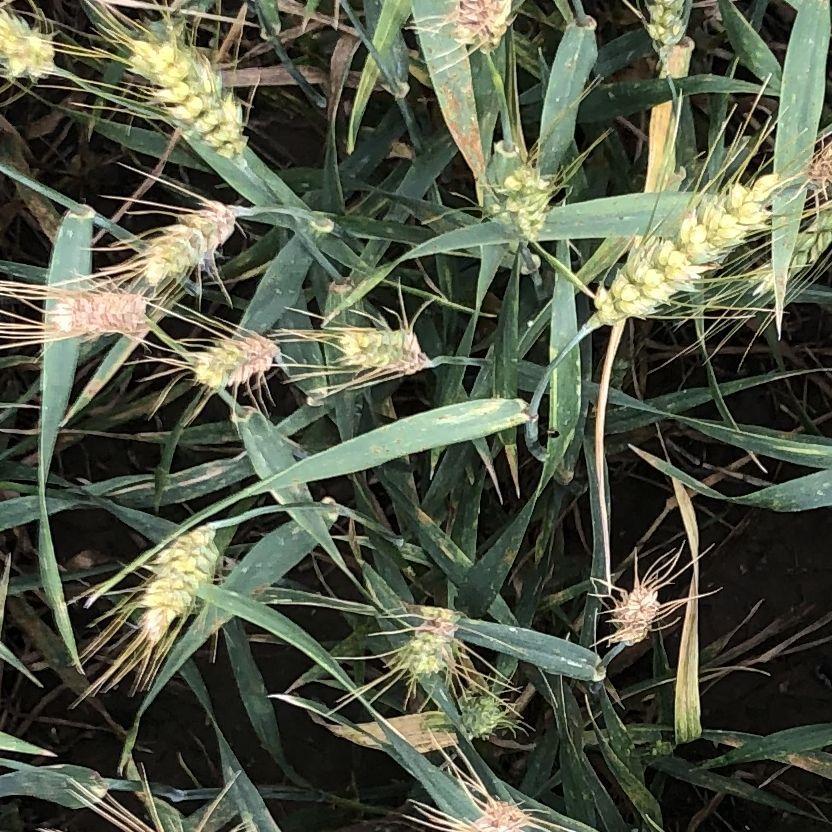

Supplement: Supplemental Information 2 [file peerj-cs-10-1948-s002.zip › data1/image0159.jpg]

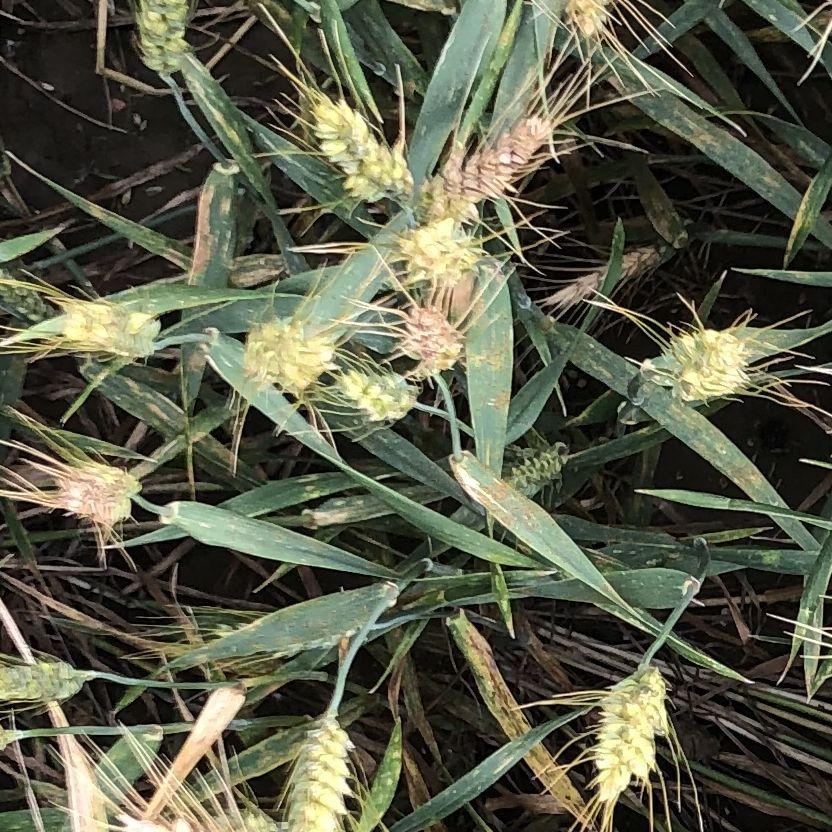

Supplement: Supplemental Information 2 [file peerj-cs-10-1948-s002.zip › data1/image0160.jpg]

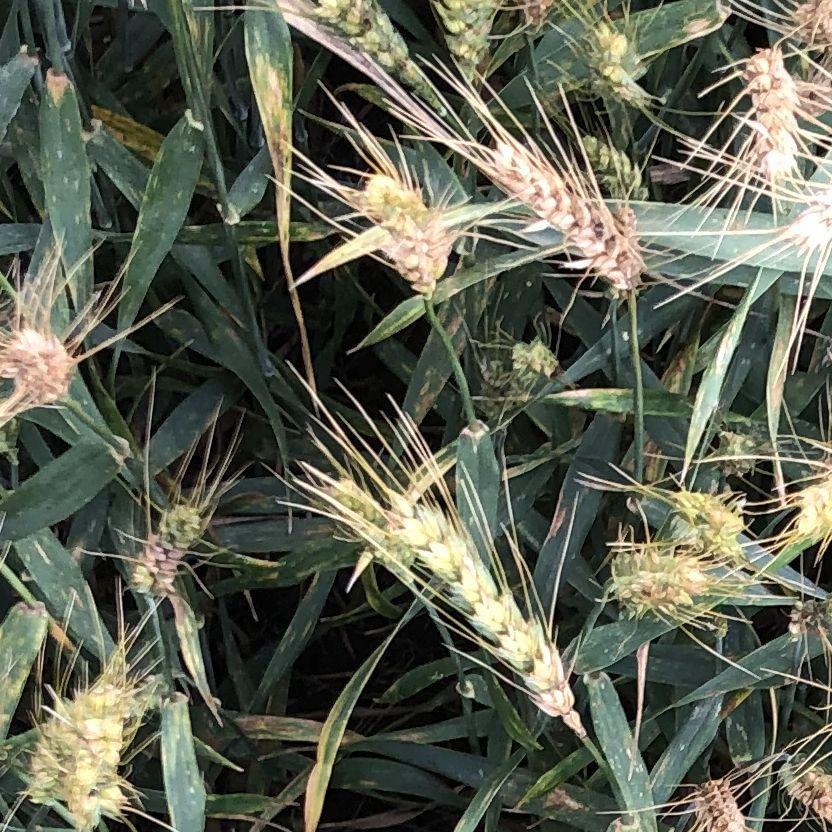

Supplement: Supplemental Information 2 [file peerj-cs-10-1948-s002.zip › data1/image0161.jpg]

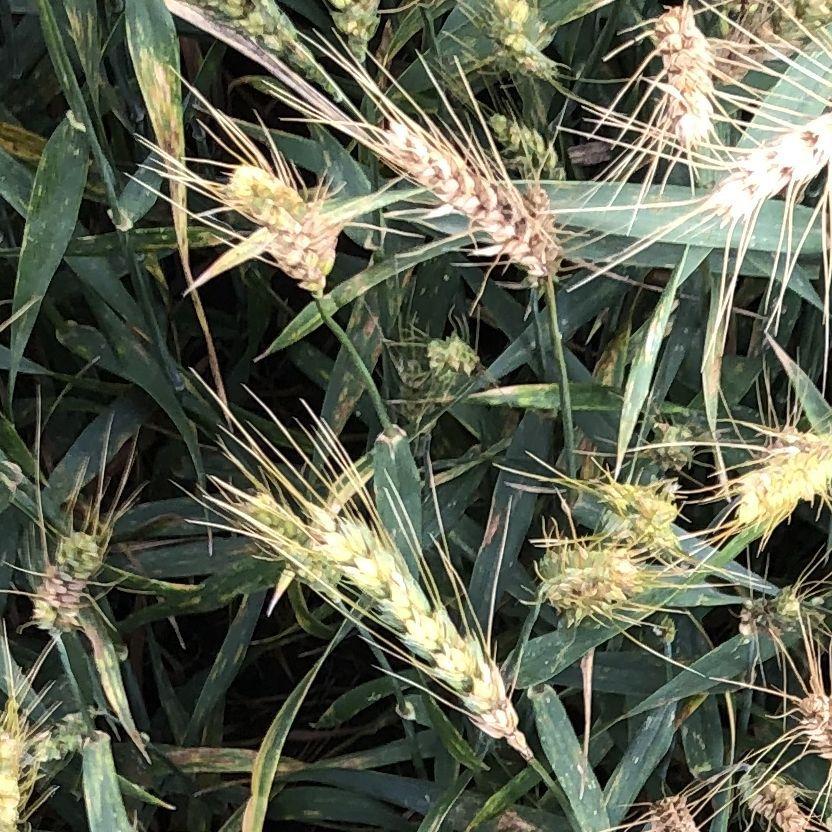

Supplement: Supplemental Information 3 [file peerj-cs-10-1948-s003.zip › data2/image0162.jpg]

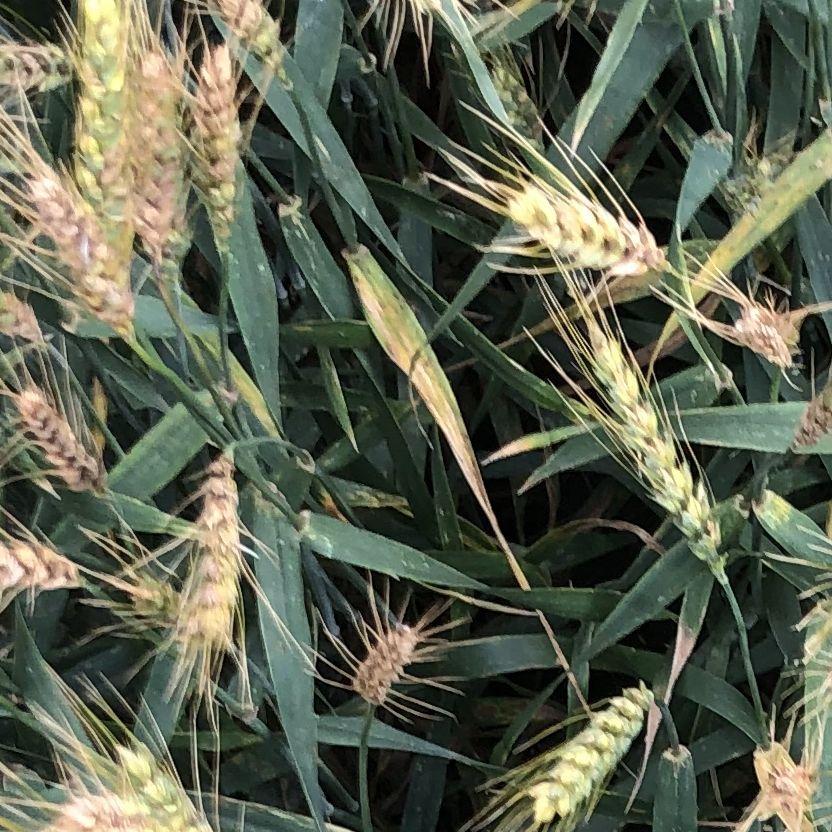

Supplement: Supplemental Information 3 [file peerj-cs-10-1948-s003.zip › data2/image0163.jpg]

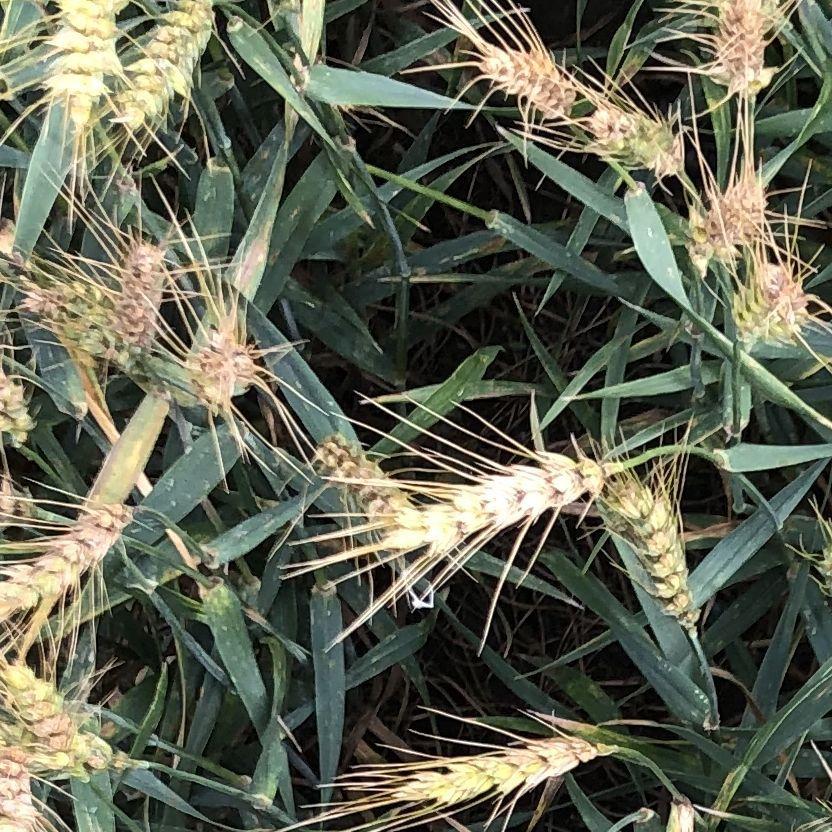

Supplement: Supplemental Information 3 [file peerj-cs-10-1948-s003.zip › data2/image0164.jpg]

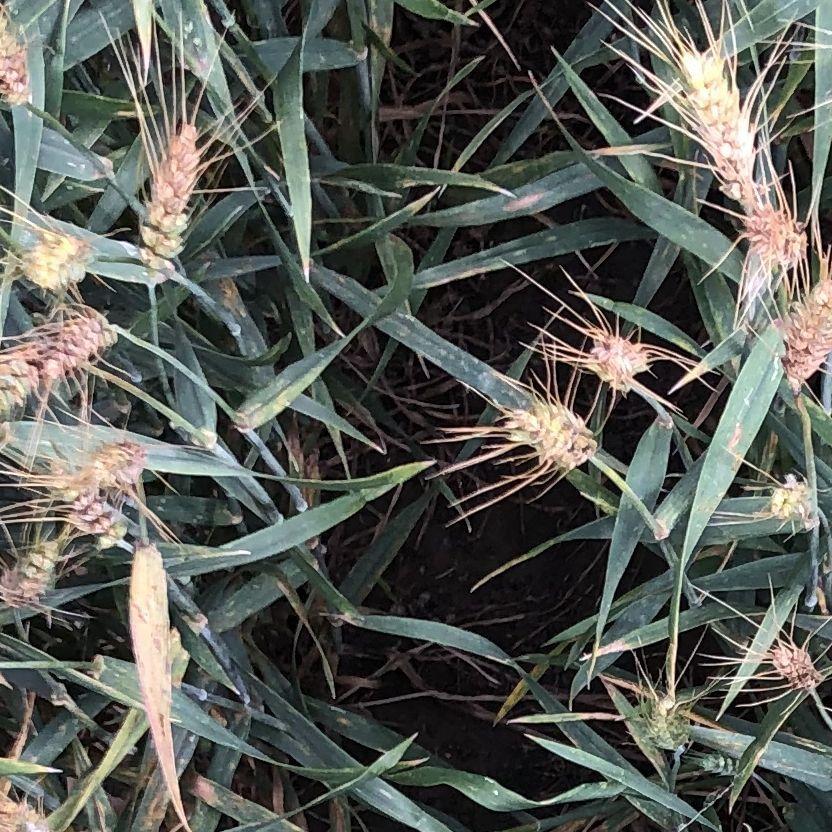

Supplement: Supplemental Information 3 [file peerj-cs-10-1948-s003.zip › data2/image0165.jpg]

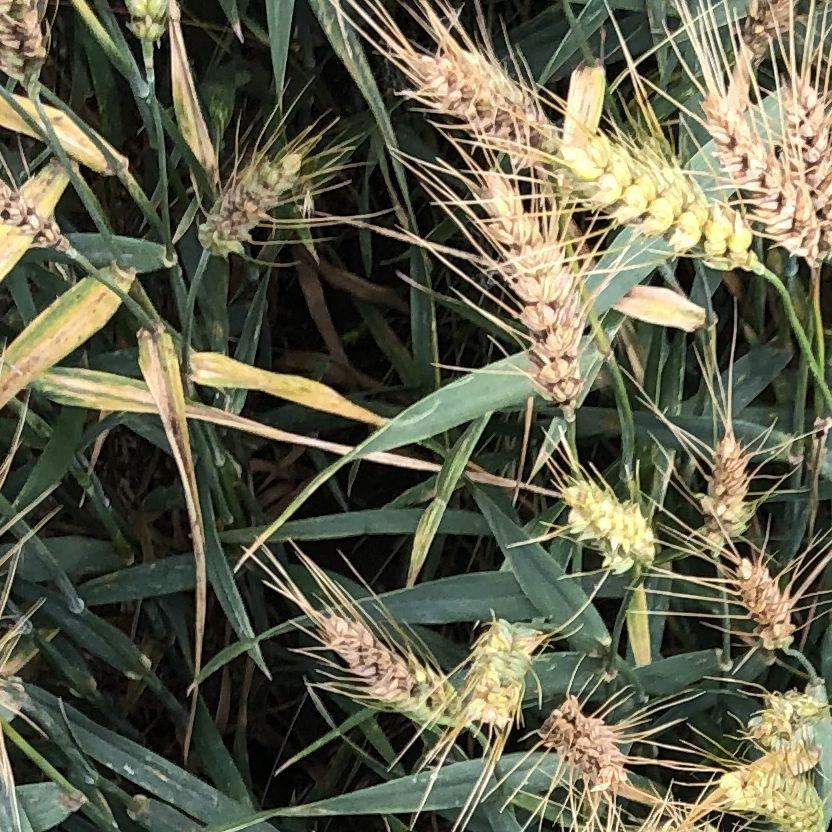

Supplement: Supplemental Information 3 [file peerj-cs-10-1948-s003.zip › data2/image0166.jpg]

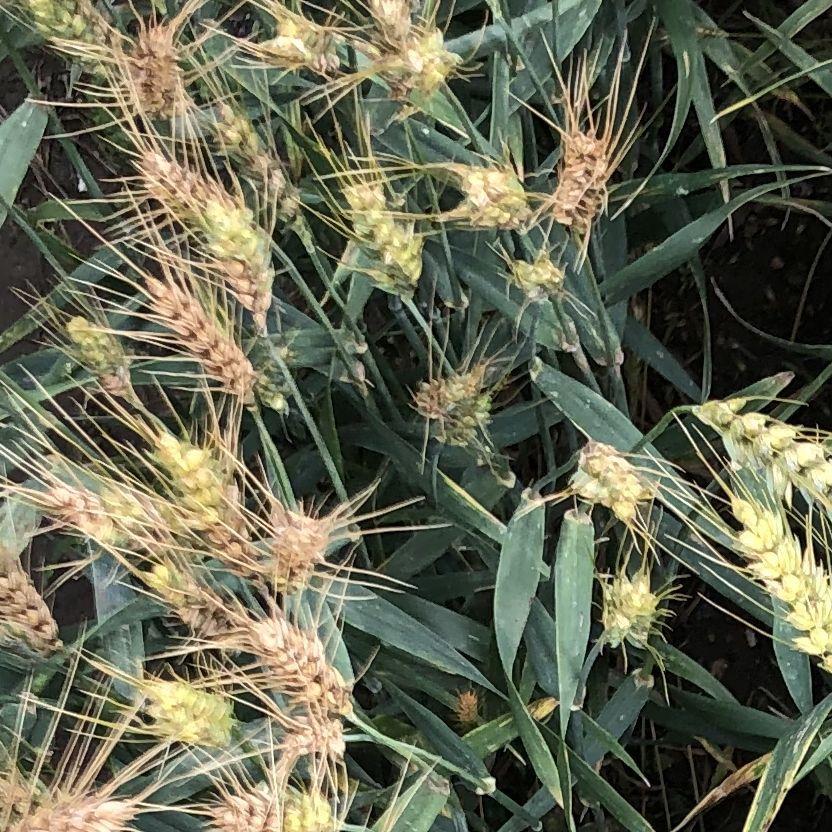

Supplement: Supplemental Information 3 [file peerj-cs-10-1948-s003.zip › data2/image0168.jpg]

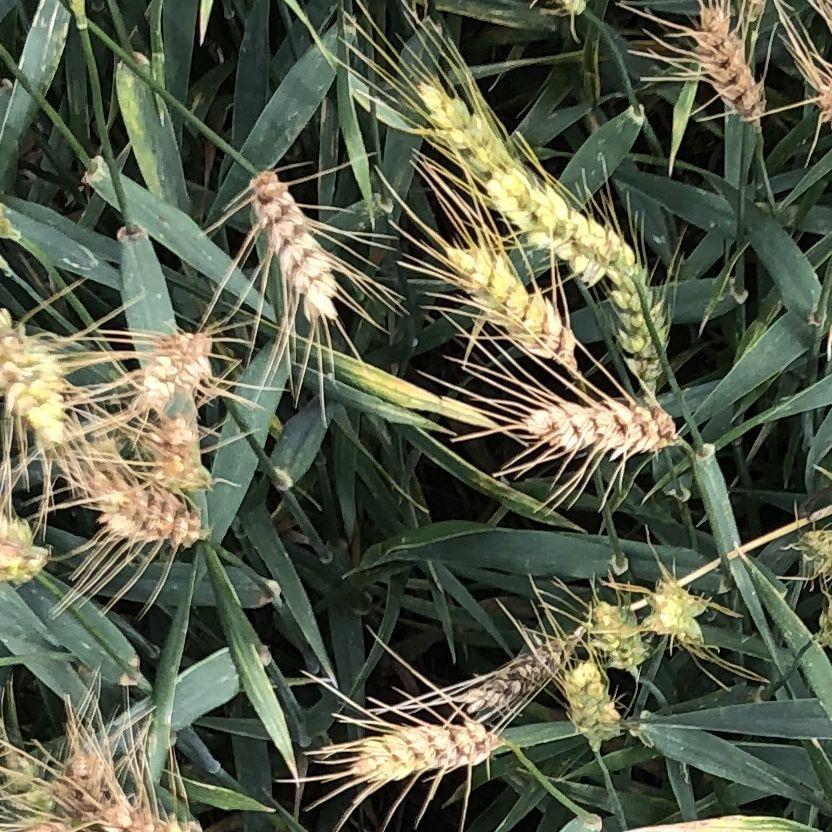

Supplement: Supplemental Information 3 [file peerj-cs-10-1948-s003.zip › data2/image0170.jpg]

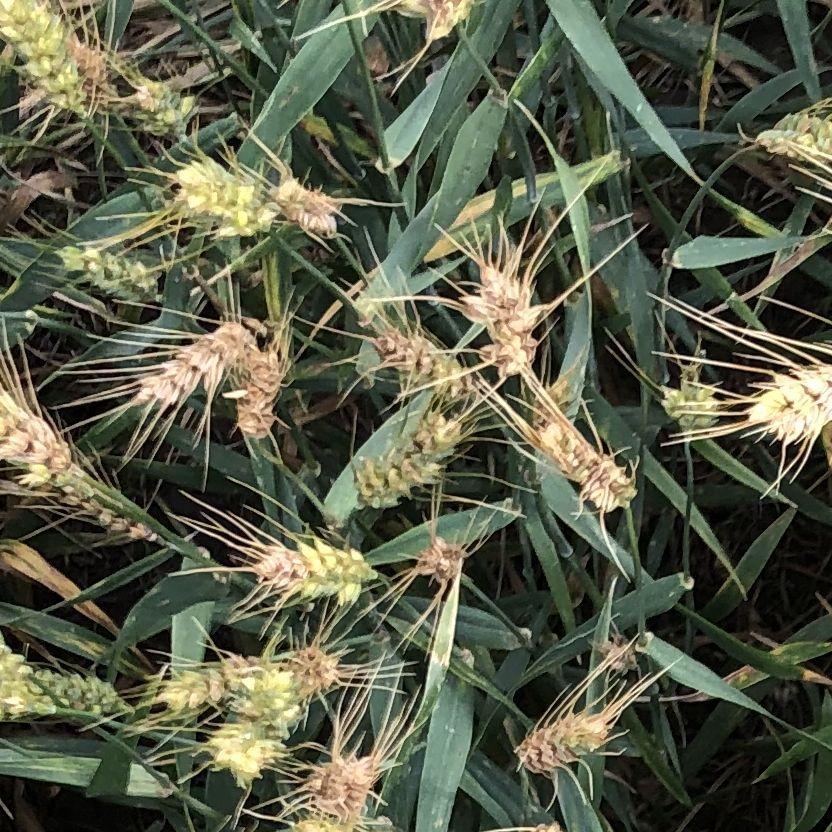

Supplement: Supplemental Information 3 [file peerj-cs-10-1948-s003.zip › data2/image0171.jpg]

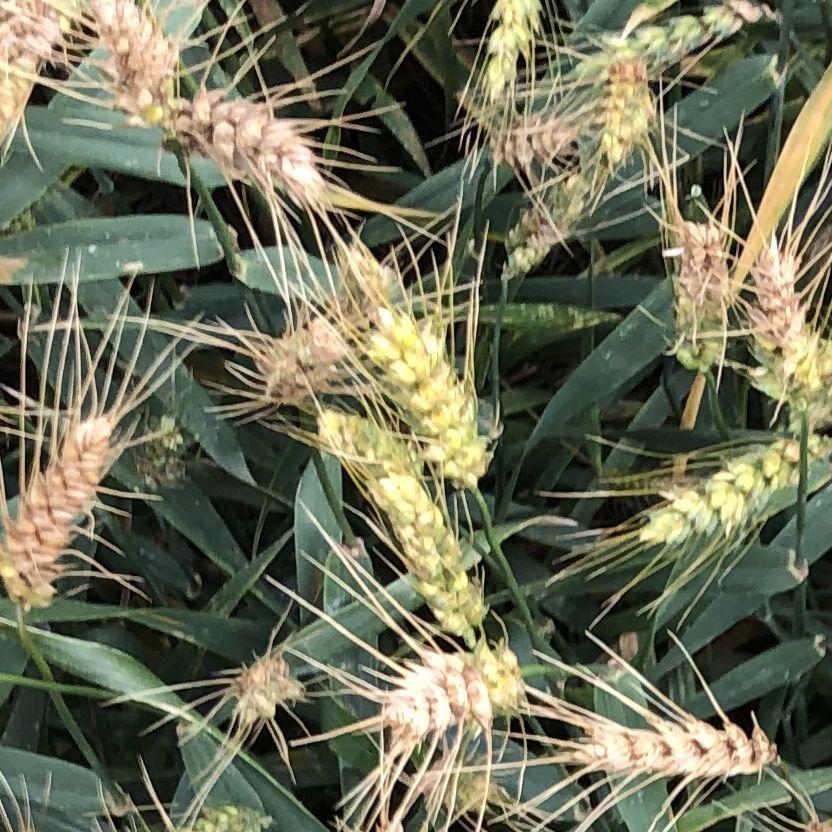

Supplement: Supplemental Information 3 [file peerj-cs-10-1948-s003.zip › data2/image0173.jpg]

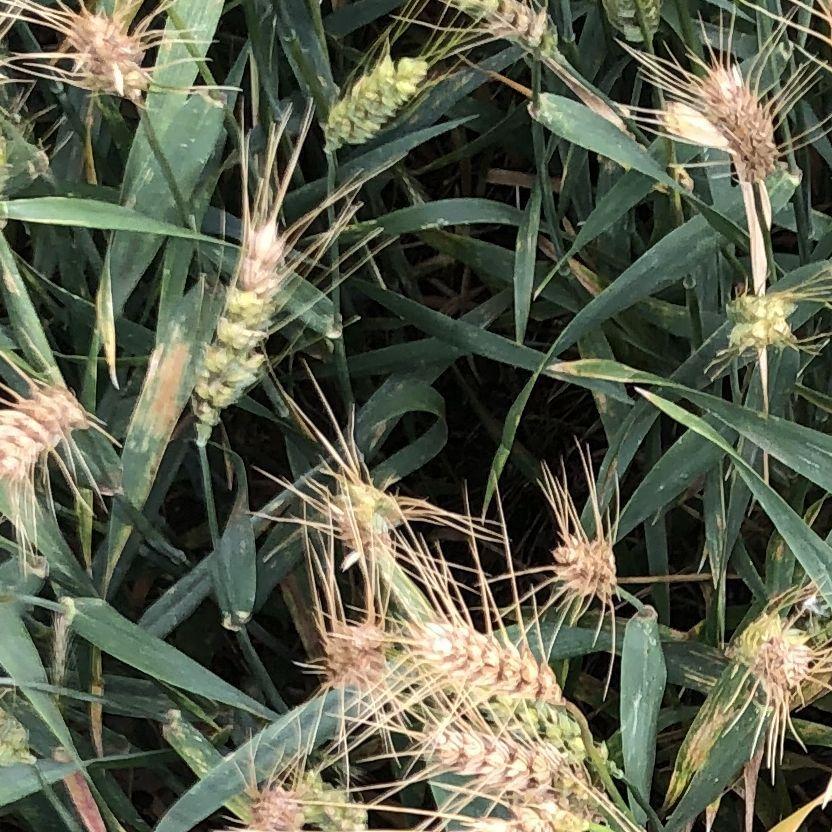

Supplement: Supplemental Information 3 [file peerj-cs-10-1948-s003.zip › data2/image0174.jpg]

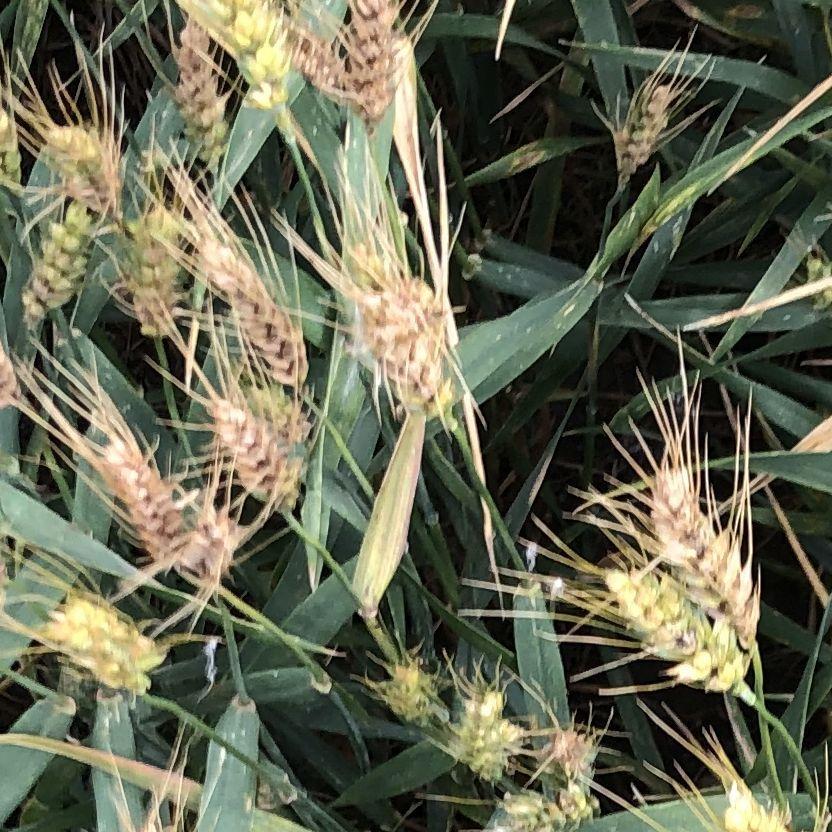

Supplement: Supplemental Information 3 [file peerj-cs-10-1948-s003.zip › data2/image0175.jpg]

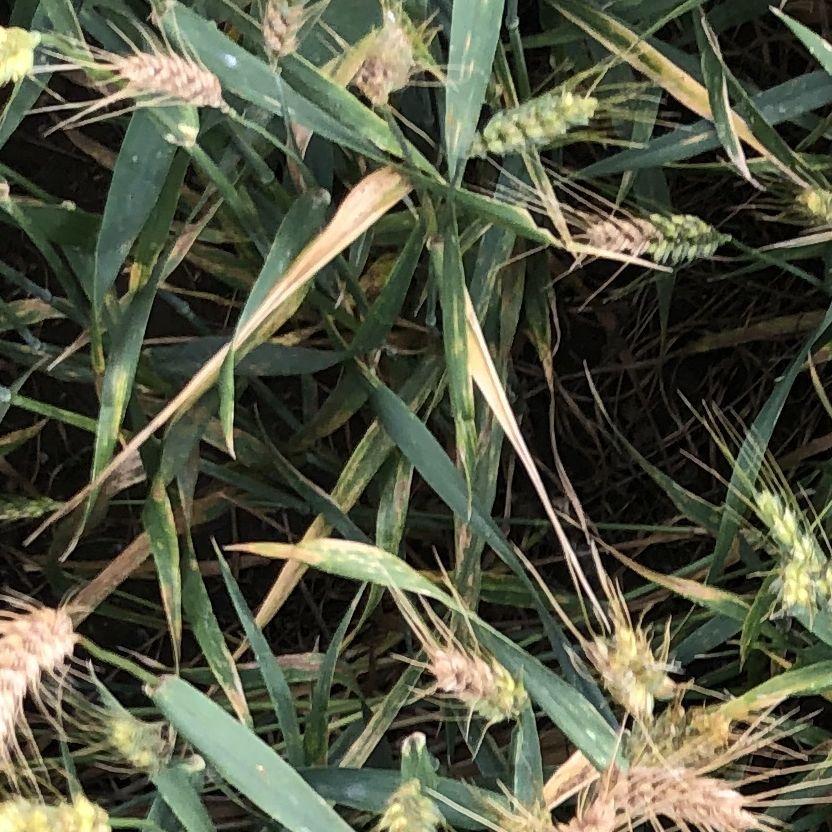

Supplement: Supplemental Information 3 [file peerj-cs-10-1948-s003.zip › data2/image0176.jpg]

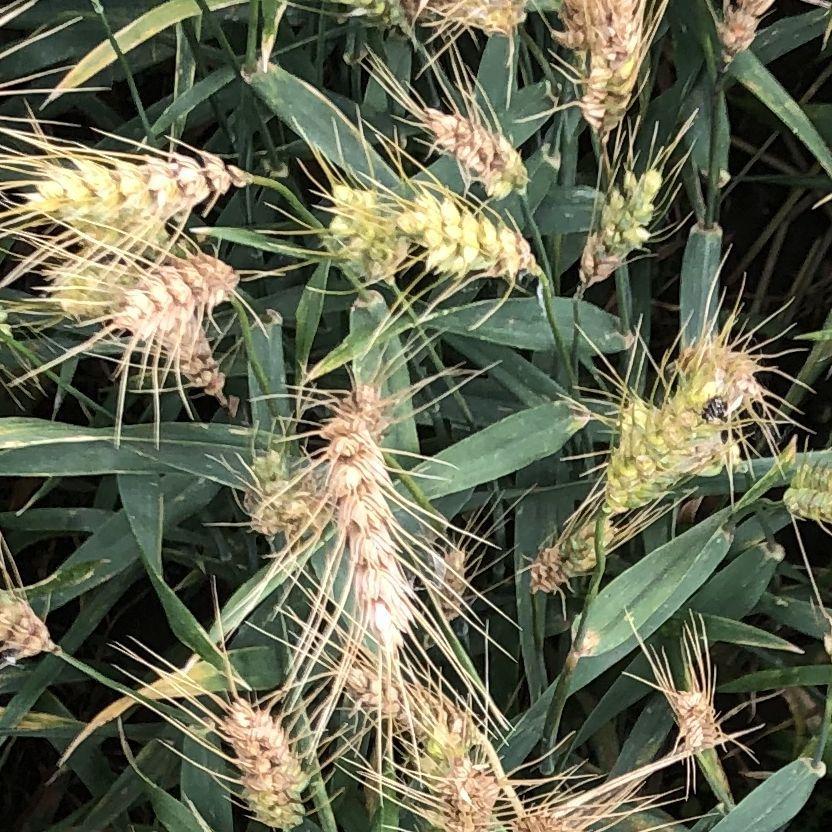

Supplement: Supplemental Information 3 [file peerj-cs-10-1948-s003.zip › data2/image0177.jpg]

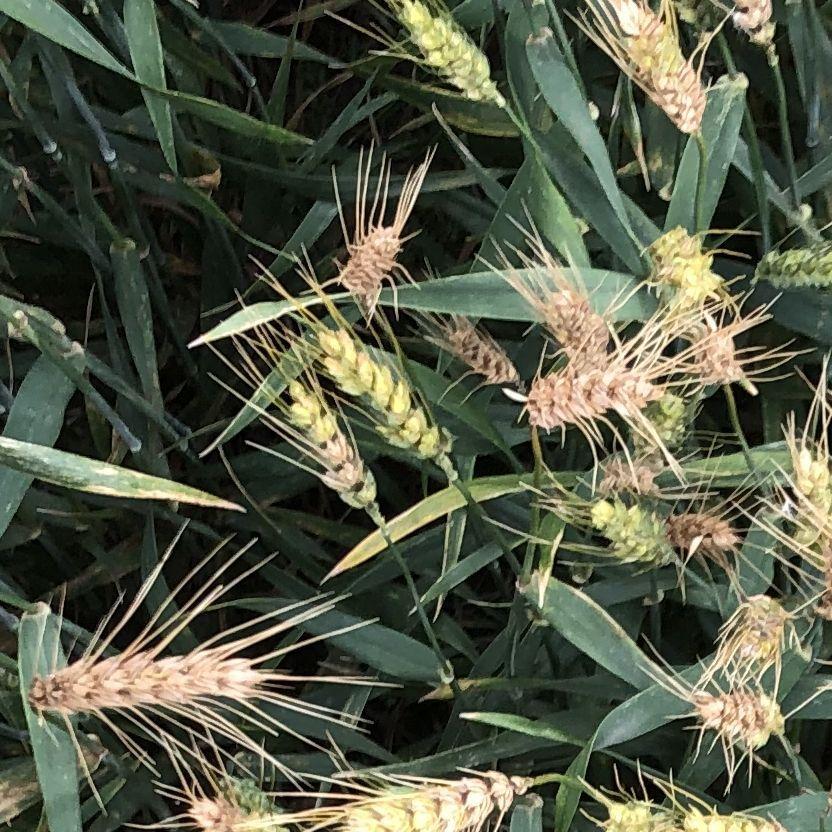

Supplement: Supplemental Information 3 [file peerj-cs-10-1948-s003.zip › data2/image0178.jpg]

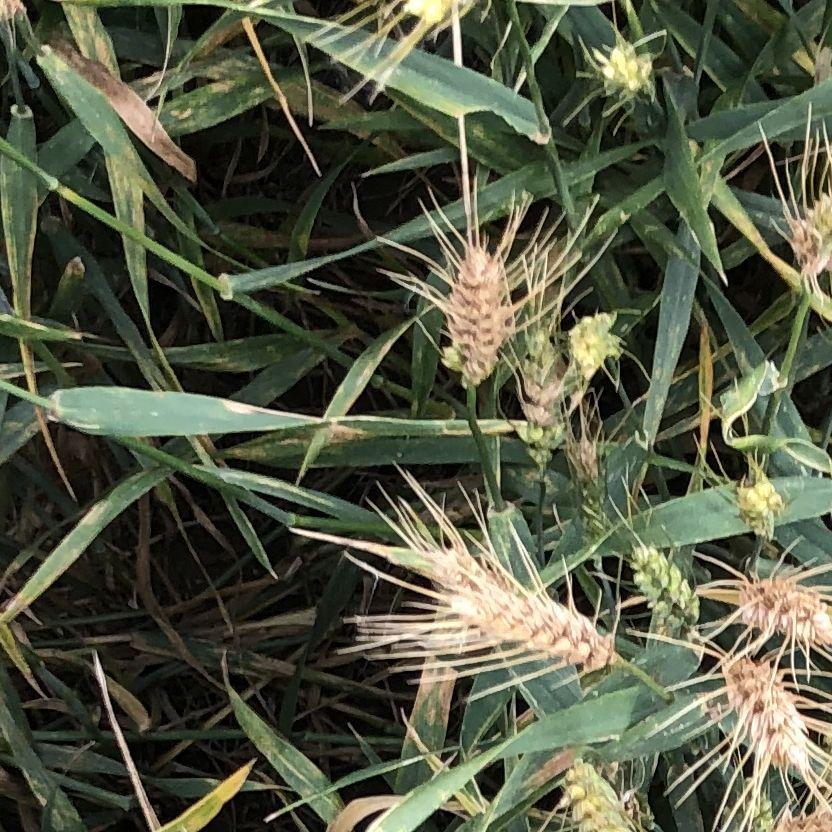

Supplement: Supplemental Information 3 [file peerj-cs-10-1948-s003.zip › data2/image0180.jpg]

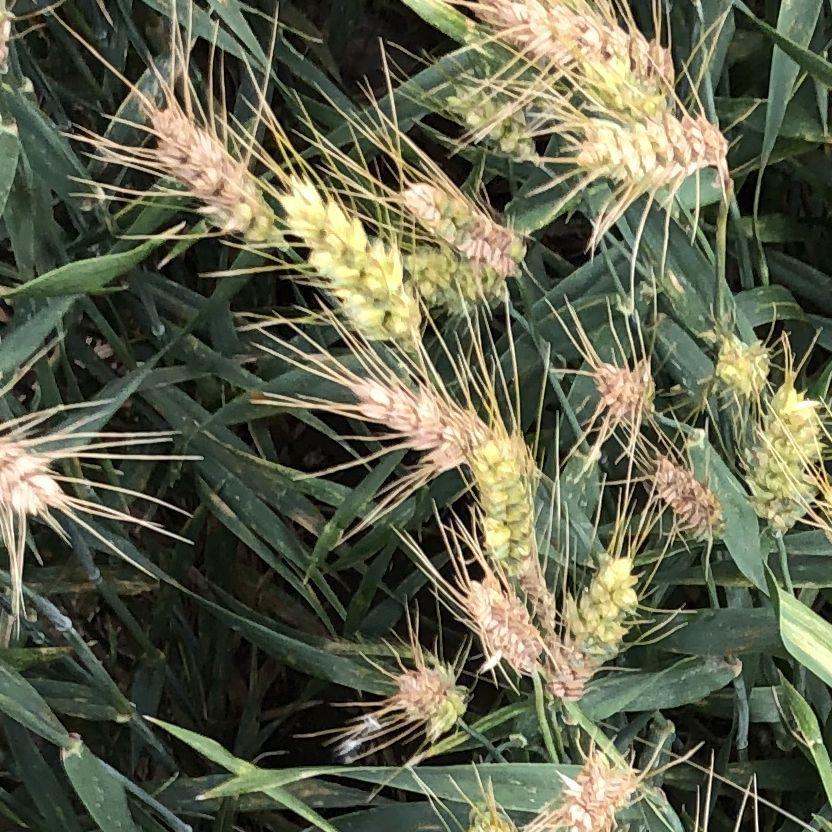

Supplement: Supplemental Information 3 [file peerj-cs-10-1948-s003.zip › data2/image0181.jpg]

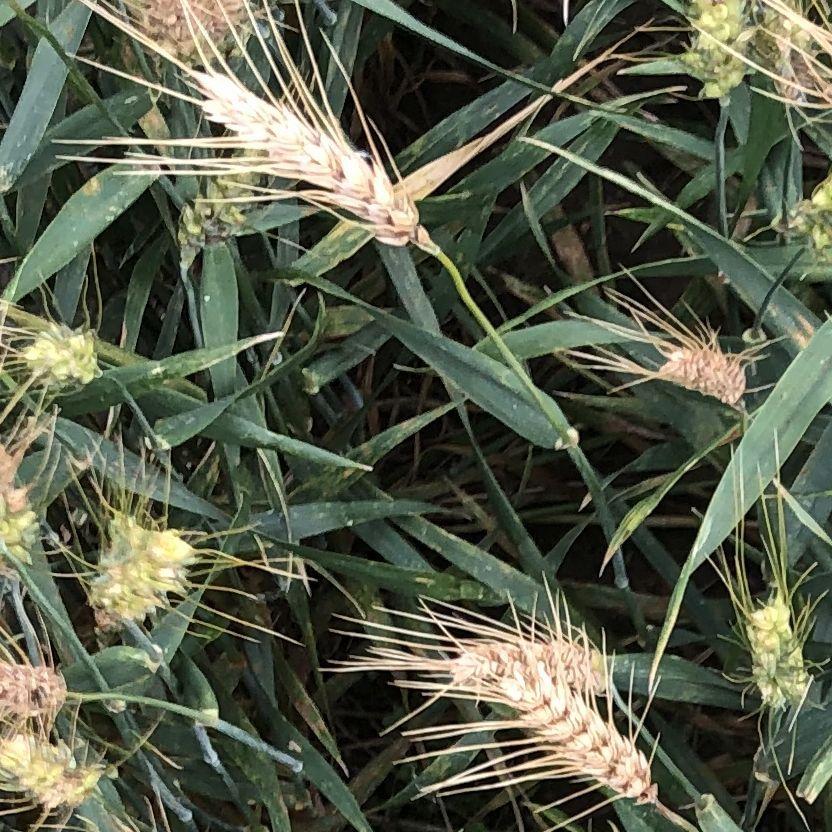

Supplement: Supplemental Information 3 [file peerj-cs-10-1948-s003.zip › data2/image0182.jpg]

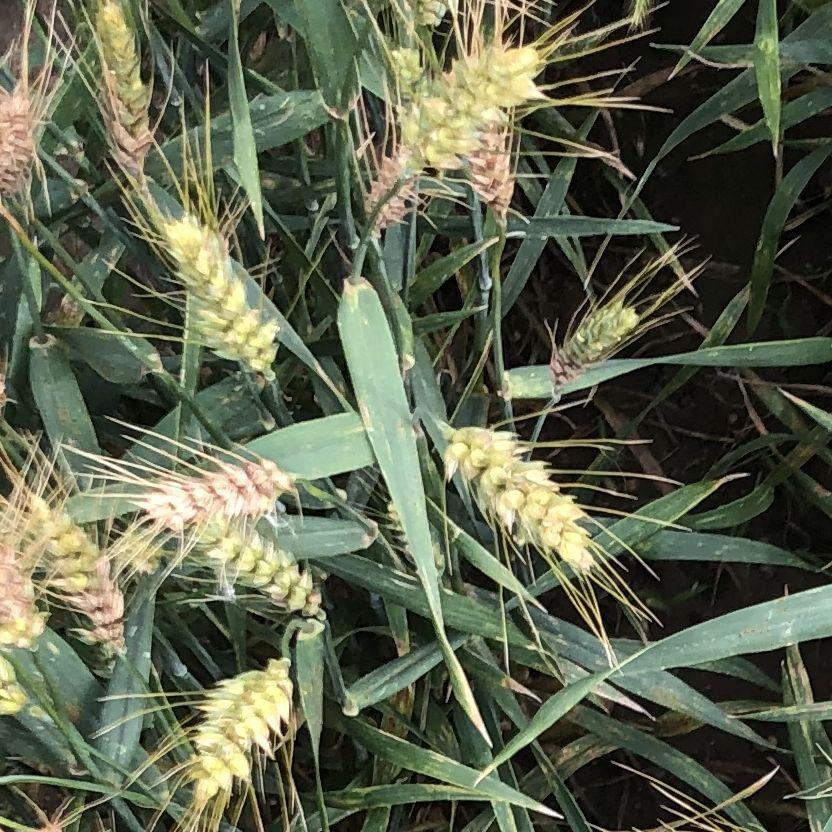

Supplement: Supplemental Information 3 [file peerj-cs-10-1948-s003.zip › data2/image0183.jpg]

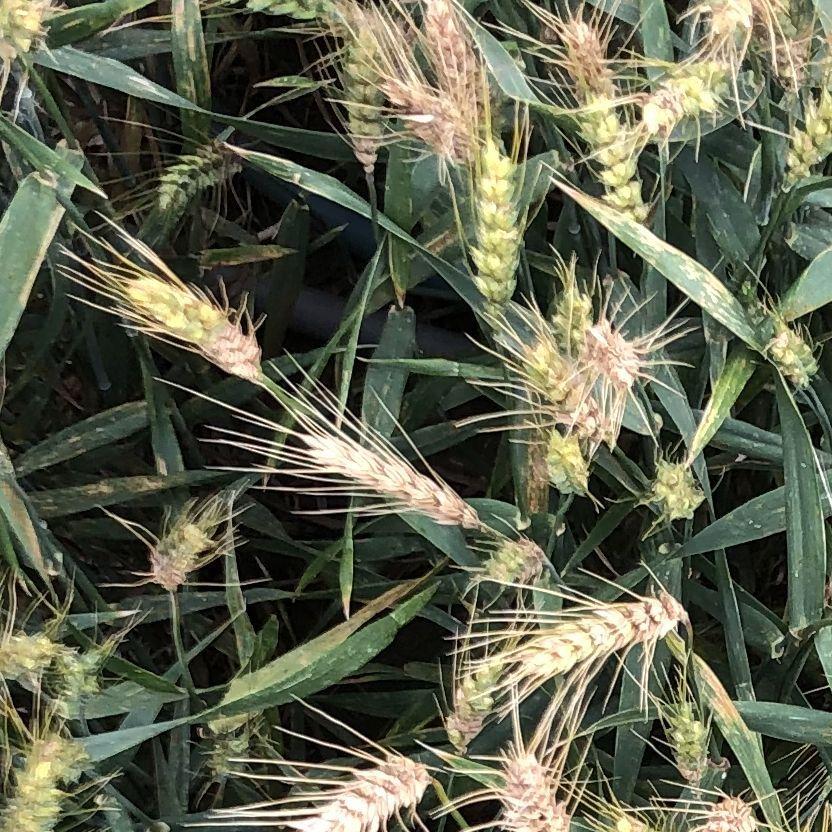

Supplement: Supplemental Information 3 [file peerj-cs-10-1948-s003.zip › data2/image0184.jpg]

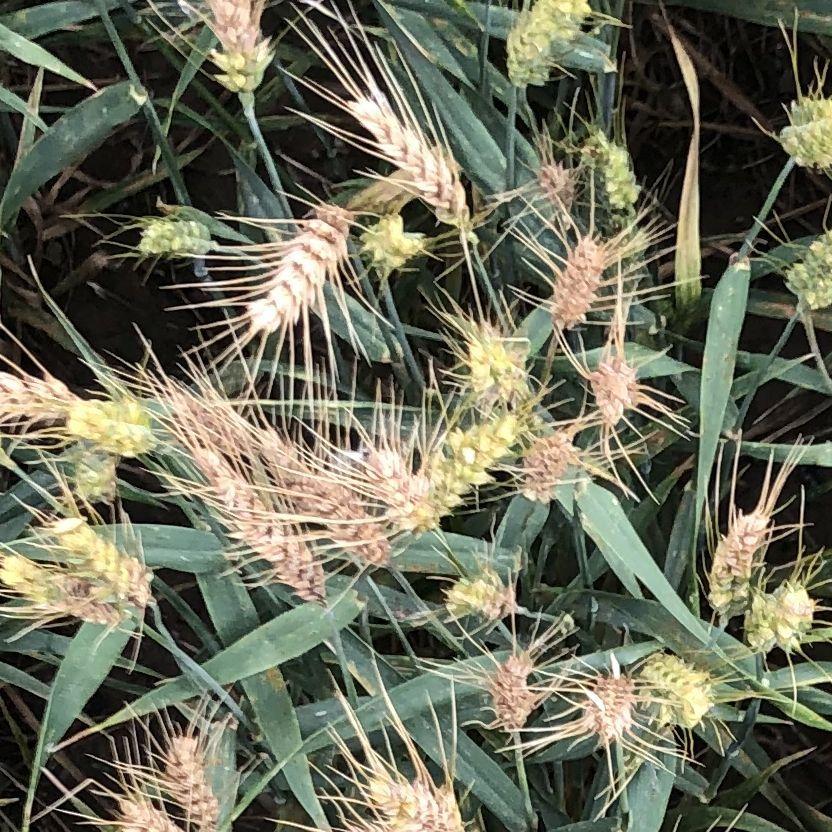

Supplement: Supplemental Information 3 [file peerj-cs-10-1948-s003.zip › data2/image0188.jpg]

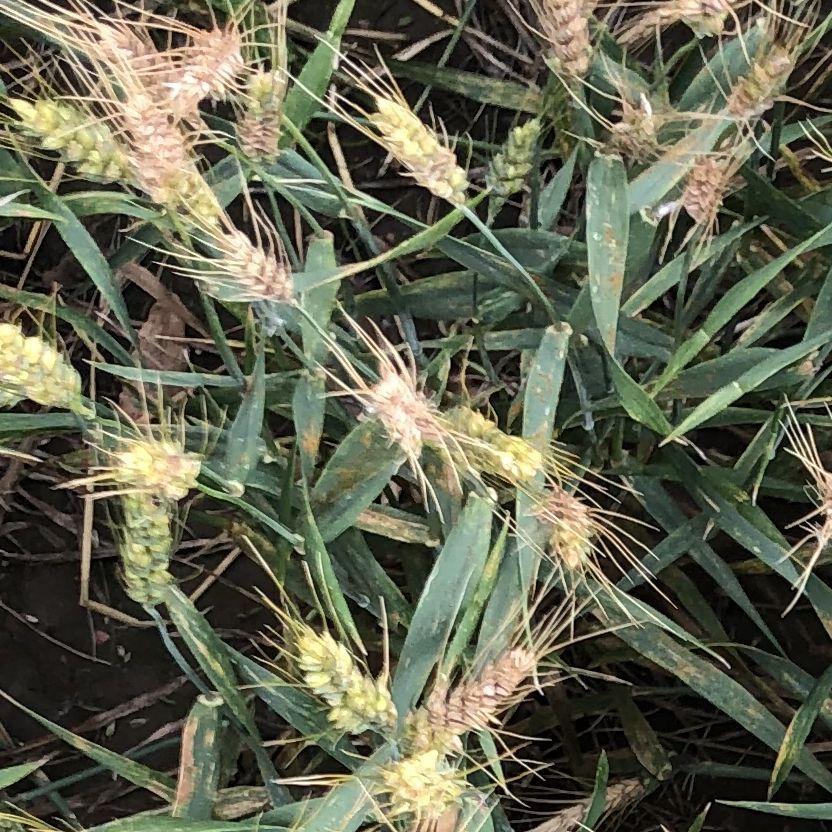

Supplement: Supplemental Information 3 [file peerj-cs-10-1948-s003.zip › data2/image0190.jpg]

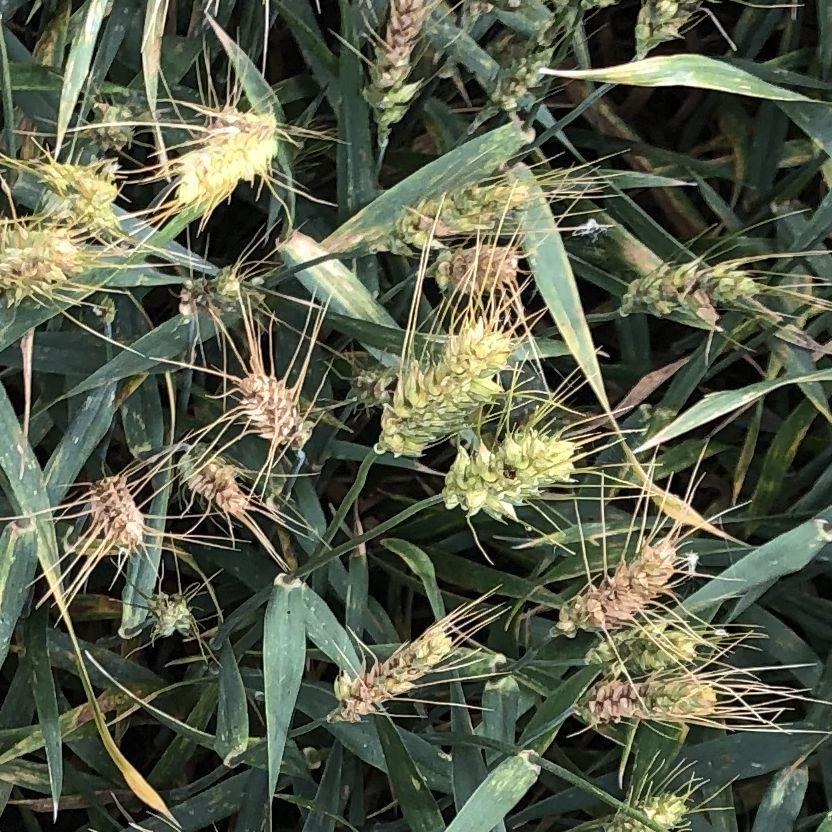

Supplement: Supplemental Information 3 [file peerj-cs-10-1948-s003.zip › data2/image0191.jpg]

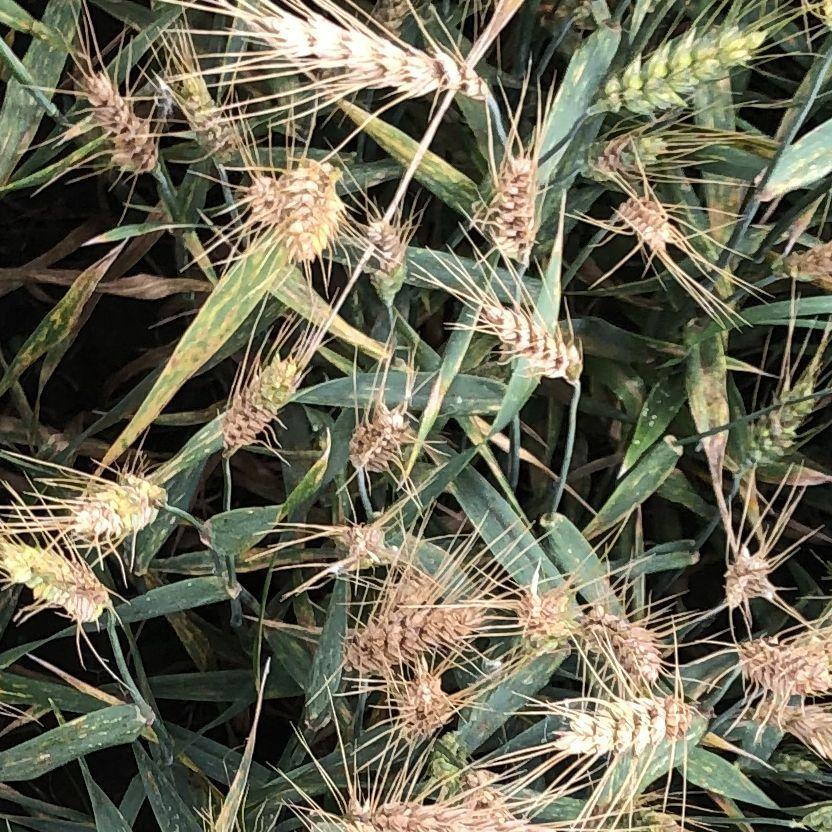

Supplement: Supplemental Information 3 [file peerj-cs-10-1948-s003.zip › data2/image0192.jpg]

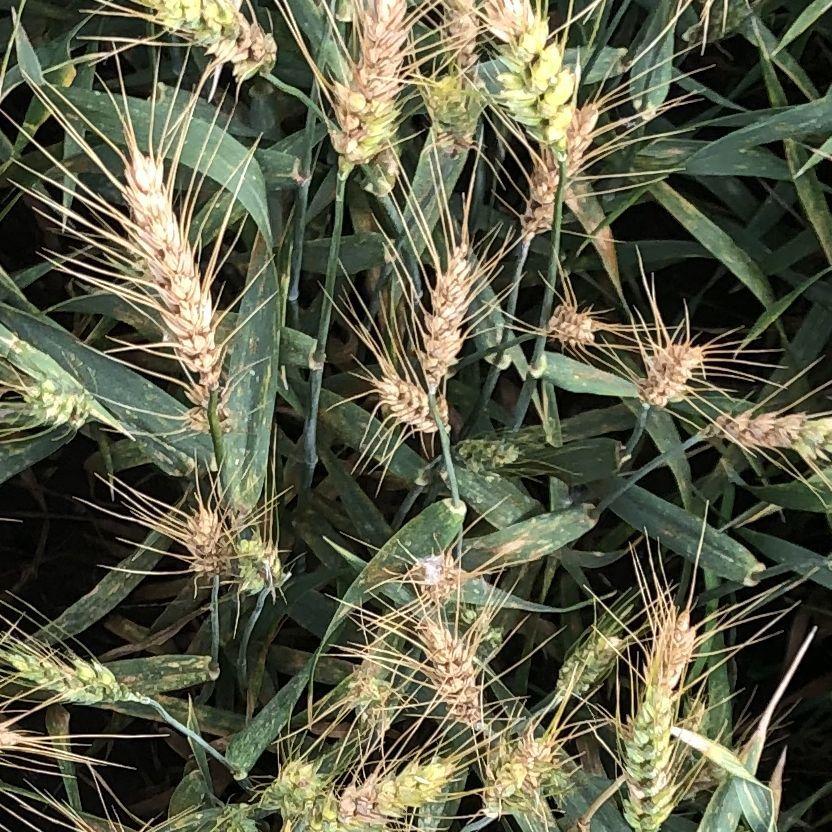

Supplement: Supplemental Information 3 [file peerj-cs-10-1948-s003.zip › data2/image0193.jpg]

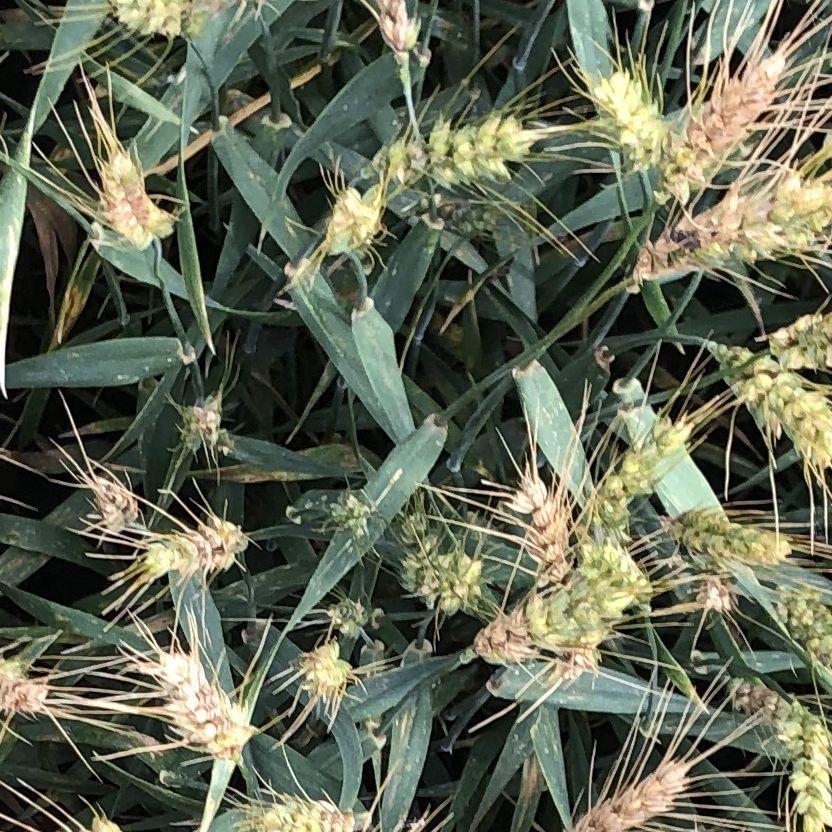

Supplement: Supplemental Information 3 [file peerj-cs-10-1948-s003.zip › data2/image0194.jpg]

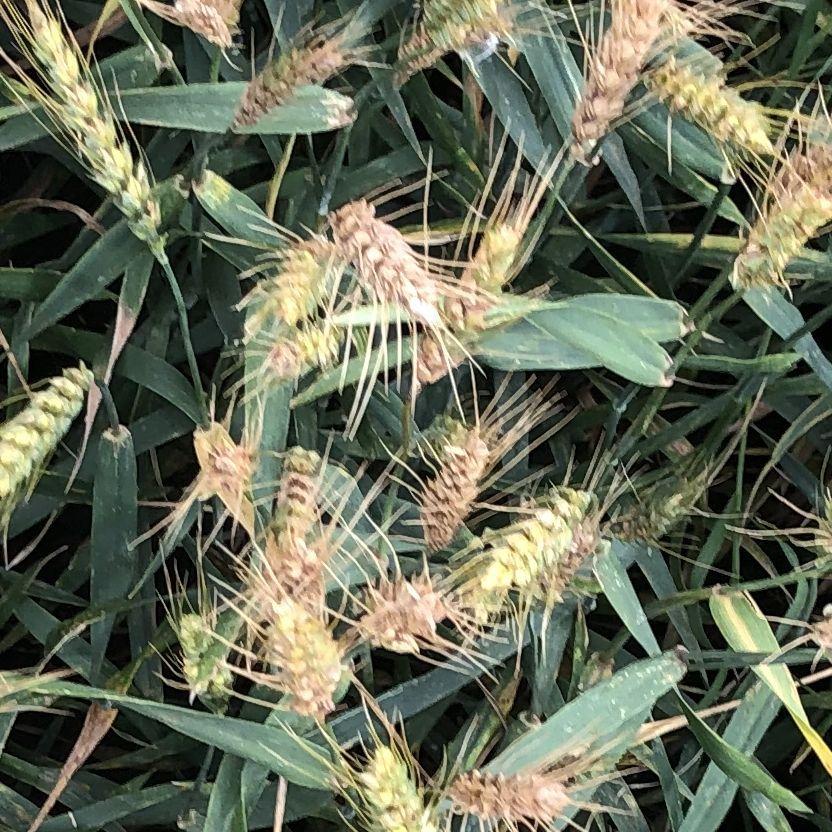

Supplement: Supplemental Information 3 [file peerj-cs-10-1948-s003.zip › data2/image0195.jpg]

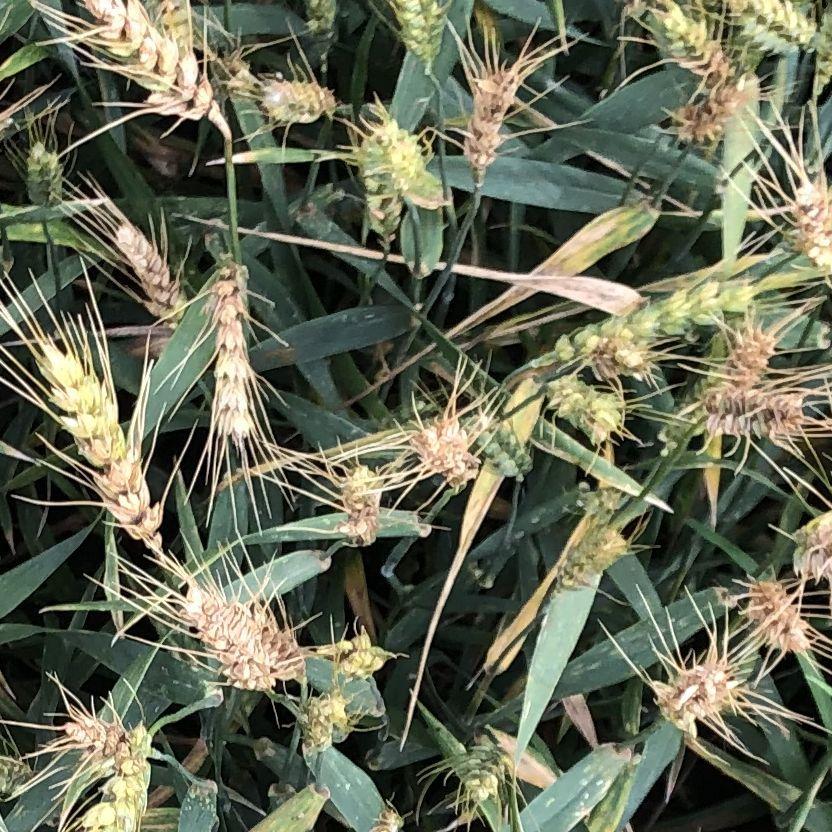

Supplement: Supplemental Information 3 [file peerj-cs-10-1948-s003.zip › data2/image0196.jpg]

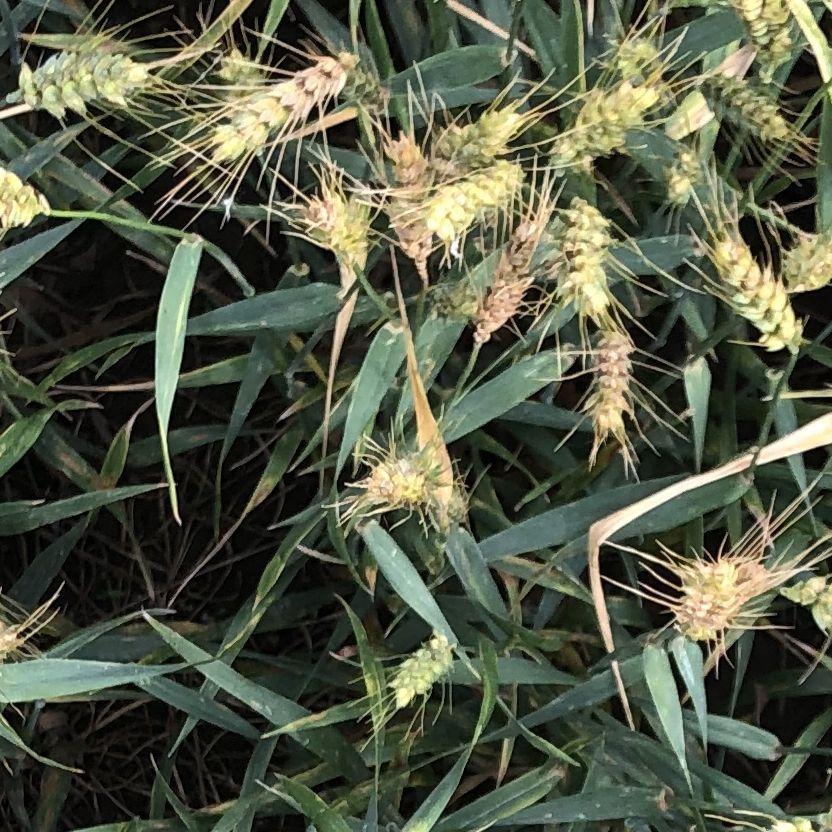

Supplement: Supplemental Information 3 [file peerj-cs-10-1948-s003.zip › data2/image0200.jpg]

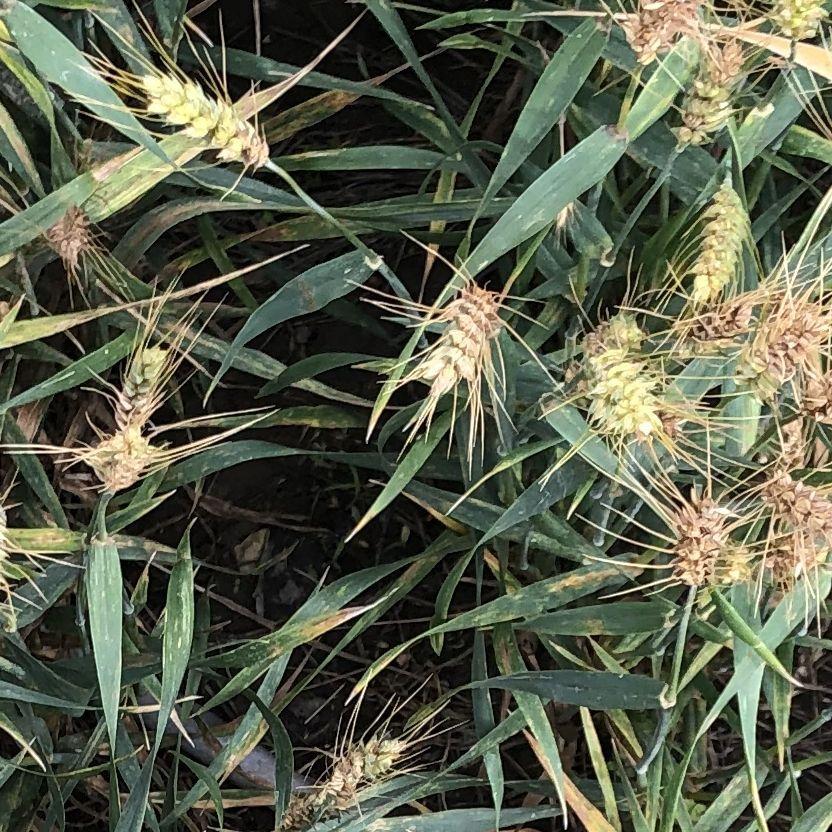

Supplement: Supplemental Information 3 [file peerj-cs-10-1948-s003.zip › data2/image0201.jpg]

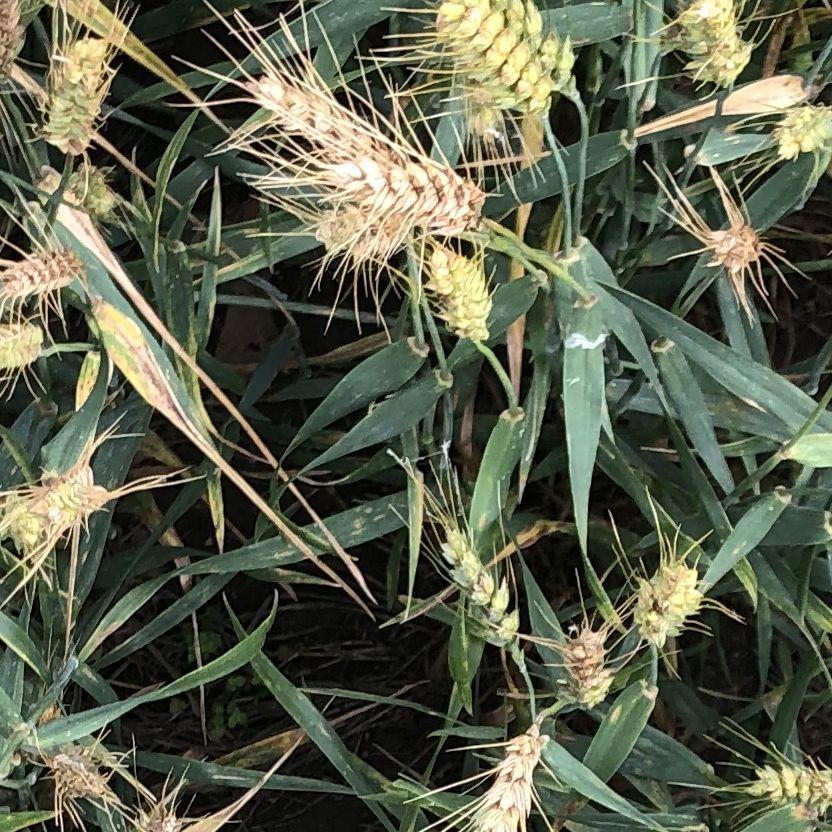

Supplement: Supplemental Information 3 [file peerj-cs-10-1948-s003.zip › data2/image0202.jpg]

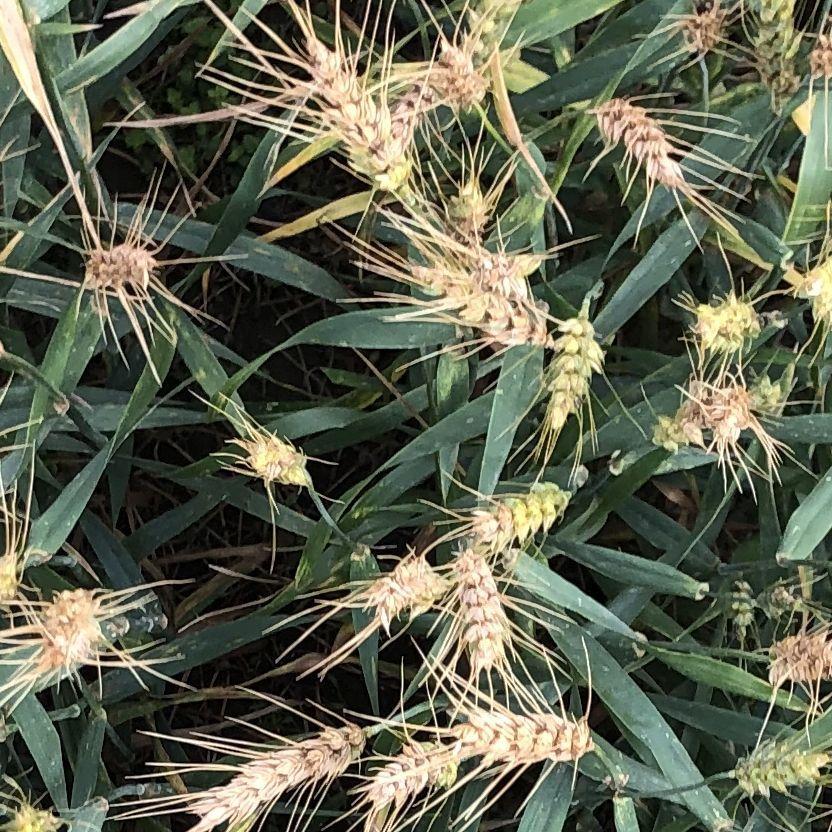

Supplement: Supplemental Information 3 [file peerj-cs-10-1948-s003.zip › data2/image0203.jpg]

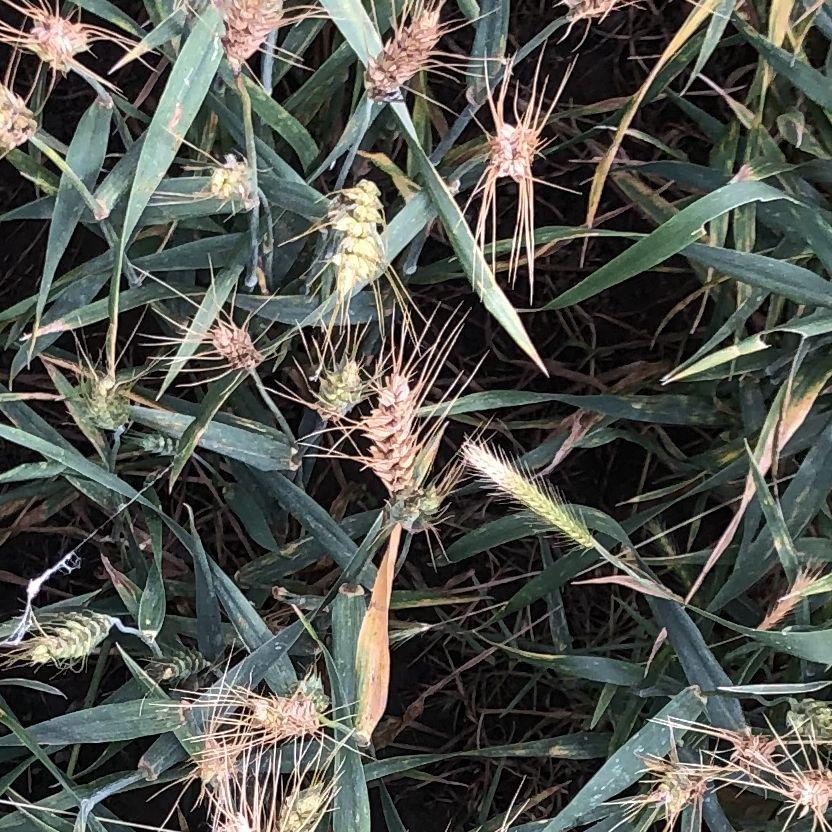

Supplement: Supplemental Information 3 [file peerj-cs-10-1948-s003.zip › data2/image0204.jpg]

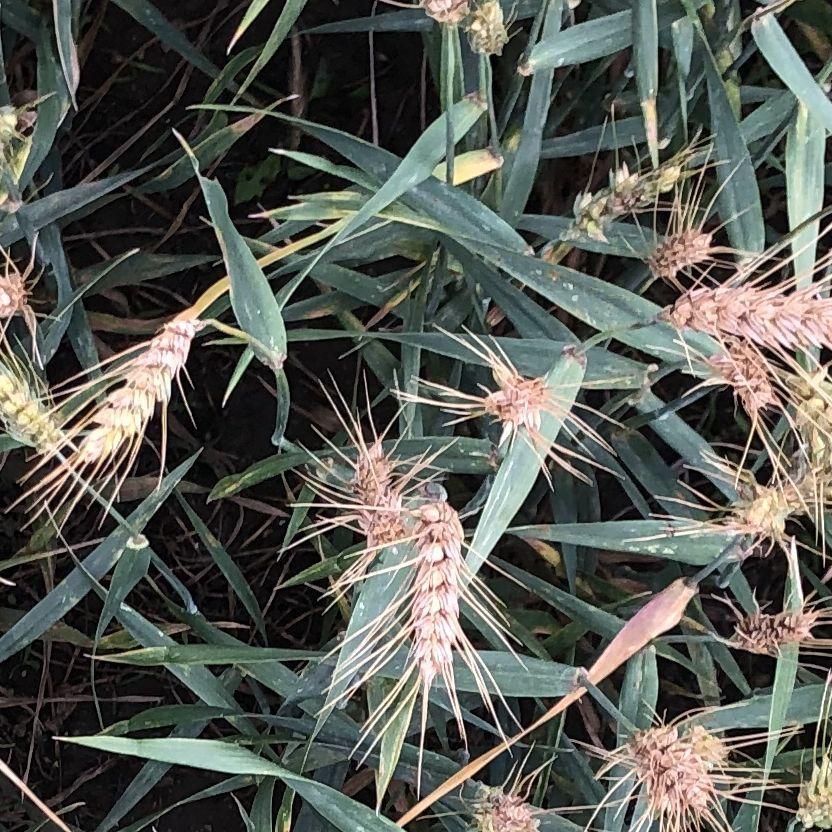

Supplement: Supplemental Information 3 [file peerj-cs-10-1948-s003.zip › data2/image0205.jpg]

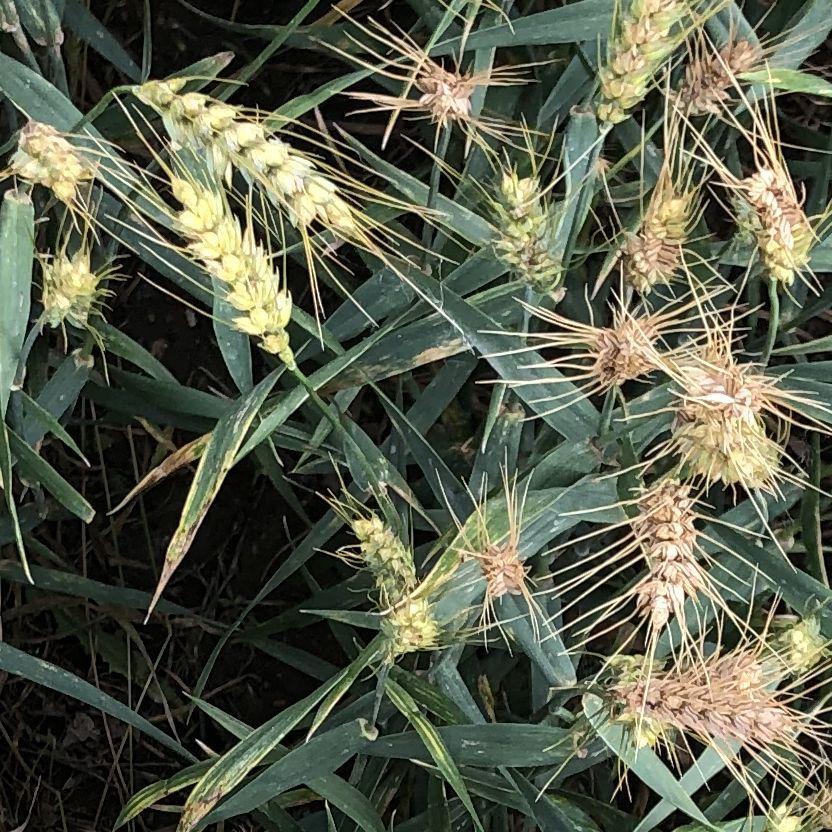

Supplement: Supplemental Information 3 [file peerj-cs-10-1948-s003.zip › data2/image0209.jpg]

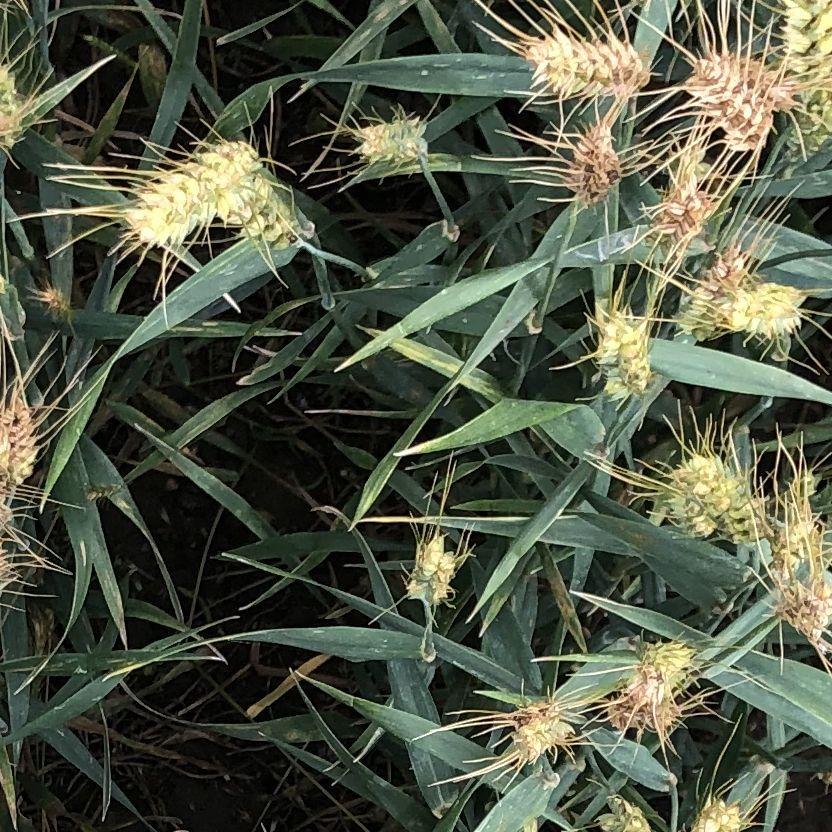

Supplement: Supplemental Information 3 [file peerj-cs-10-1948-s003.zip › data2/image0210.jpg]

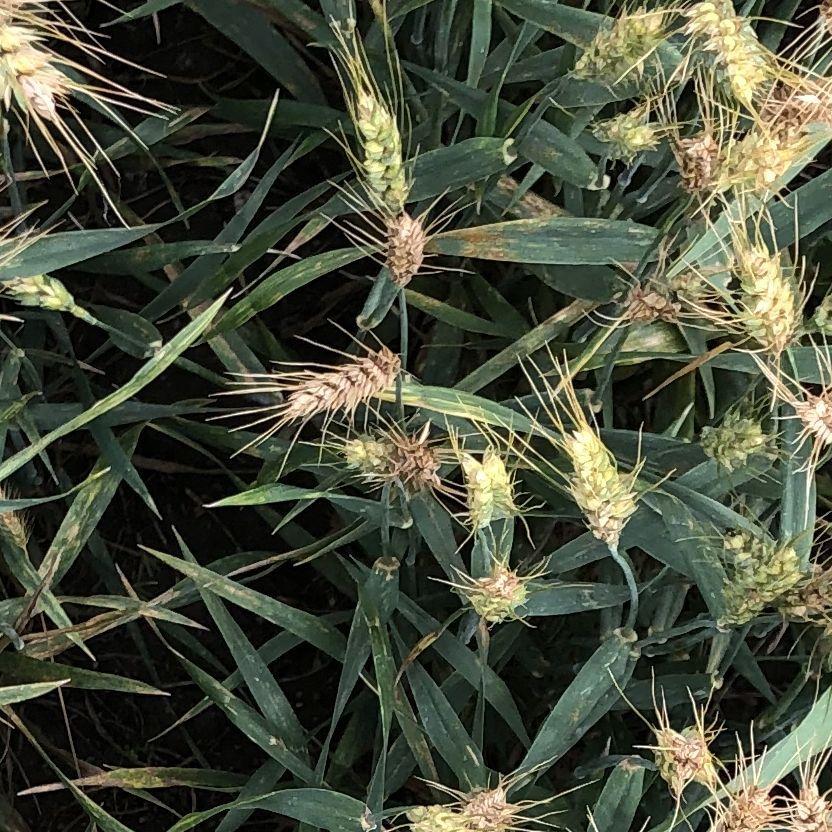

Supplement: Supplemental Information 3 [file peerj-cs-10-1948-s003.zip › data2/image0211.jpg]

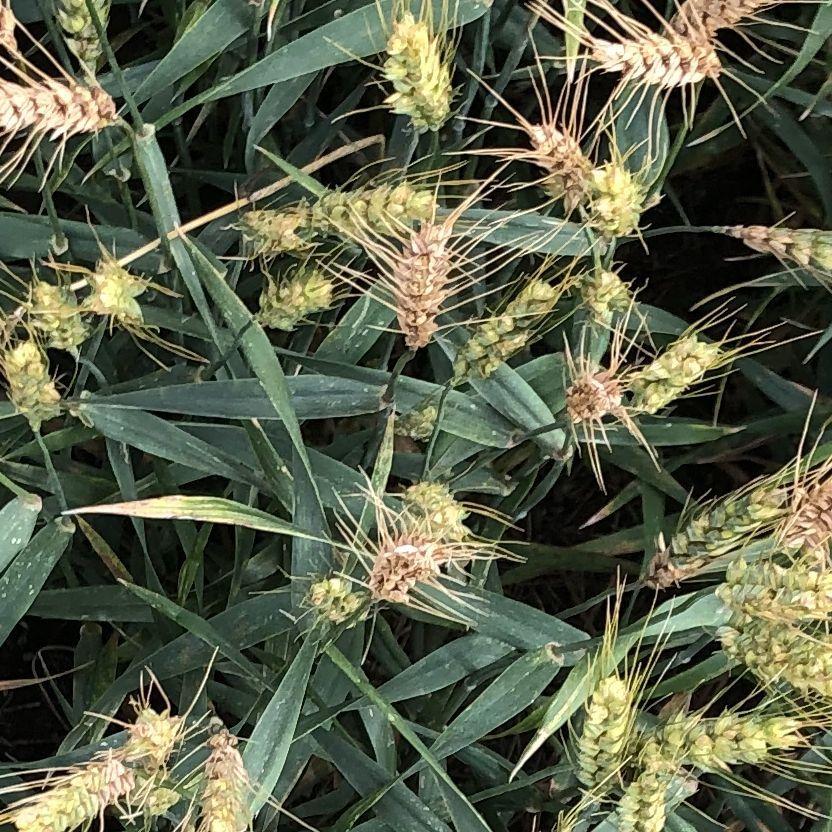

Supplement: Supplemental Information 3 [file peerj-cs-10-1948-s003.zip › data2/image0212.jpg]

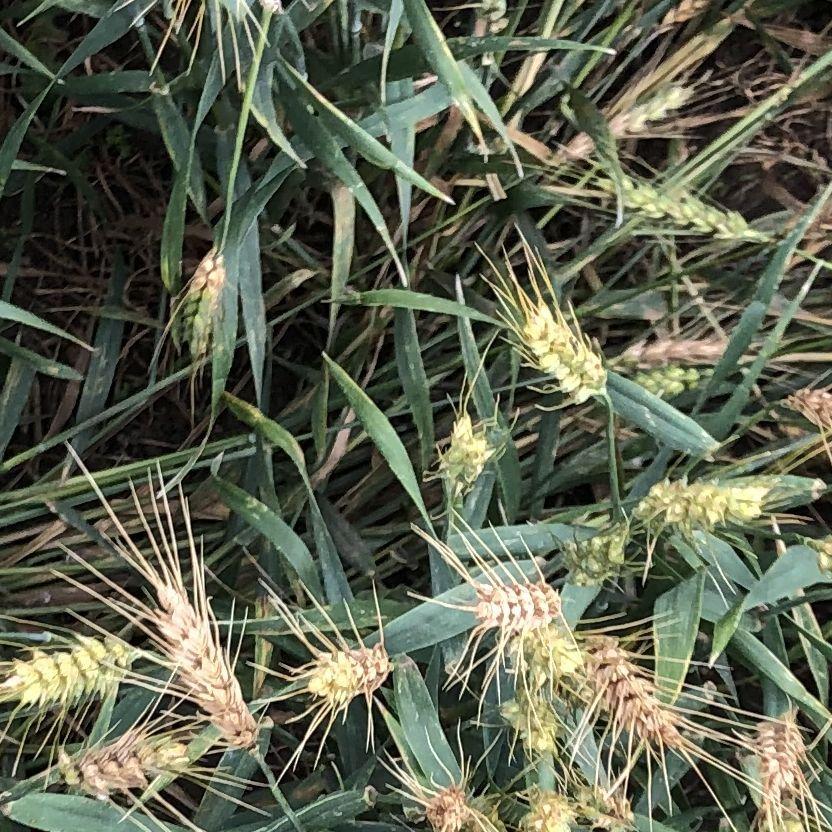

Supplement: Supplemental Information 3 [file peerj-cs-10-1948-s003.zip › data2/image0213.jpg]

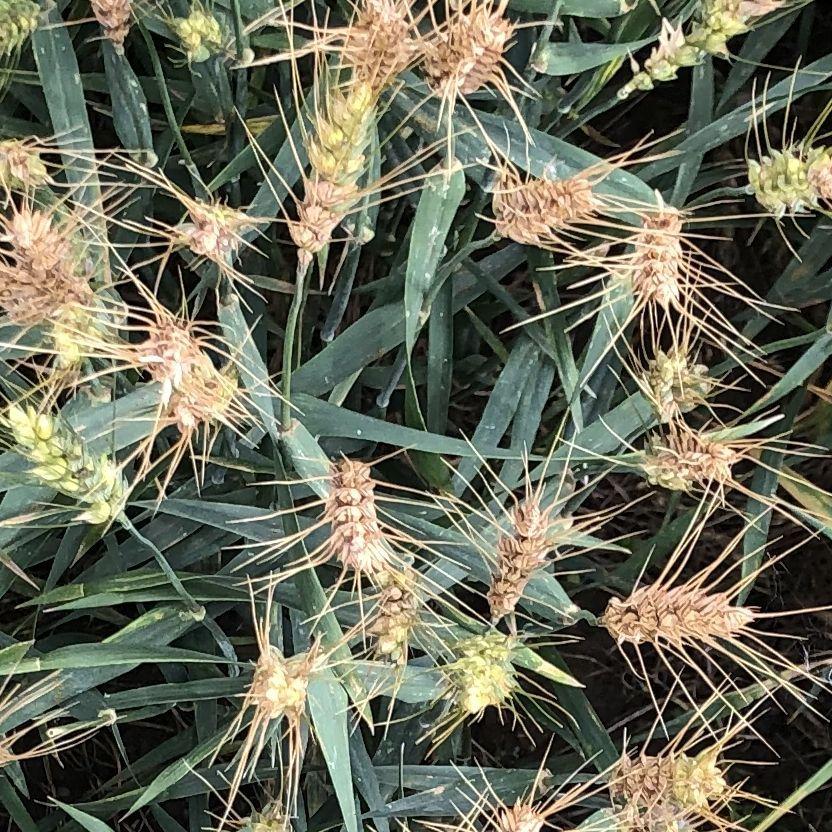

Supplement: Supplemental Information 3 [file peerj-cs-10-1948-s003.zip › data2/image0214.jpg]

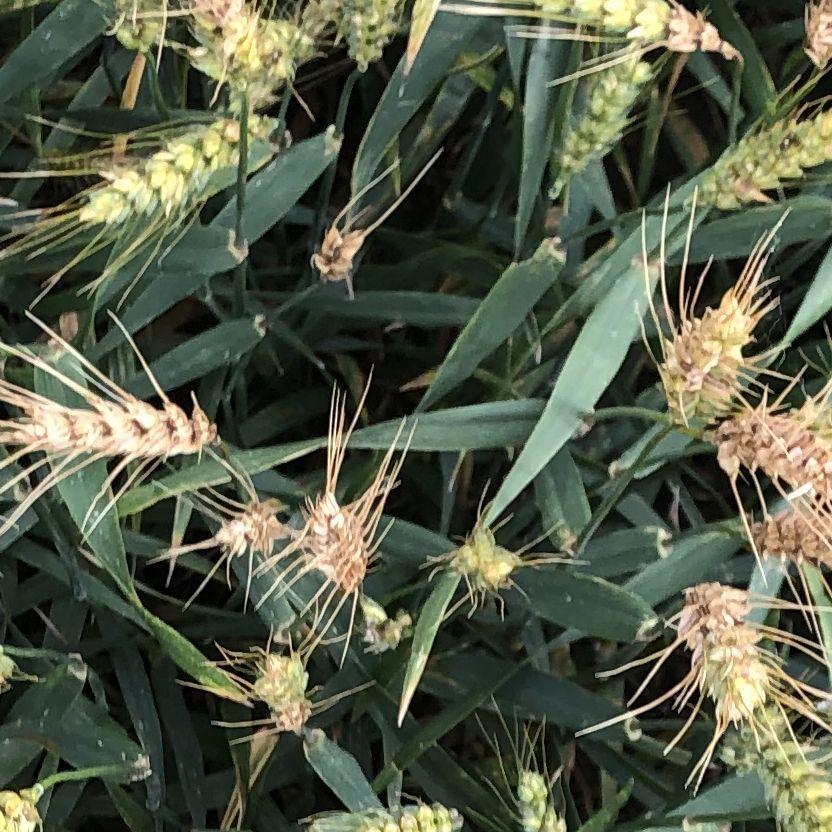

Supplement: Supplemental Information 3 [file peerj-cs-10-1948-s003.zip › data2/image0215.jpg]

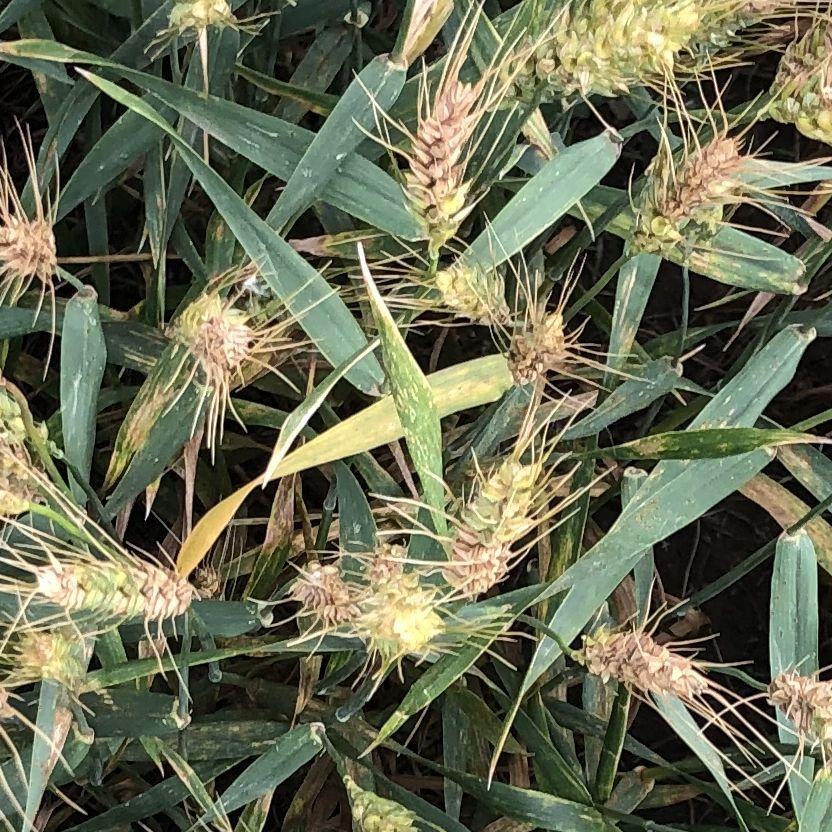

Supplement: Supplemental Information 3 [file peerj-cs-10-1948-s003.zip › data2/image0216.jpg]

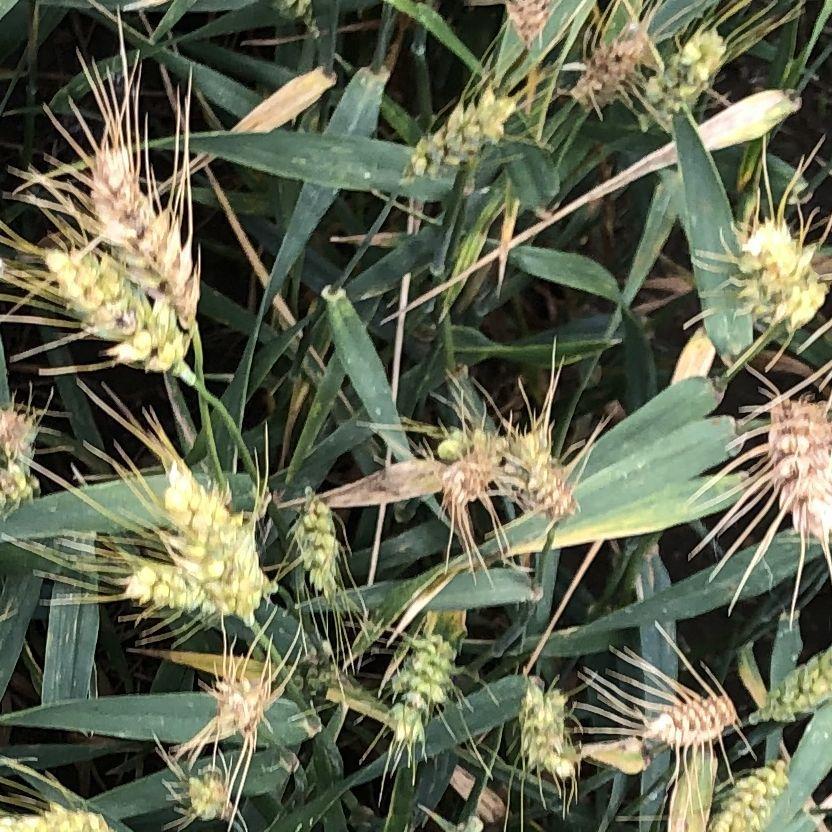

Supplement: Supplemental Information 3 [file peerj-cs-10-1948-s003.zip › data2/image0217.jpg]

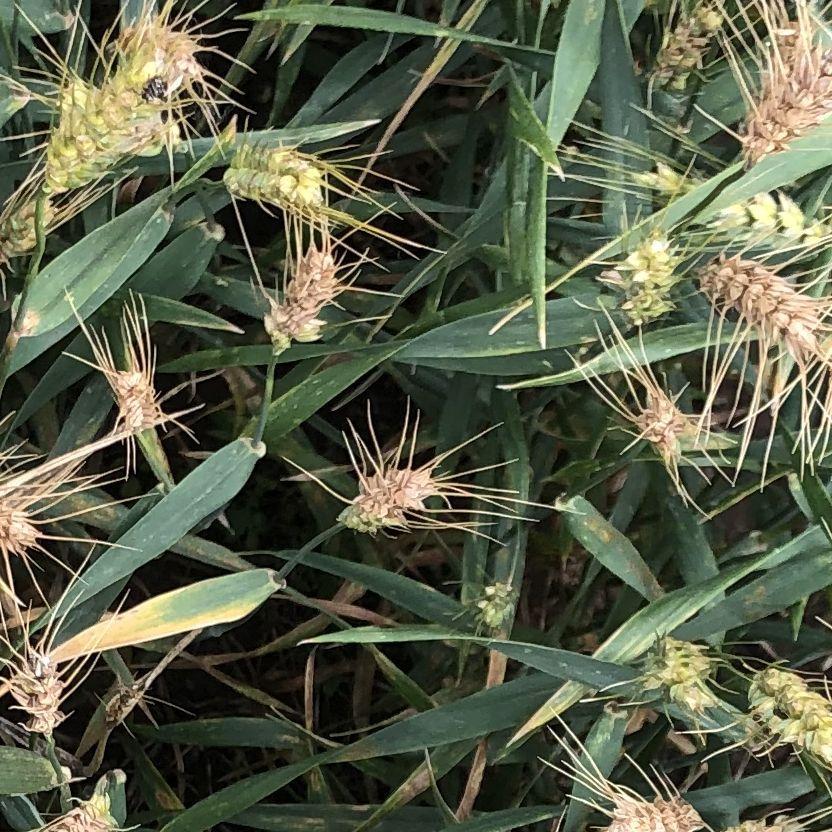

Supplement: Supplemental Information 3 [file peerj-cs-10-1948-s003.zip › data2/image0218.jpg]

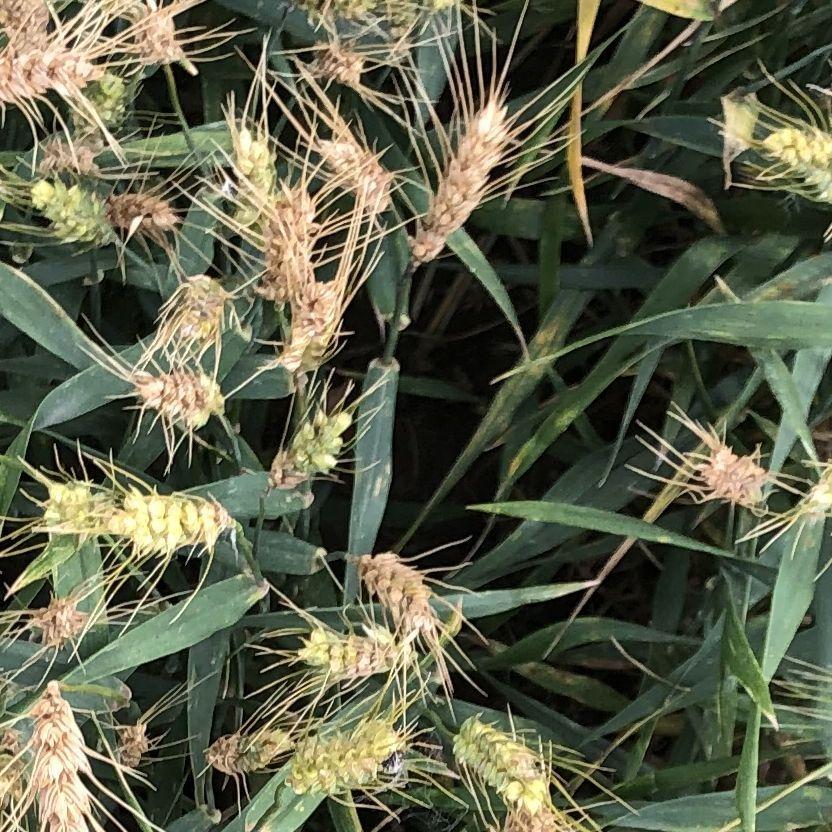

Supplement: Supplemental Information 3 [file peerj-cs-10-1948-s003.zip › data2/image0219.jpg]

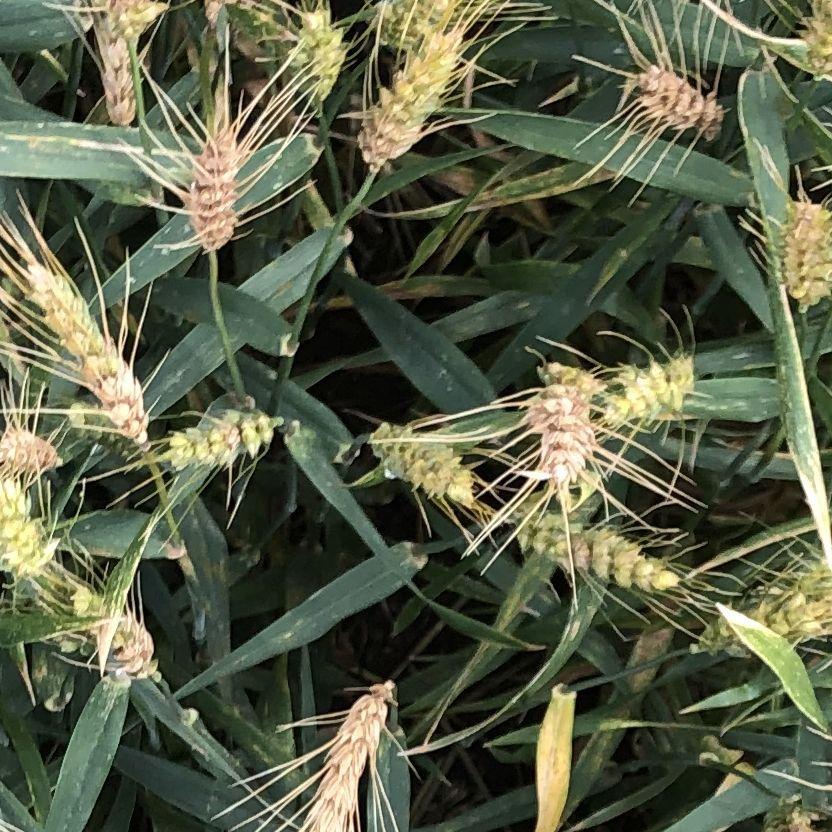

Supplement: Supplemental Information 3 [file peerj-cs-10-1948-s003.zip › data2/image0220.jpg]

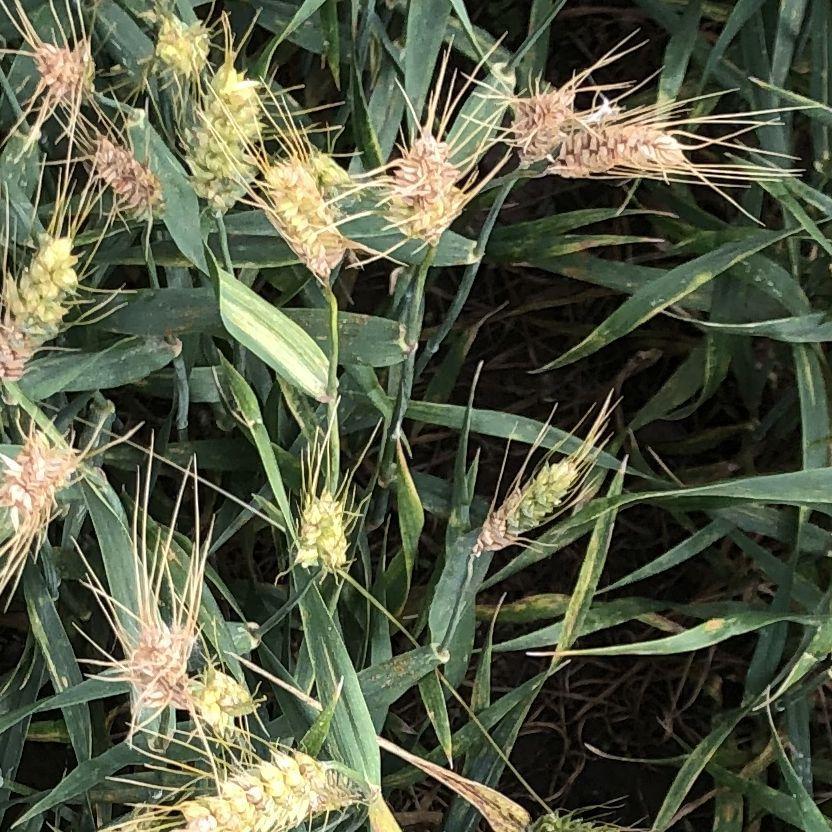

Supplement: Supplemental Information 3 [file peerj-cs-10-1948-s003.zip › data2/image0222.jpg]

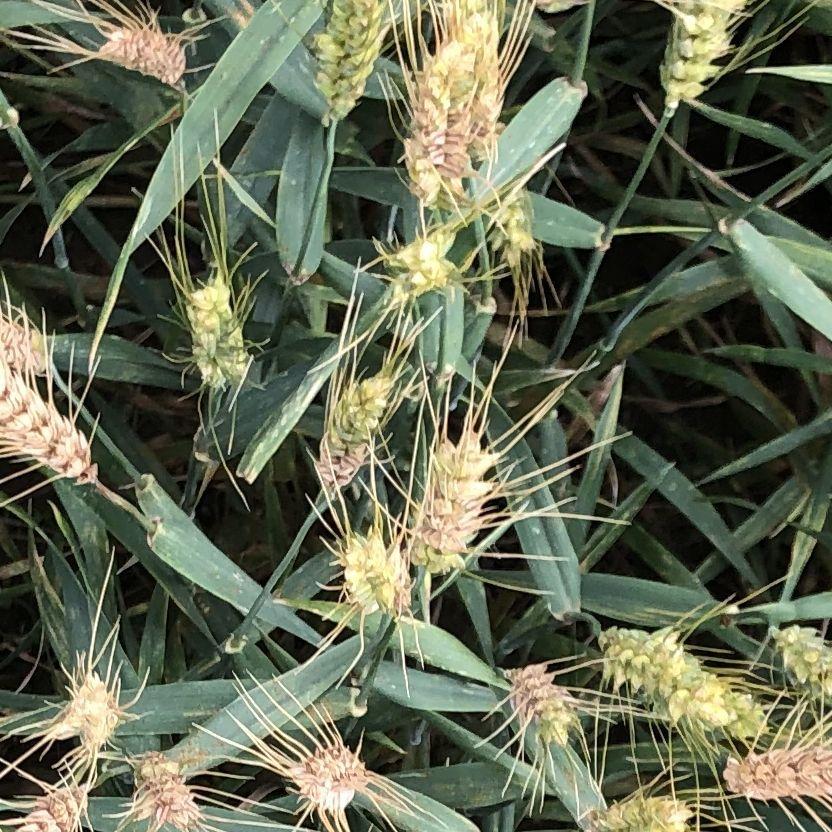

Supplement: Supplemental Information 3 [file peerj-cs-10-1948-s003.zip › data2/image0223.jpg]

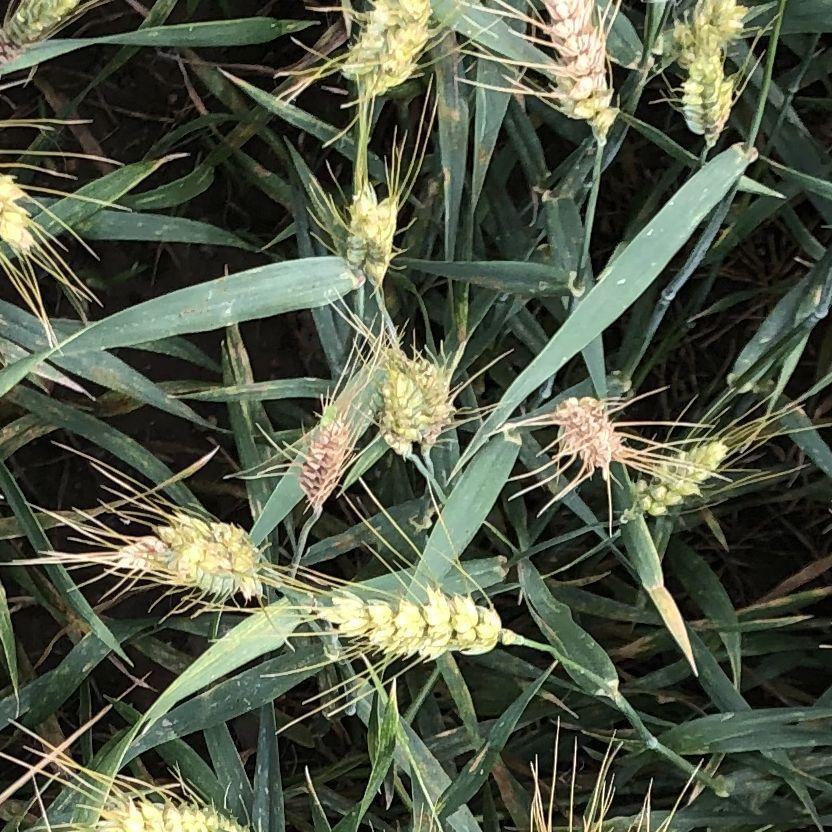

Supplement: Supplemental Information 3 [file peerj-cs-10-1948-s003.zip › data2/image0224.jpg]

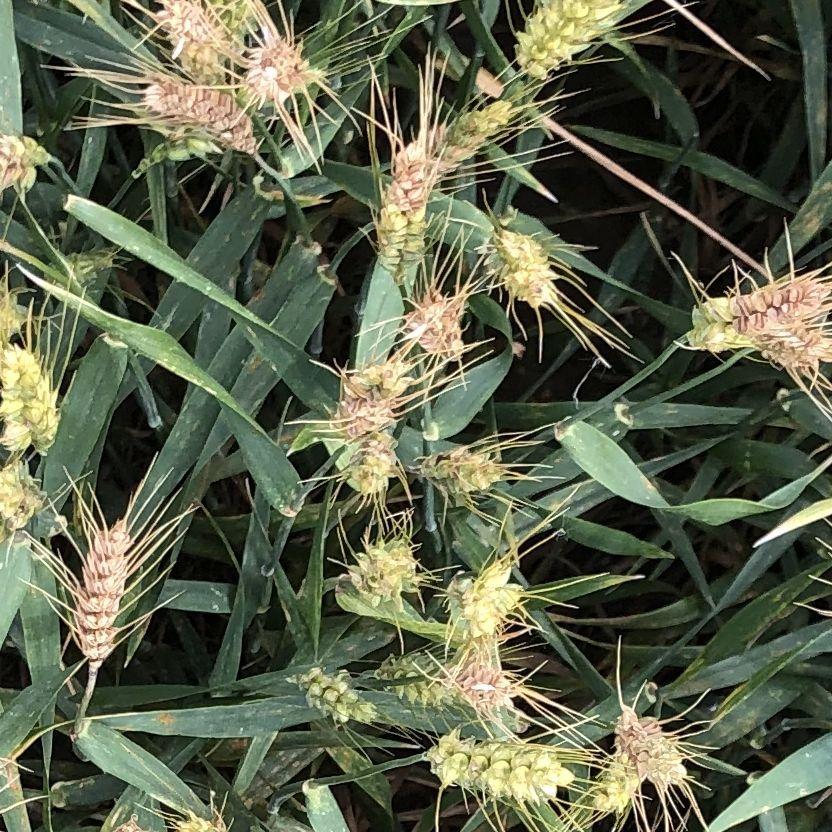

Supplement: Supplemental Information 3 [file peerj-cs-10-1948-s003.zip › data2/image0225.jpg]

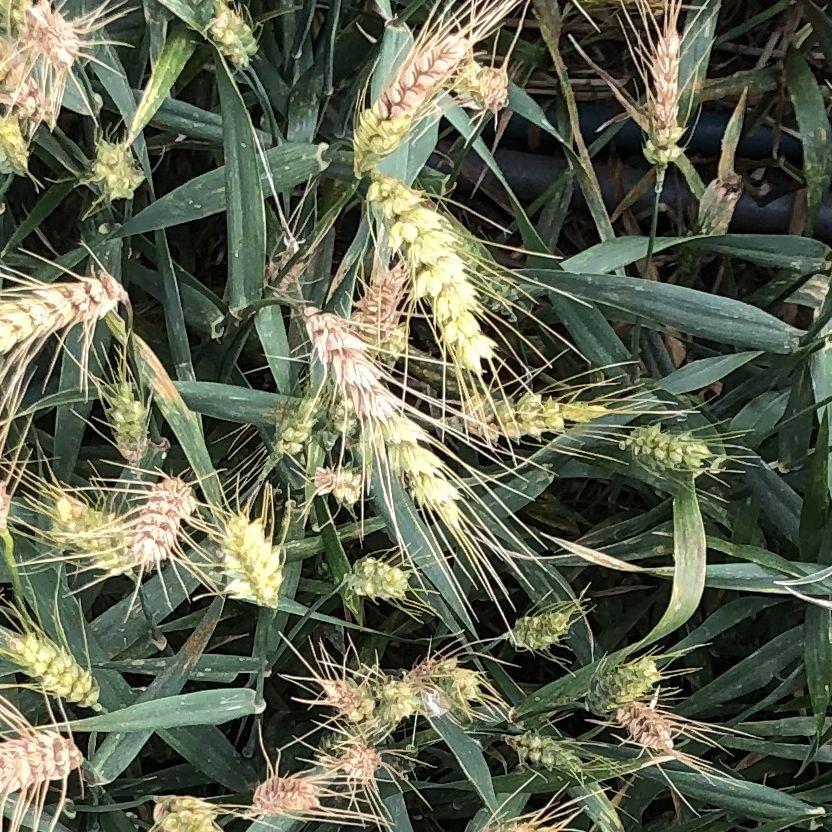

Supplement: Supplemental Information 3 [file peerj-cs-10-1948-s003.zip › data2/image0226.jpg]

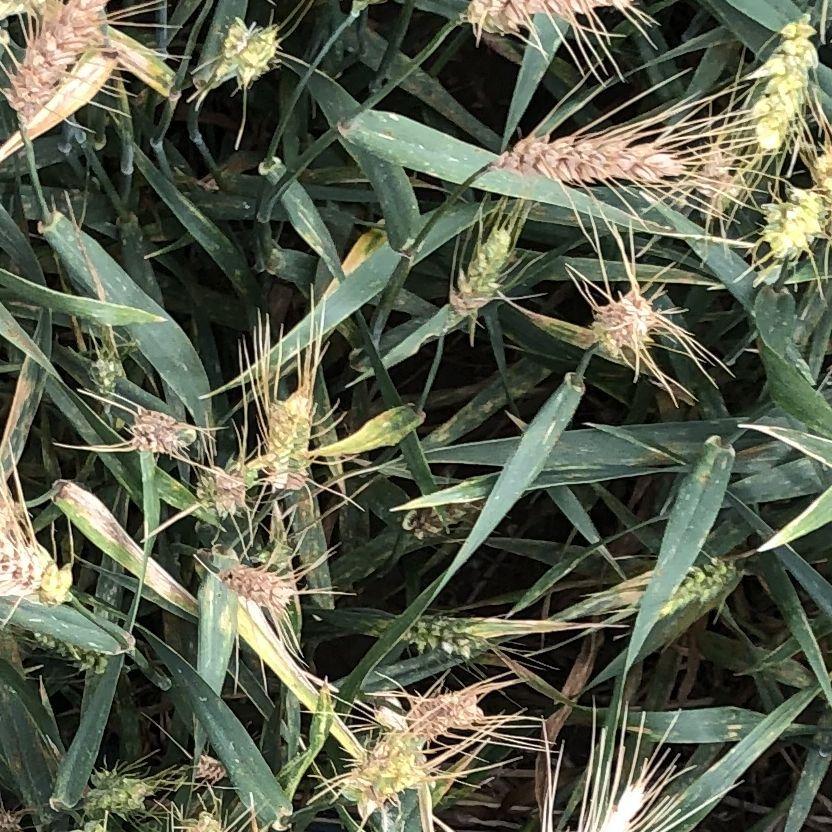

Supplement: Supplemental Information 3 [file peerj-cs-10-1948-s003.zip › data2/image0227.jpg]

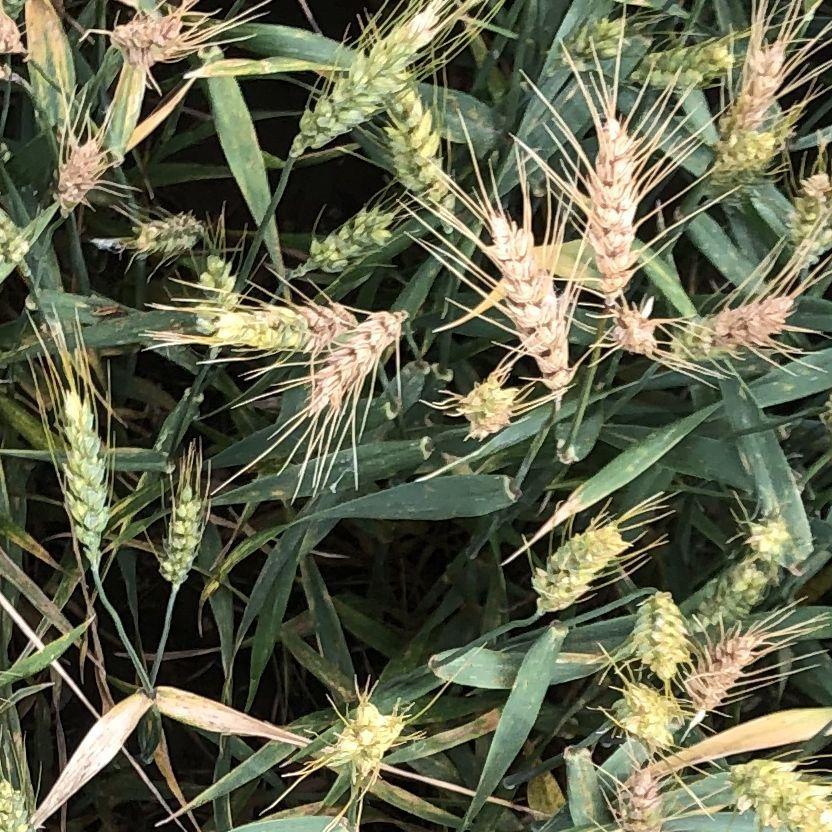

Supplement: Supplemental Information 3 [file peerj-cs-10-1948-s003.zip › data2/image0228.jpg]

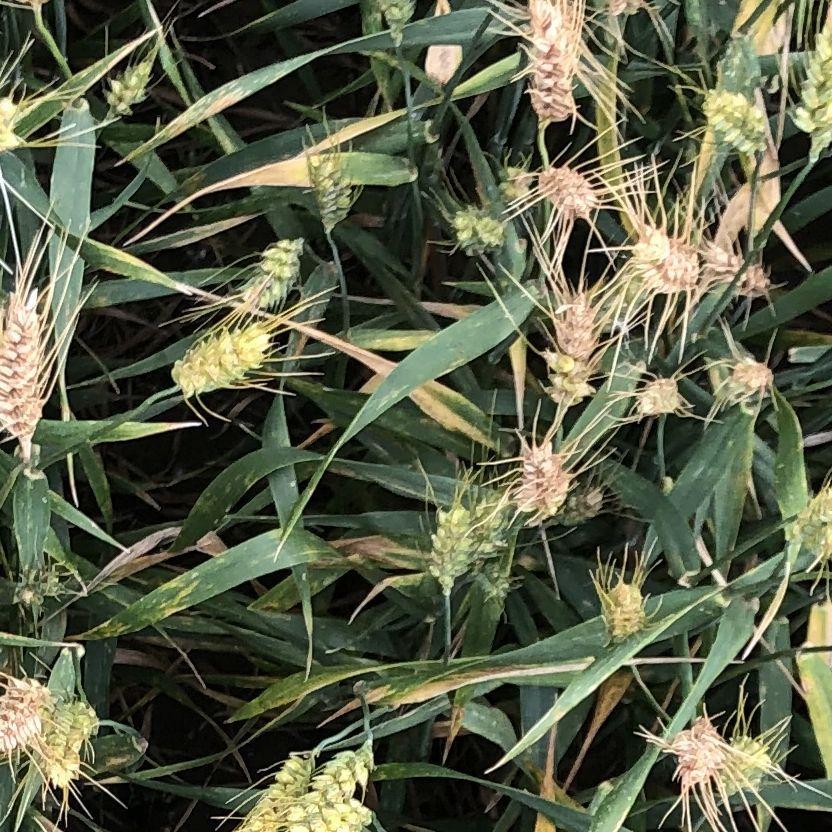

Supplement: Supplemental Information 3 [file peerj-cs-10-1948-s003.zip › data2/image0229.jpg]

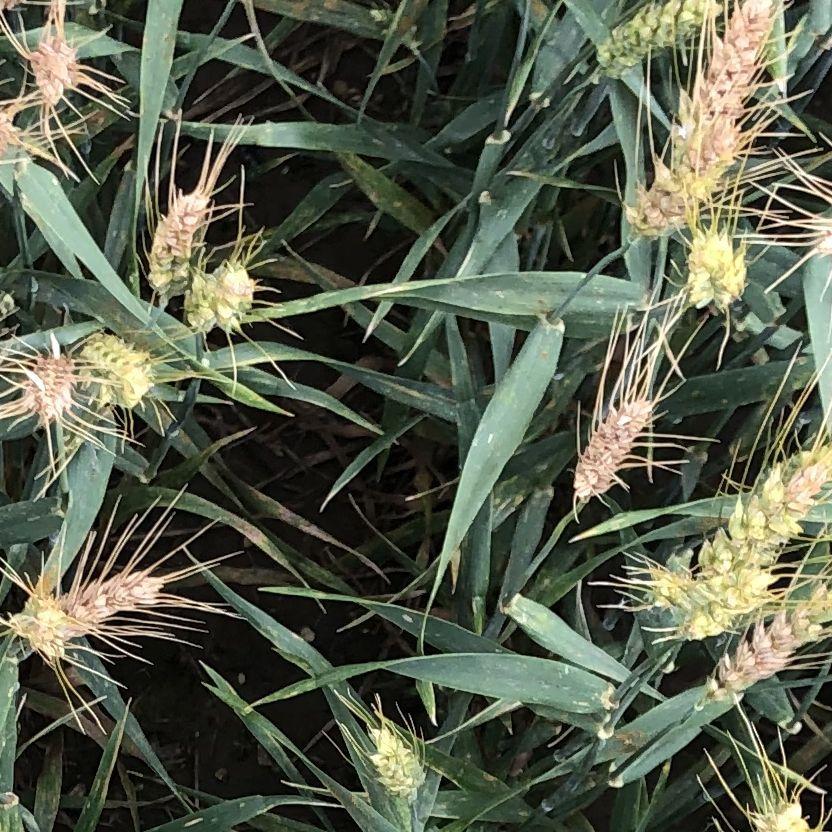

Supplement: Supplemental Information 3 [file peerj-cs-10-1948-s003.zip › data2/image0230.jpg]

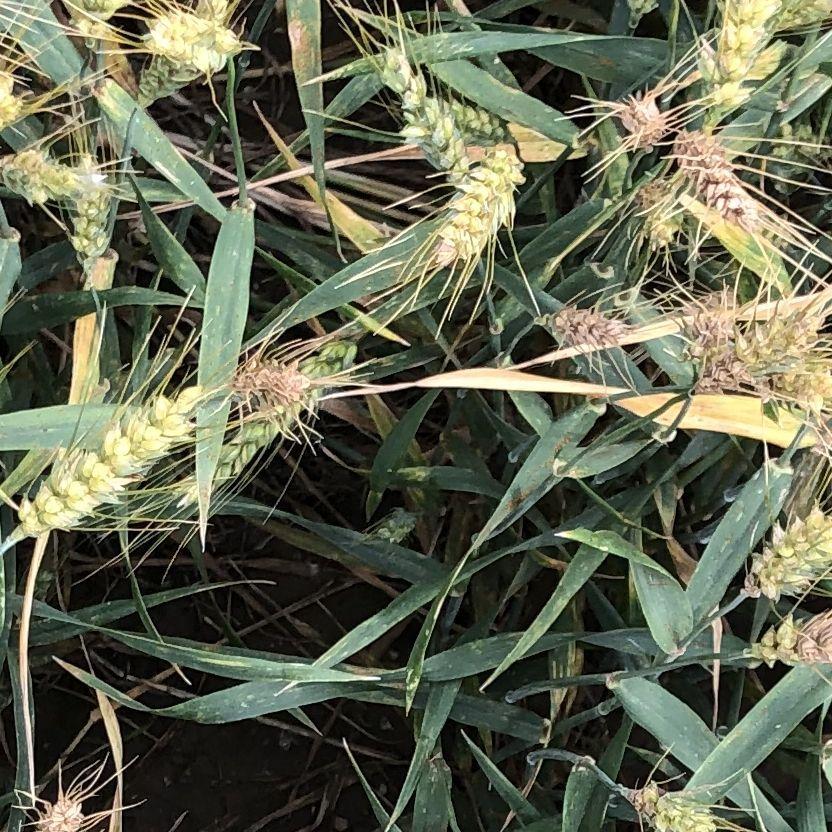

Supplement: Supplemental Information 3 [file peerj-cs-10-1948-s003.zip › data2/image0231.jpg]

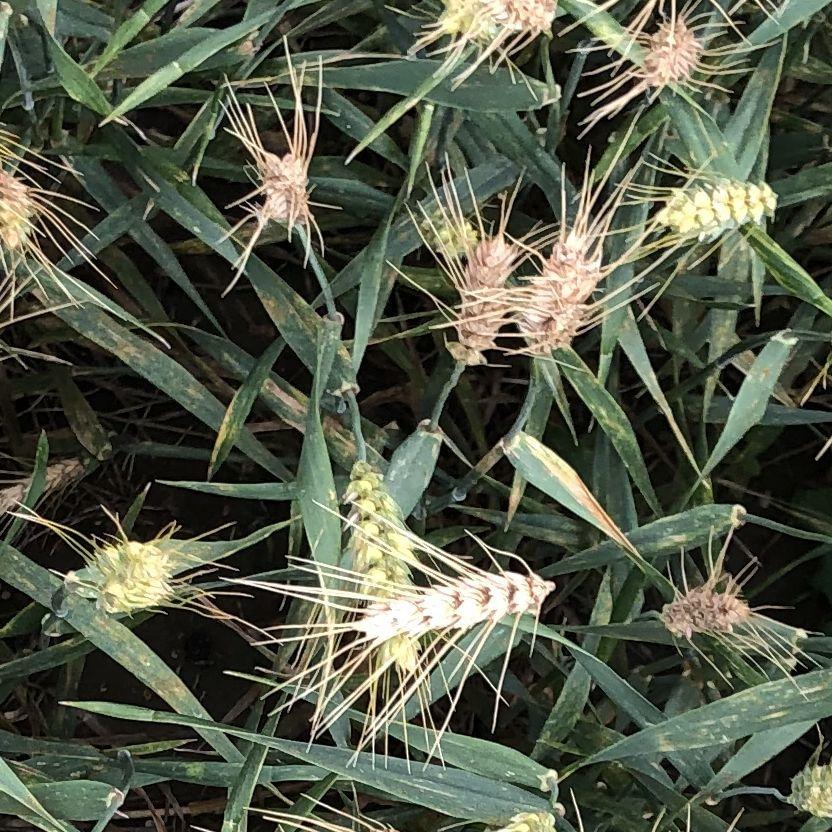

Supplement: Supplemental Information 3 [file peerj-cs-10-1948-s003.zip › data2/image0232.jpg]

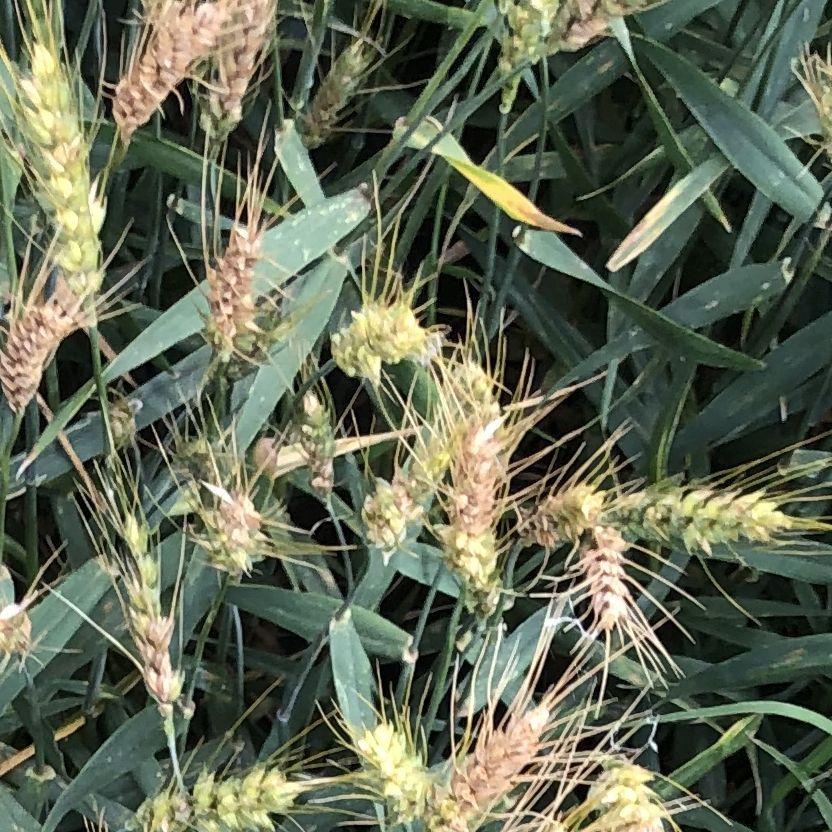

Supplement: Supplemental Information 3 [file peerj-cs-10-1948-s003.zip › data2/image0233.jpg]

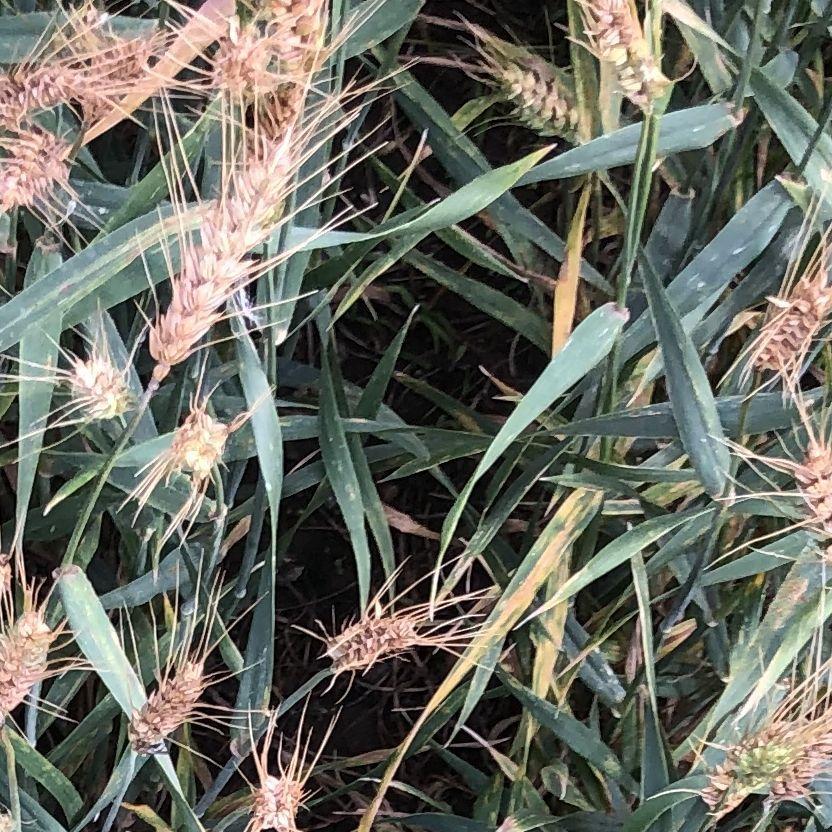

Supplement: Supplemental Information 3 [file peerj-cs-10-1948-s003.zip › data2/image0241.jpg]

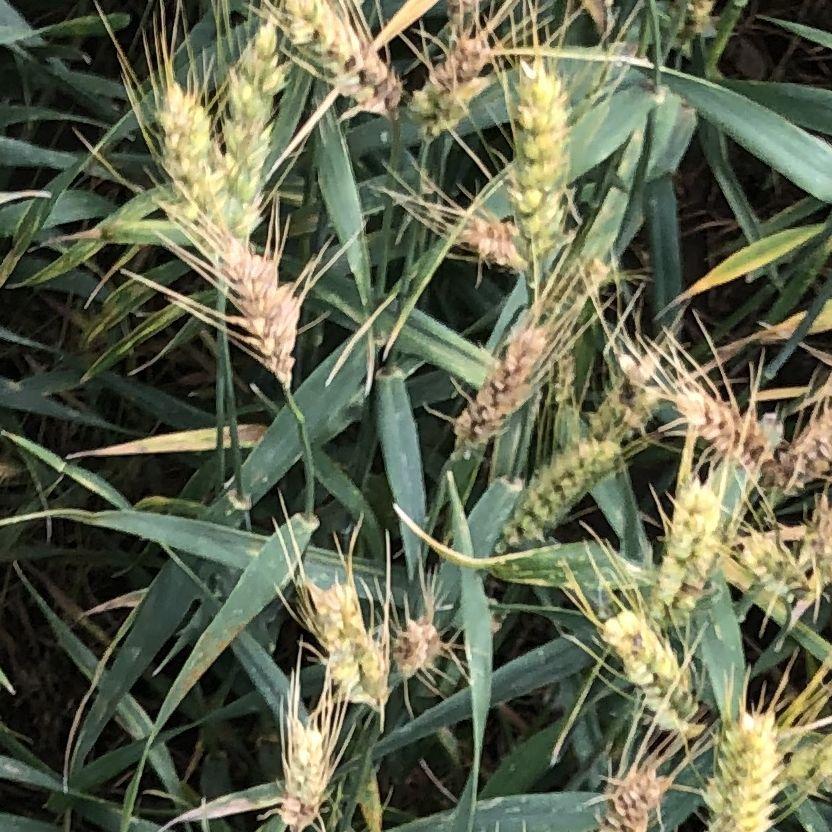

Supplement: Supplemental Information 3 [file peerj-cs-10-1948-s003.zip › data2/image0242.jpg]

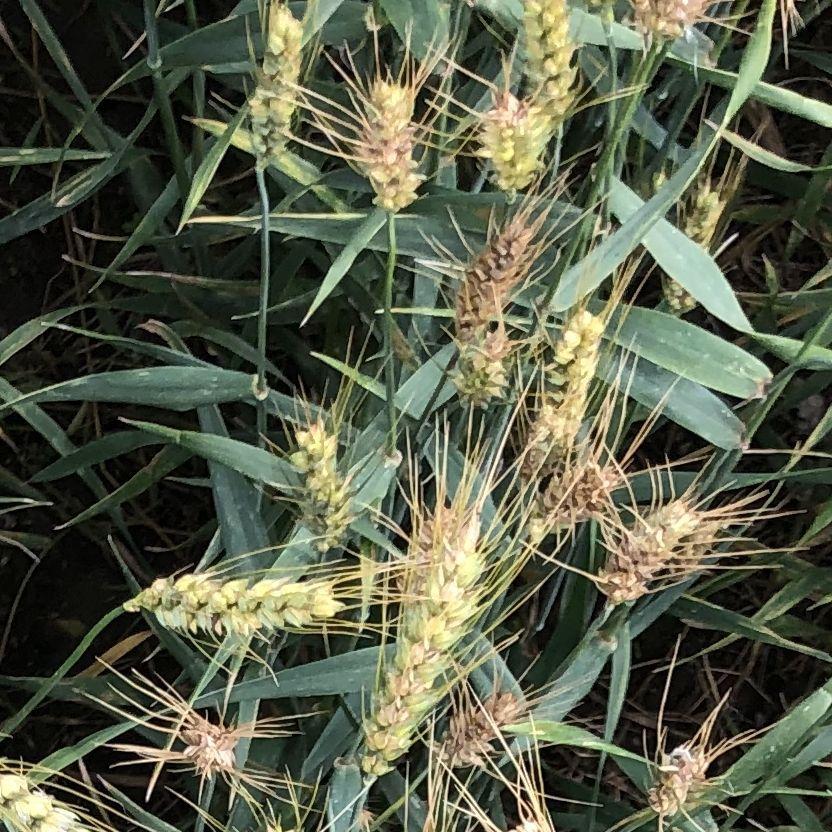

Supplement: Supplemental Information 3 [file peerj-cs-10-1948-s003.zip › data2/image0243.jpg]

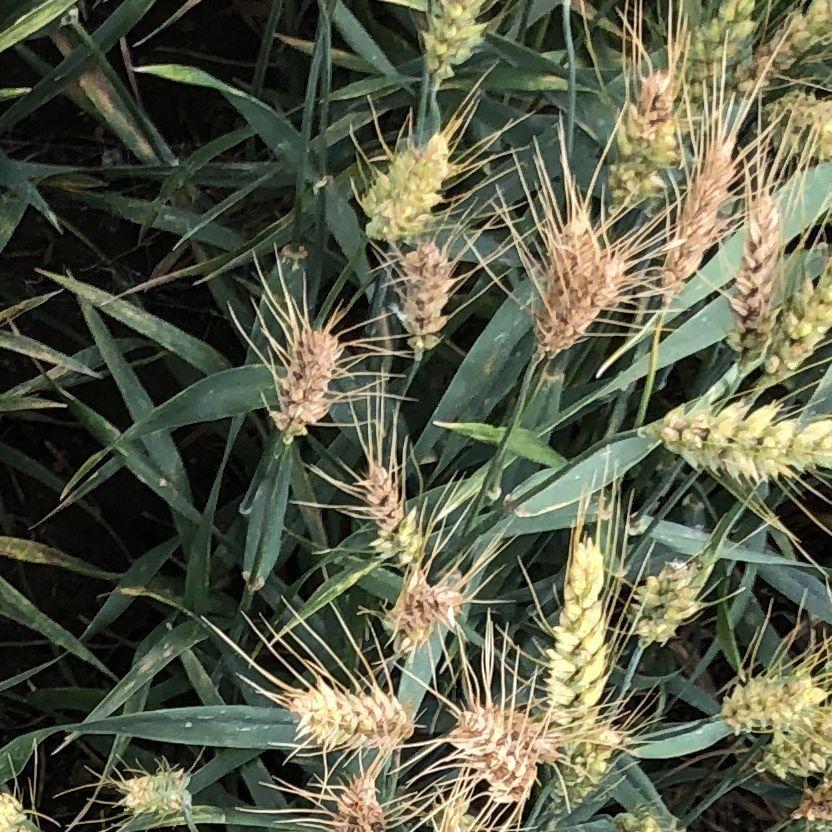

Supplement: Supplemental Information 3 [file peerj-cs-10-1948-s003.zip › data2/image0244.jpg]

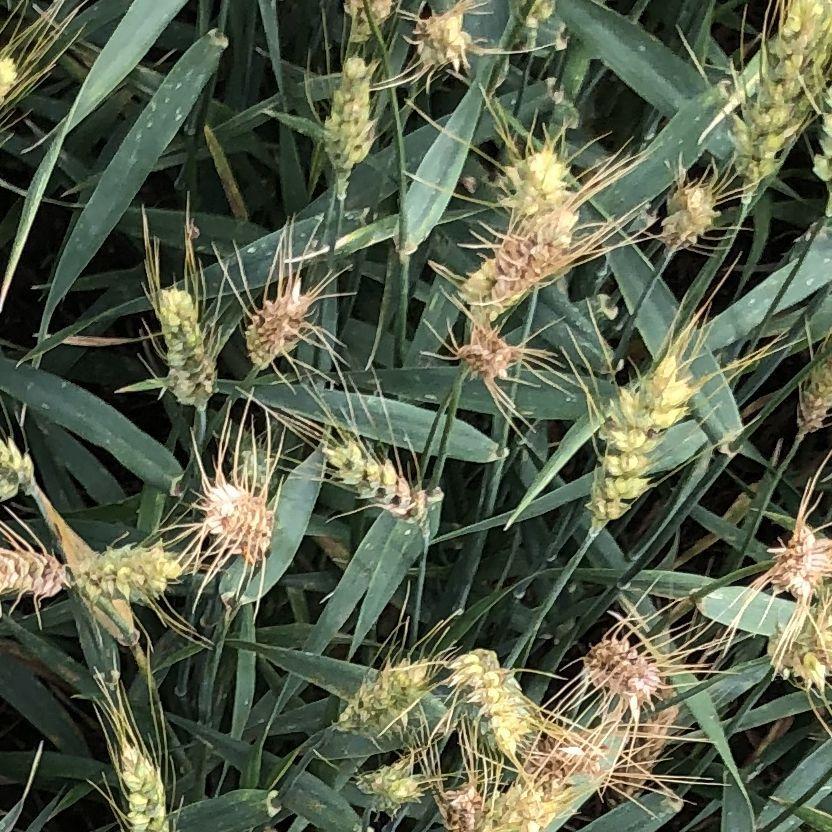

Supplement: Supplemental Information 3 [file peerj-cs-10-1948-s003.zip › data2/image0245.jpg]

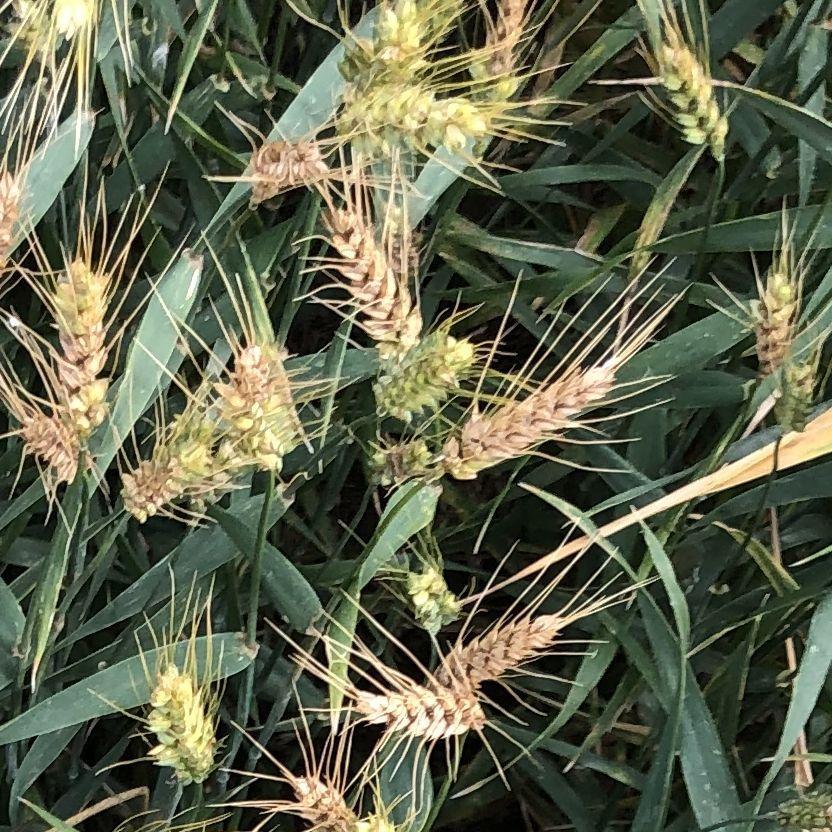

Supplement: Supplemental Information 3 [file peerj-cs-10-1948-s003.zip › data2/image0246.jpg]

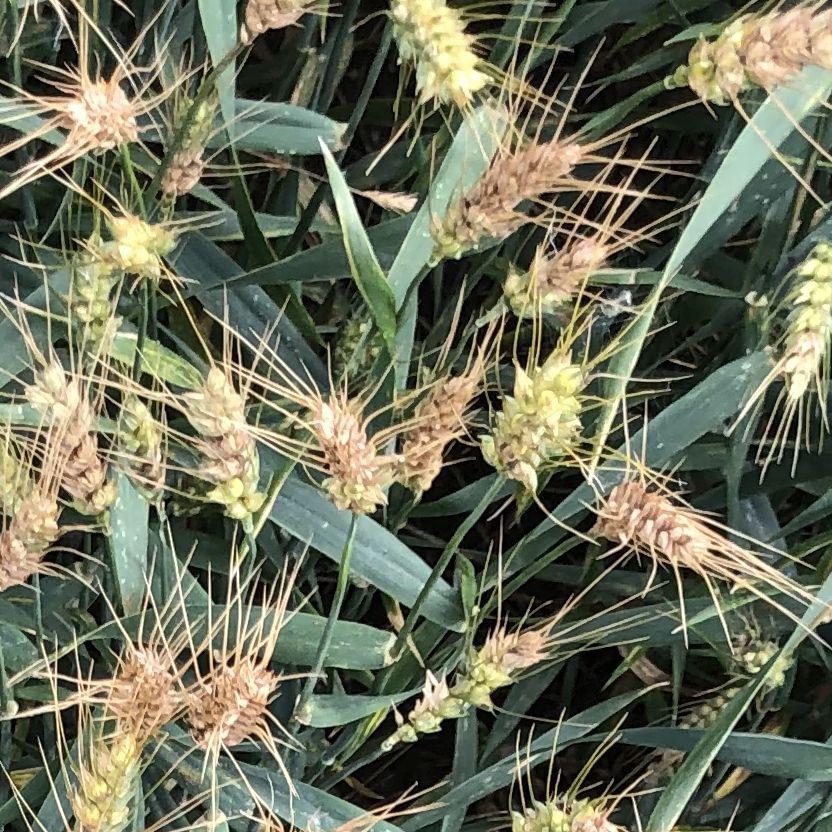

Supplement: Supplemental Information 3 [file peerj-cs-10-1948-s003.zip › data2/image0247.jpg]

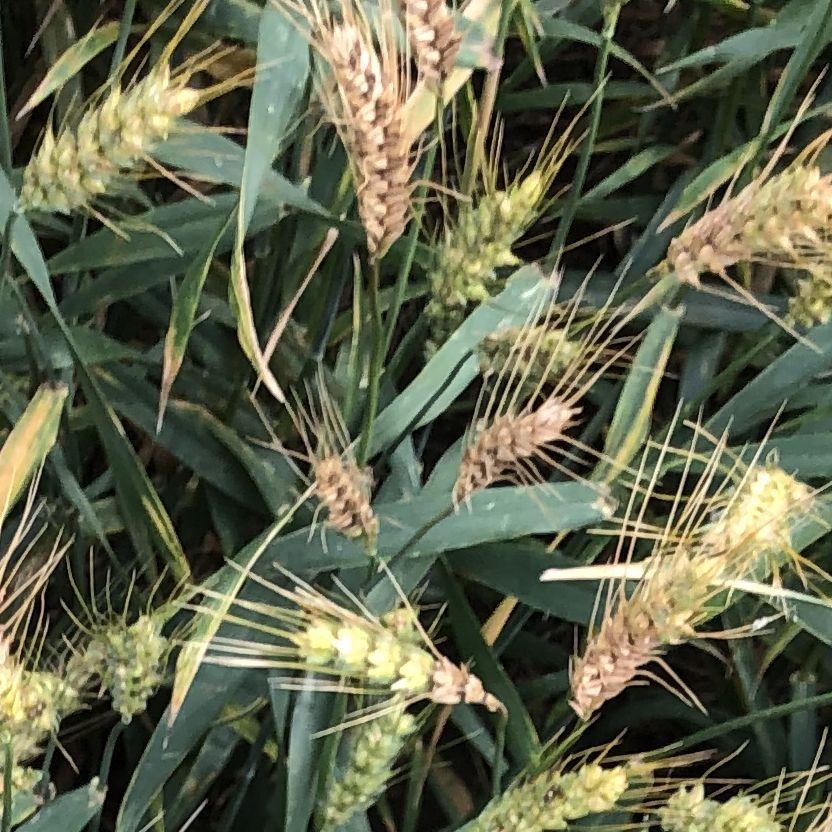

Supplement: Supplemental Information 3 [file peerj-cs-10-1948-s003.zip › data2/image0248.jpg]

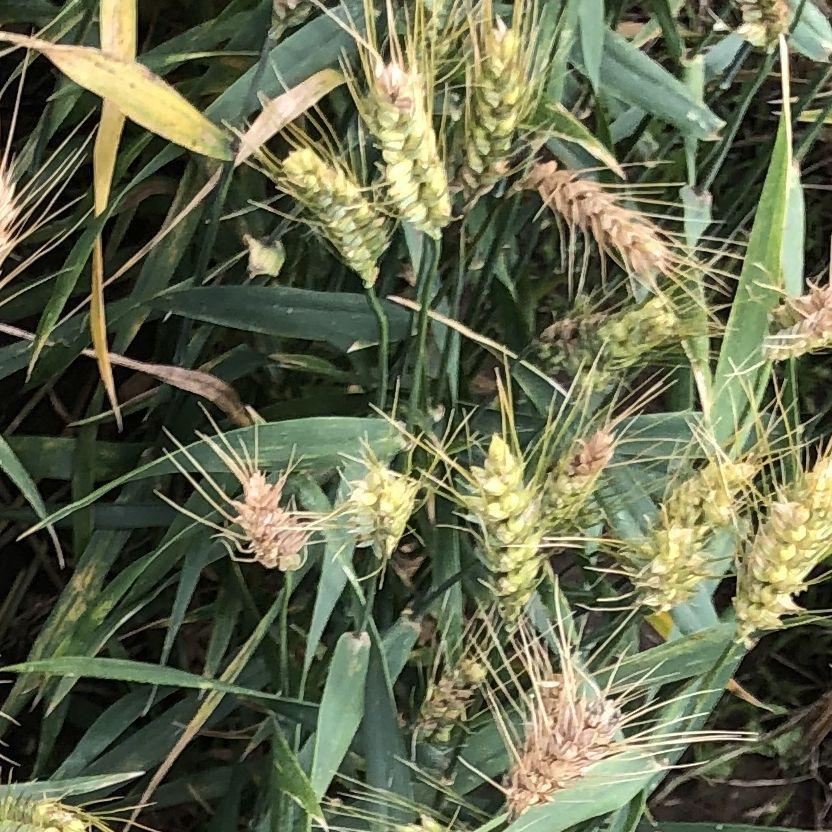

Supplement: Supplemental Information 3 [file peerj-cs-10-1948-s003.zip › data2/image0250.jpg]

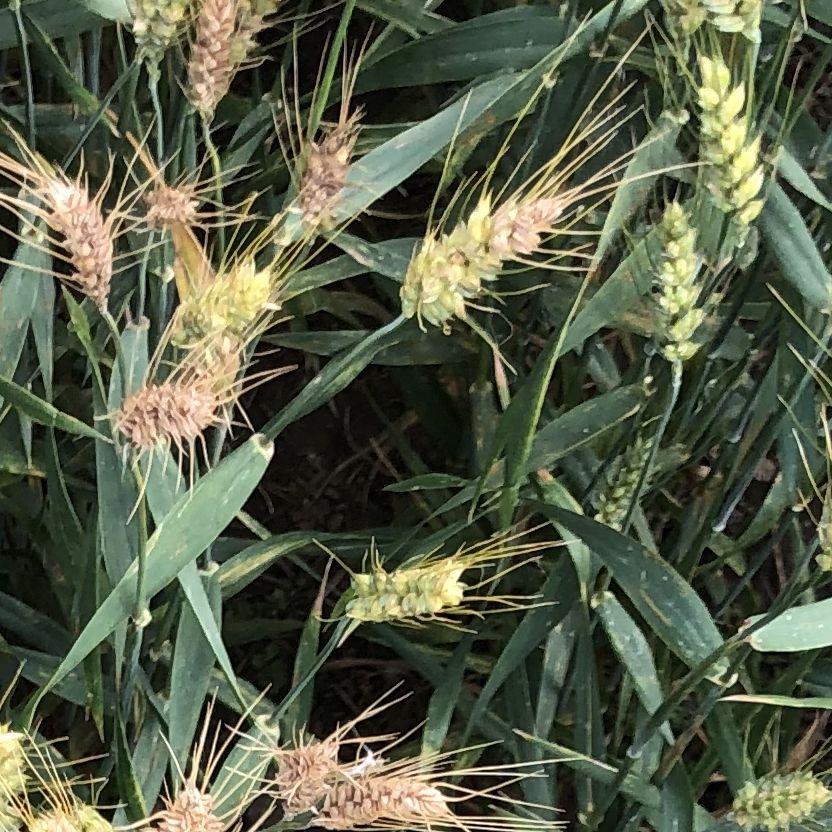

Supplement: Supplemental Information 3 [file peerj-cs-10-1948-s003.zip › data2/image0251.jpg]

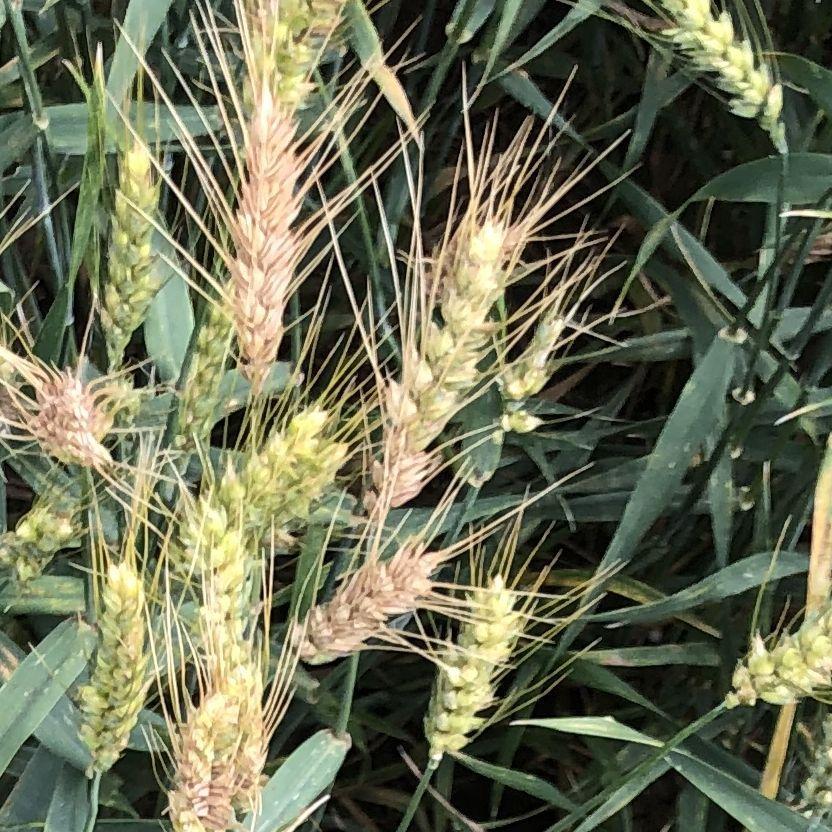

Supplement: Supplemental Information 3 [file peerj-cs-10-1948-s003.zip › data2/image0252.jpg]

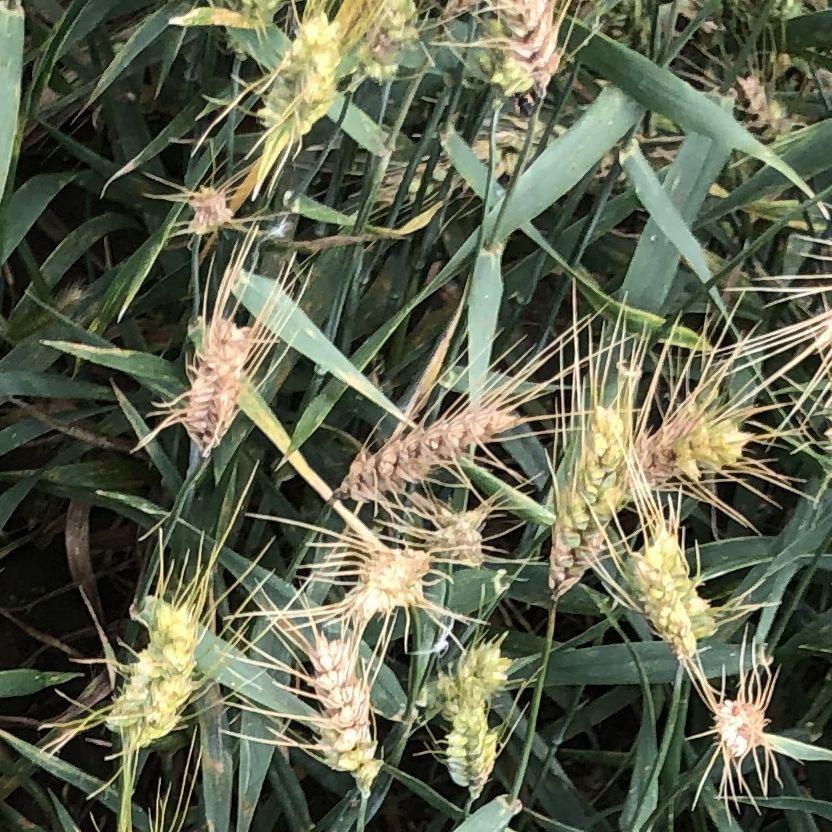

Supplement: Supplemental Information 3 [file peerj-cs-10-1948-s003.zip › data2/image0253.jpg]

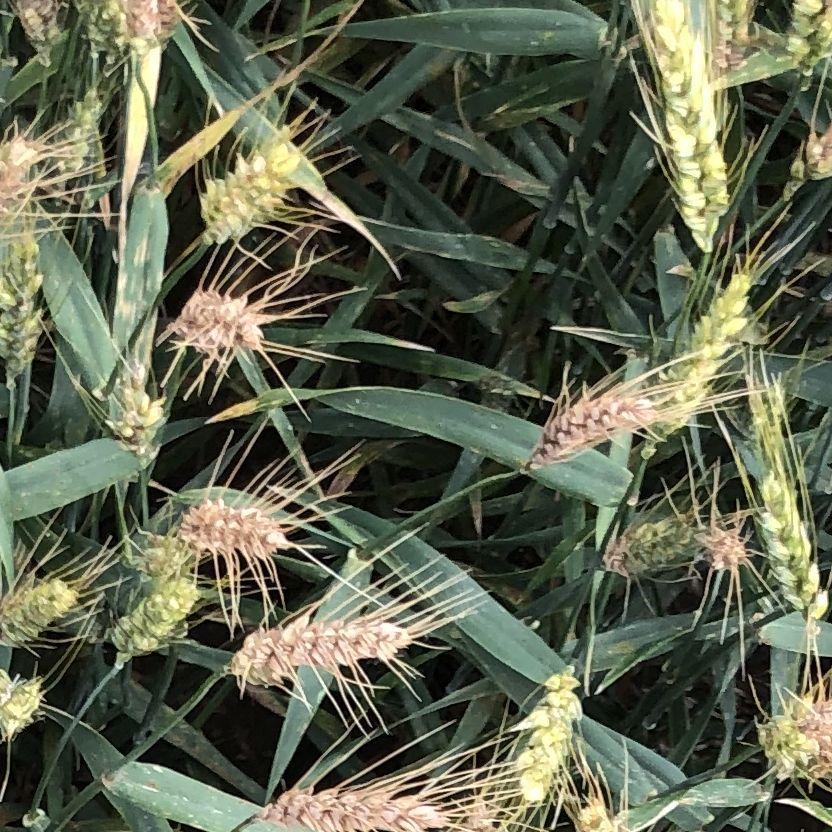

Supplement: Supplemental Information 3 [file peerj-cs-10-1948-s003.zip › data2/image0254.jpg]

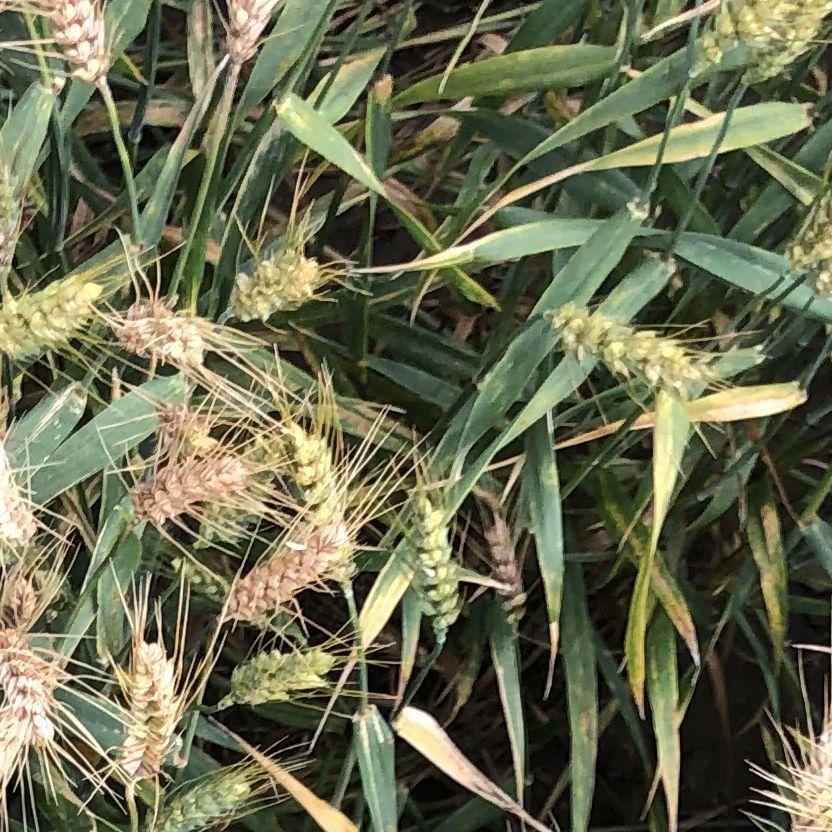

Supplement: Supplemental Information 3 [file peerj-cs-10-1948-s003.zip › data2/image0255.jpg]

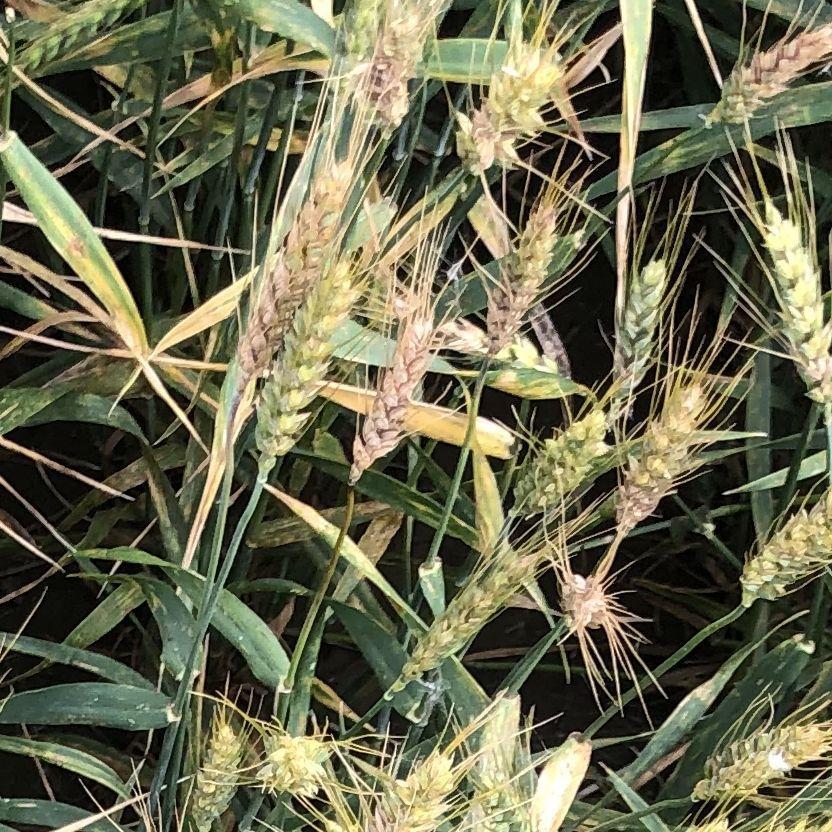

Supplement: Supplemental Information 3 [file peerj-cs-10-1948-s003.zip › data2/image0256.jpg]

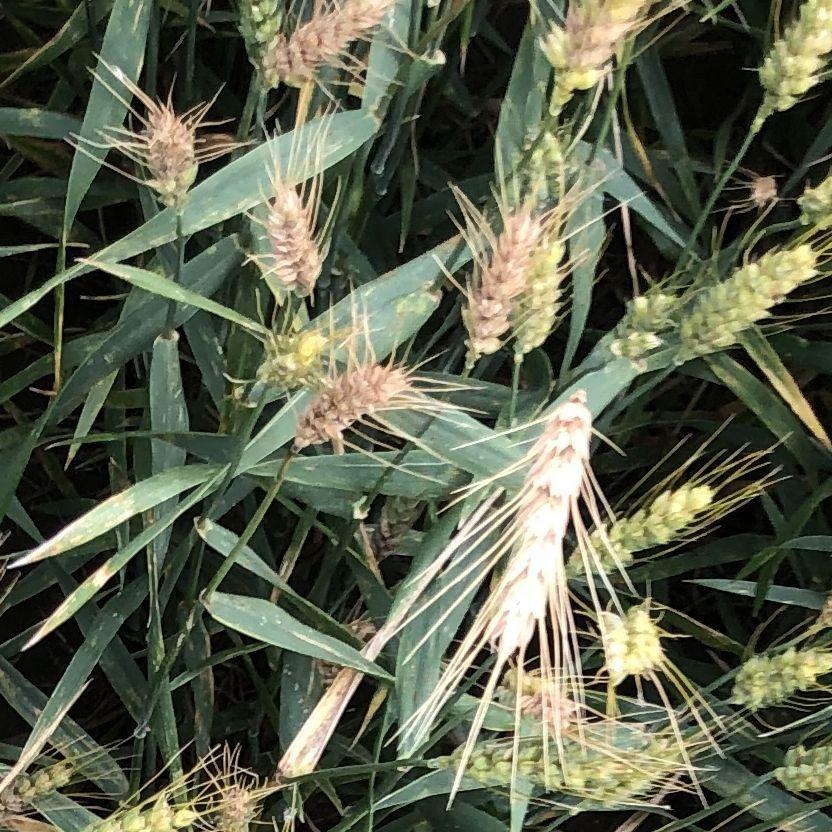

Supplement: Supplemental Information 3 [file peerj-cs-10-1948-s003.zip › data2/image0257.jpg]

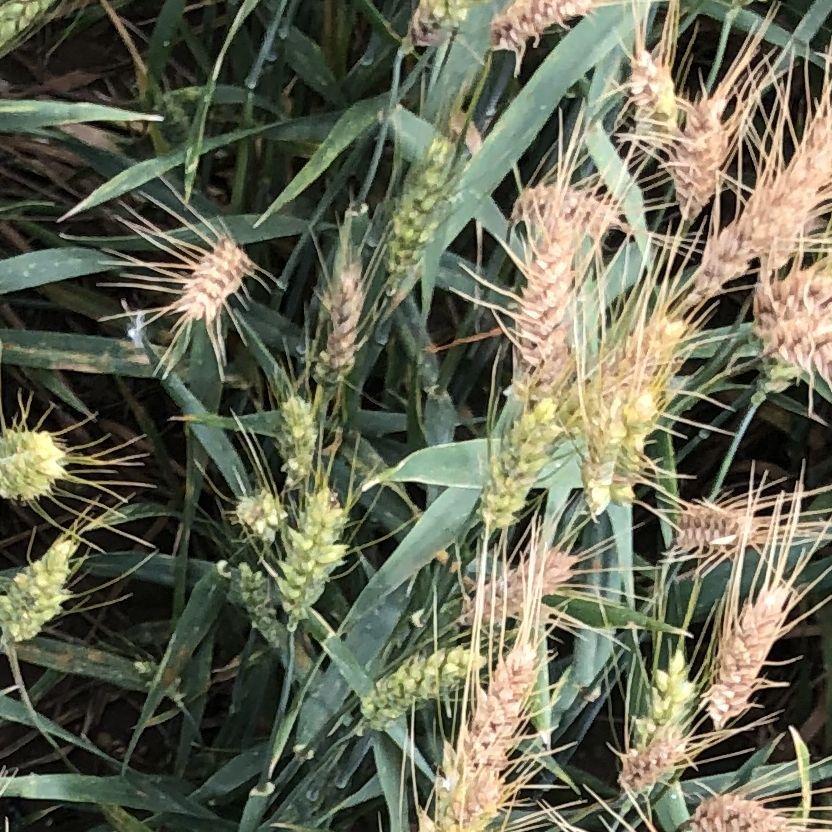

Supplement: Supplemental Information 3 [file peerj-cs-10-1948-s003.zip › data2/image0258.jpg]

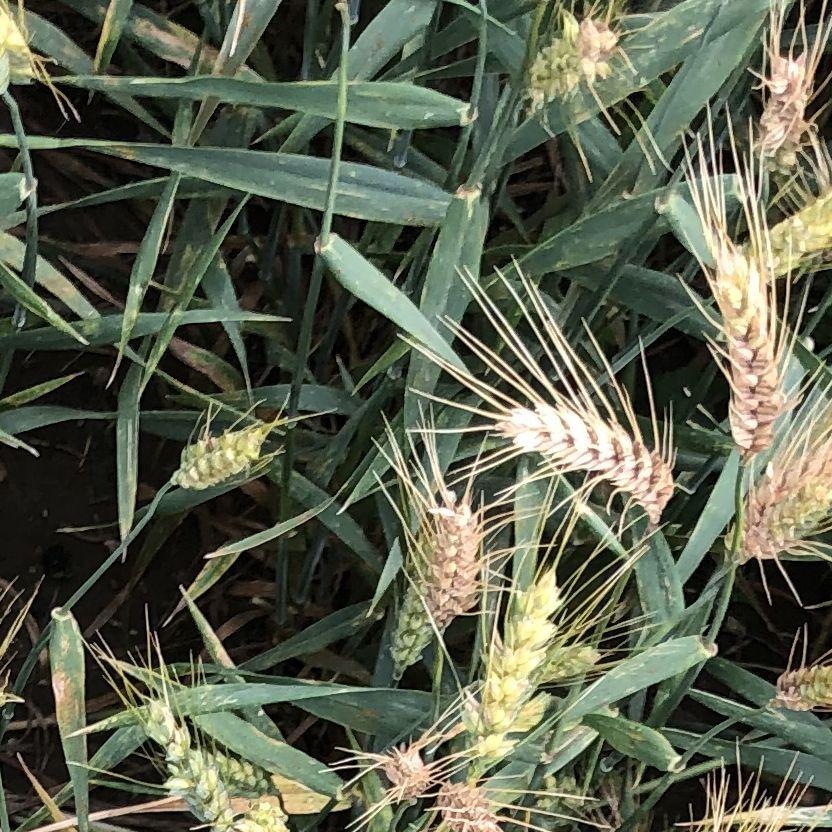

Supplement: Supplemental Information 3 [file peerj-cs-10-1948-s003.zip › data2/image0259.jpg]

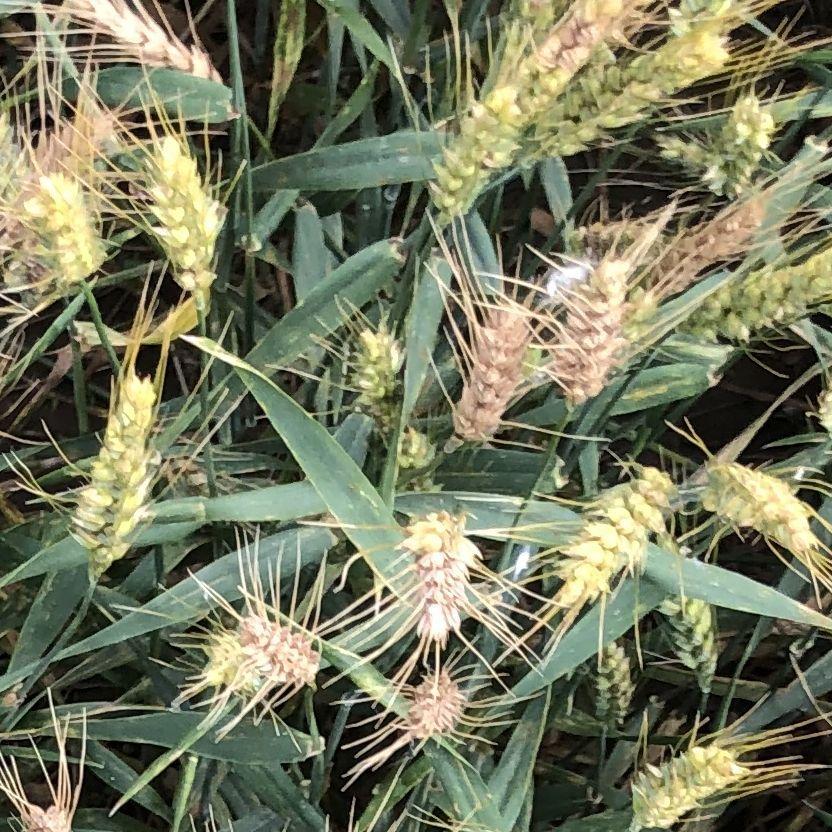

Supplement: Supplemental Information 3 [file peerj-cs-10-1948-s003.zip › data2/image0260.jpg]

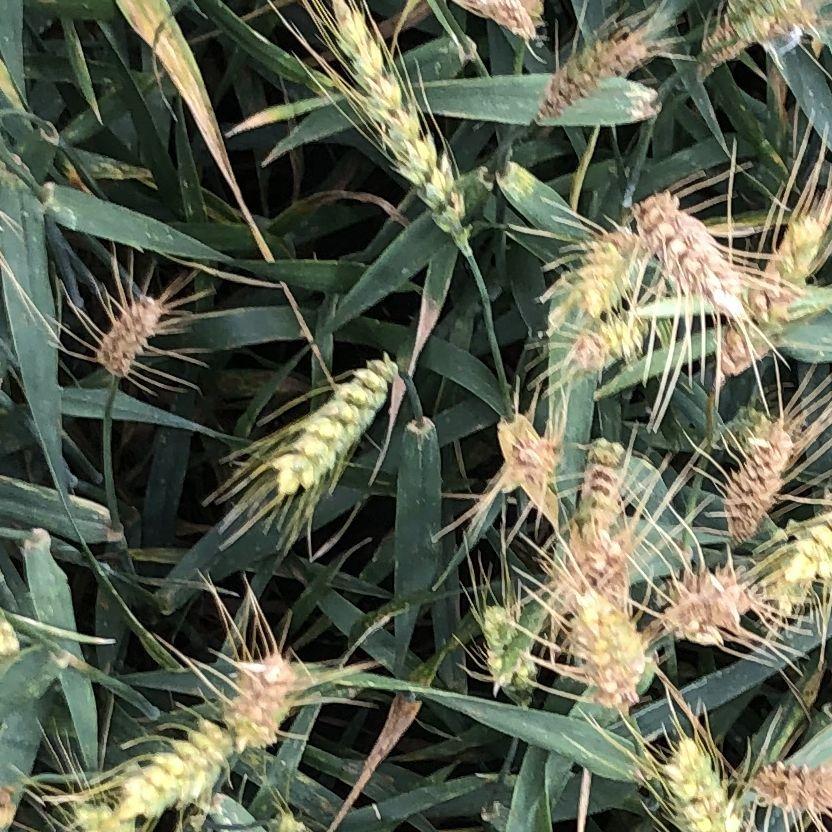

Supplement: Supplemental Information 3 [file peerj-cs-10-1948-s003.zip › data2/image0261.jpg]

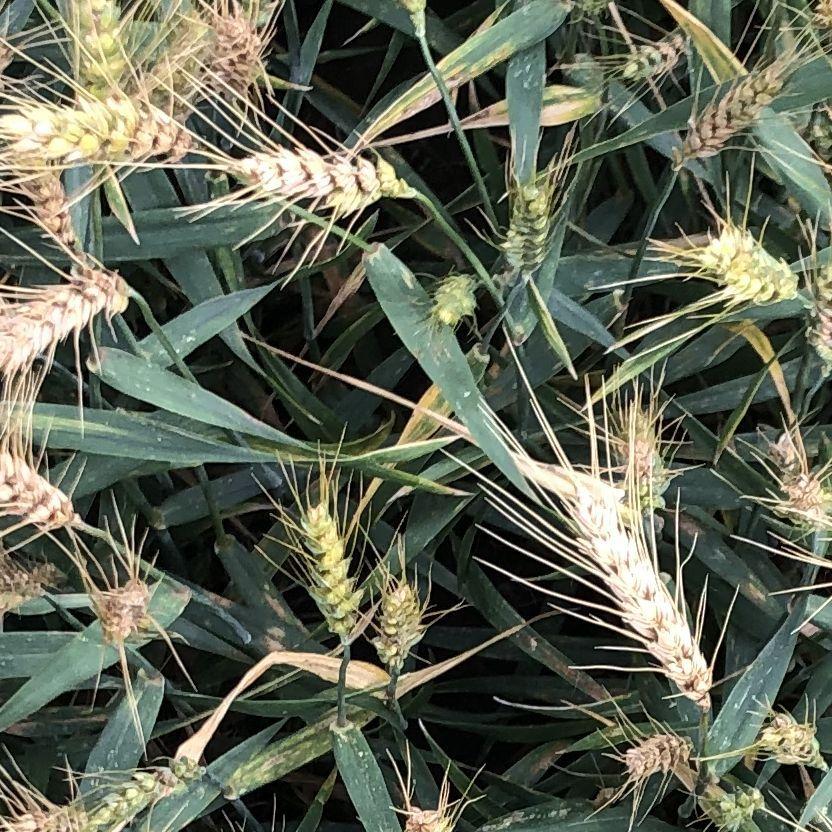

Supplement: Supplemental Information 3 [file peerj-cs-10-1948-s003.zip › data2/image0262.jpg]

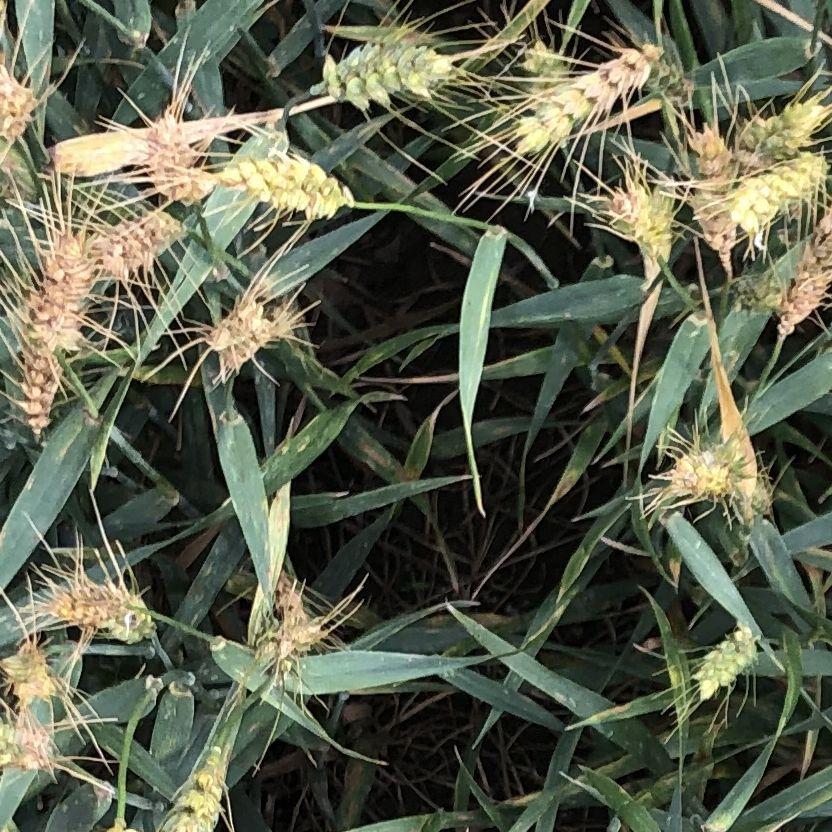

Supplement: Supplemental Information 3 [file peerj-cs-10-1948-s003.zip › data2/image0263.jpg]

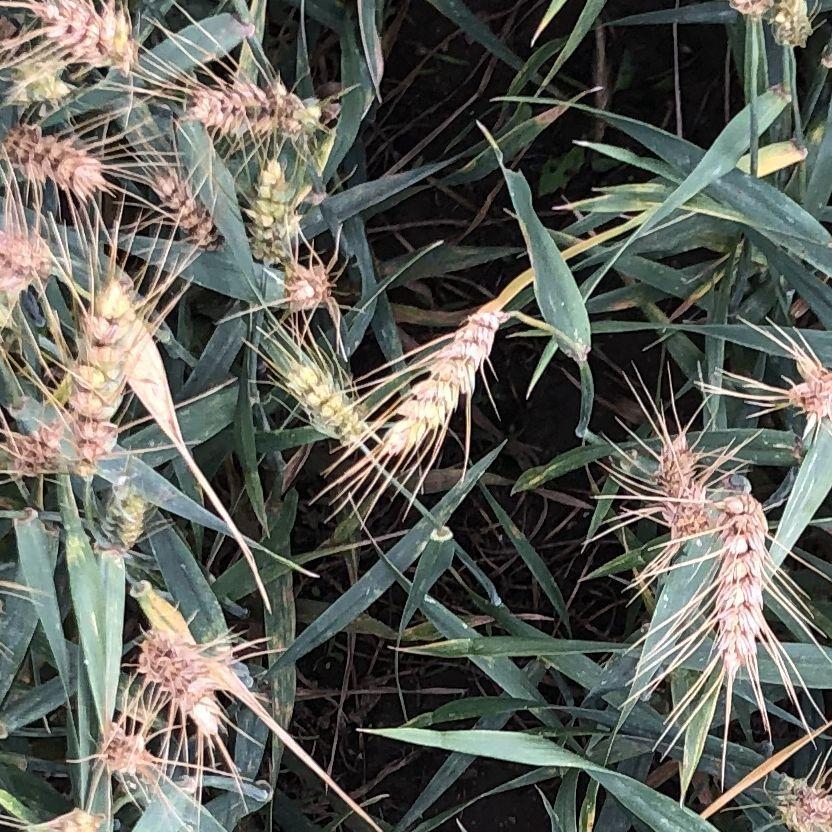

Supplement: Supplemental Information 3 [file peerj-cs-10-1948-s003.zip › data2/image0265.jpg]

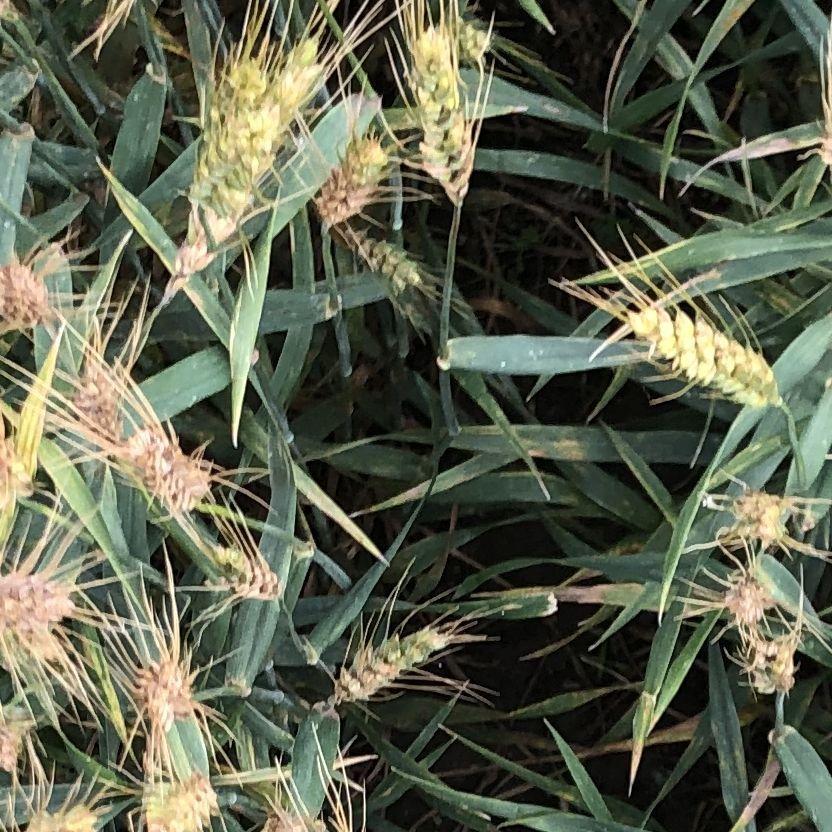

Supplement: Supplemental Information 3 [file peerj-cs-10-1948-s003.zip › data2/image0266.jpg]

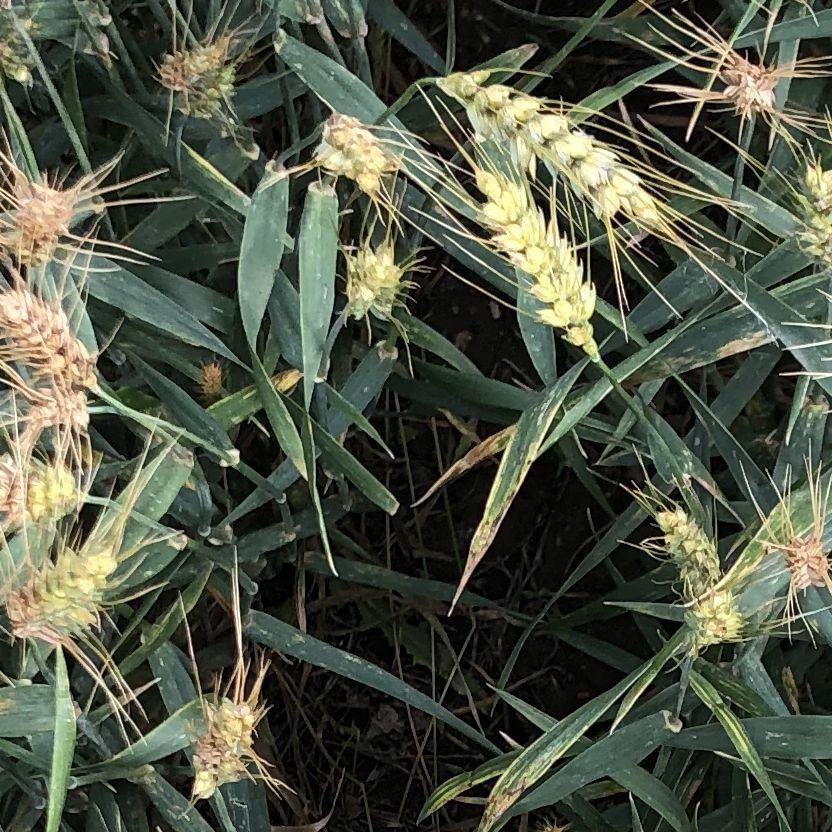

Supplement: Supplemental Information 3 [file peerj-cs-10-1948-s003.zip › data2/image0268.jpg]

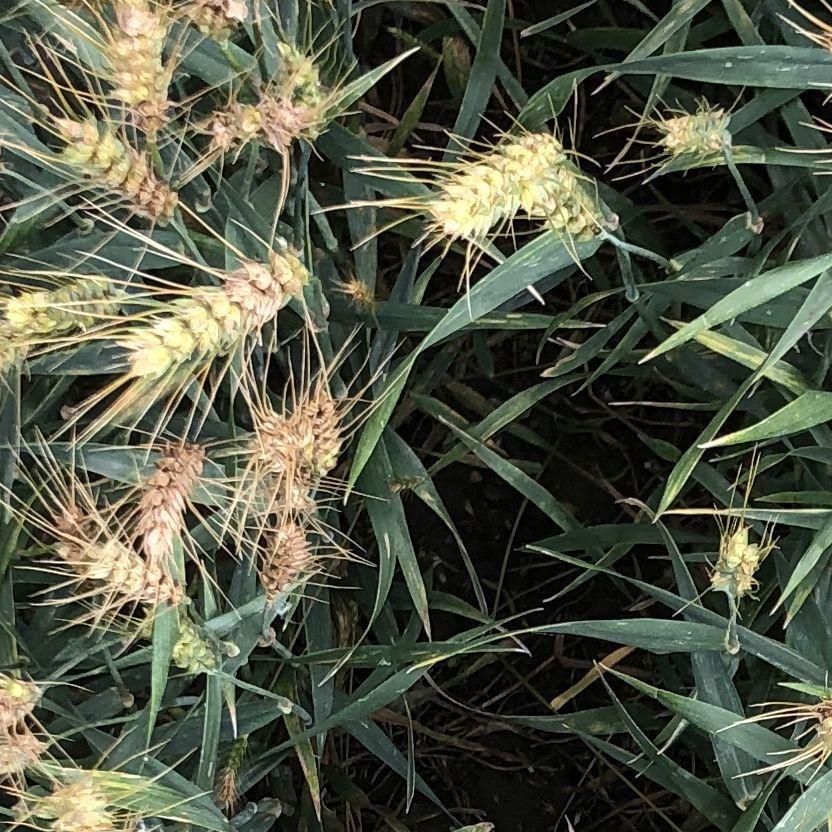

Supplement: Supplemental Information 3 [file peerj-cs-10-1948-s003.zip › data2/image0269.jpg]
