# Supplementary figures and images for: YOLOv8s-CGF: a lightweight model for wheat ear Fusarium head blight detection (part 1 of 3)
Source: PeerJ Comput Sci. 2024 Mar 27;10:e1948. doi: 10.7717/peerj-cs.1948 (PMC11041926; doi:10.7717/peerj-cs.1948)

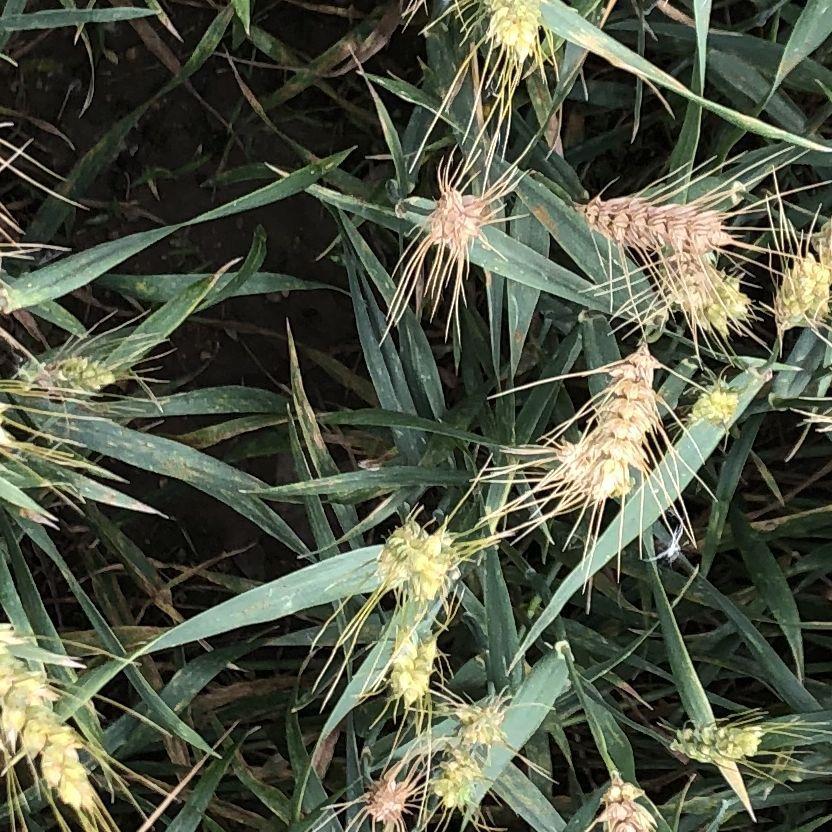

Supplement: Supplemental Information 2 [file peerj-cs-10-1948-s002.zip › data1/image0001.jpg]

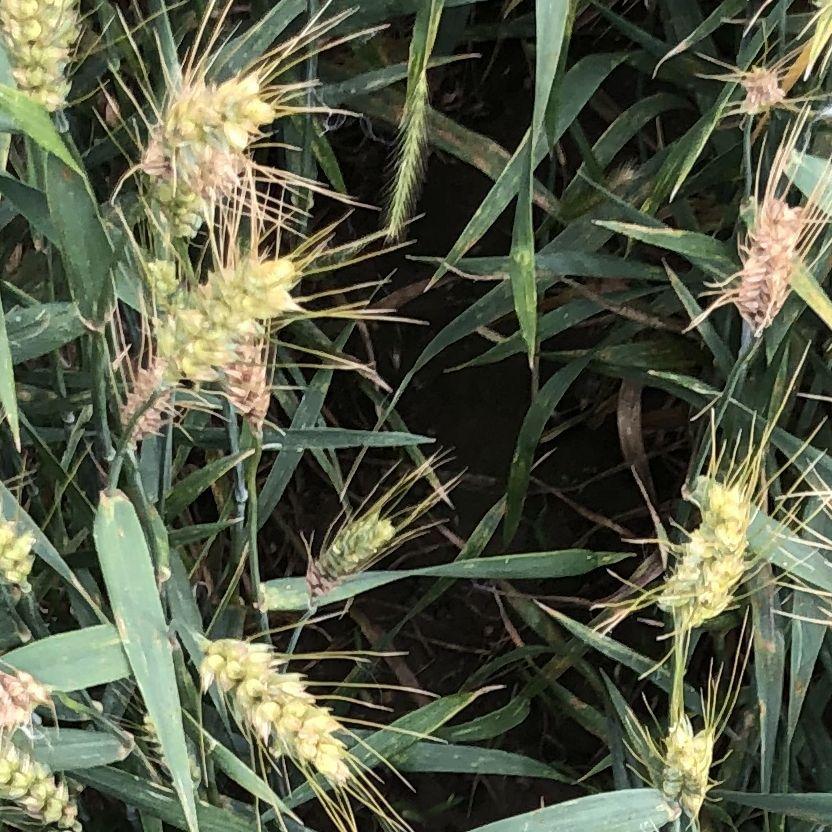

Supplement: Supplemental Information 2 [file peerj-cs-10-1948-s002.zip › data1/image0011.jpg]

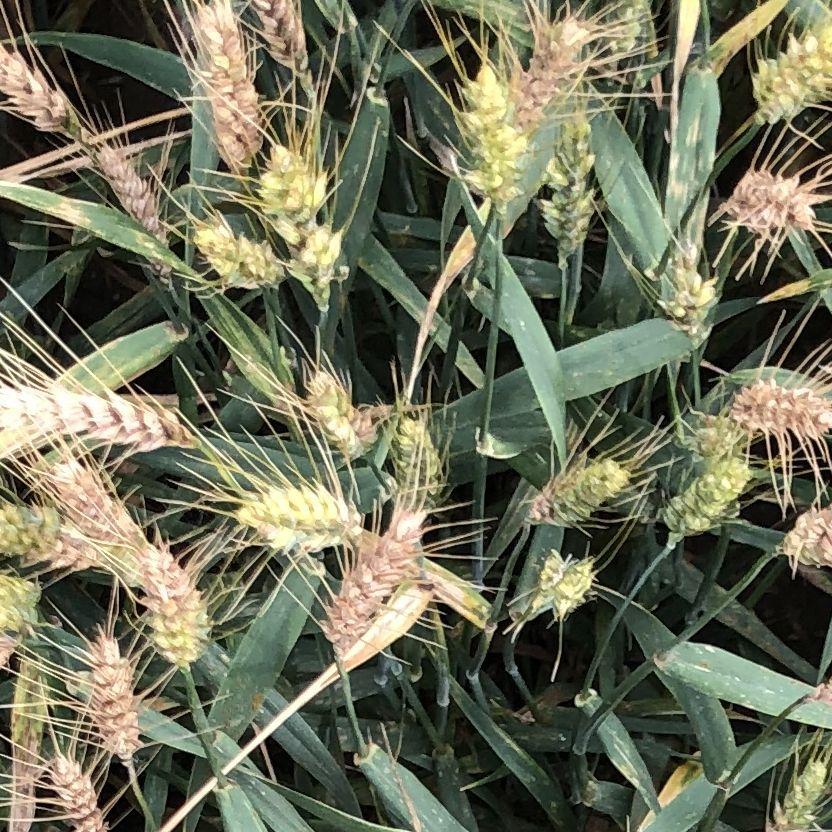

Supplement: Supplemental Information 2 [file peerj-cs-10-1948-s002.zip › data1/image0012.jpg]

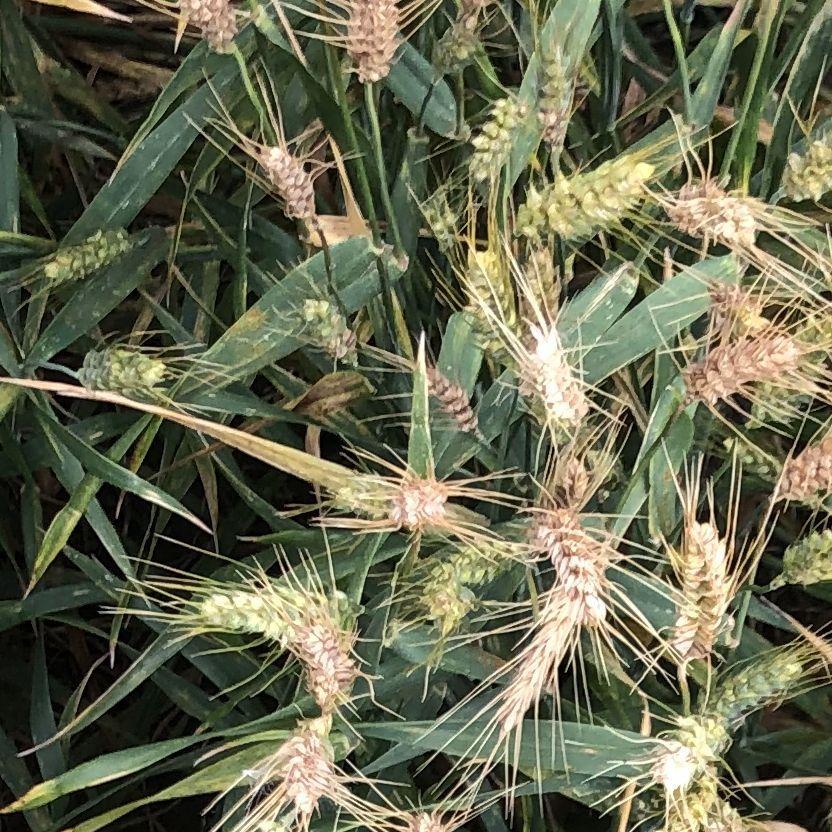

Supplement: Supplemental Information 2 [file peerj-cs-10-1948-s002.zip › data1/image0013.jpg]

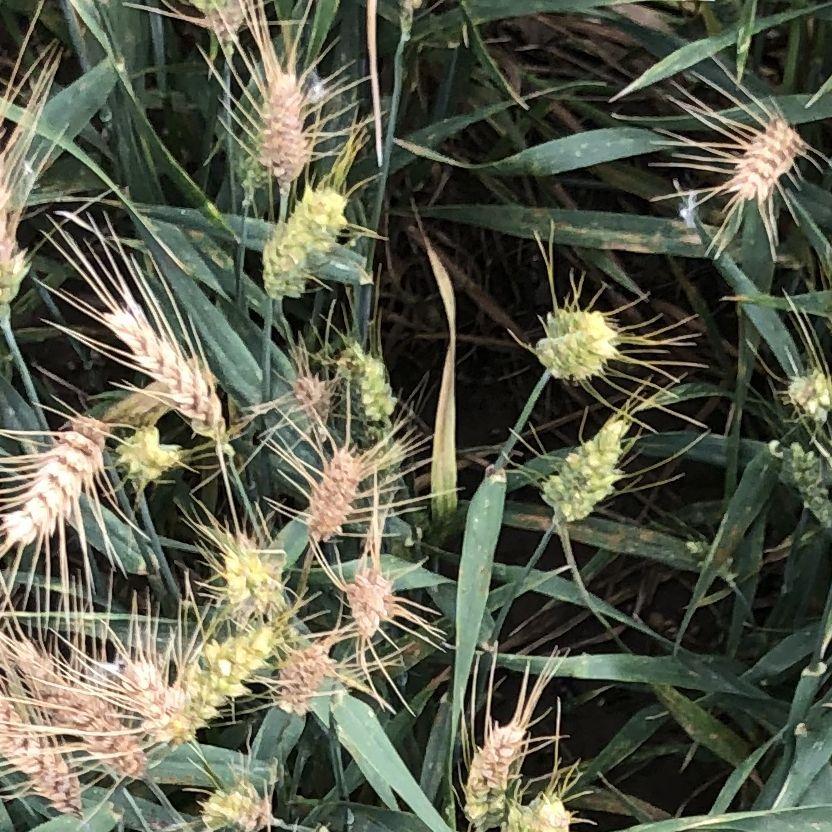

Supplement: Supplemental Information 2 [file peerj-cs-10-1948-s002.zip › data1/image0016.jpg]

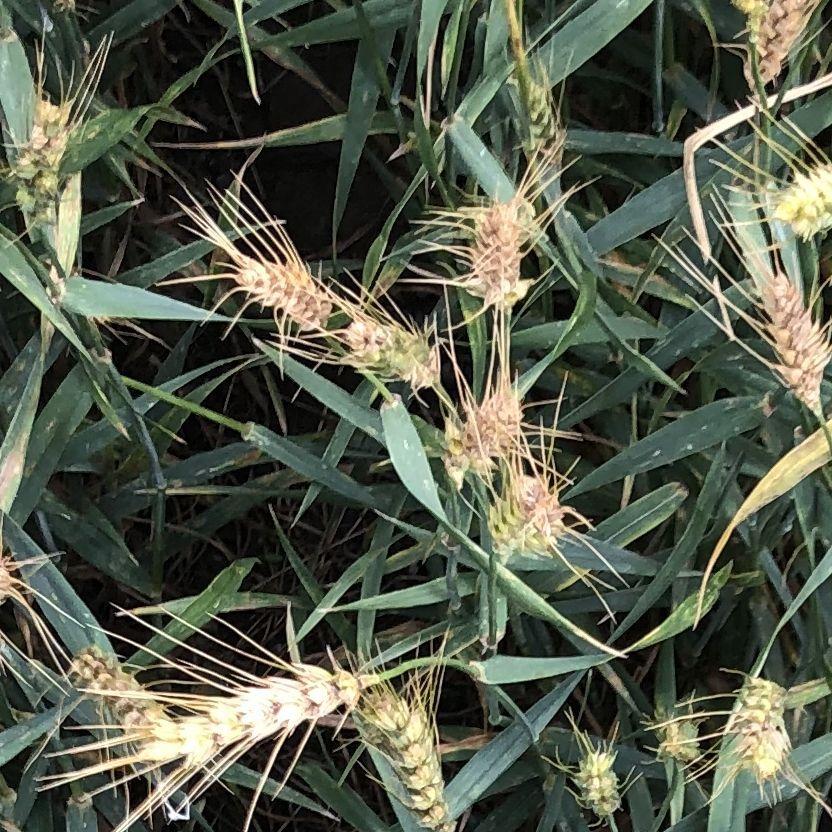

Supplement: Supplemental Information 2 [file peerj-cs-10-1948-s002.zip › data1/image0020.jpg]

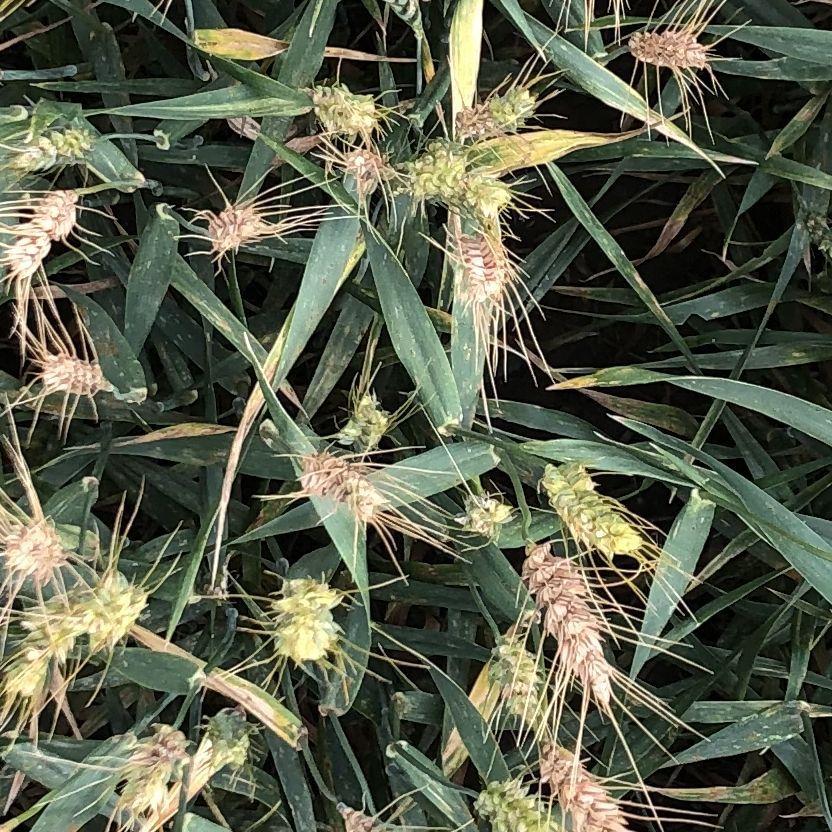

Supplement: Supplemental Information 2 [file peerj-cs-10-1948-s002.zip › data1/image0021.jpg]

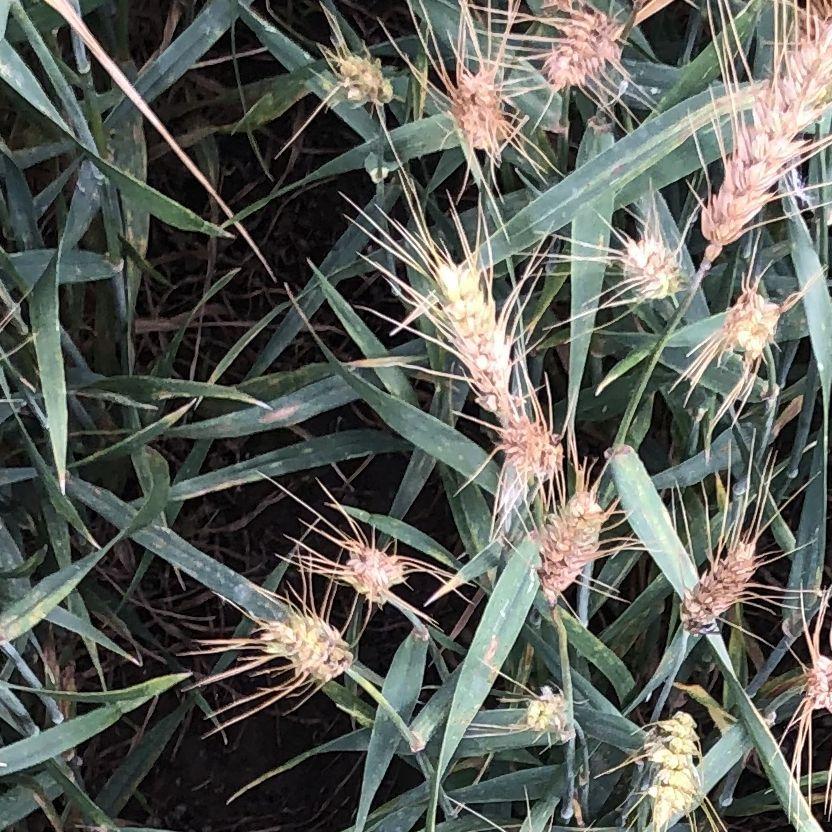

Supplement: Supplemental Information 2 [file peerj-cs-10-1948-s002.zip › data1/image0022.jpg]

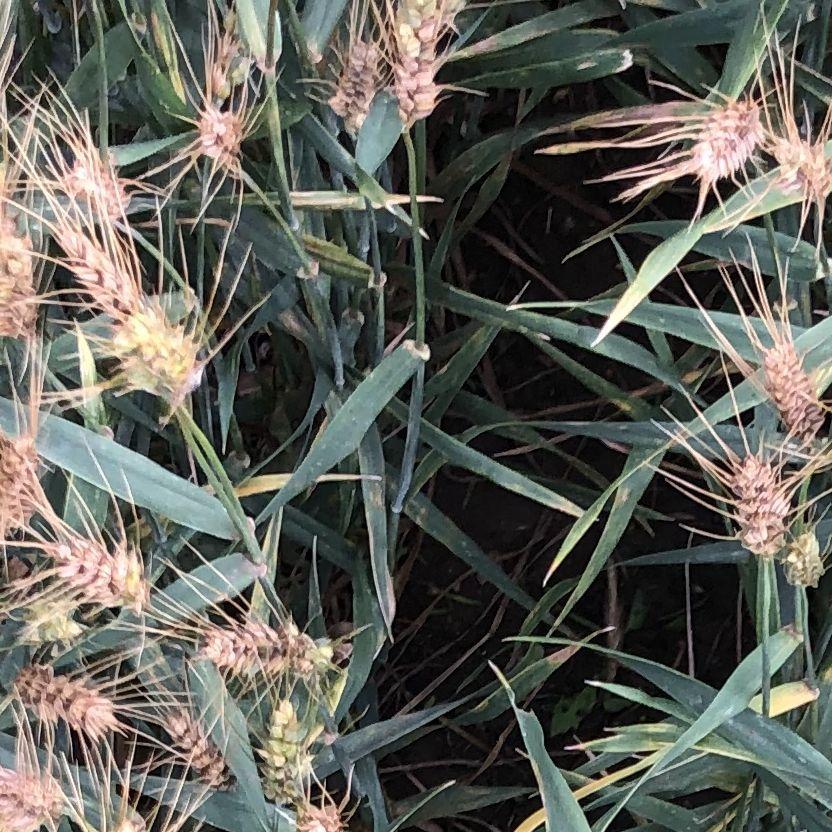

Supplement: Supplemental Information 2 [file peerj-cs-10-1948-s002.zip › data1/image0023.jpg]

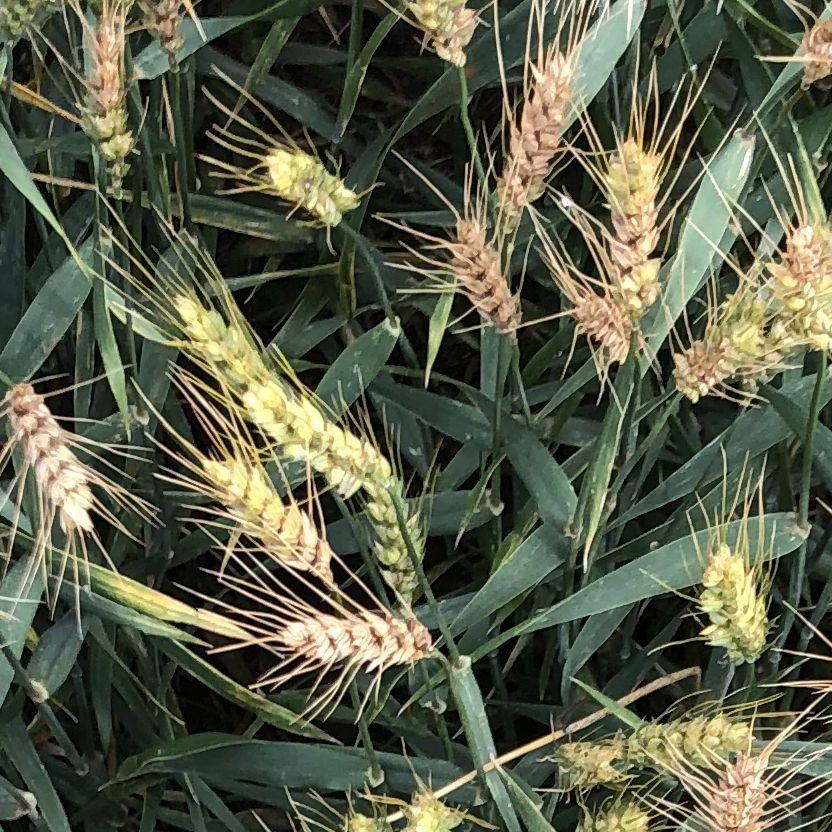

Supplement: Supplemental Information 2 [file peerj-cs-10-1948-s002.zip › data1/image0024.jpg]

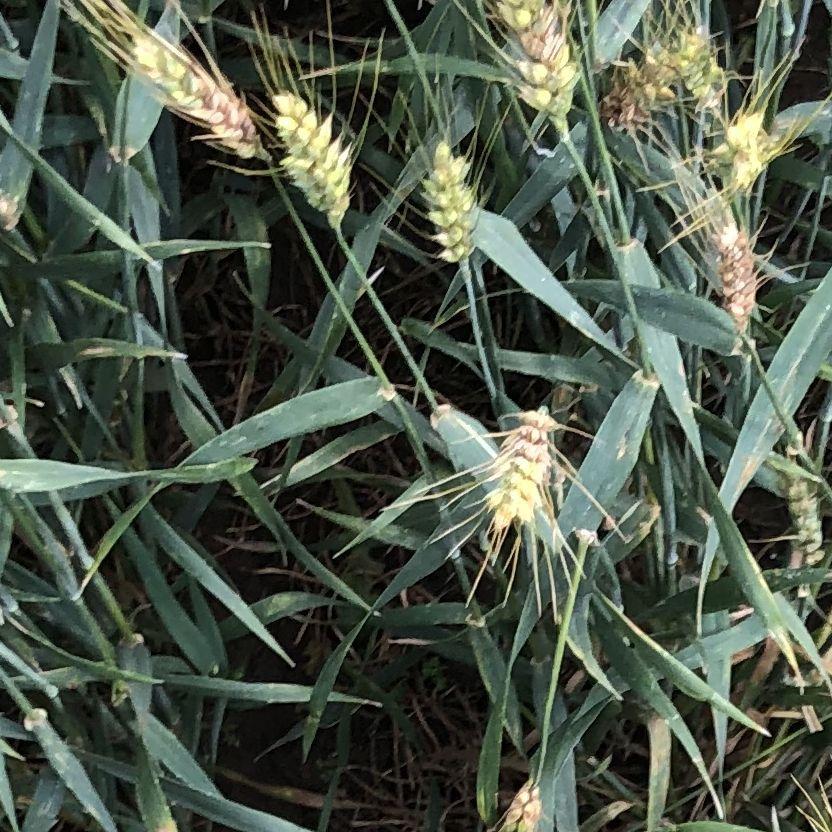

Supplement: Supplemental Information 2 [file peerj-cs-10-1948-s002.zip › data1/image0025.jpg]

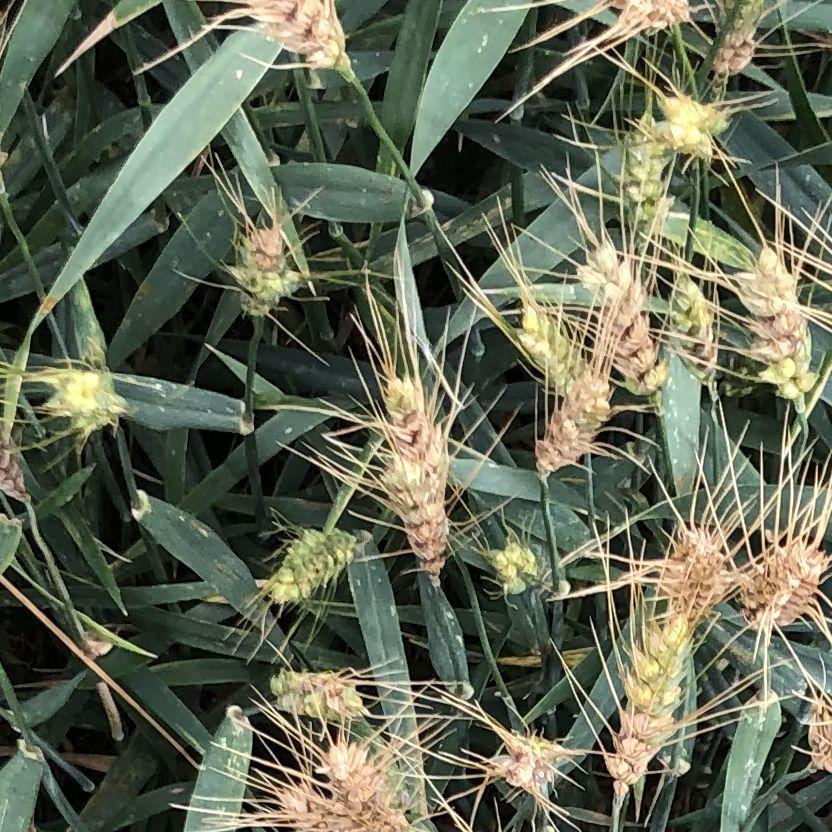

Supplement: Supplemental Information 2 [file peerj-cs-10-1948-s002.zip › data1/image0026.jpg]

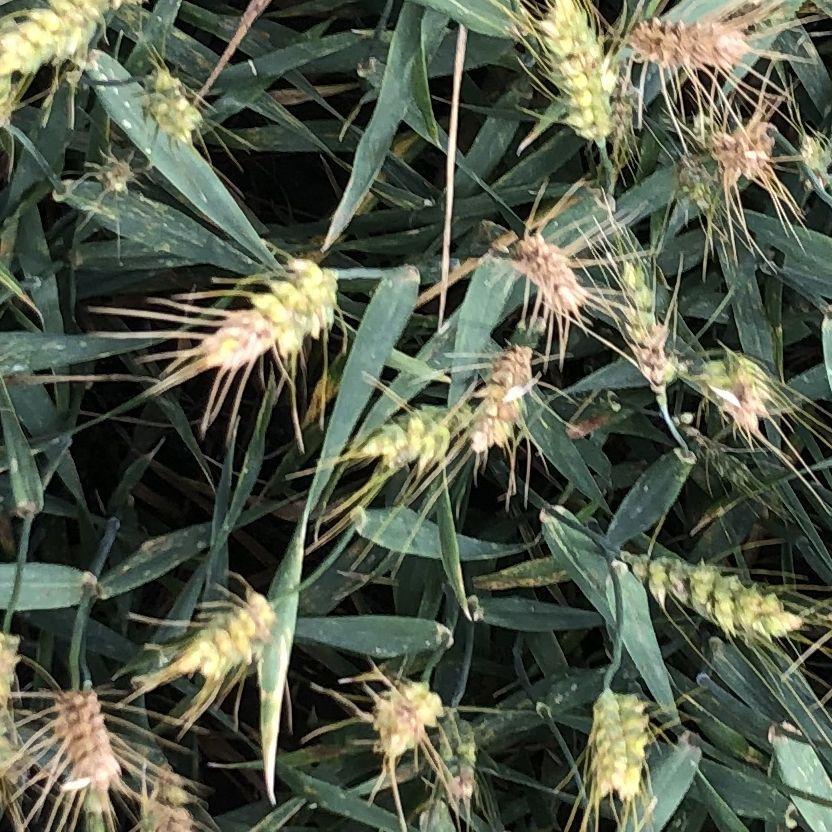

Supplement: Supplemental Information 2 [file peerj-cs-10-1948-s002.zip › data1/image0027.jpg]

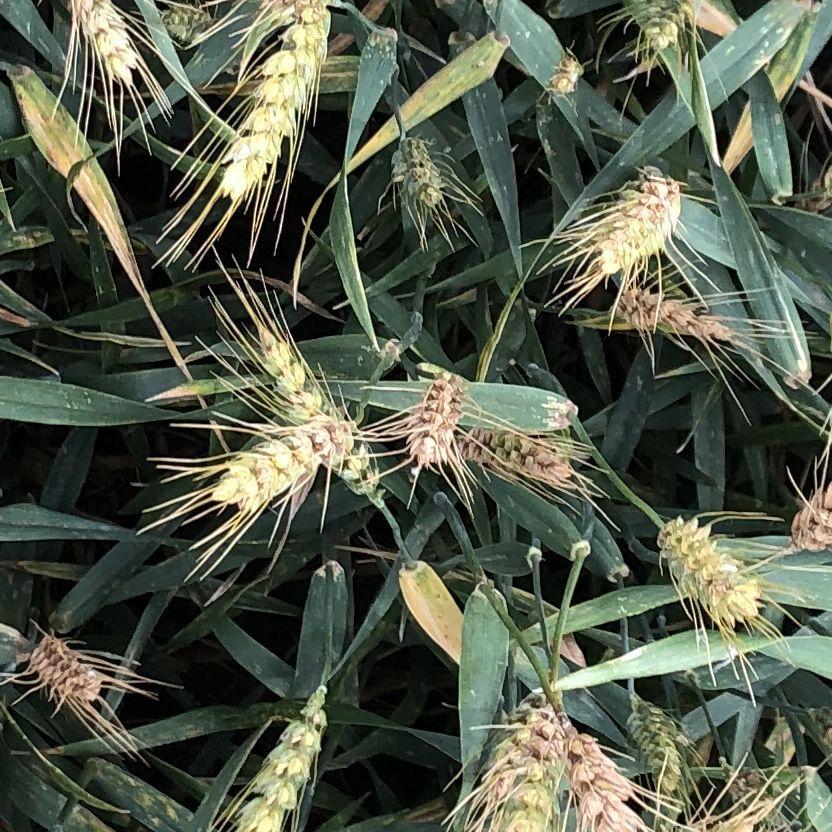

Supplement: Supplemental Information 2 [file peerj-cs-10-1948-s002.zip › data1/image0028.jpg]

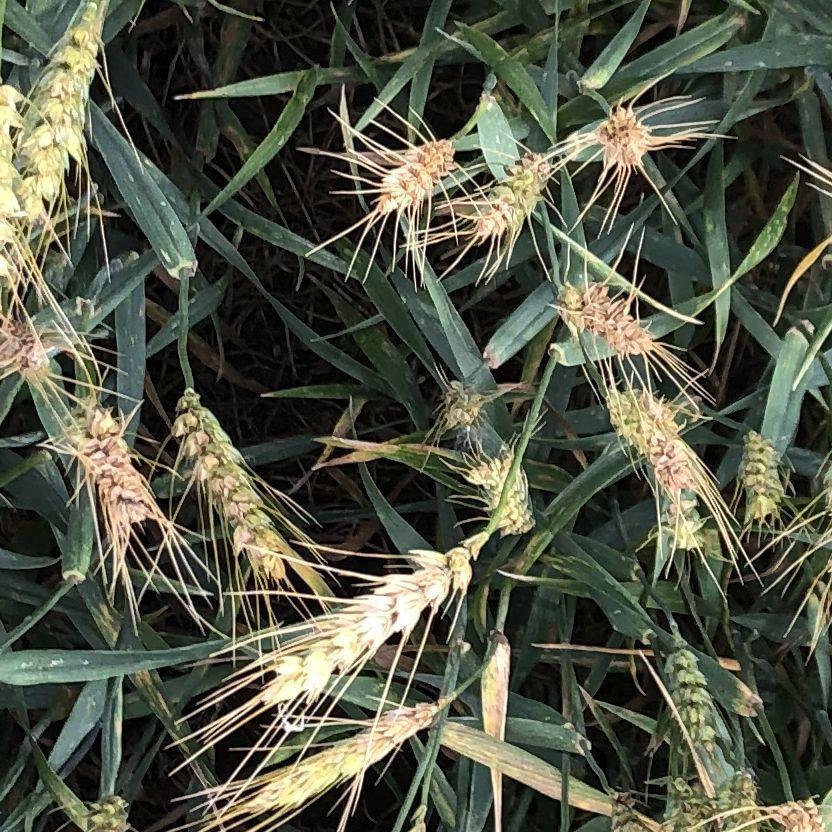

Supplement: Supplemental Information 2 [file peerj-cs-10-1948-s002.zip › data1/image0029.jpg]

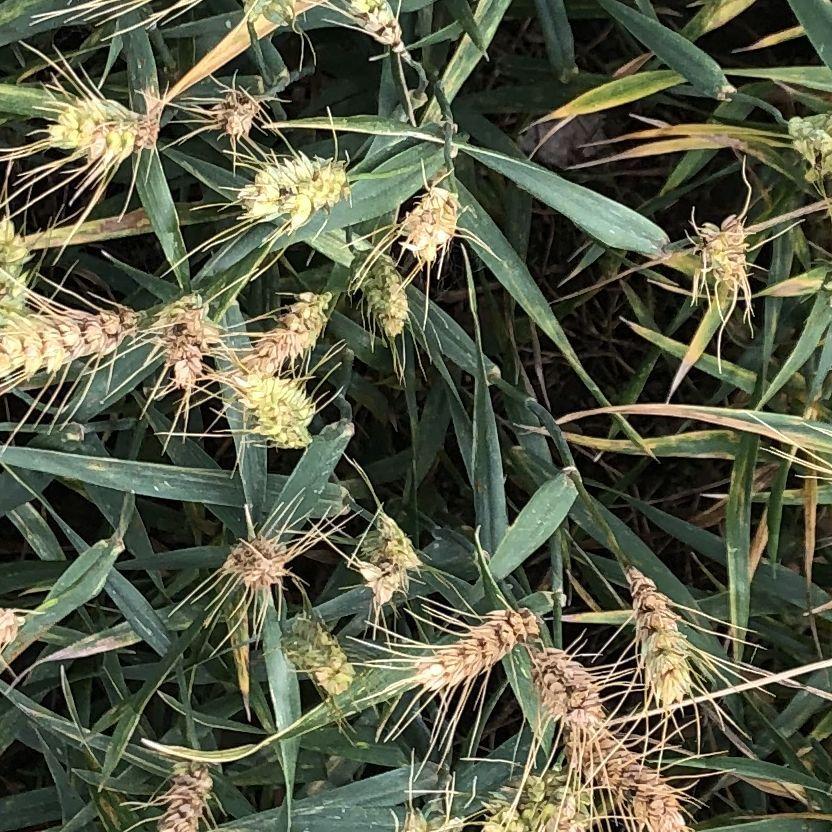

Supplement: Supplemental Information 2 [file peerj-cs-10-1948-s002.zip › data1/image0030.jpg]

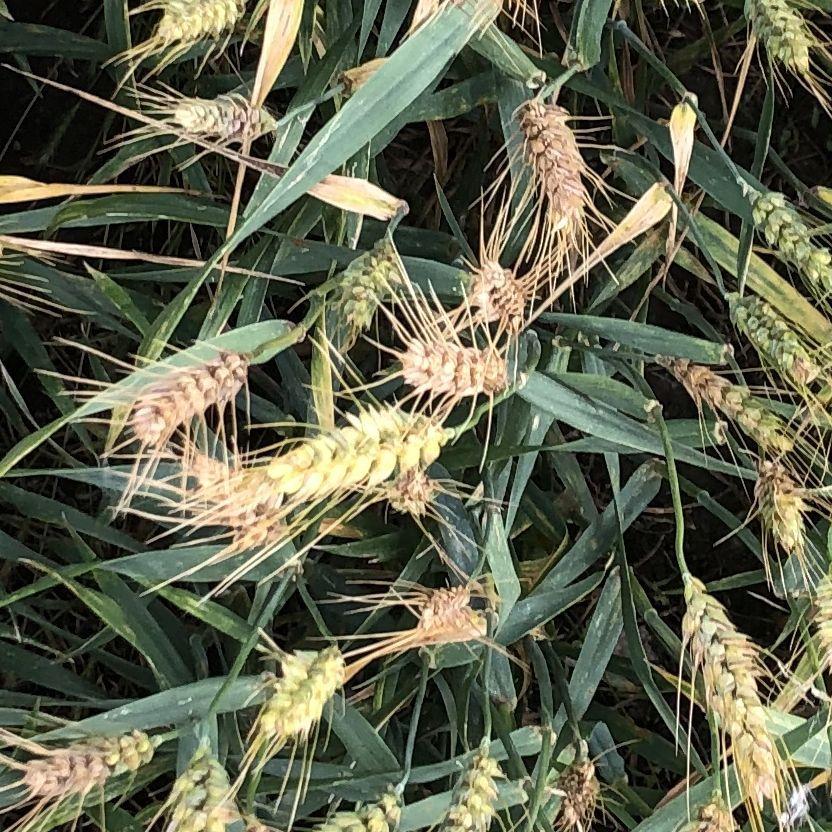

Supplement: Supplemental Information 2 [file peerj-cs-10-1948-s002.zip › data1/image0031.jpg]

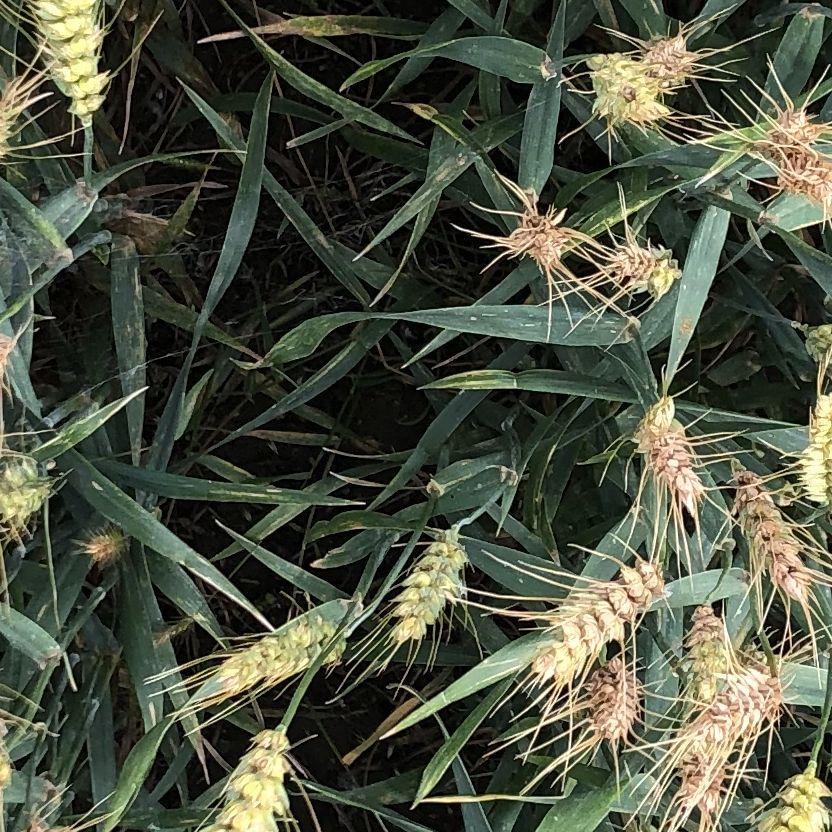

Supplement: Supplemental Information 2 [file peerj-cs-10-1948-s002.zip › data1/image0032.jpg]

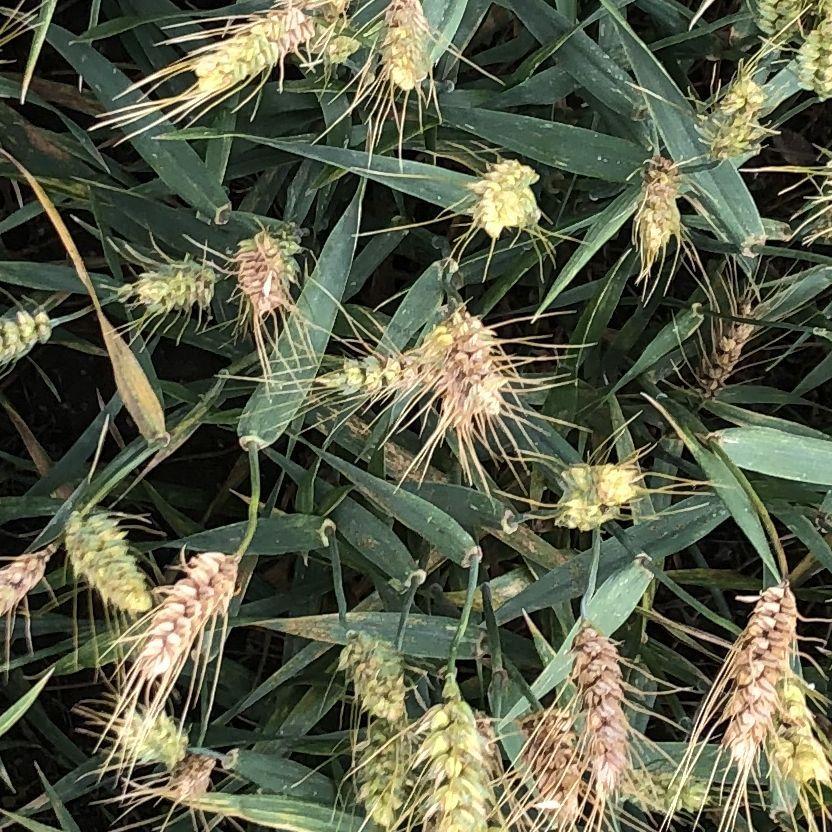

Supplement: Supplemental Information 2 [file peerj-cs-10-1948-s002.zip › data1/image0033.jpg]

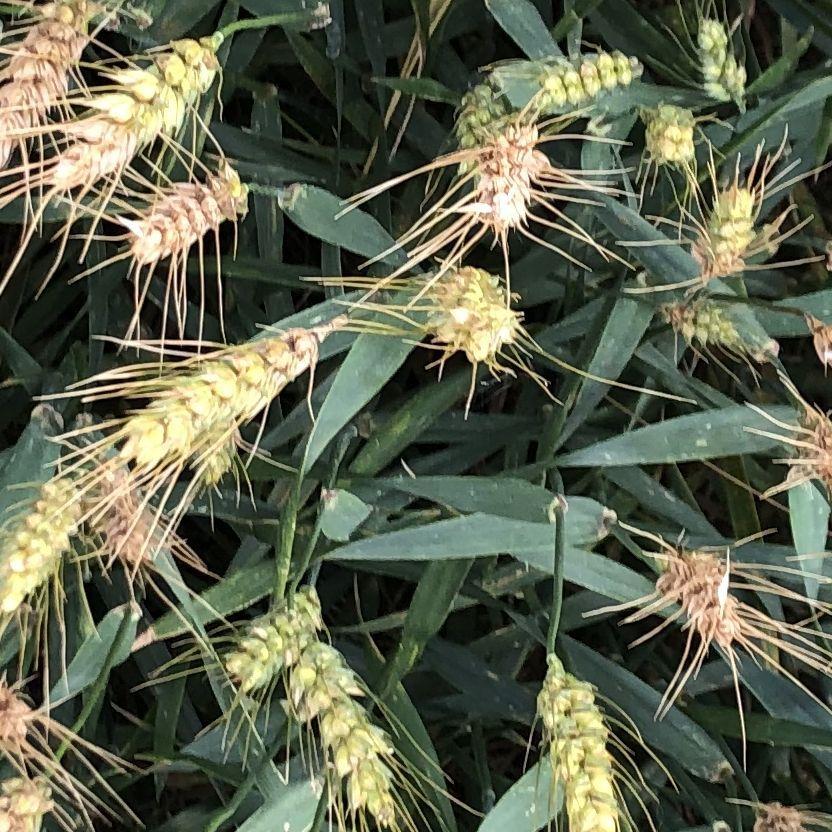

Supplement: Supplemental Information 2 [file peerj-cs-10-1948-s002.zip › data1/image0034.jpg]

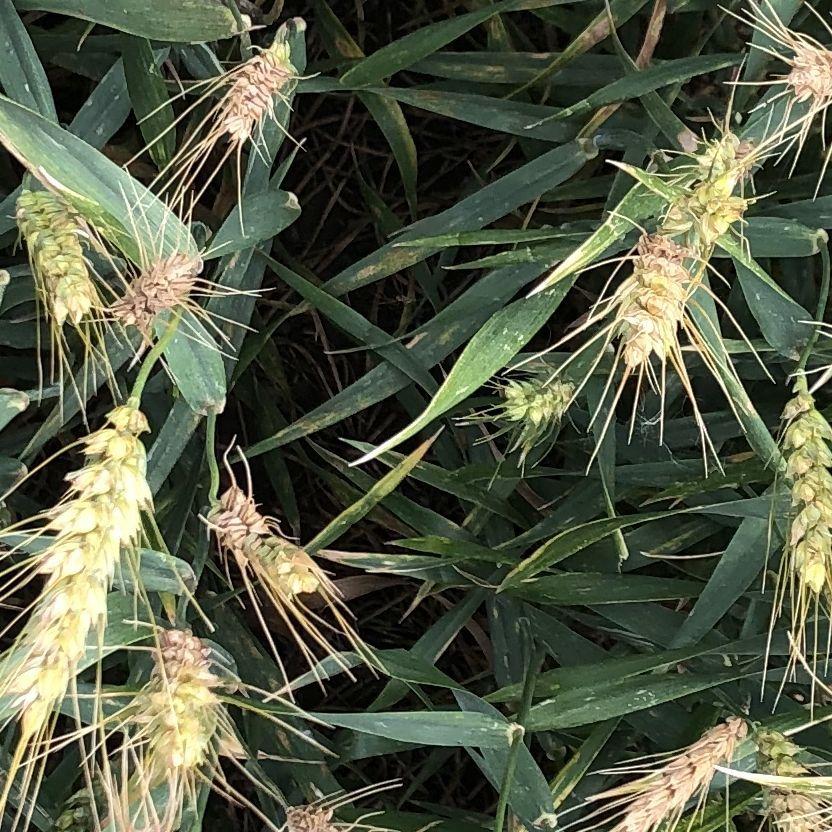

Supplement: Supplemental Information 2 [file peerj-cs-10-1948-s002.zip › data1/image0036.jpg]

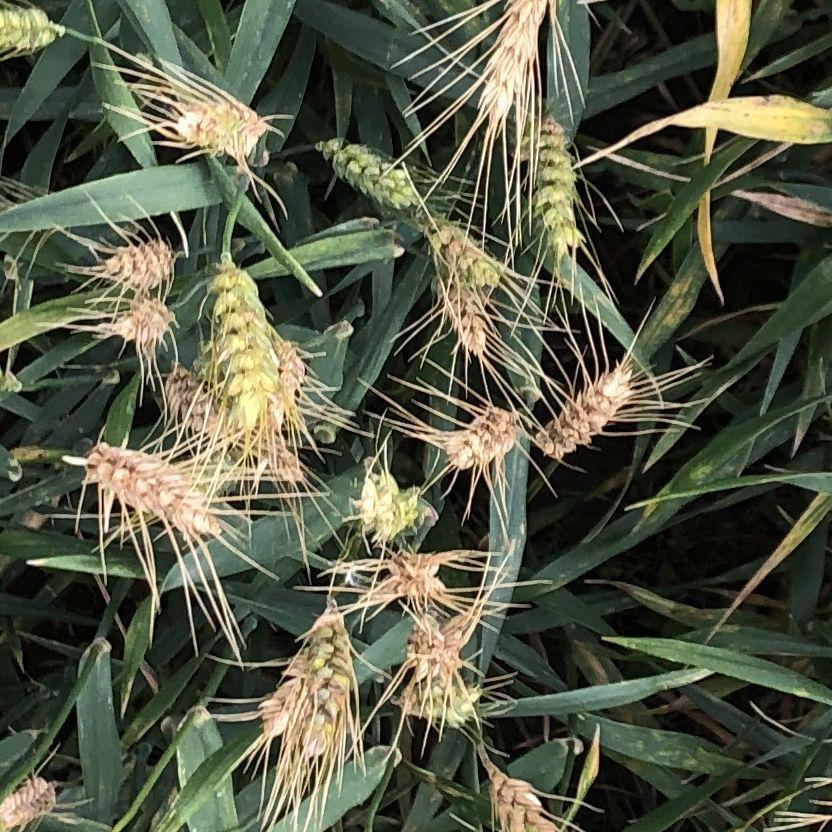

Supplement: Supplemental Information 2 [file peerj-cs-10-1948-s002.zip › data1/image0037.jpg]

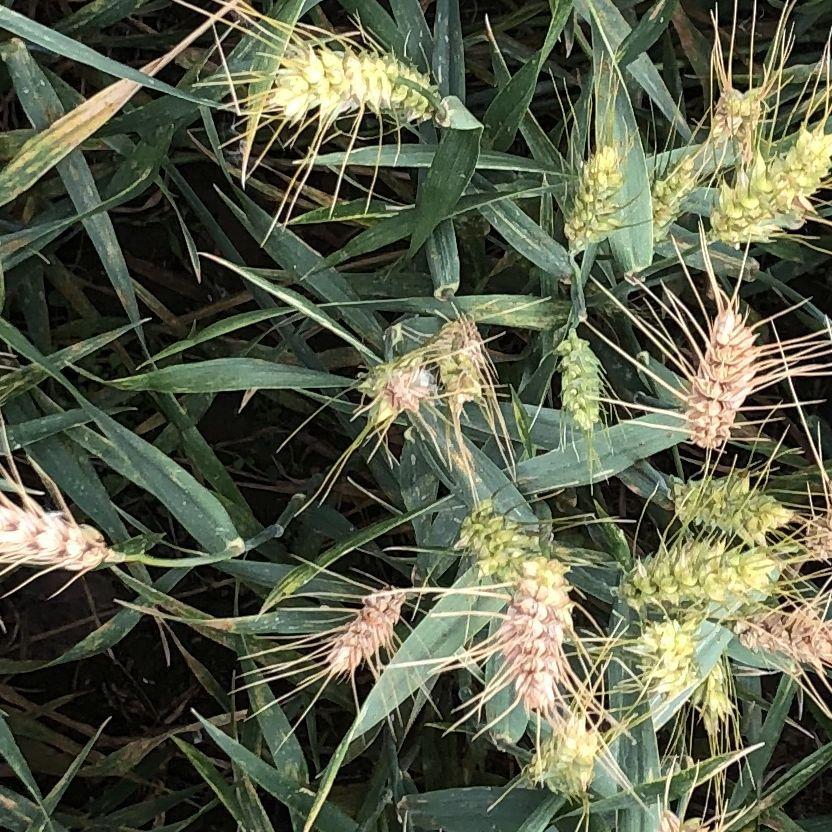

Supplement: Supplemental Information 2 [file peerj-cs-10-1948-s002.zip › data1/image0039.jpg]

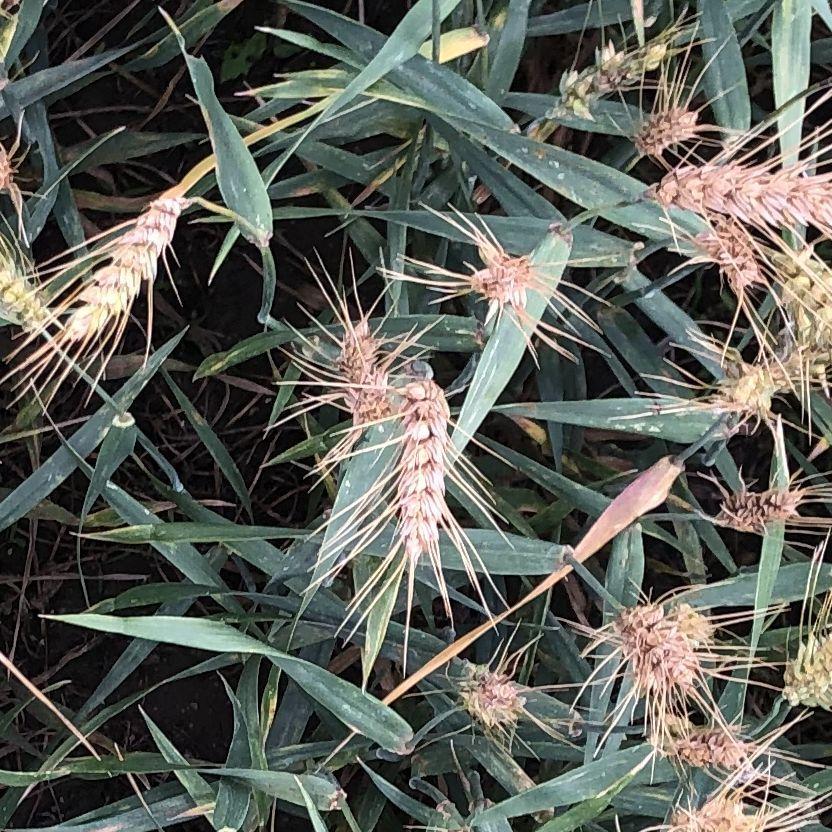

Supplement: Supplemental Information 2 [file peerj-cs-10-1948-s002.zip › data1/image0044.jpg]

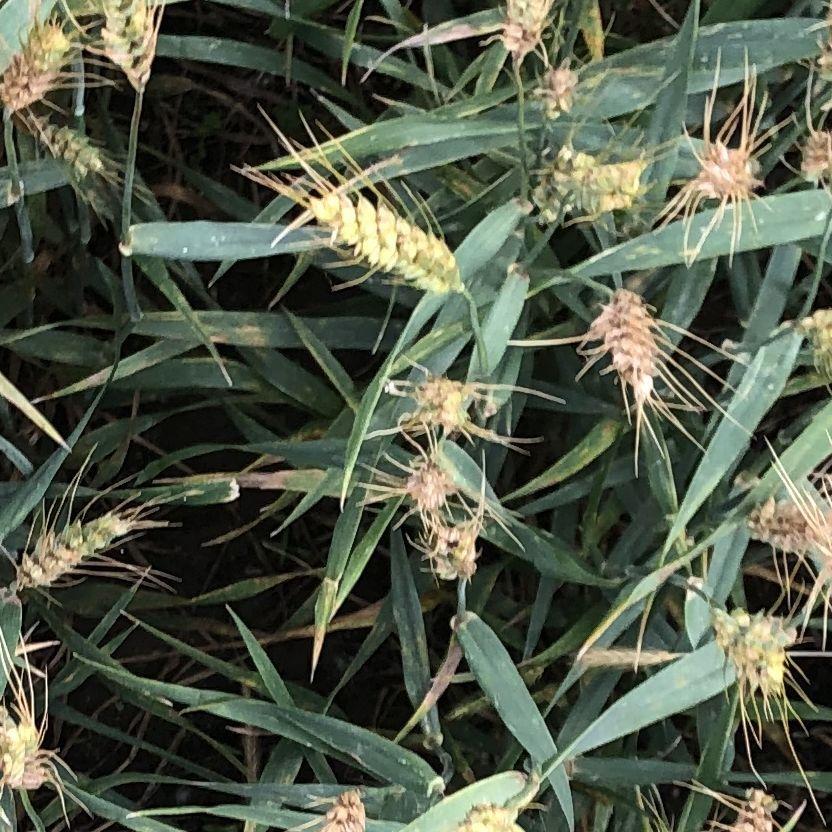

Supplement: Supplemental Information 2 [file peerj-cs-10-1948-s002.zip › data1/image0045.jpg]

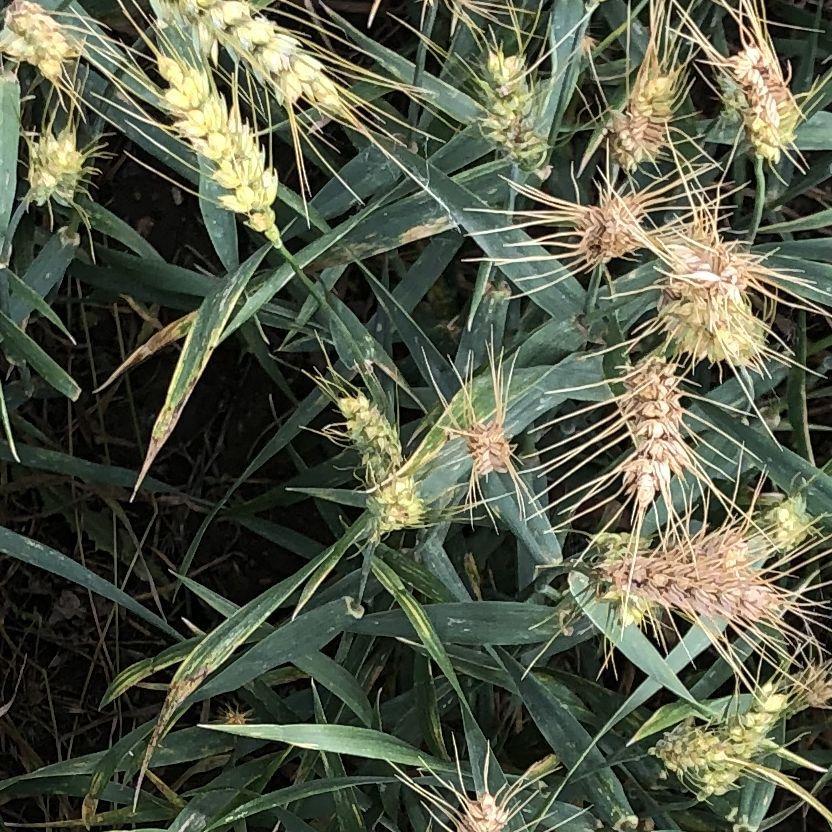

Supplement: Supplemental Information 2 [file peerj-cs-10-1948-s002.zip › data1/image0046.jpg]

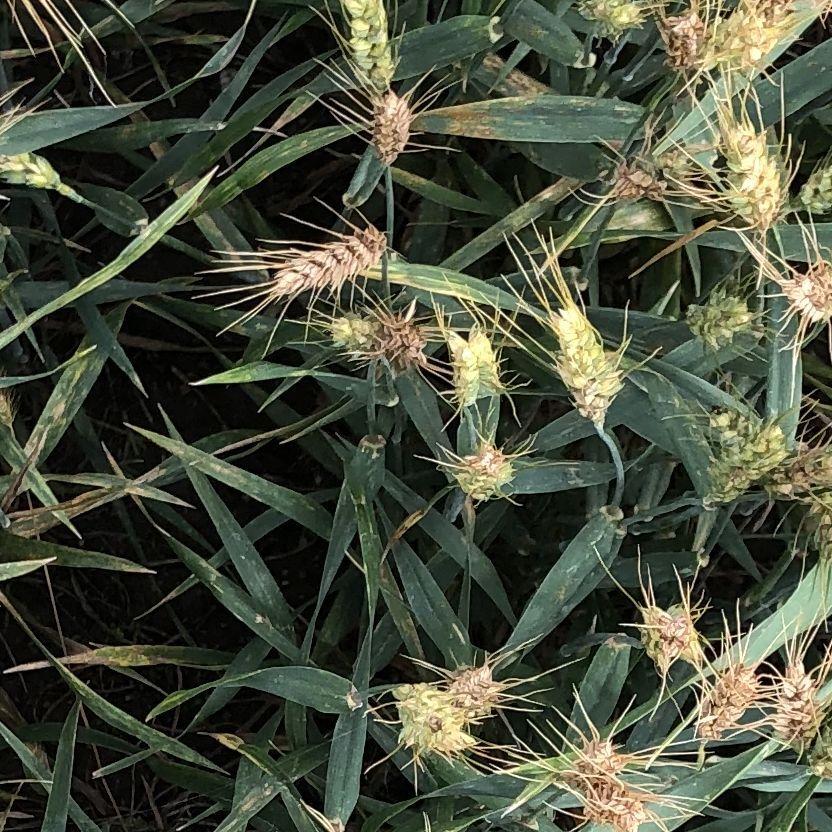

Supplement: Supplemental Information 2 [file peerj-cs-10-1948-s002.zip › data1/image0048.jpg]

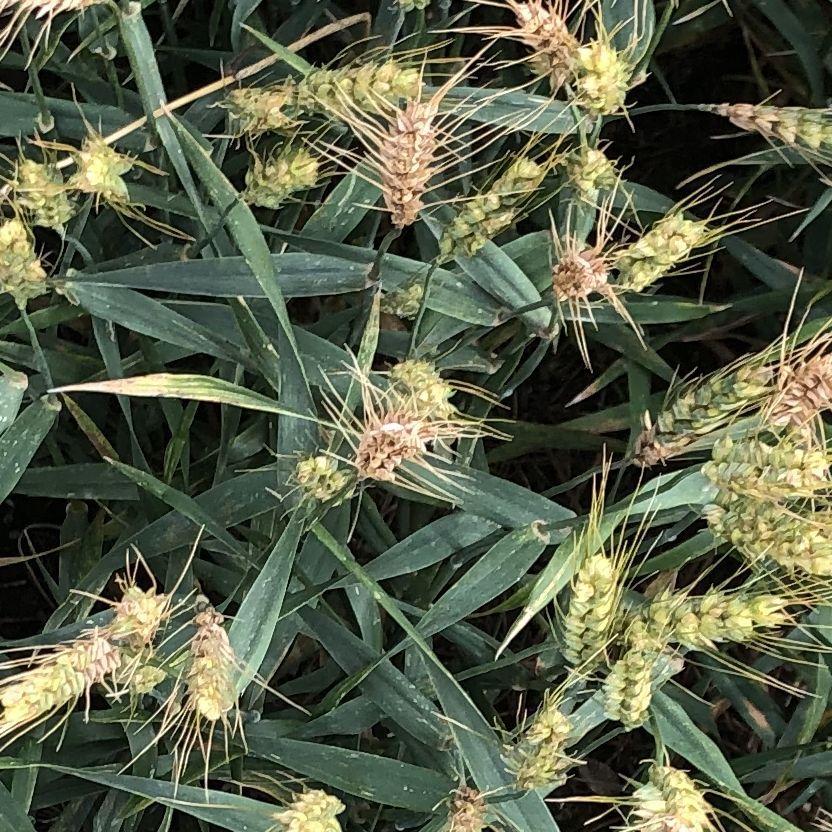

Supplement: Supplemental Information 2 [file peerj-cs-10-1948-s002.zip › data1/image0049.jpg]

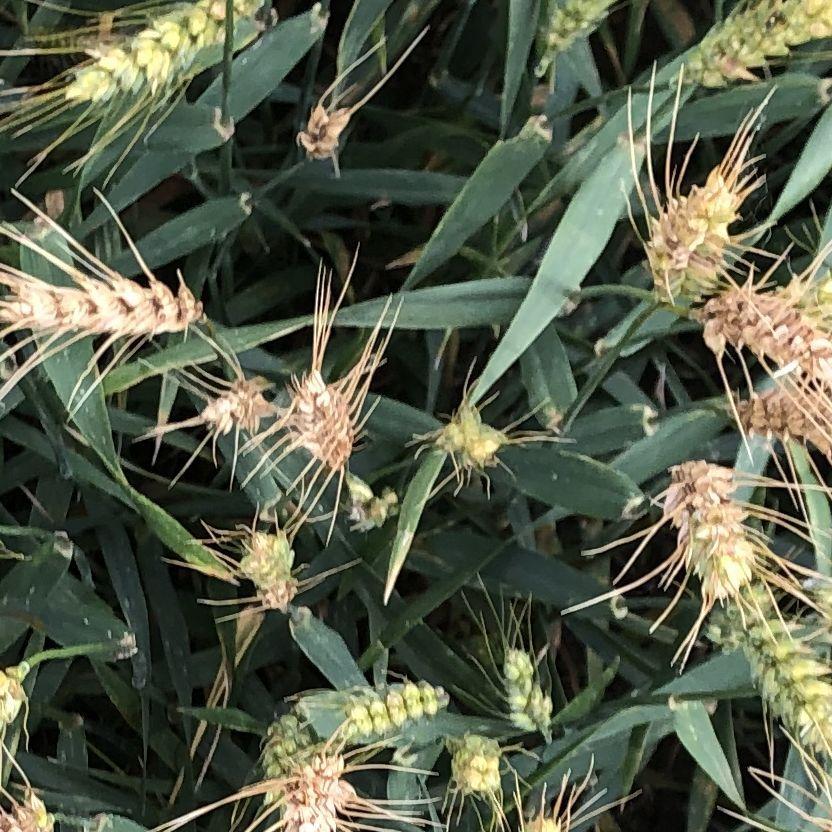

Supplement: Supplemental Information 2 [file peerj-cs-10-1948-s002.zip › data1/image0051.jpg]

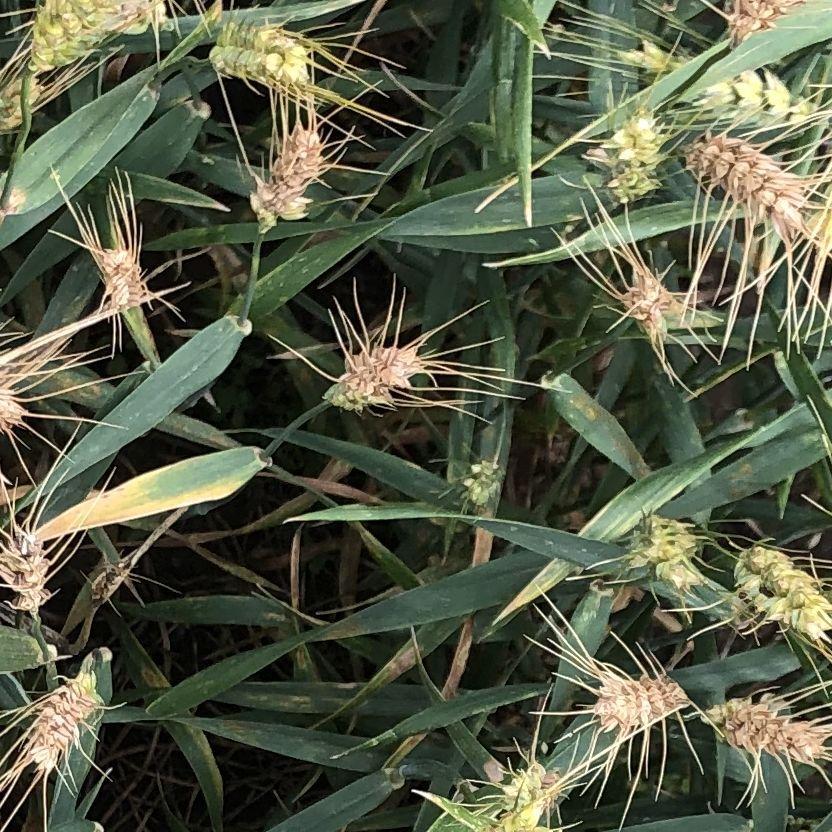

Supplement: Supplemental Information 2 [file peerj-cs-10-1948-s002.zip › data1/image0052.jpg]

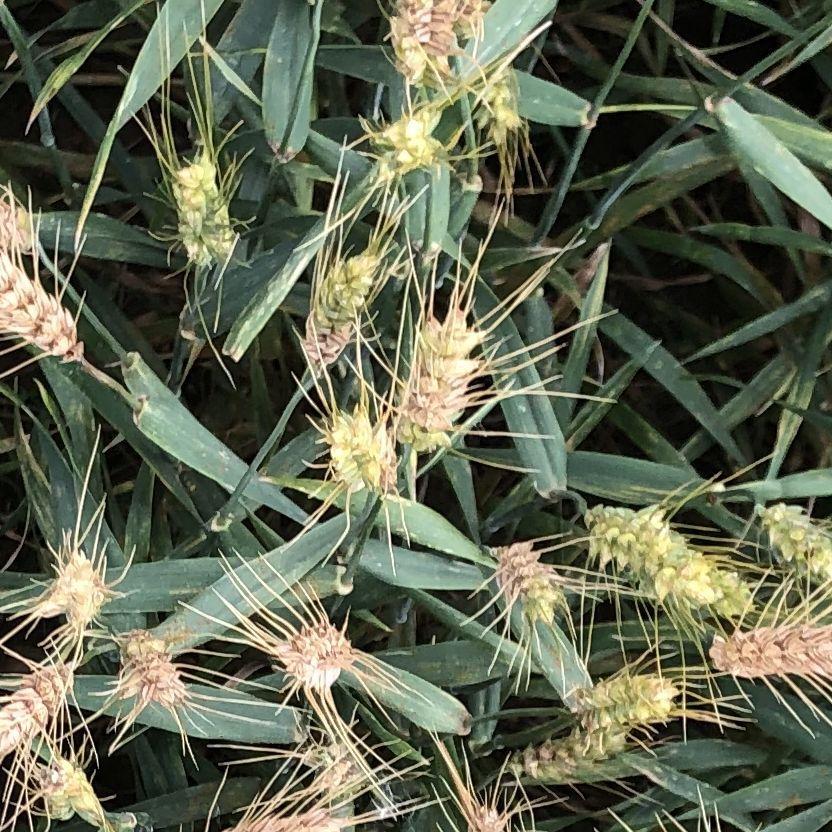

Supplement: Supplemental Information 2 [file peerj-cs-10-1948-s002.zip › data1/image0056.jpg]

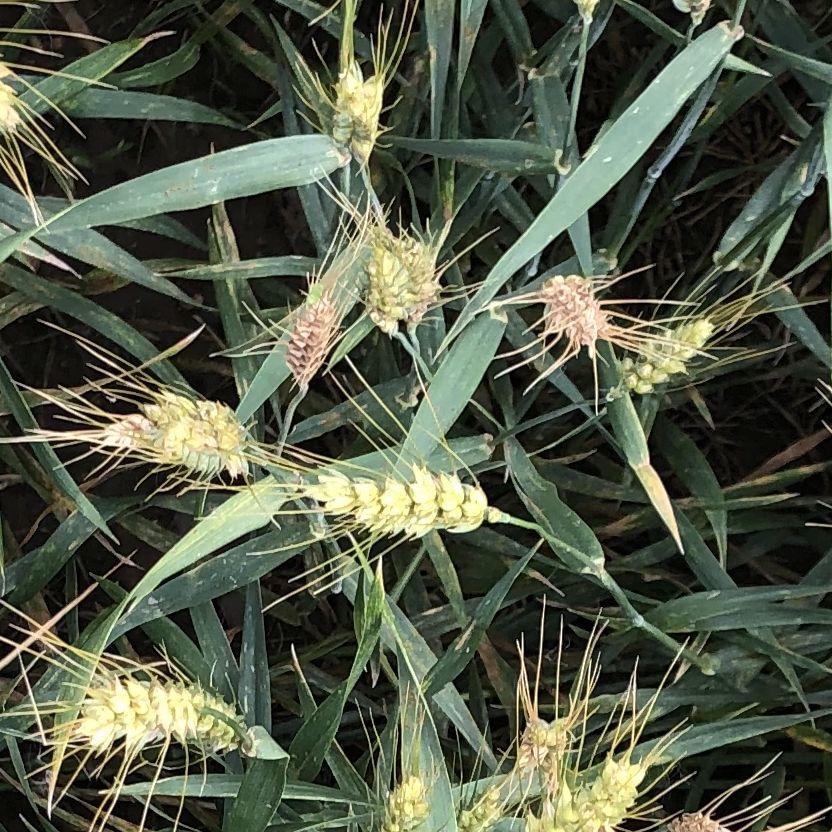

Supplement: Supplemental Information 2 [file peerj-cs-10-1948-s002.zip › data1/image0057.jpg]

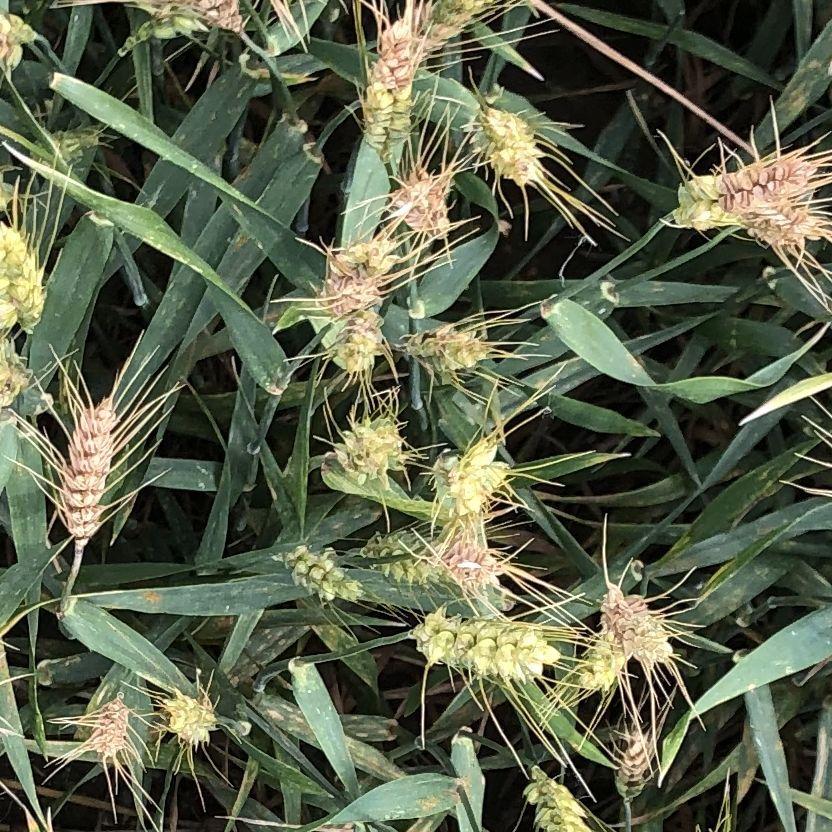

Supplement: Supplemental Information 2 [file peerj-cs-10-1948-s002.zip › data1/image0058.jpg]

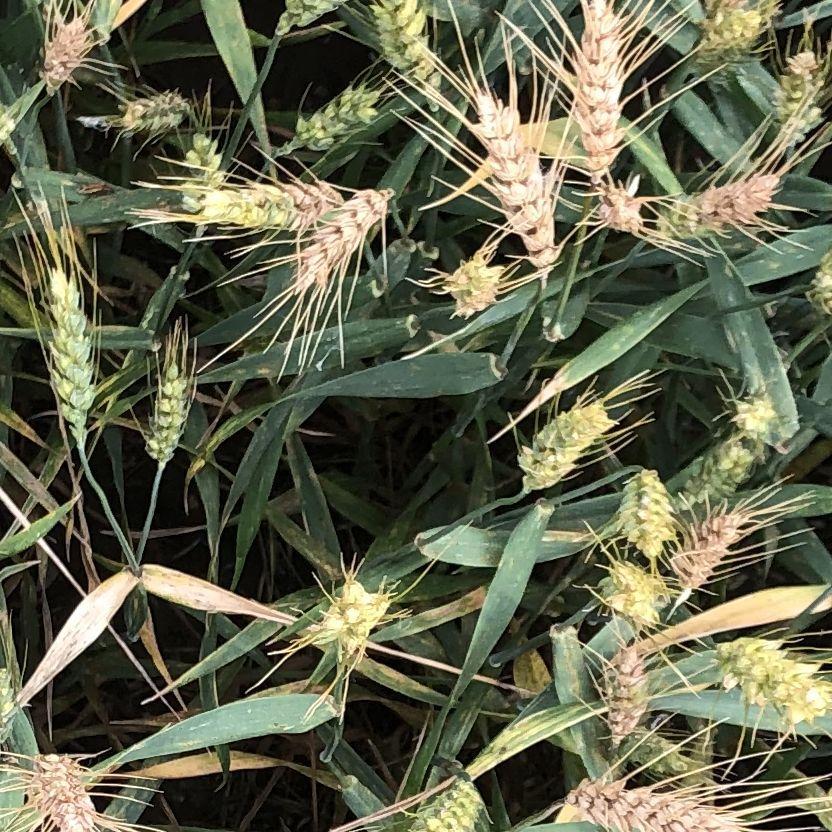

Supplement: Supplemental Information 2 [file peerj-cs-10-1948-s002.zip › data1/image0059.jpg]

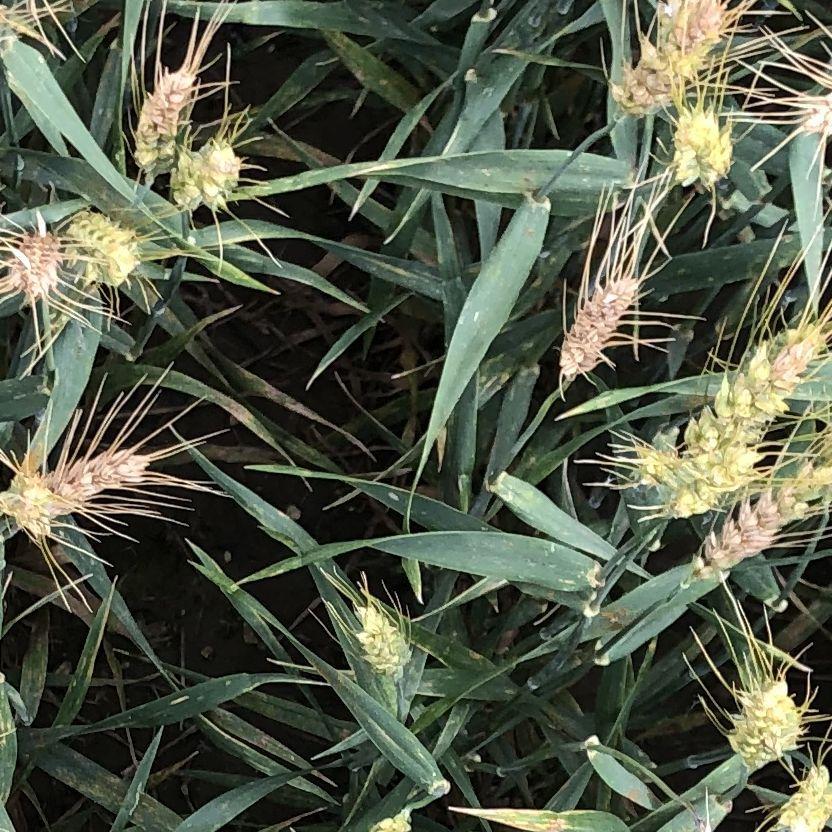

Supplement: Supplemental Information 2 [file peerj-cs-10-1948-s002.zip › data1/image0060.jpg]

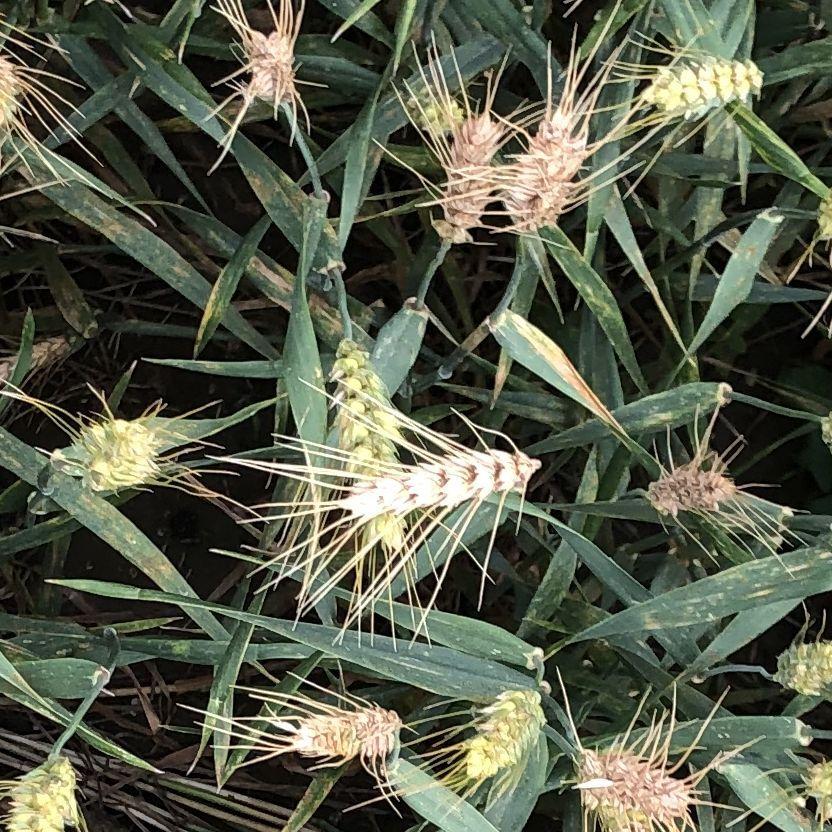

Supplement: Supplemental Information 2 [file peerj-cs-10-1948-s002.zip › data1/image0061.jpg]

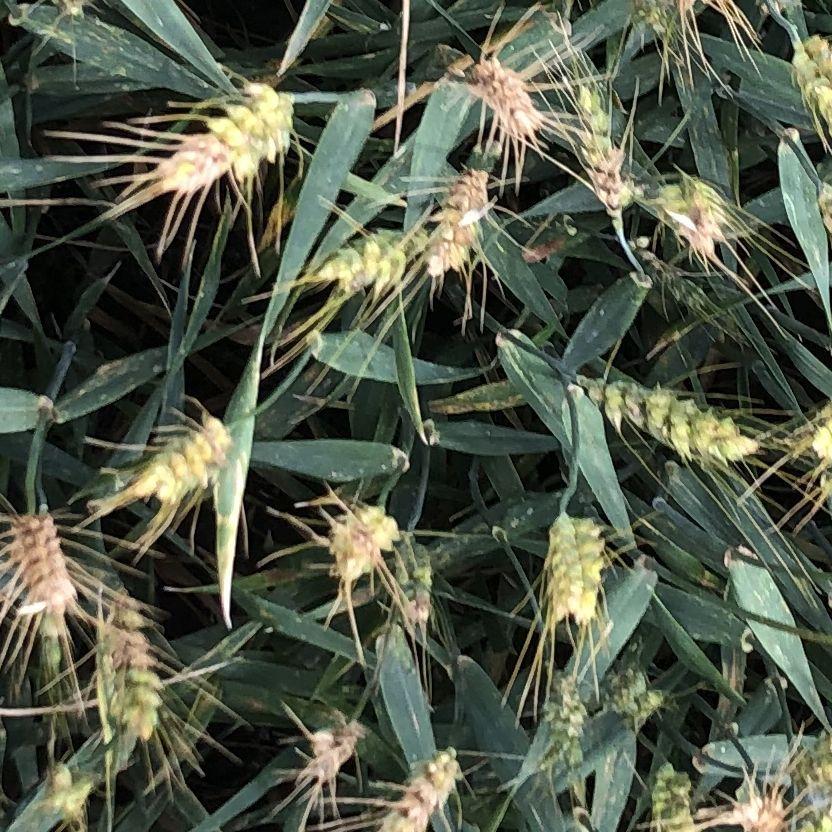

Supplement: Supplemental Information 2 [file peerj-cs-10-1948-s002.zip › data1/image0062.jpg]

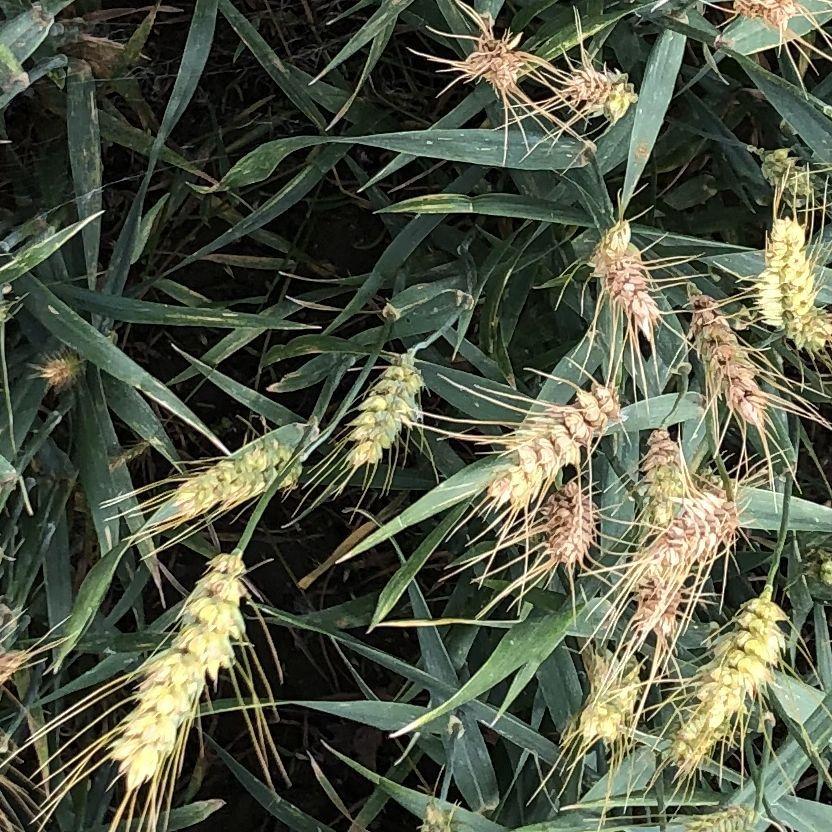

Supplement: Supplemental Information 2 [file peerj-cs-10-1948-s002.zip › data1/image0065.jpg]

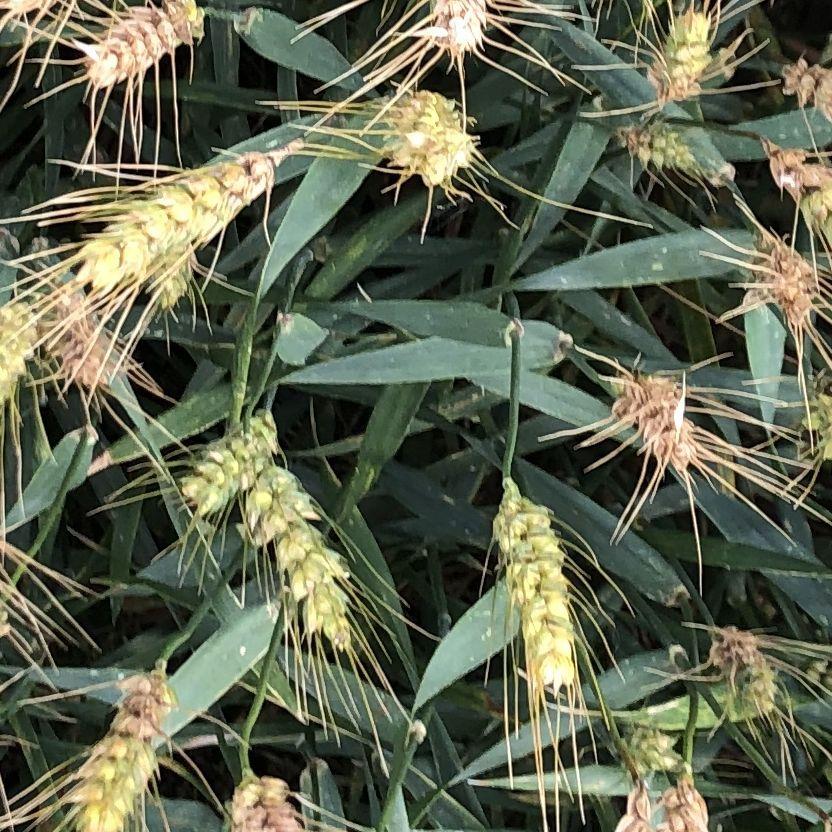

Supplement: Supplemental Information 2 [file peerj-cs-10-1948-s002.zip › data1/image0066.jpg]

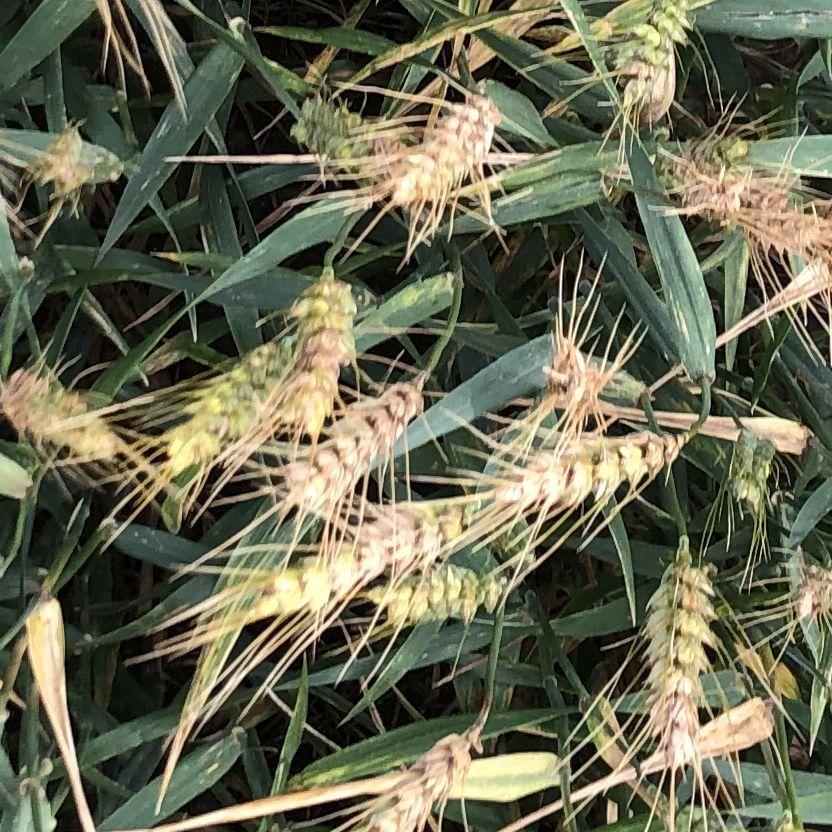

Supplement: Supplemental Information 2 [file peerj-cs-10-1948-s002.zip › data1/image0067.jpg]

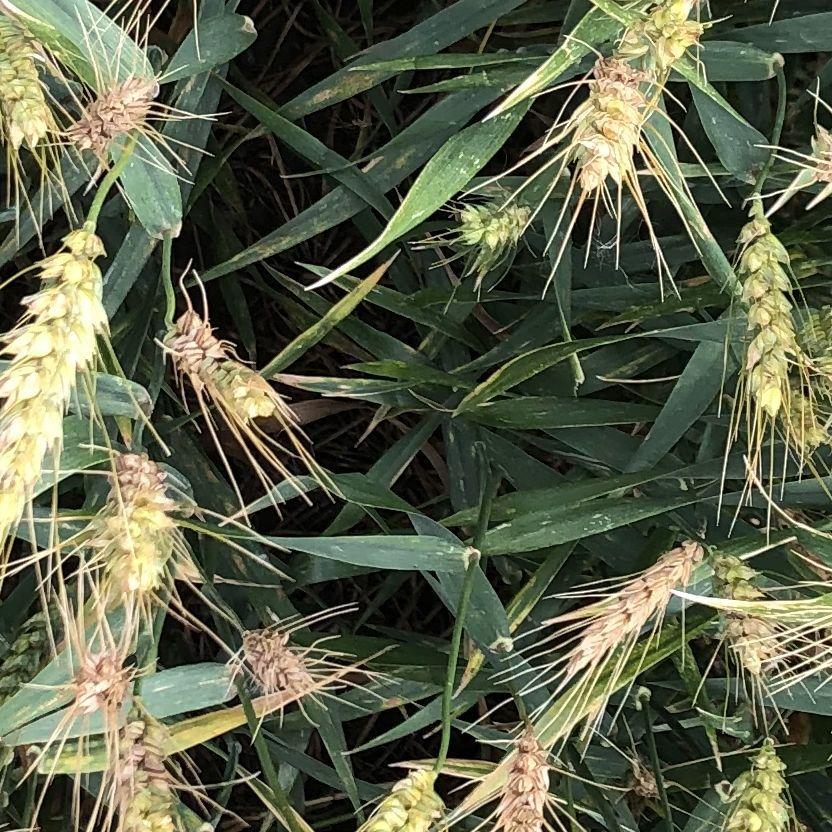

Supplement: Supplemental Information 2 [file peerj-cs-10-1948-s002.zip › data1/image0068.jpg]

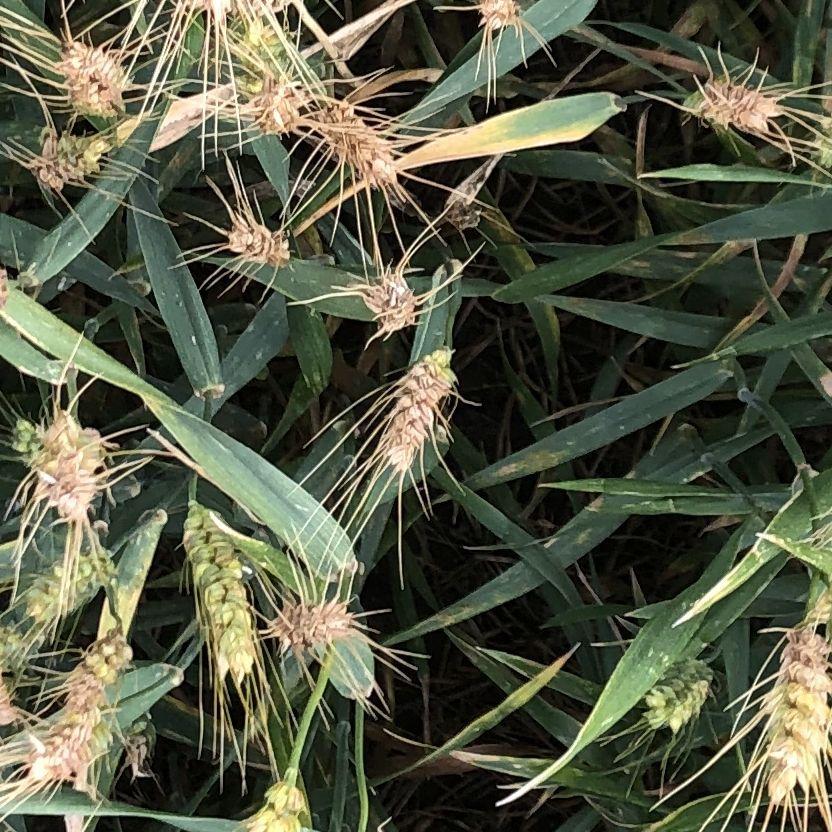

Supplement: Supplemental Information 2 [file peerj-cs-10-1948-s002.zip › data1/image0069.jpg]

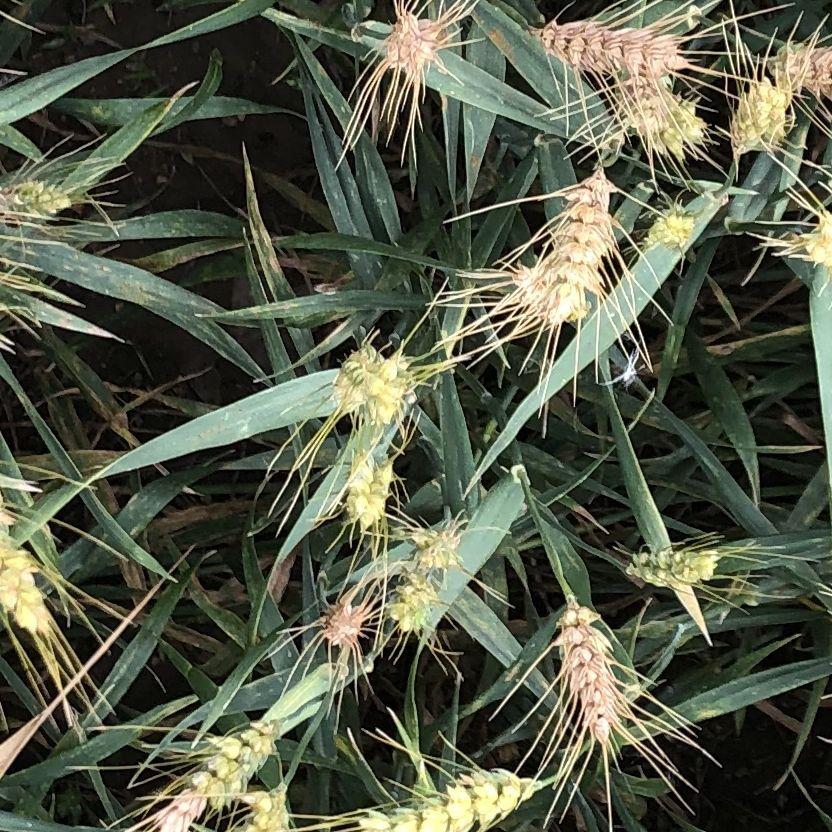

Supplement: Supplemental Information 2 [file peerj-cs-10-1948-s002.zip › data1/image0071.jpg]

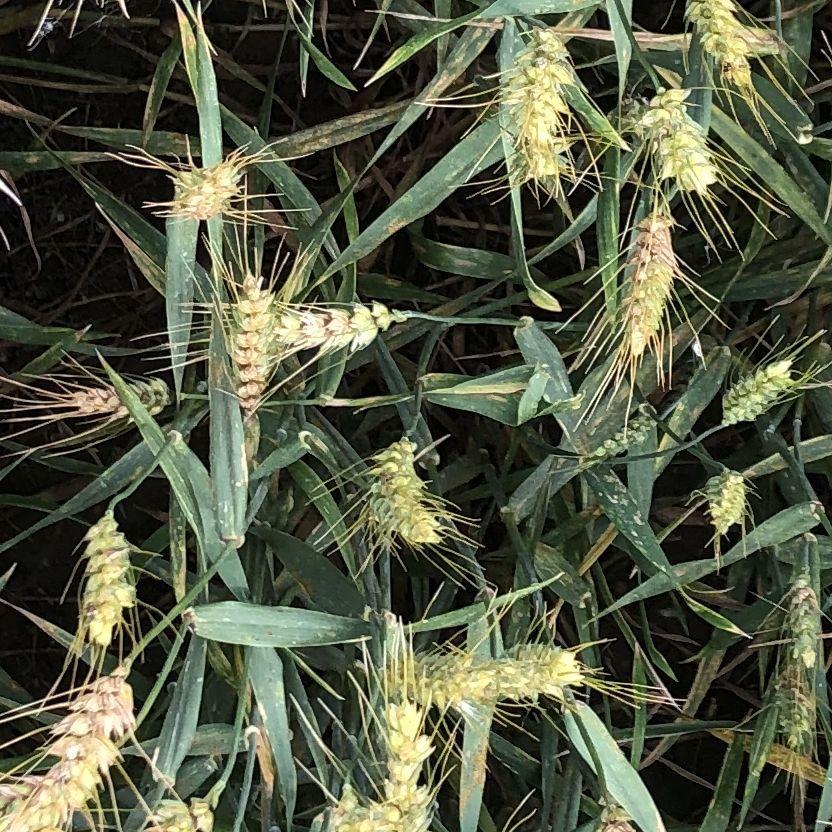

Supplement: Supplemental Information 2 [file peerj-cs-10-1948-s002.zip › data1/image0072.jpg]

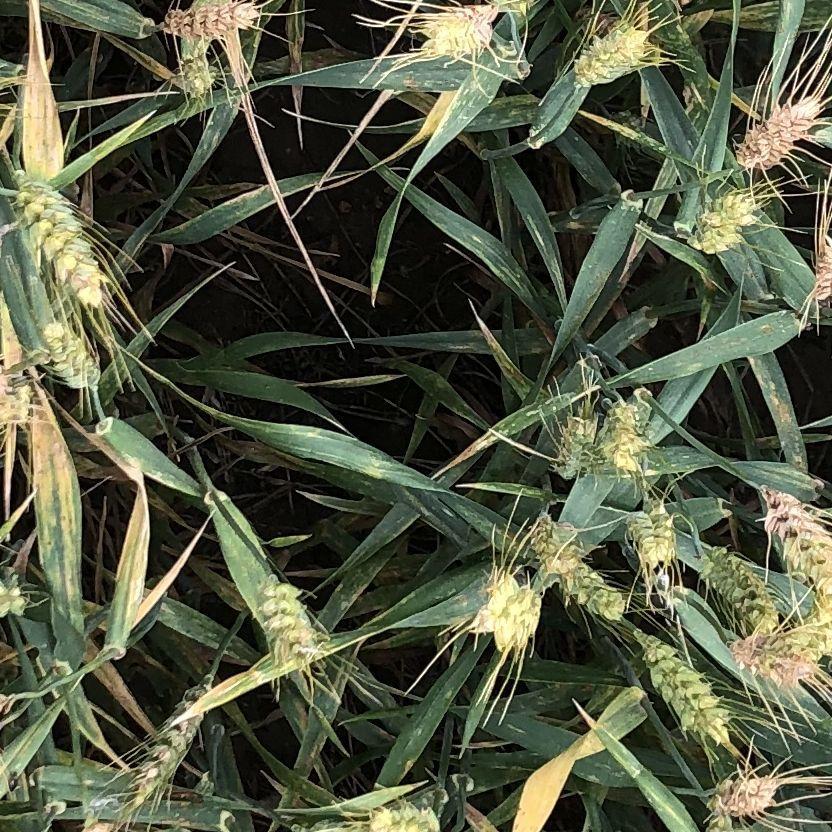

Supplement: Supplemental Information 2 [file peerj-cs-10-1948-s002.zip › data1/image0073.jpg]

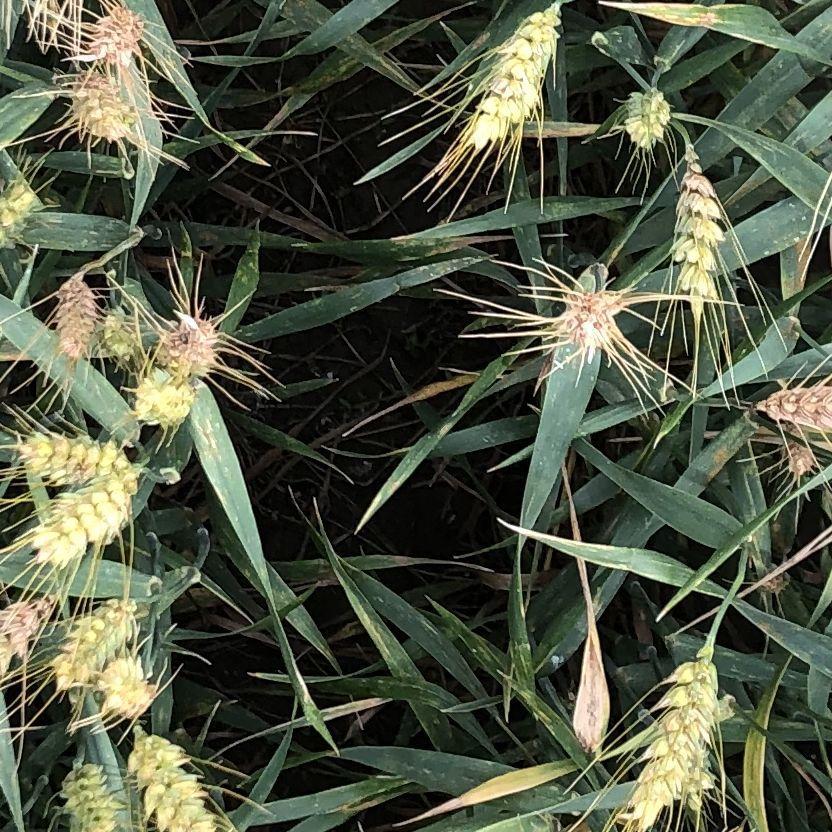

Supplement: Supplemental Information 2 [file peerj-cs-10-1948-s002.zip › data1/image0075.jpg]

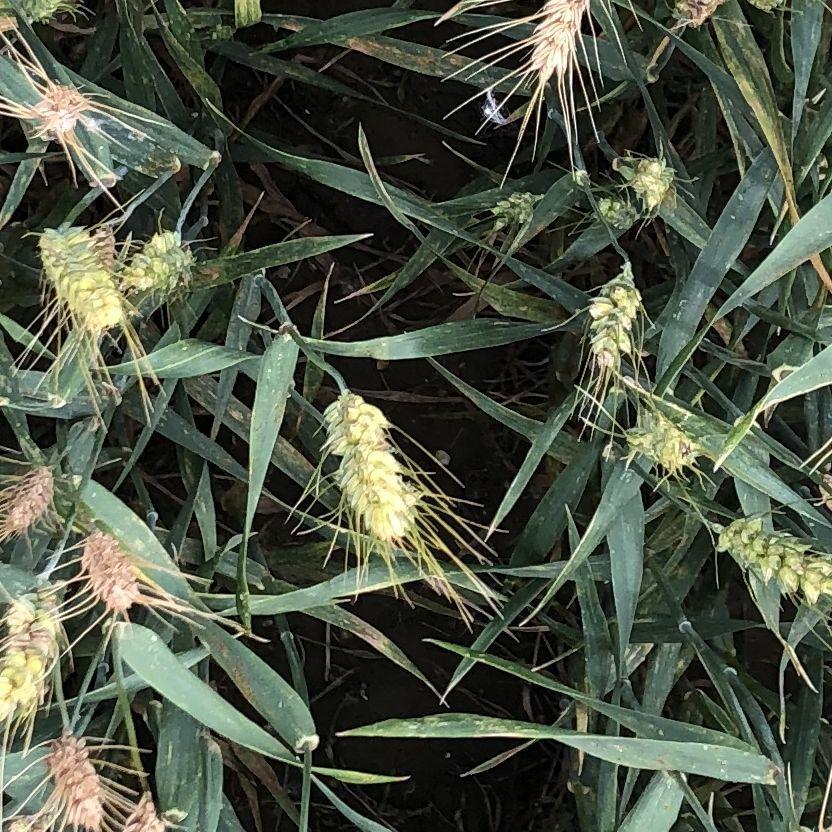

Supplement: Supplemental Information 2 [file peerj-cs-10-1948-s002.zip › data1/image0076.jpg]

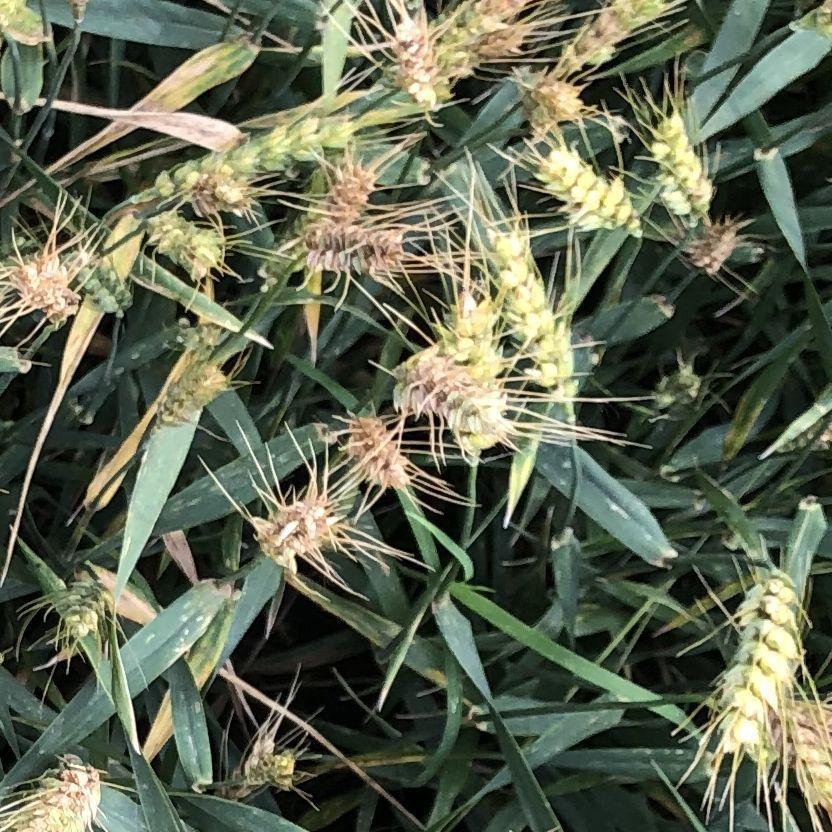

Supplement: Supplemental Information 2 [file peerj-cs-10-1948-s002.zip › data1/image0077.jpg]

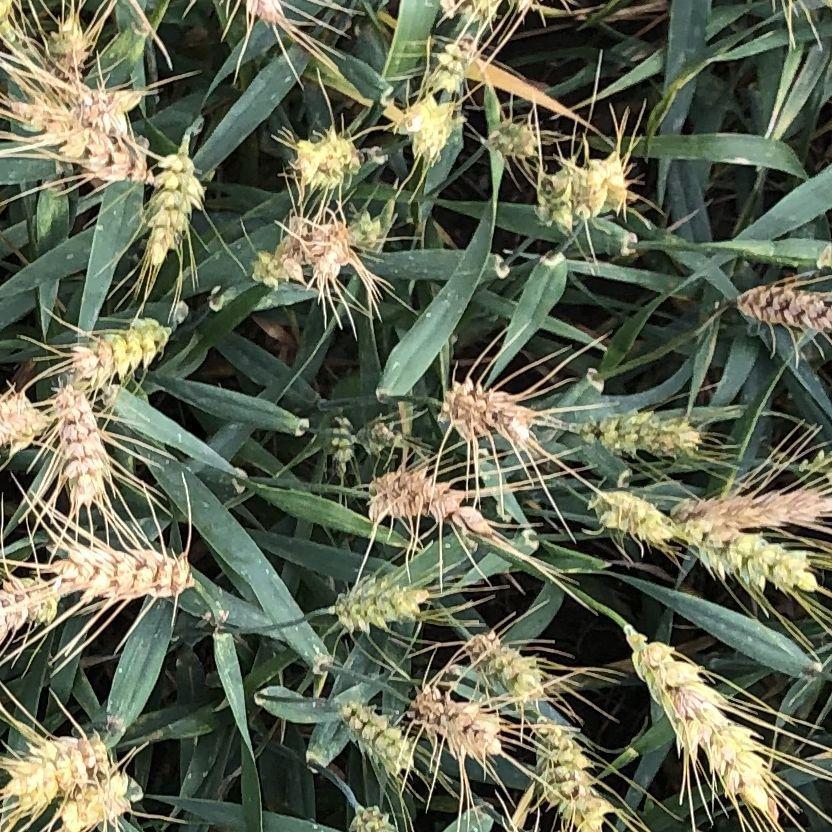

Supplement: Supplemental Information 2 [file peerj-cs-10-1948-s002.zip › data1/image0080.jpg]

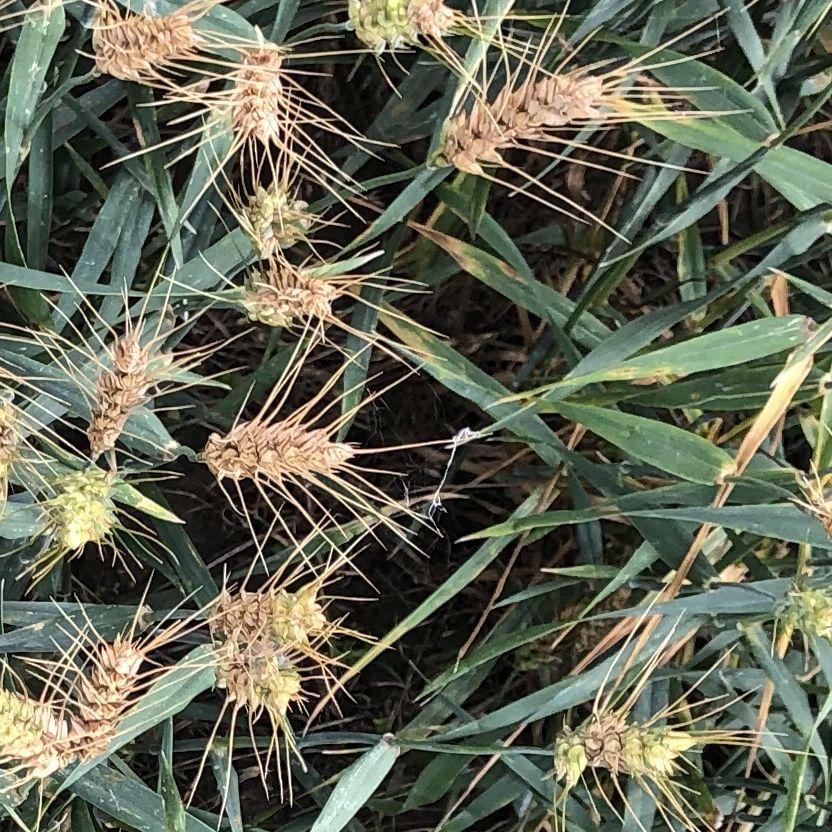

Supplement: Supplemental Information 2 [file peerj-cs-10-1948-s002.zip › data1/image0081.jpg]

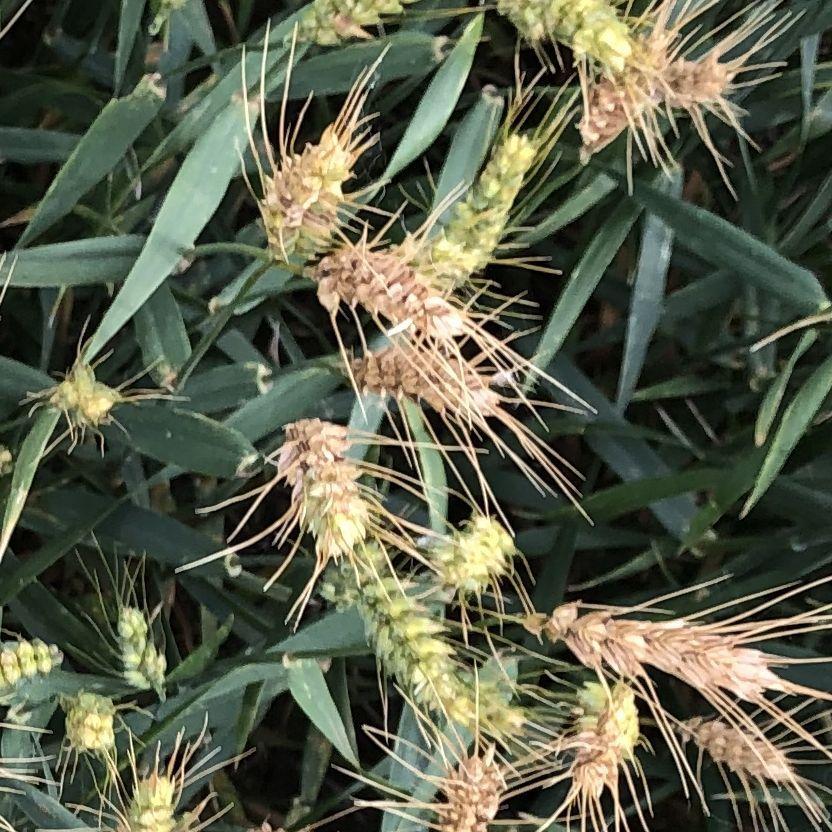

Supplement: Supplemental Information 2 [file peerj-cs-10-1948-s002.zip › data1/image0082.jpg]

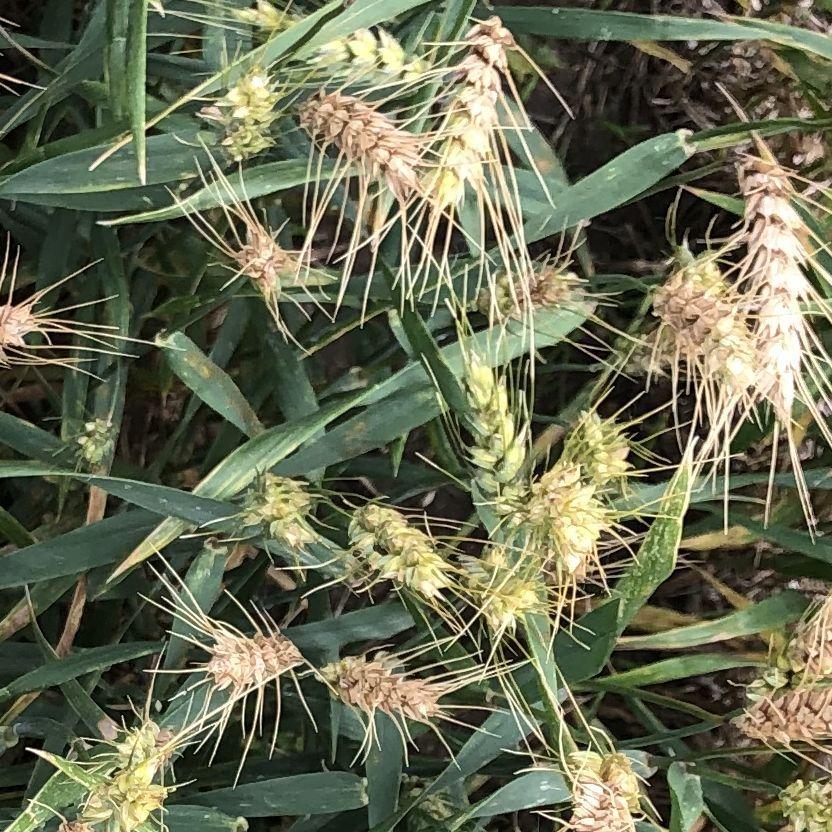

Supplement: Supplemental Information 2 [file peerj-cs-10-1948-s002.zip › data1/image0083.jpg]

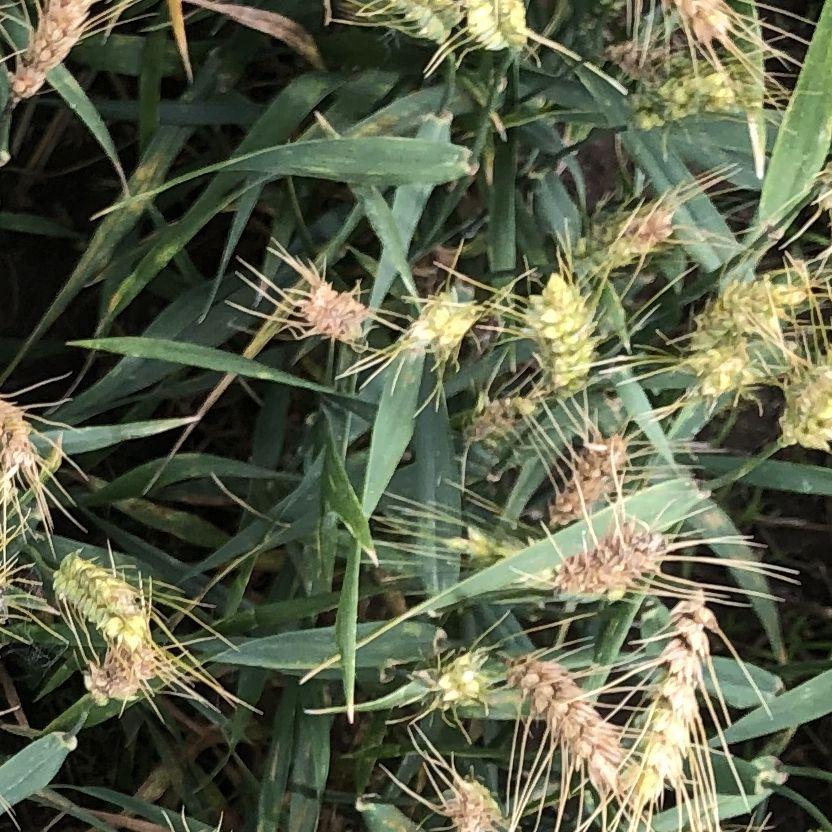

Supplement: Supplemental Information 2 [file peerj-cs-10-1948-s002.zip › data1/image0084.jpg]

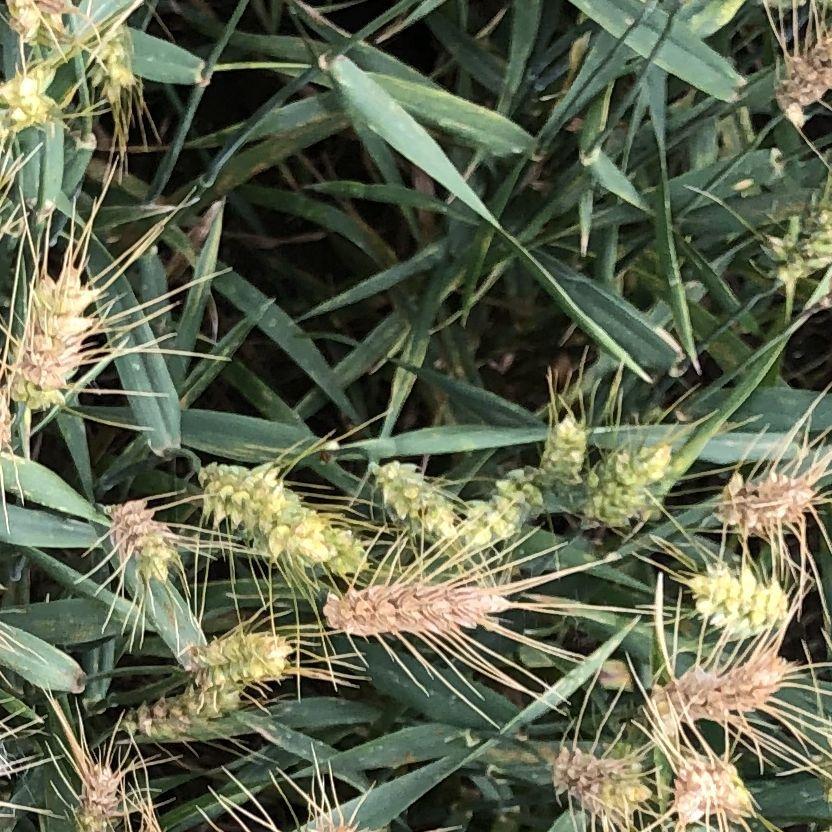

Supplement: Supplemental Information 2 [file peerj-cs-10-1948-s002.zip › data1/image0085.jpg]

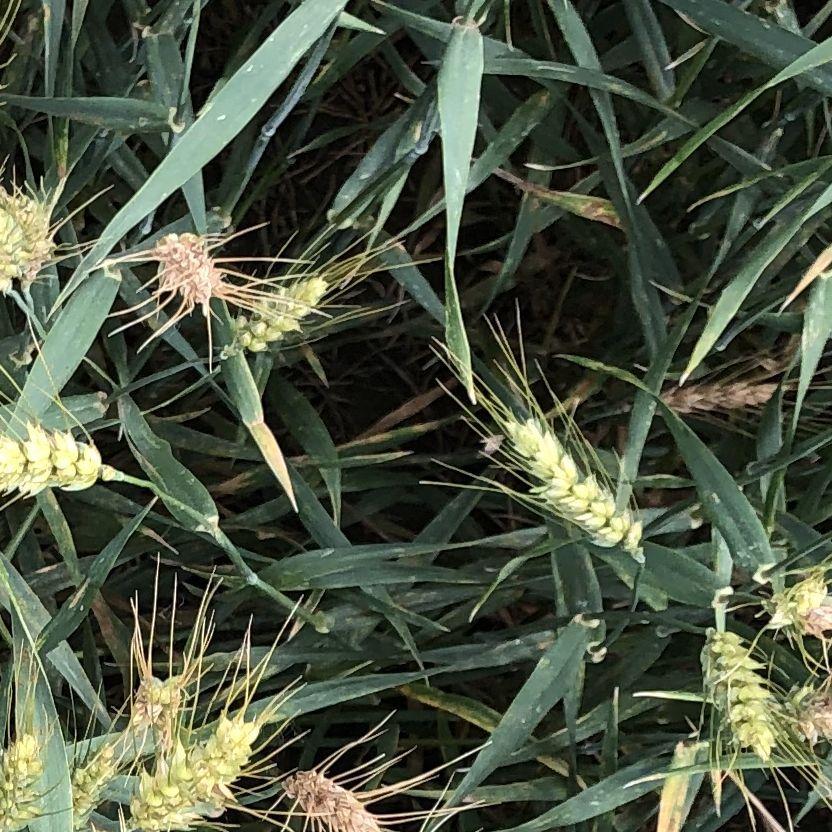

Supplement: Supplemental Information 2 [file peerj-cs-10-1948-s002.zip › data1/image0086.jpg]

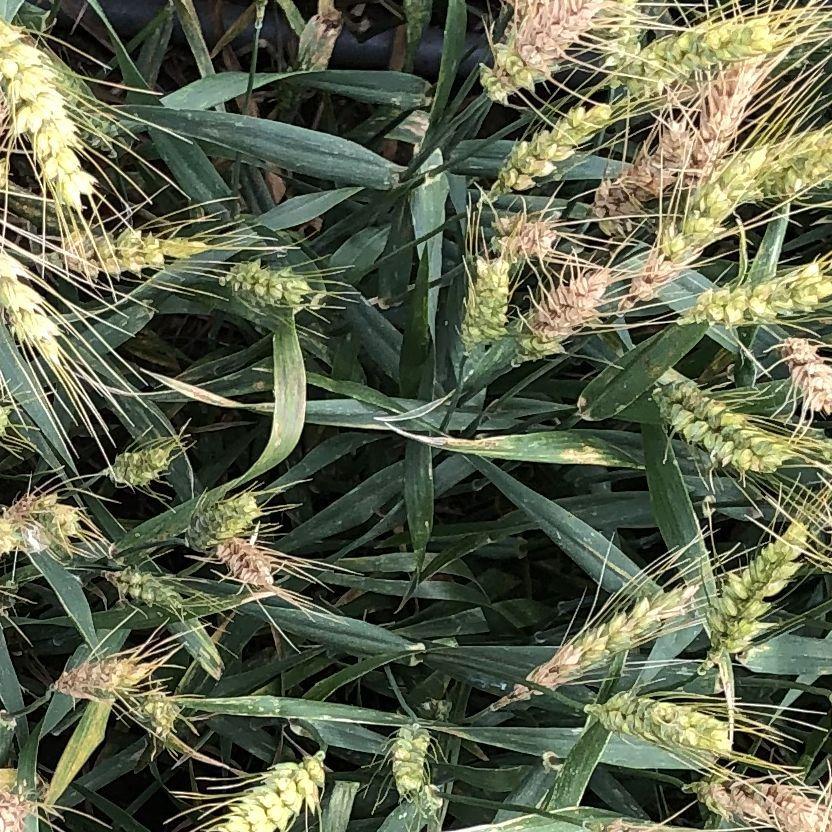

Supplement: Supplemental Information 2 [file peerj-cs-10-1948-s002.zip › data1/image0087.jpg]

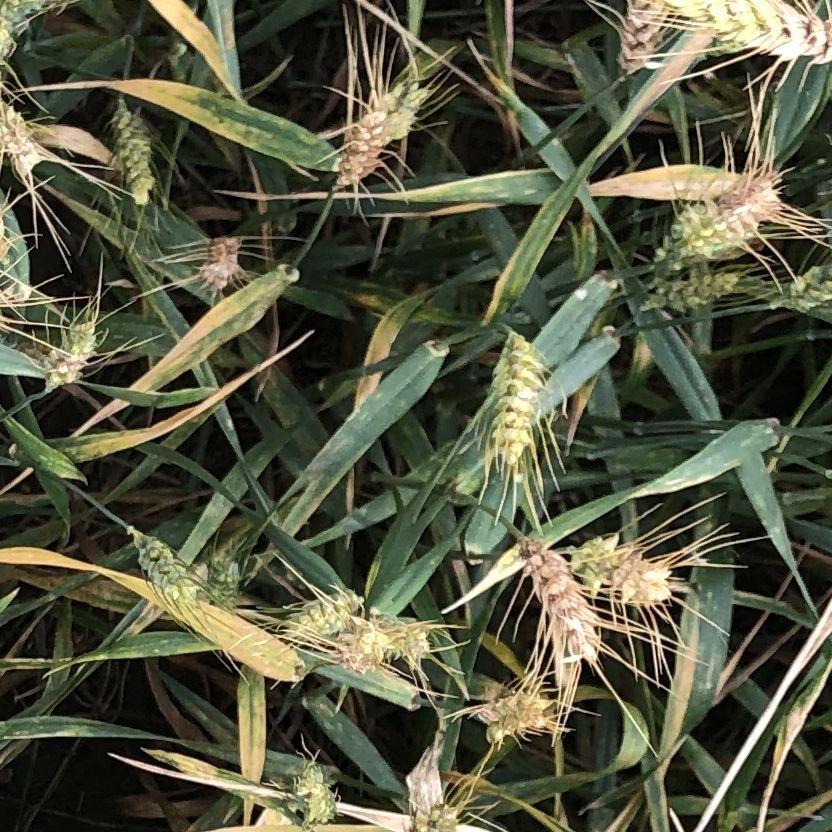

Supplement: Supplemental Information 2 [file peerj-cs-10-1948-s002.zip › data1/image0088.jpg]

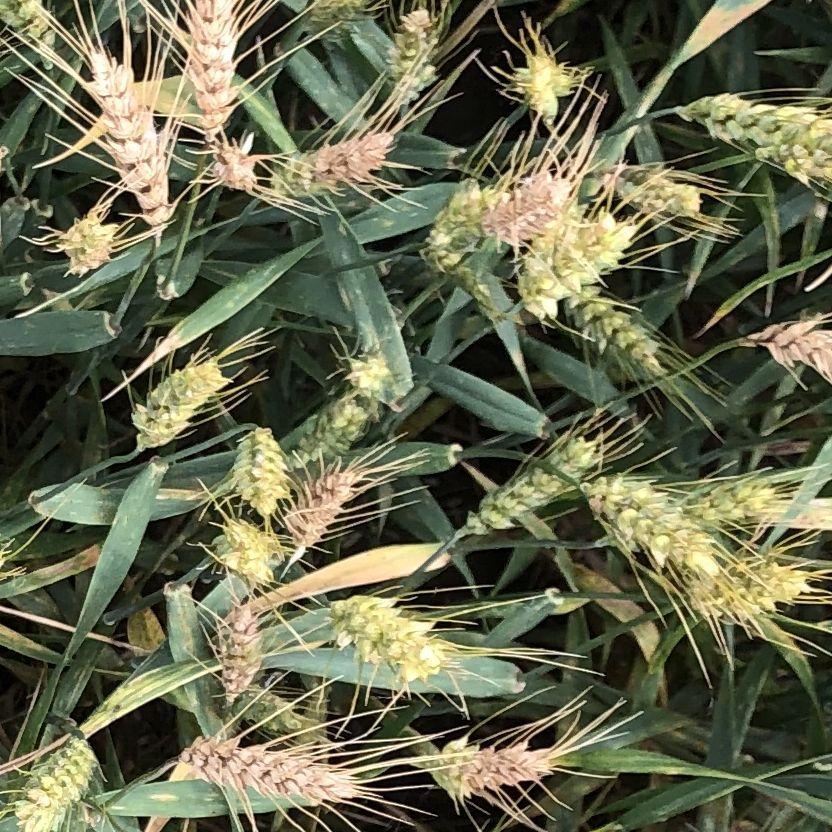

Supplement: Supplemental Information 2 [file peerj-cs-10-1948-s002.zip › data1/image0089.jpg]

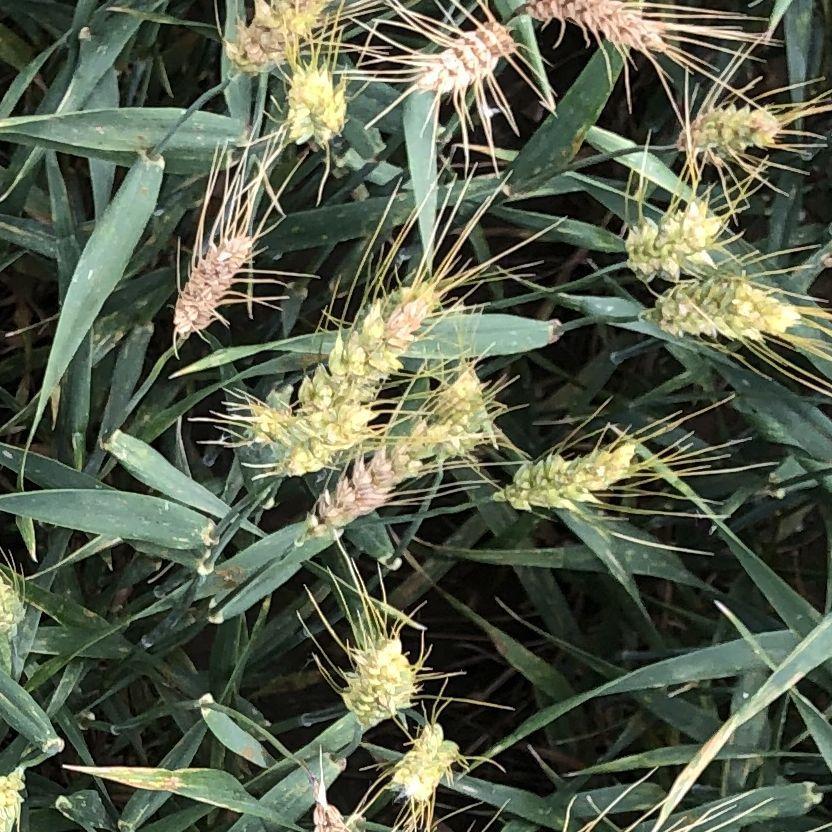

Supplement: Supplemental Information 2 [file peerj-cs-10-1948-s002.zip › data1/image0090.jpg]

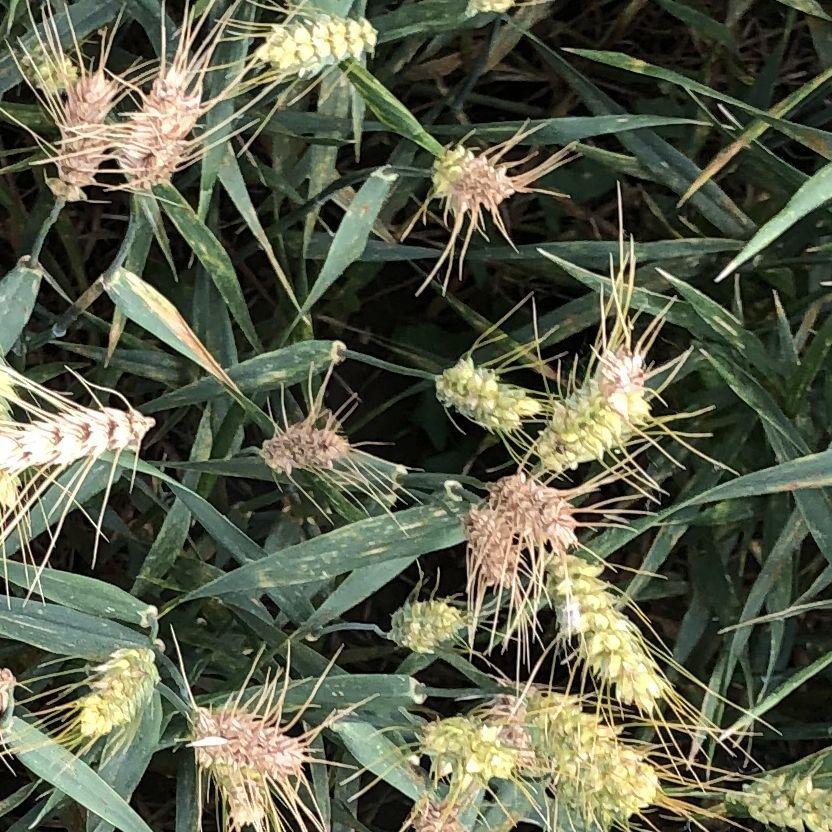

Supplement: Supplemental Information 2 [file peerj-cs-10-1948-s002.zip › data1/image0091.jpg]

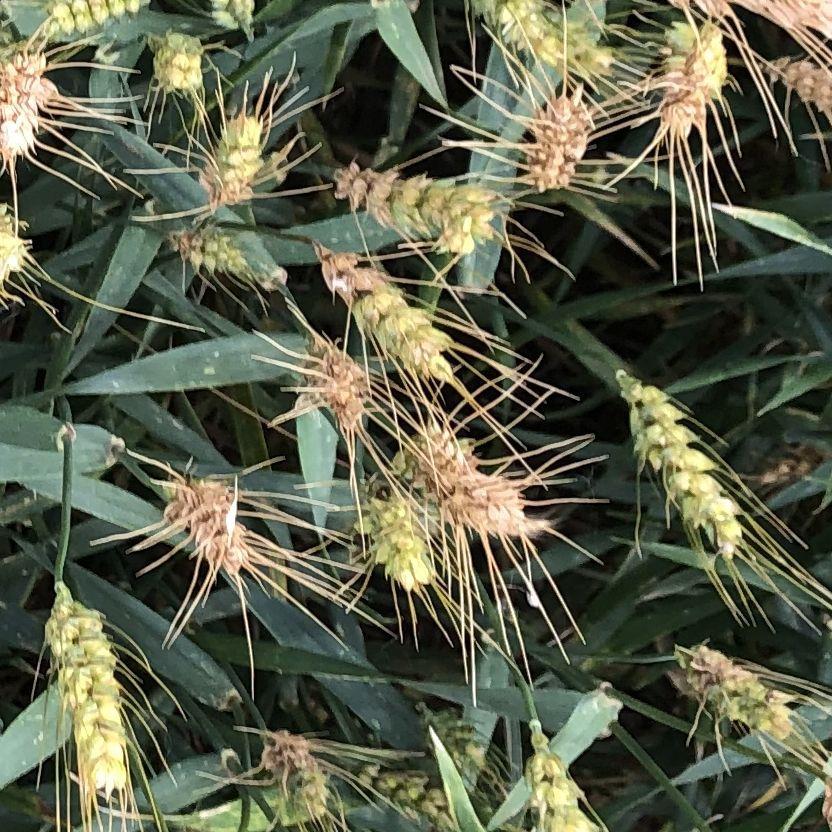

Supplement: Supplemental Information 2 [file peerj-cs-10-1948-s002.zip › data1/image0092.jpg]

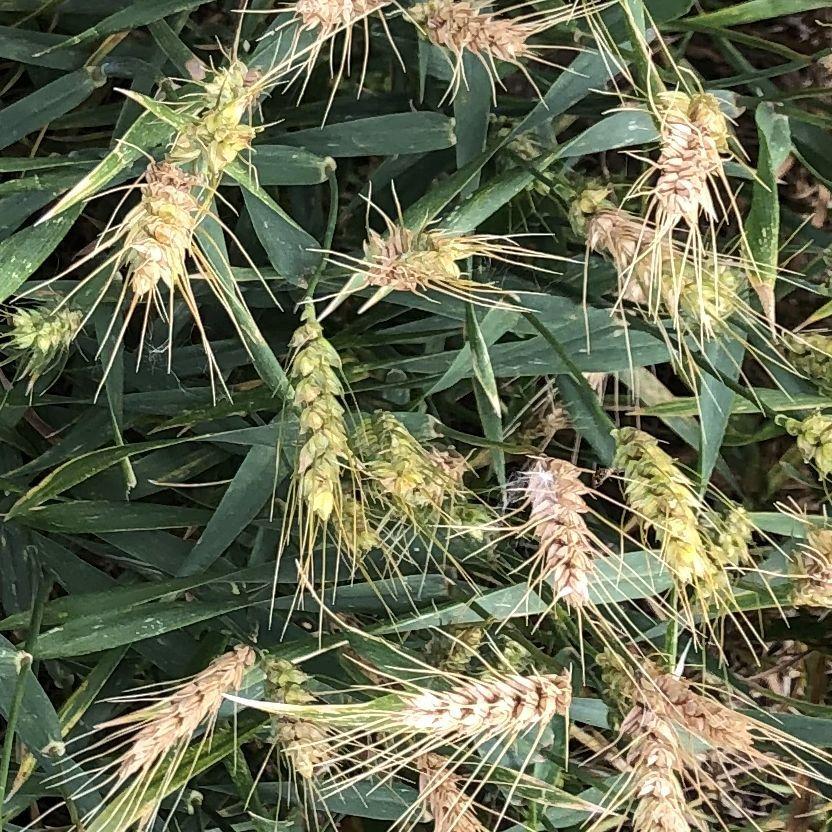

Supplement: Supplemental Information 2 [file peerj-cs-10-1948-s002.zip › data1/image0095.jpg]

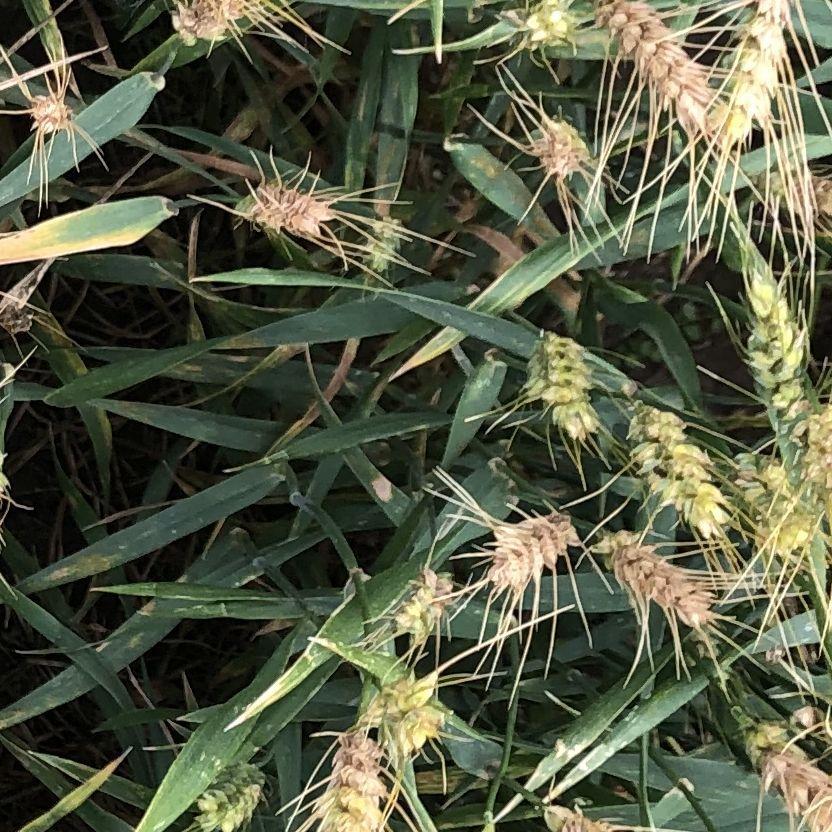

Supplement: Supplemental Information 2 [file peerj-cs-10-1948-s002.zip › data1/image0096.jpg]

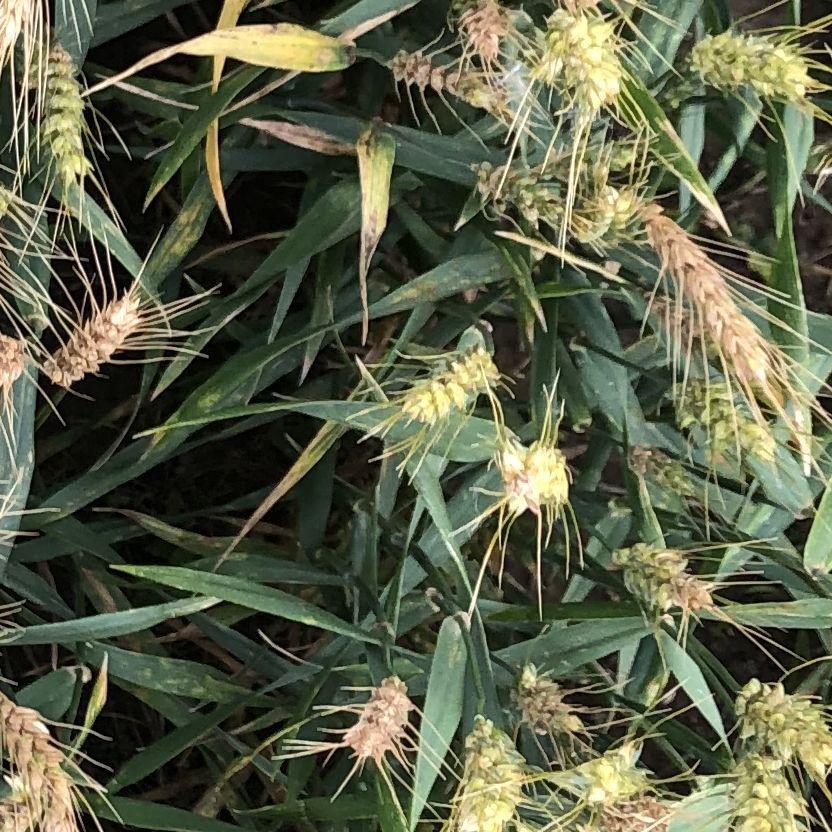

Supplement: Supplemental Information 2 [file peerj-cs-10-1948-s002.zip › data1/image0097.jpg]

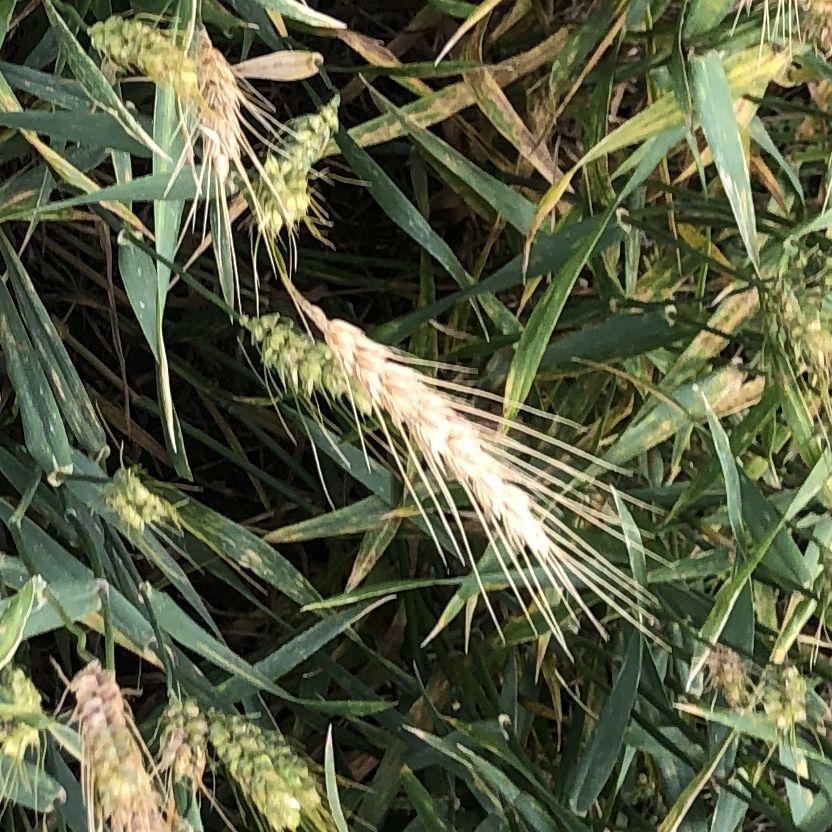

Supplement: Supplemental Information 2 [file peerj-cs-10-1948-s002.zip › data1/image0098.jpg]

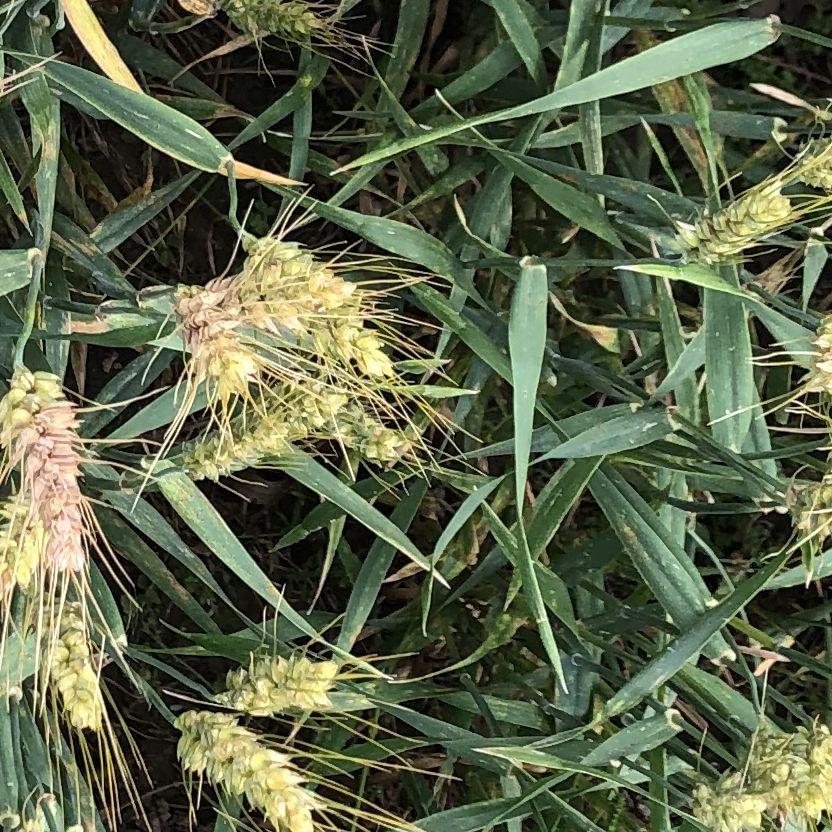

Supplement: Supplemental Information 2 [file peerj-cs-10-1948-s002.zip › data1/image0099.jpg]

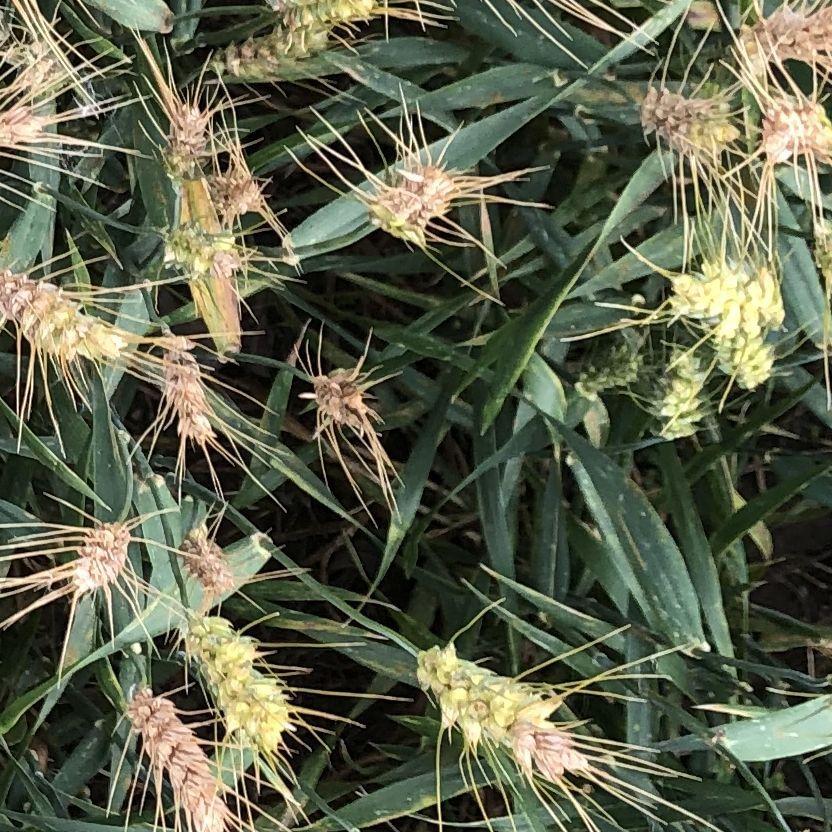

Supplement: Supplemental Information 2 [file peerj-cs-10-1948-s002.zip › data1/image0100.jpg]

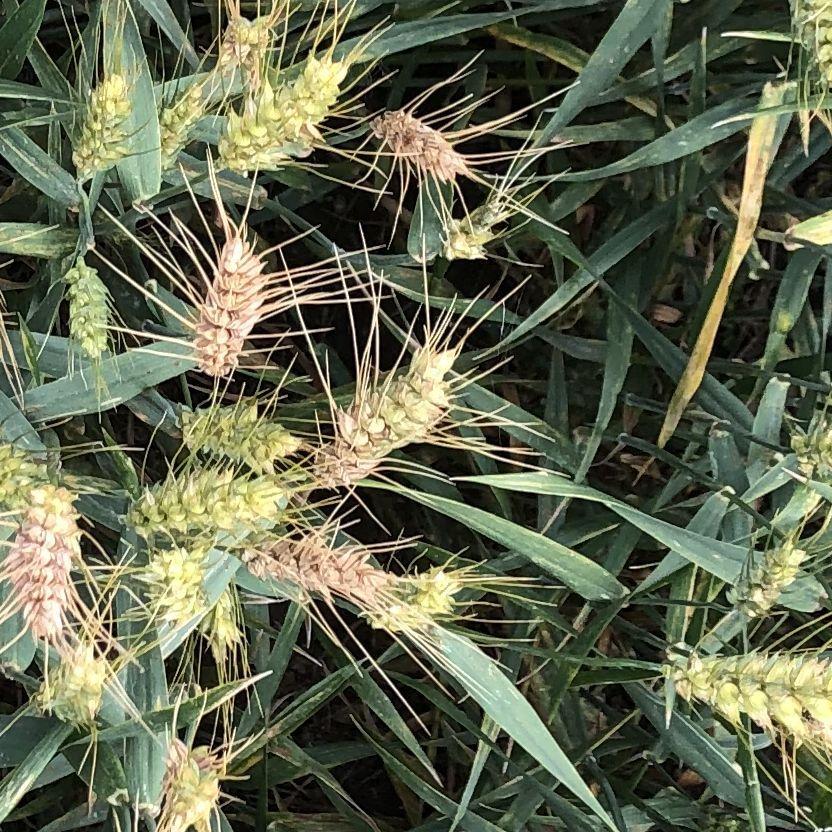

Supplement: Supplemental Information 2 [file peerj-cs-10-1948-s002.zip › data1/image0101.jpg]

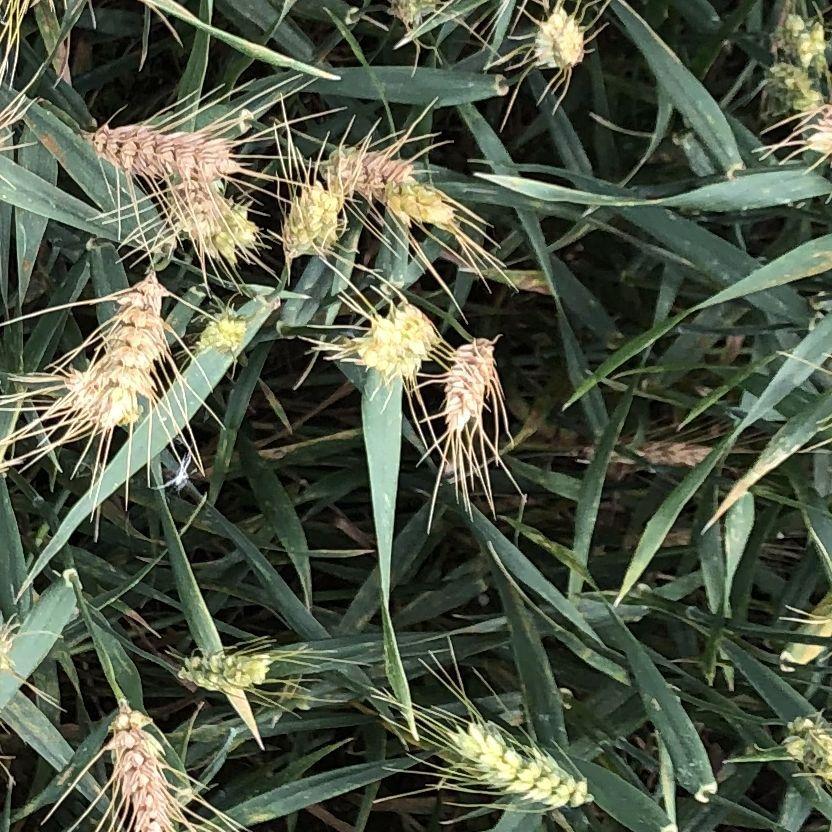

Supplement: Supplemental Information 2 [file peerj-cs-10-1948-s002.zip › data1/image0102.jpg]

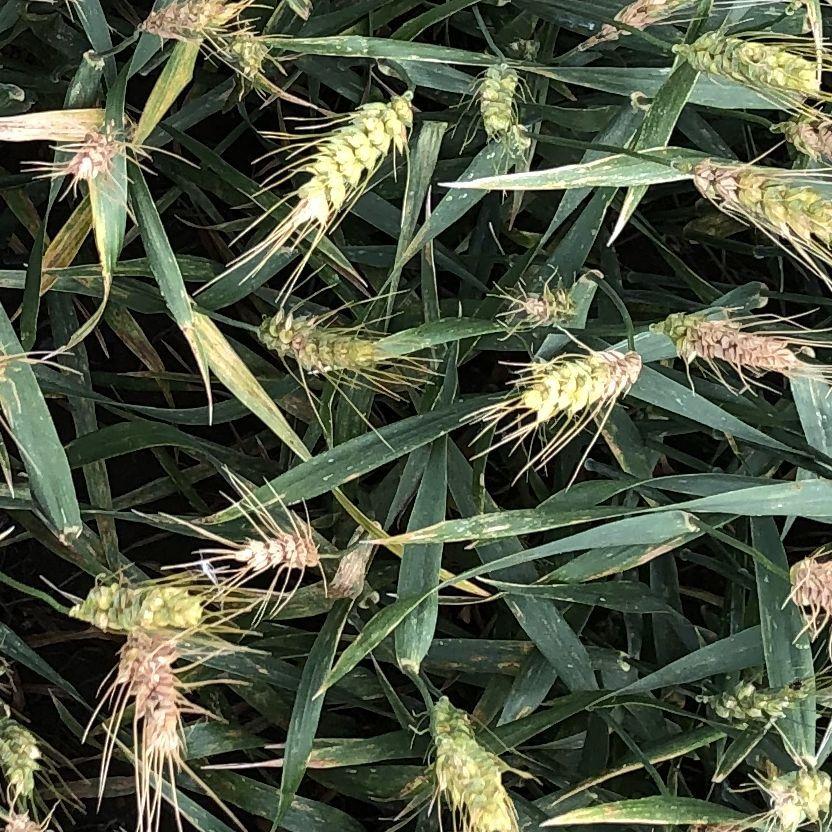

Supplement: Supplemental Information 2 [file peerj-cs-10-1948-s002.zip › data1/image0103.jpg]

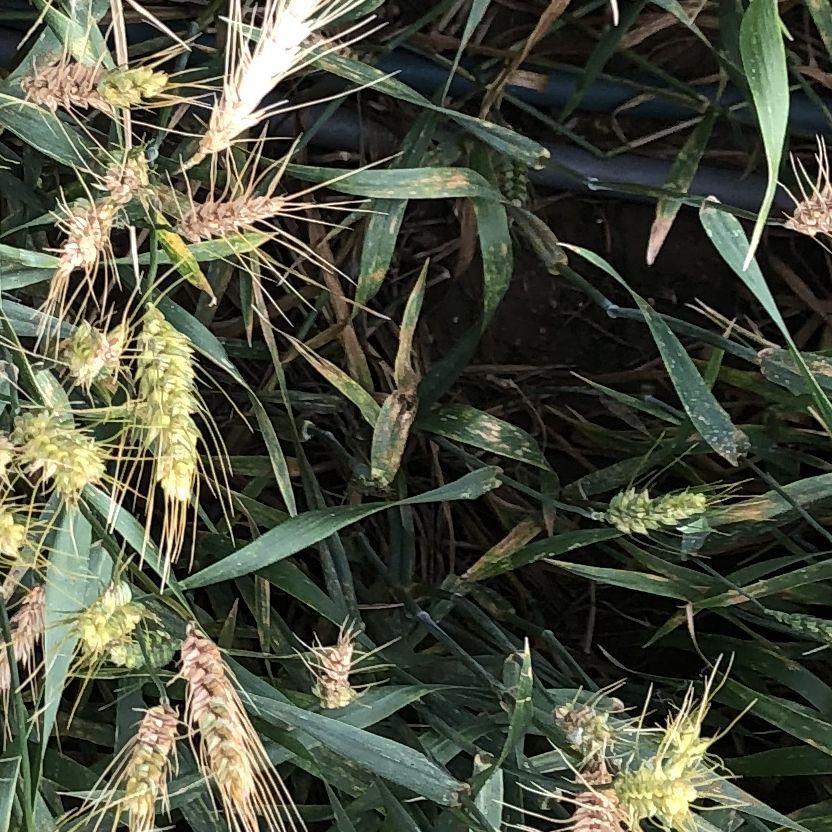

Supplement: Supplemental Information 2 [file peerj-cs-10-1948-s002.zip › data1/image0104.jpg]

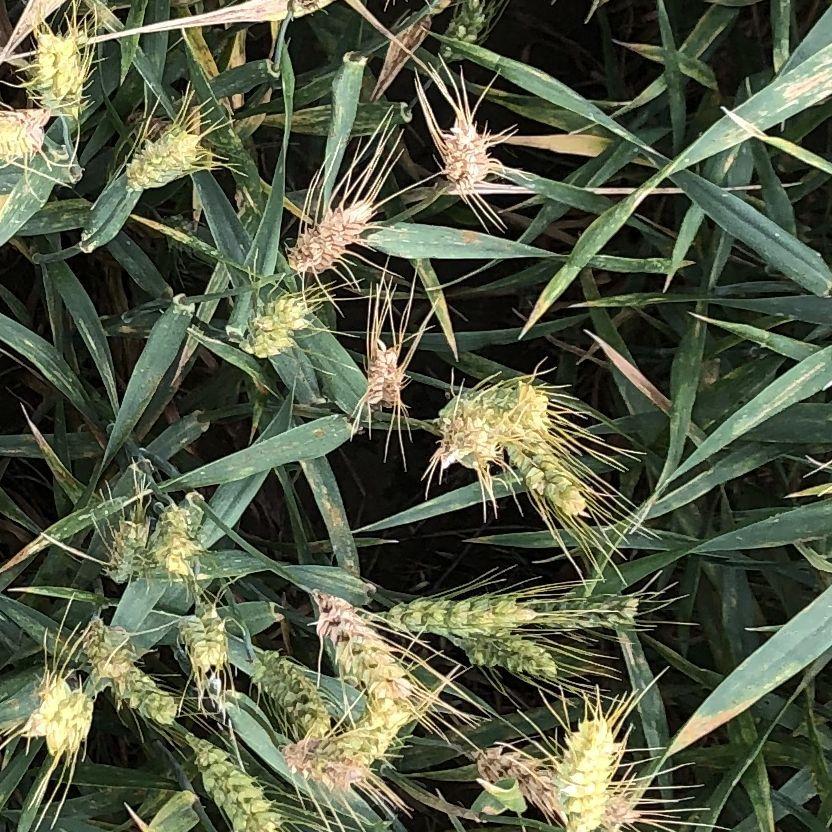

Supplement: Supplemental Information 2 [file peerj-cs-10-1948-s002.zip › data1/image0105.jpg]

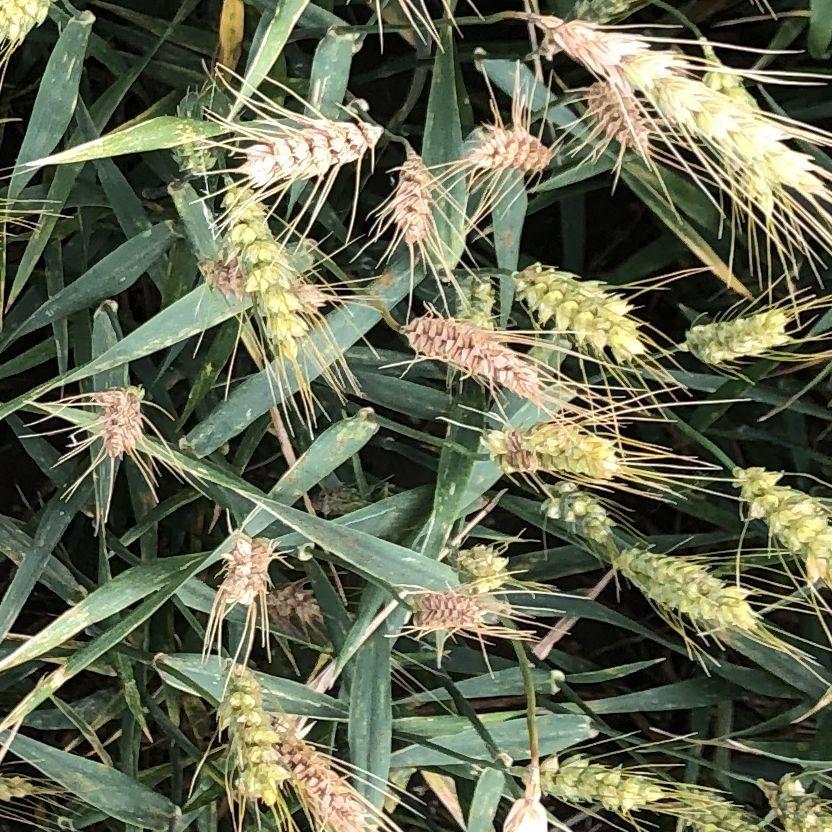

Supplement: Supplemental Information 2 [file peerj-cs-10-1948-s002.zip › data1/image0106.jpg]

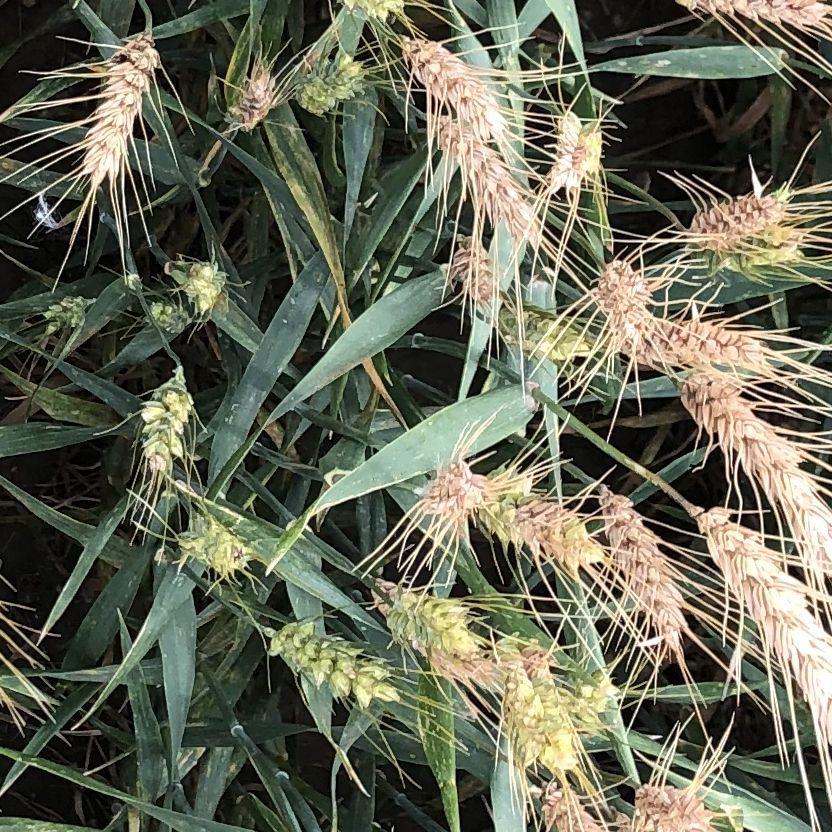

Supplement: Supplemental Information 2 [file peerj-cs-10-1948-s002.zip › data1/image0107.jpg]

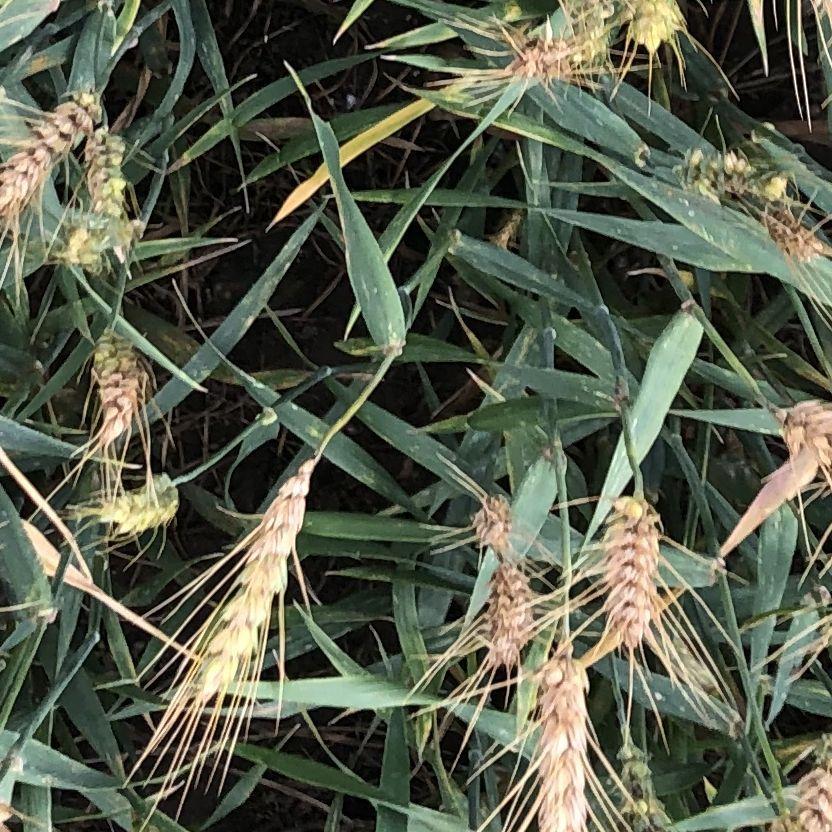

Supplement: Supplemental Information 2 [file peerj-cs-10-1948-s002.zip › data1/image0110.jpg]

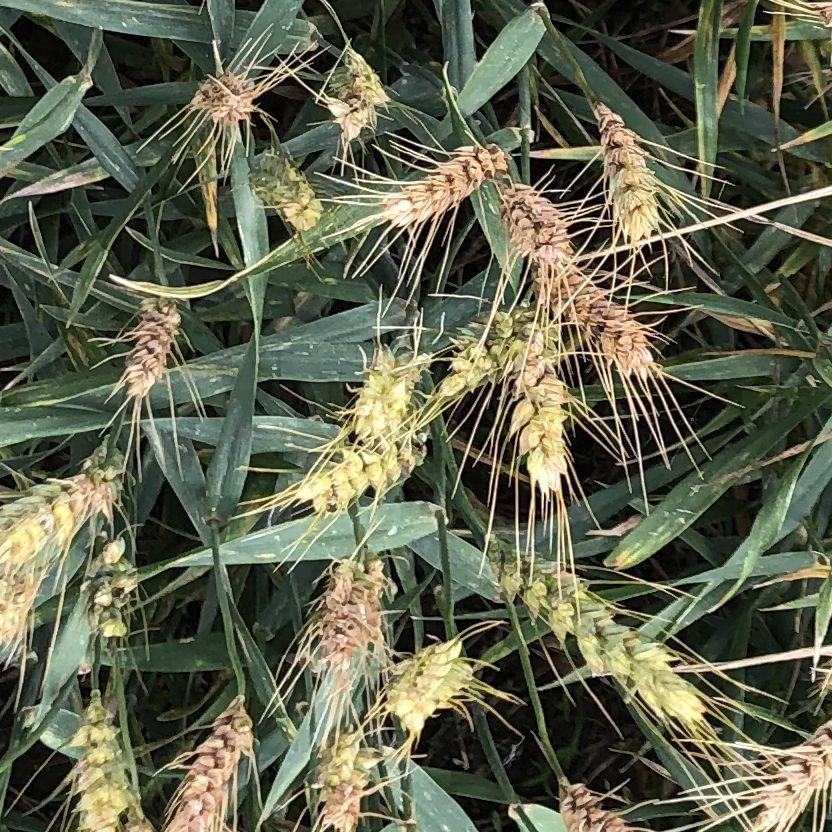

Supplement: Supplemental Information 2 [file peerj-cs-10-1948-s002.zip › data1/image0111.jpg]

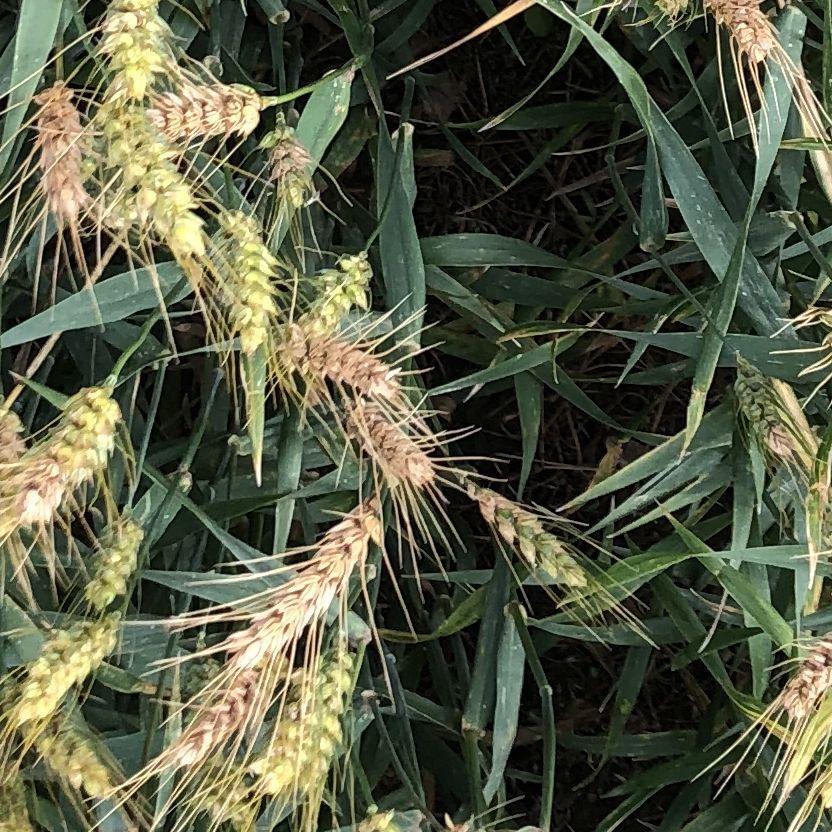

Supplement: Supplemental Information 2 [file peerj-cs-10-1948-s002.zip › data1/image0113.jpg]

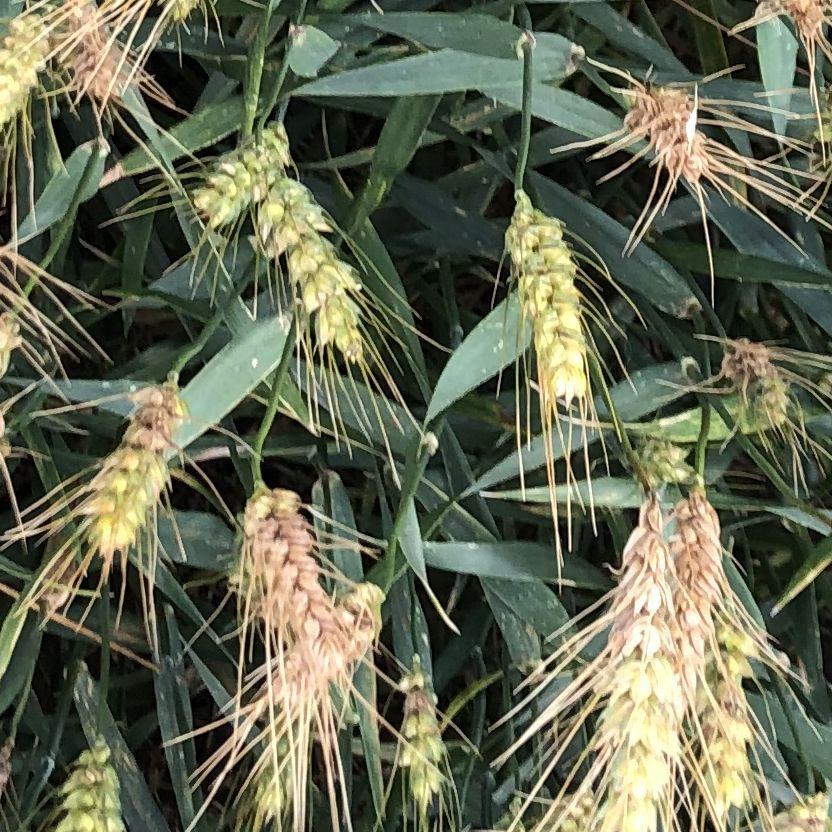

Supplement: Supplemental Information 2 [file peerj-cs-10-1948-s002.zip › data1/image0114.jpg]

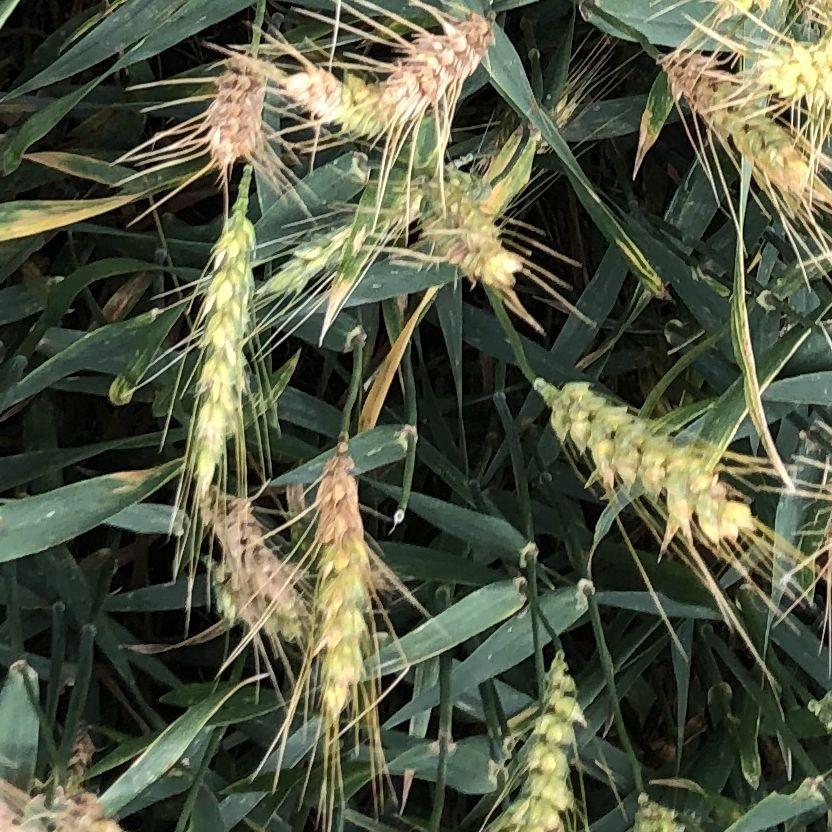

Supplement: Supplemental Information 2 [file peerj-cs-10-1948-s002.zip › data1/image0115.jpg]

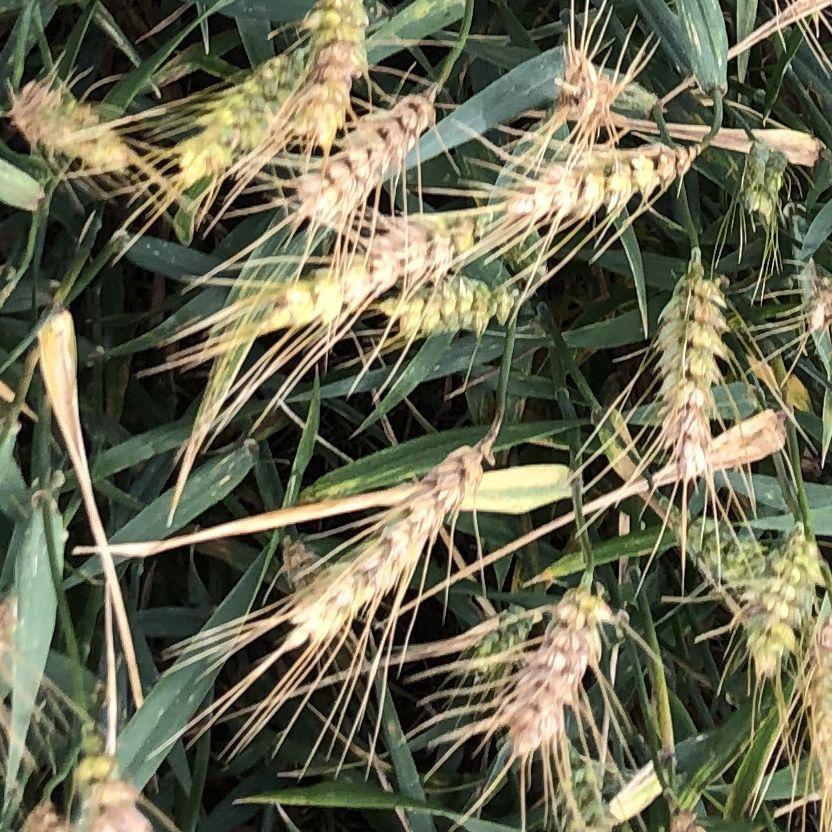

Supplement: Supplemental Information 2 [file peerj-cs-10-1948-s002.zip › data1/image0116.jpg]

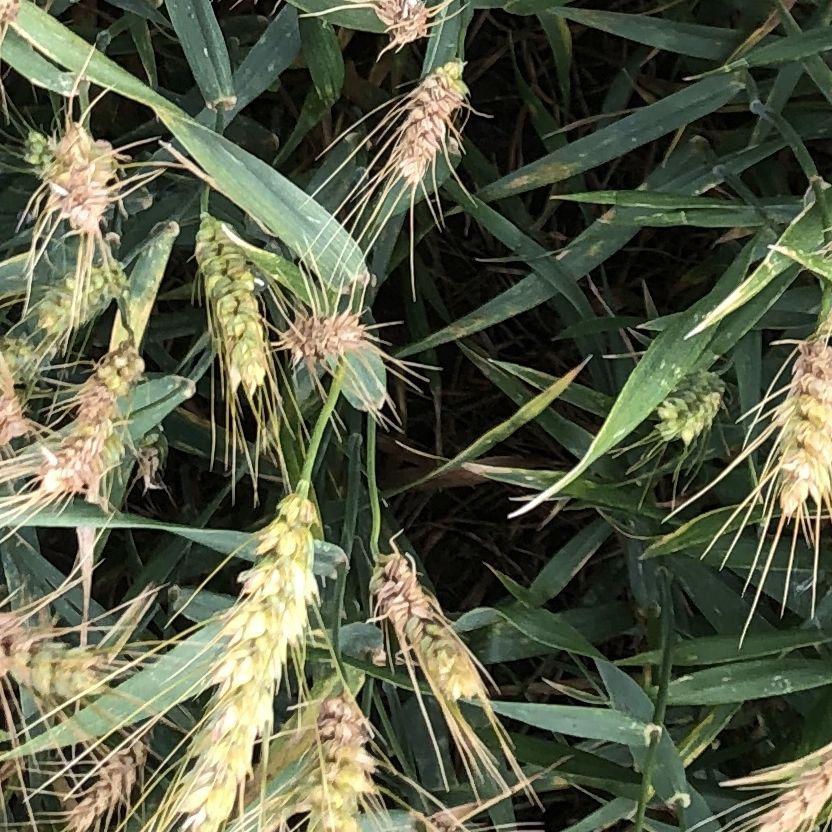

Supplement: Supplemental Information 2 [file peerj-cs-10-1948-s002.zip › data1/image0117.jpg]

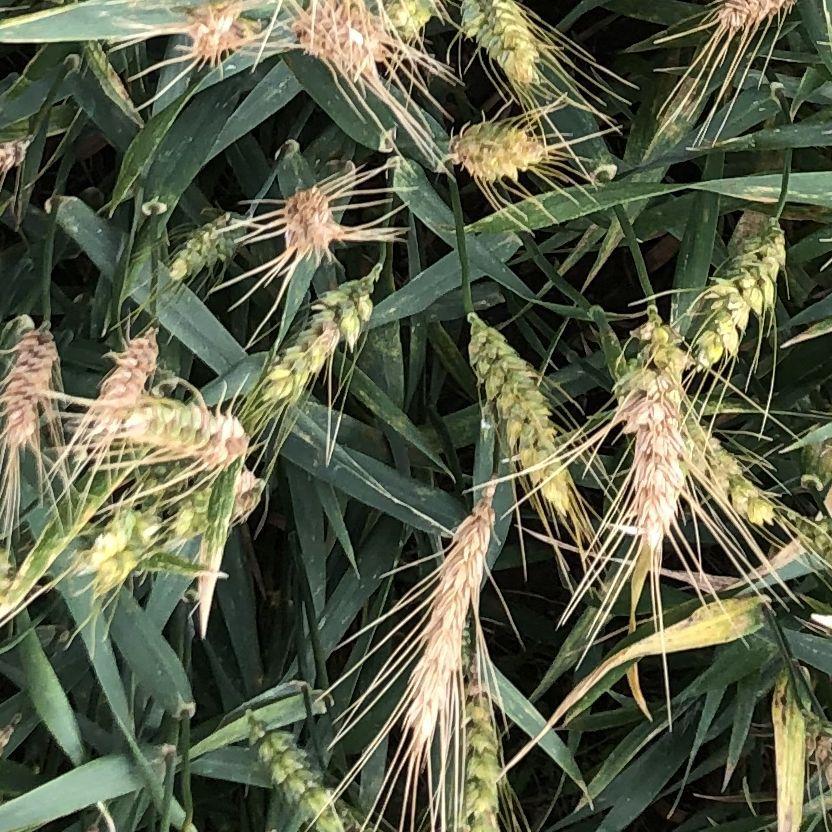

Supplement: Supplemental Information 2 [file peerj-cs-10-1948-s002.zip › data1/image0118.jpg]

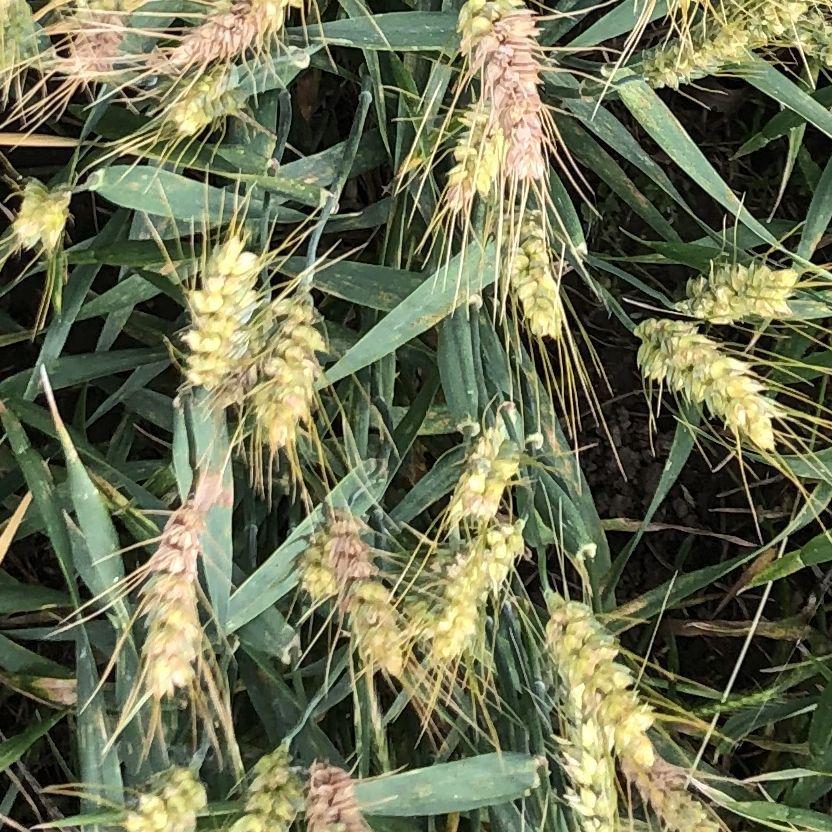

Supplement: Supplemental Information 2 [file peerj-cs-10-1948-s002.zip › data1/image0119.jpg]

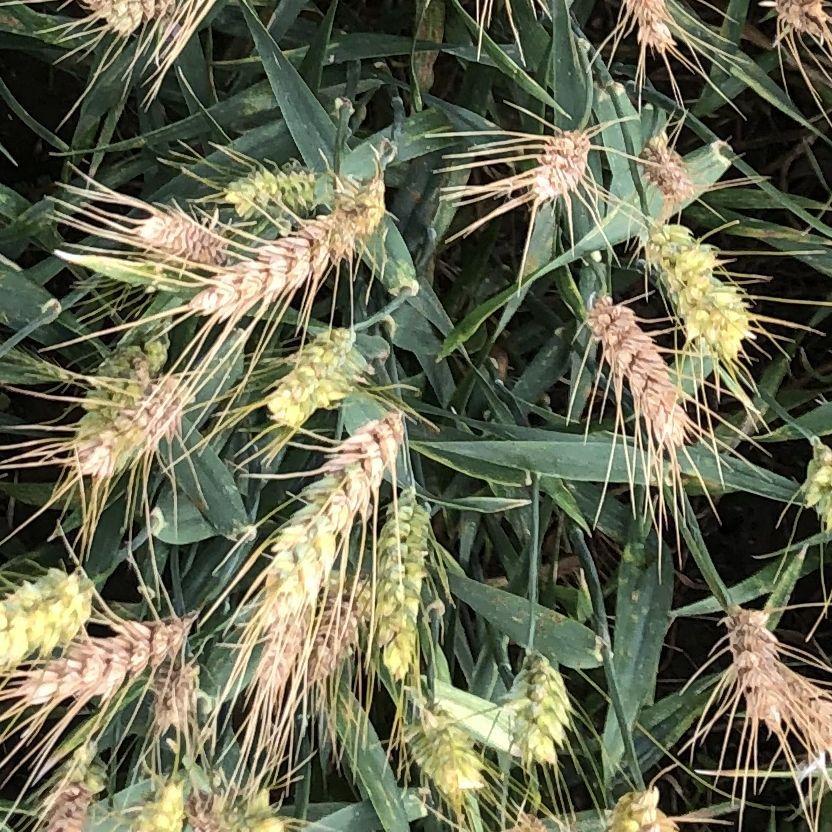

Supplement: Supplemental Information 2 [file peerj-cs-10-1948-s002.zip › data1/image0120.jpg]

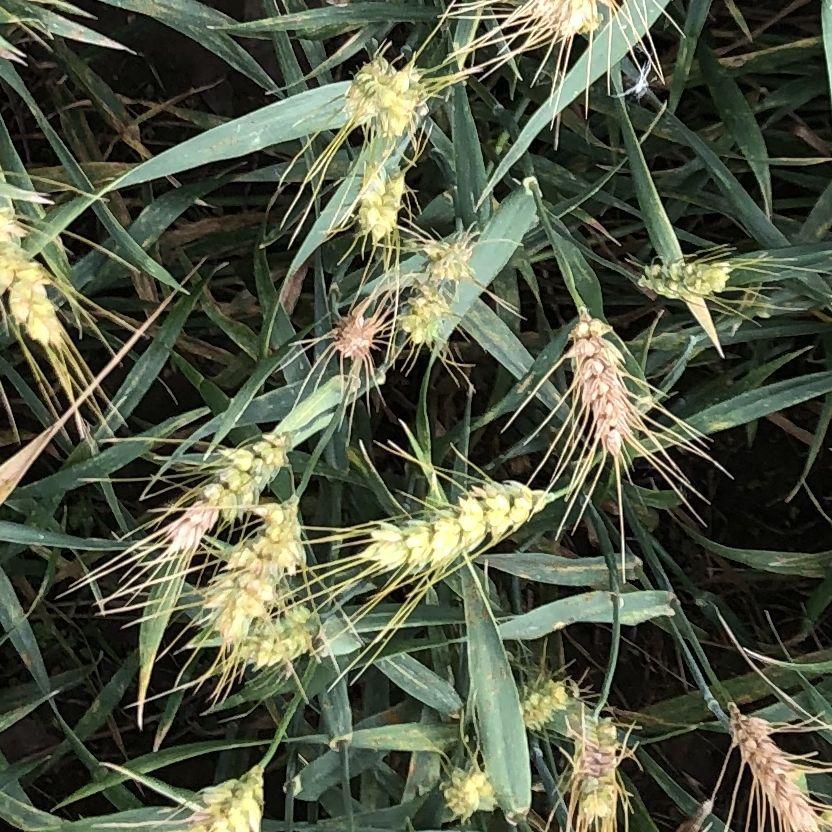

Supplement: Supplemental Information 2 [file peerj-cs-10-1948-s002.zip › data1/image0122.jpg]

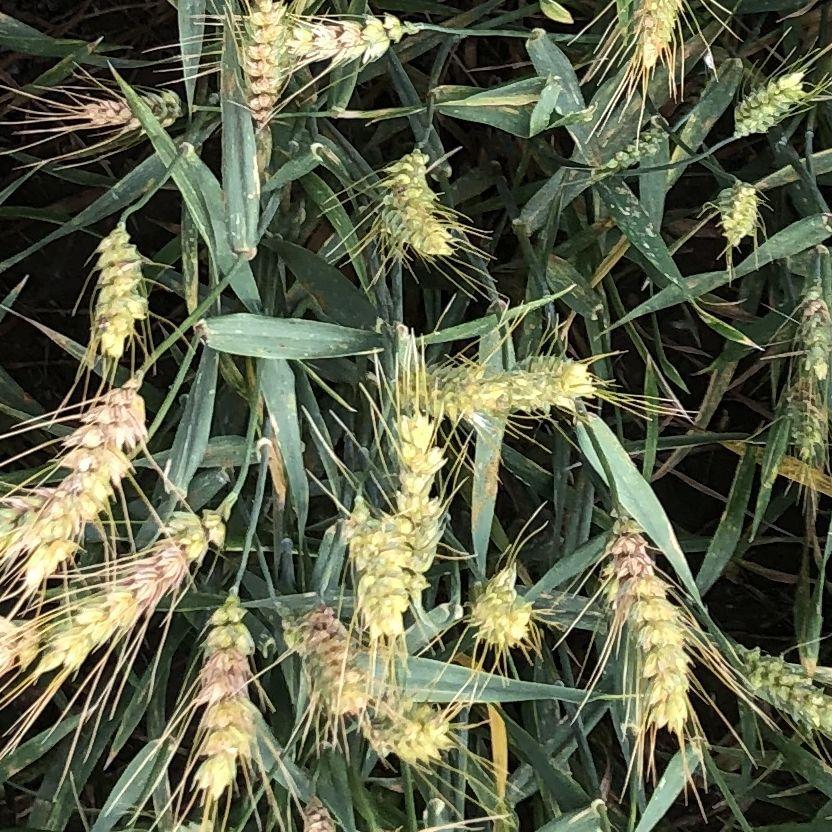

Supplement: Supplemental Information 2 [file peerj-cs-10-1948-s002.zip › data1/image0123.jpg]

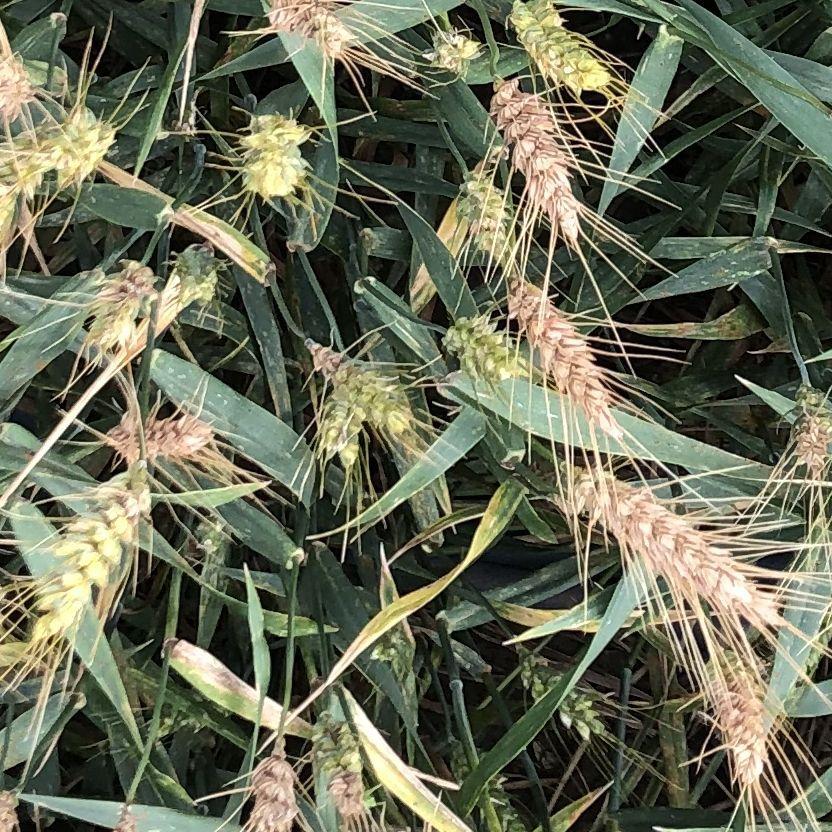

Supplement: Supplemental Information 2 [file peerj-cs-10-1948-s002.zip › data1/image0125.jpg]

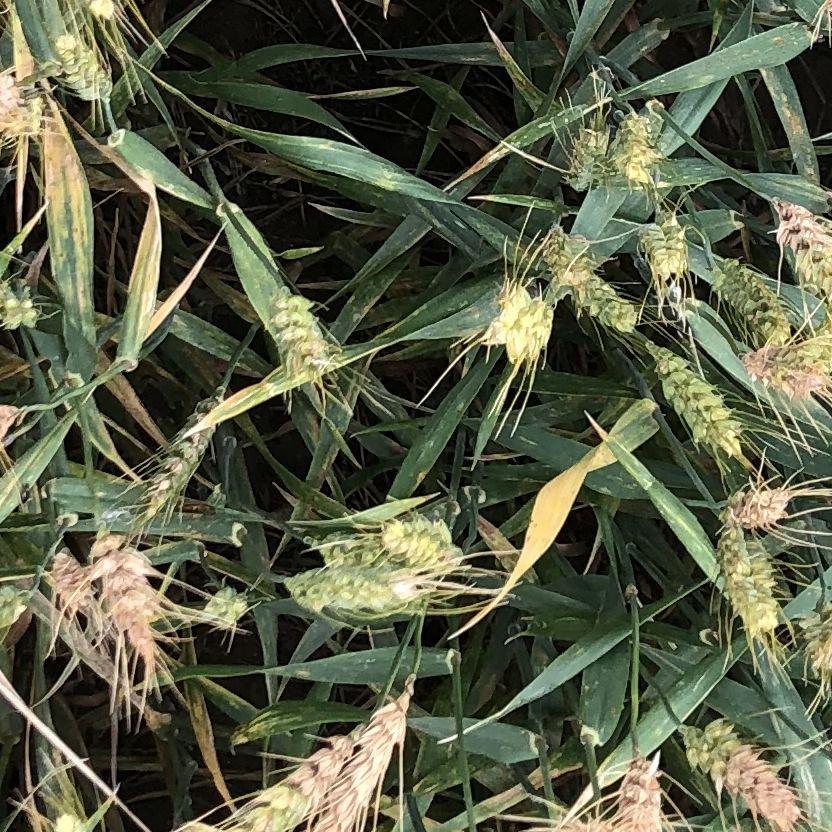

Supplement: Supplemental Information 2 [file peerj-cs-10-1948-s002.zip › data1/image0126.jpg]

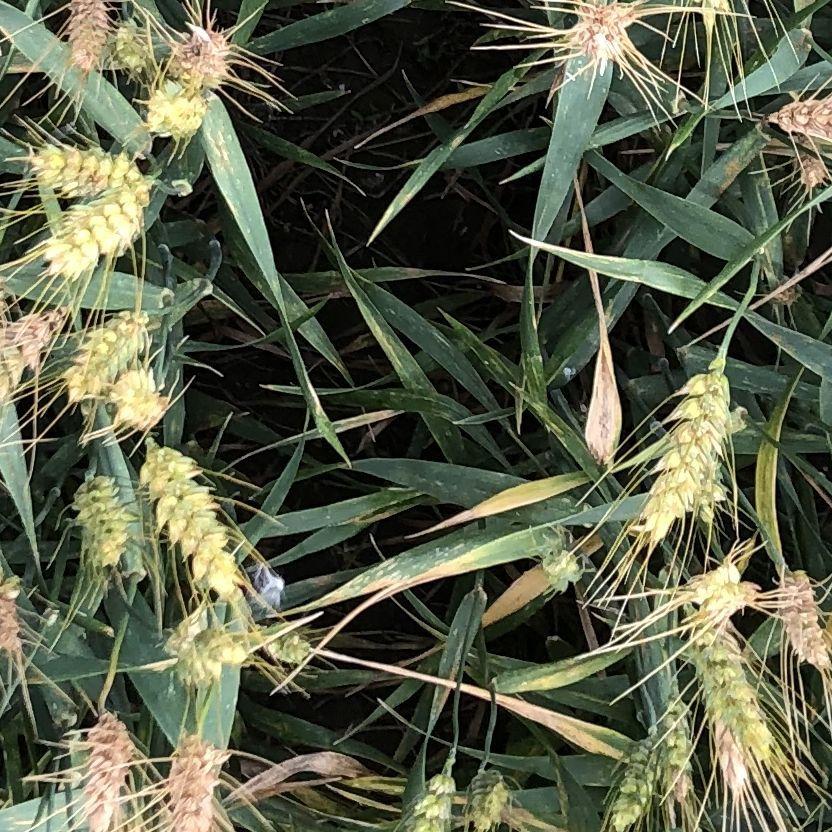

Supplement: Supplemental Information 2 [file peerj-cs-10-1948-s002.zip › data1/image0127.jpg]

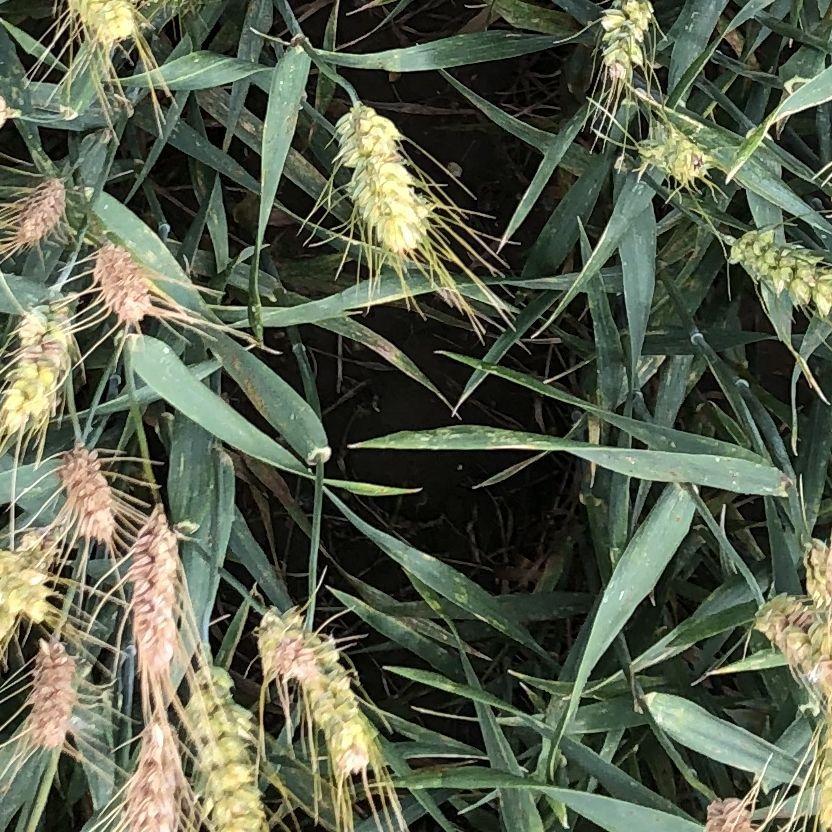

Supplement: Supplemental Information 2 [file peerj-cs-10-1948-s002.zip › data1/image0128.jpg]

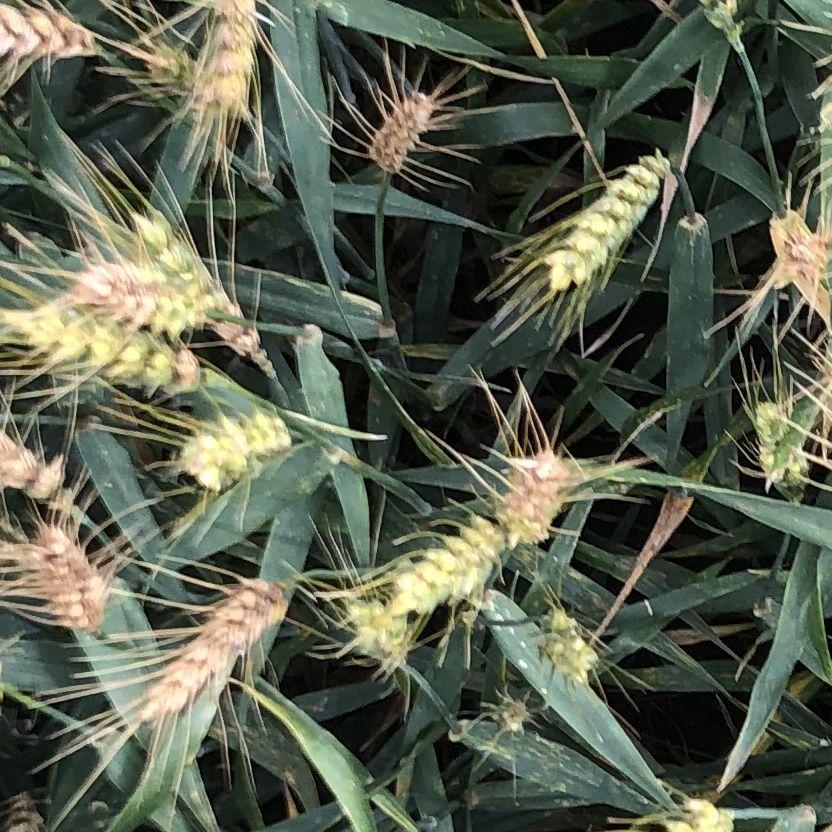

Supplement: Supplemental Information 2 [file peerj-cs-10-1948-s002.zip › data1/image0129.jpg]

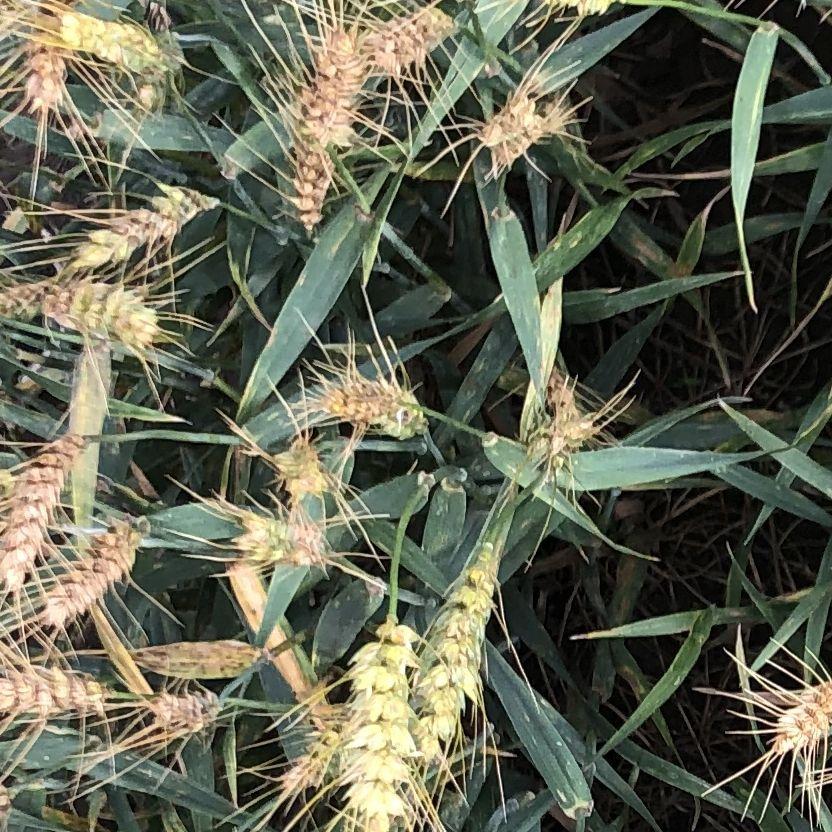

Supplement: Supplemental Information 2 [file peerj-cs-10-1948-s002.zip › data1/image0131.jpg]

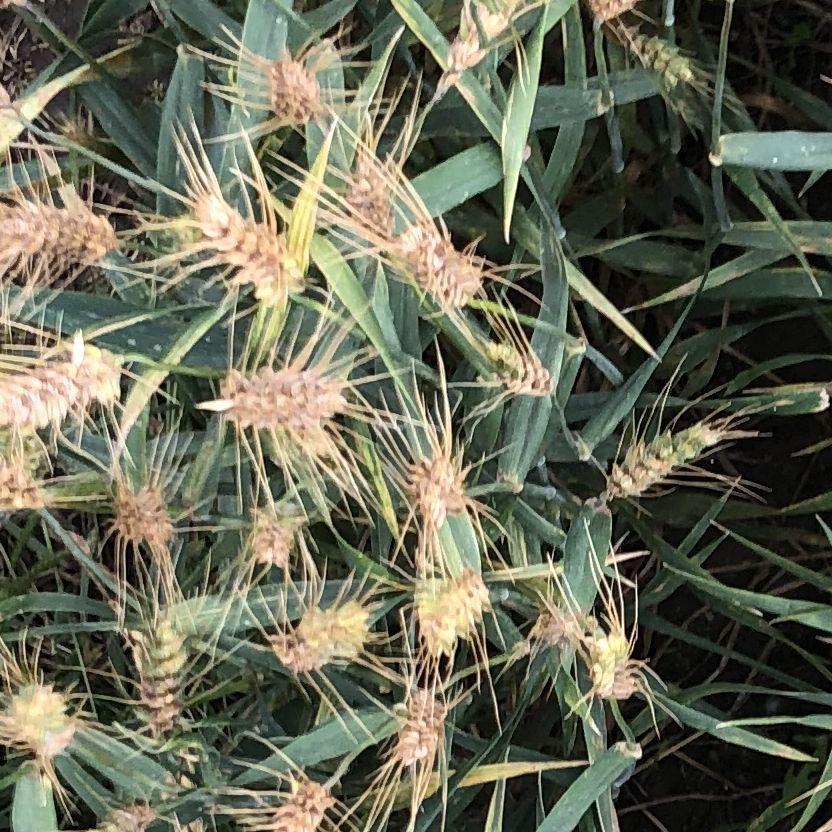

Supplement: Supplemental Information 2 [file peerj-cs-10-1948-s002.zip › data1/image0132.jpg]

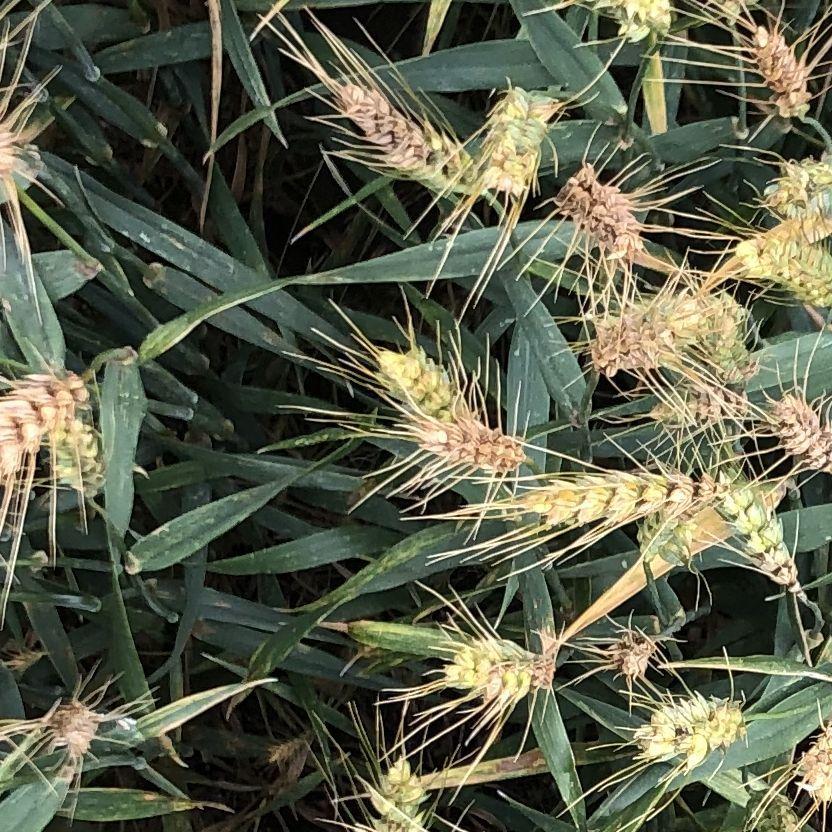

Supplement: Supplemental Information 2 [file peerj-cs-10-1948-s002.zip › data1/image0133.jpg]

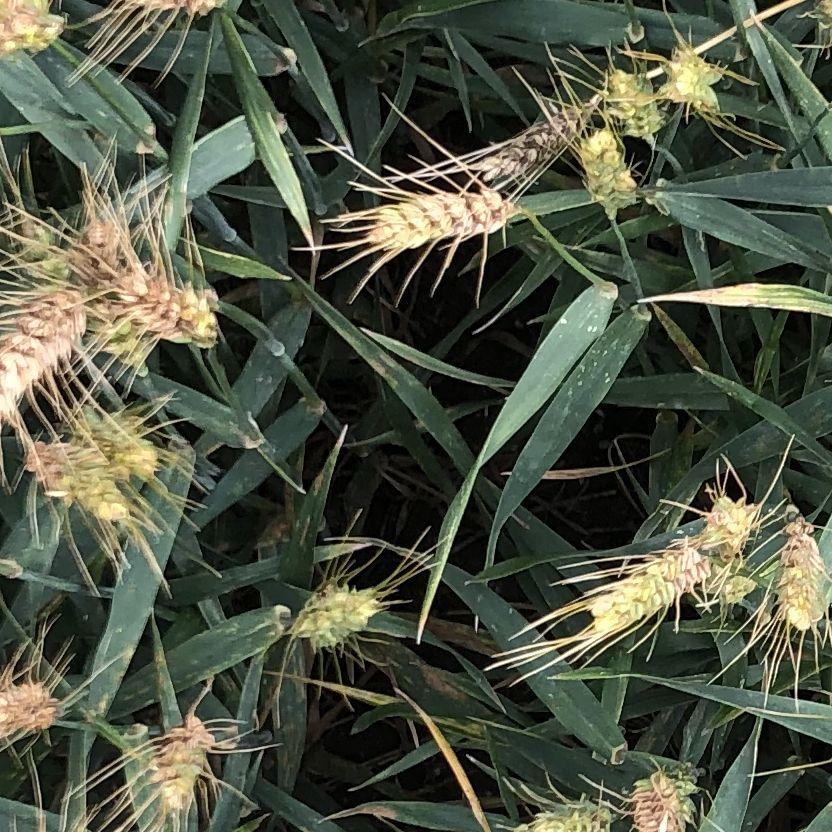

Supplement: Supplemental Information 2 [file peerj-cs-10-1948-s002.zip › data1/image0134.jpg]

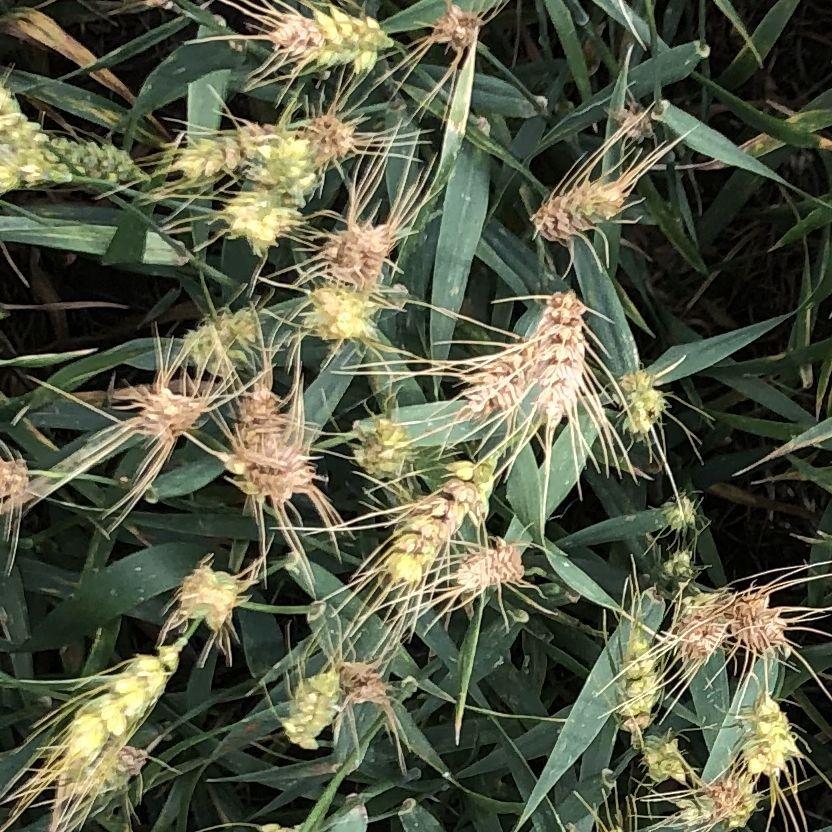

Supplement: Supplemental Information 2 [file peerj-cs-10-1948-s002.zip › data1/image0135.jpg]

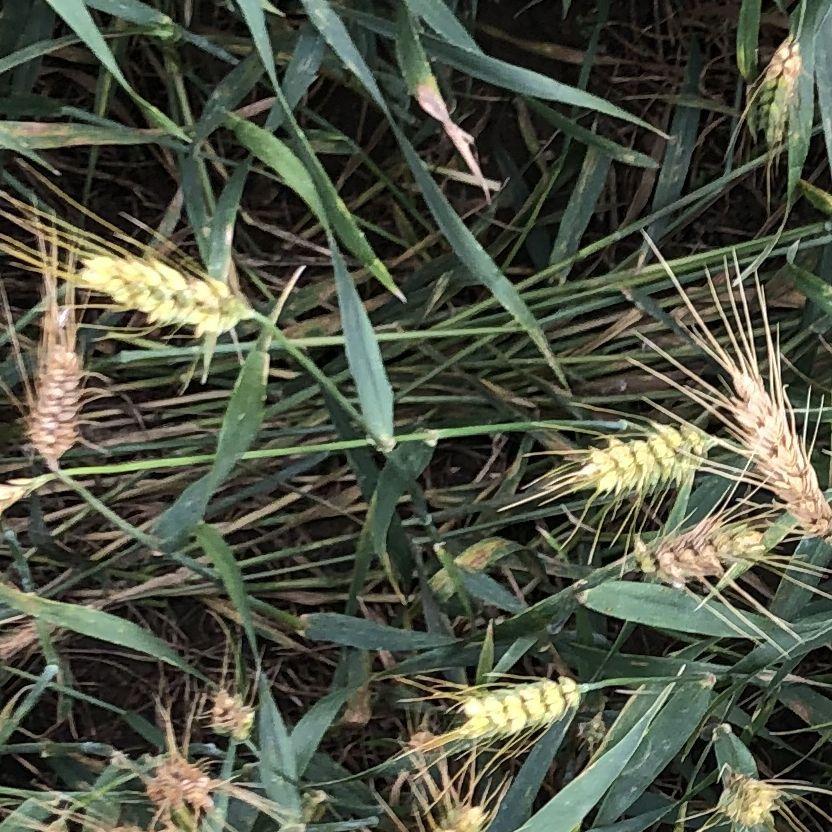

Supplement: Supplemental Information 2 [file peerj-cs-10-1948-s002.zip › data1/image0136.jpg]

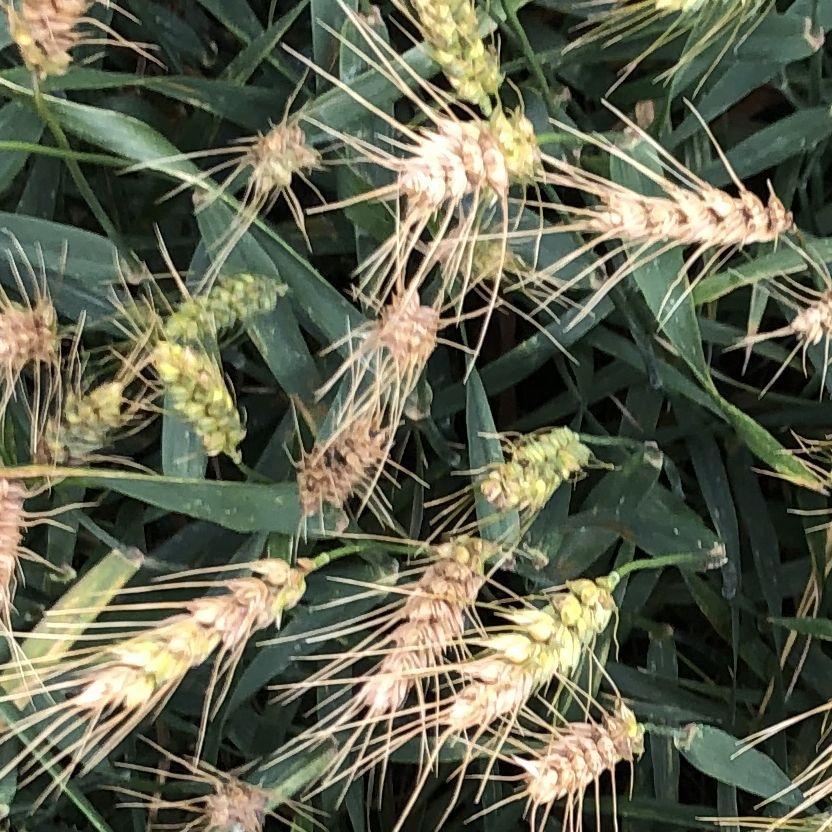

Supplement: Supplemental Information 2 [file peerj-cs-10-1948-s002.zip › data1/image0138.jpg]

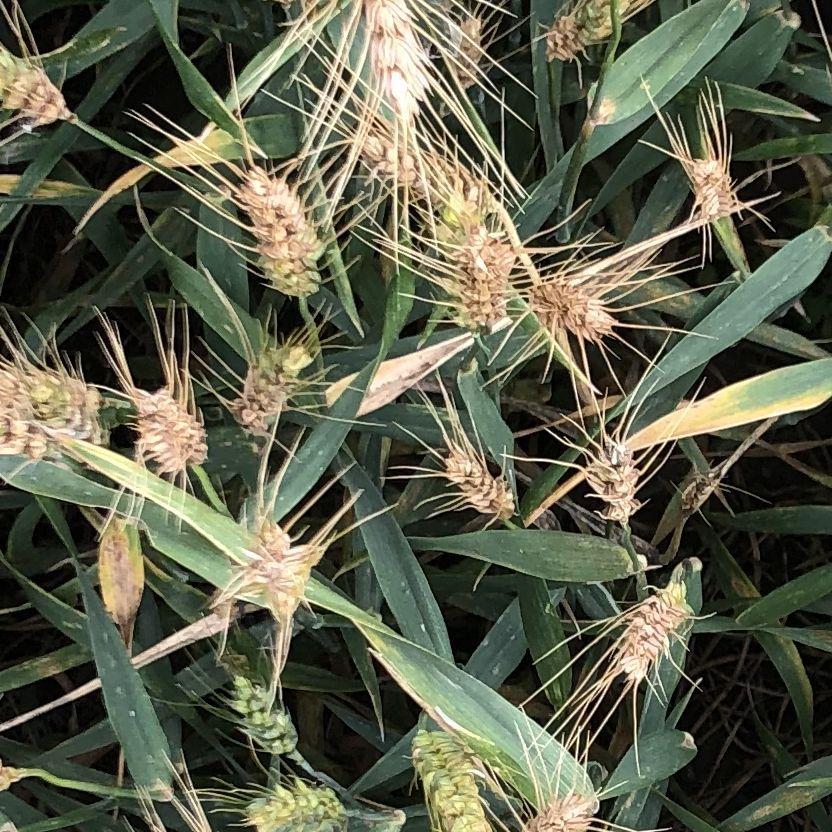

Supplement: Supplemental Information 2 [file peerj-cs-10-1948-s002.zip › data1/image0142.jpg]

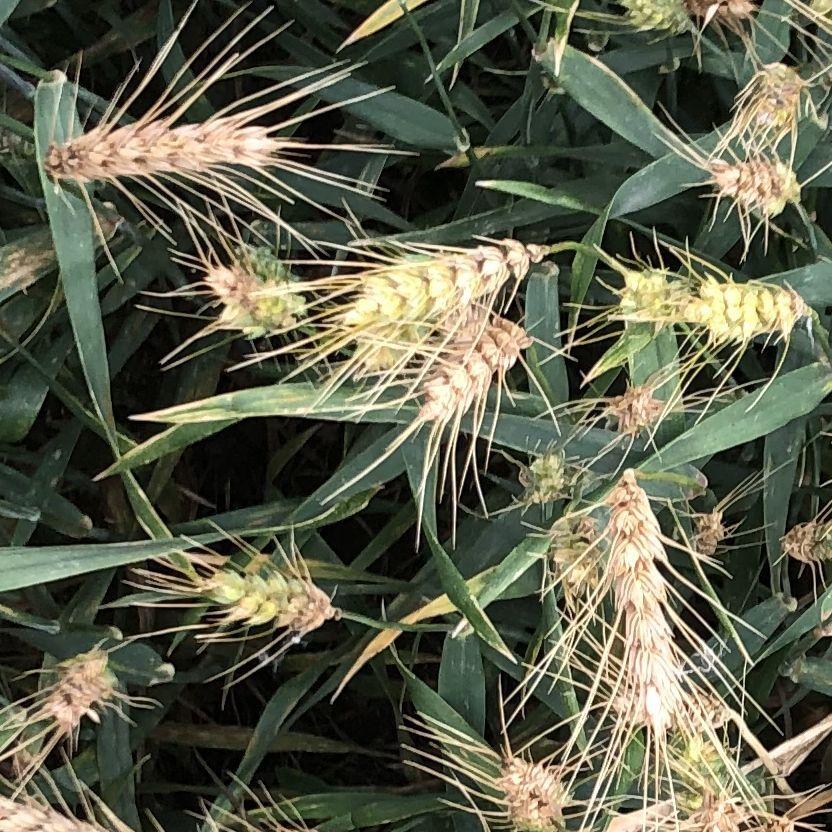

Supplement: Supplemental Information 2 [file peerj-cs-10-1948-s002.zip › data1/image0143.jpg]
