# Supplementary figures and images for: YOLOv8s-CGF: a lightweight model for wheat ear Fusarium head blight detection (part 3 of 3)
Source: PeerJ Comput Sci. 2024 Mar 27;10:e1948. doi: 10.7717/peerj-cs.1948 (PMC11041926; doi:10.7717/peerj-cs.1948)

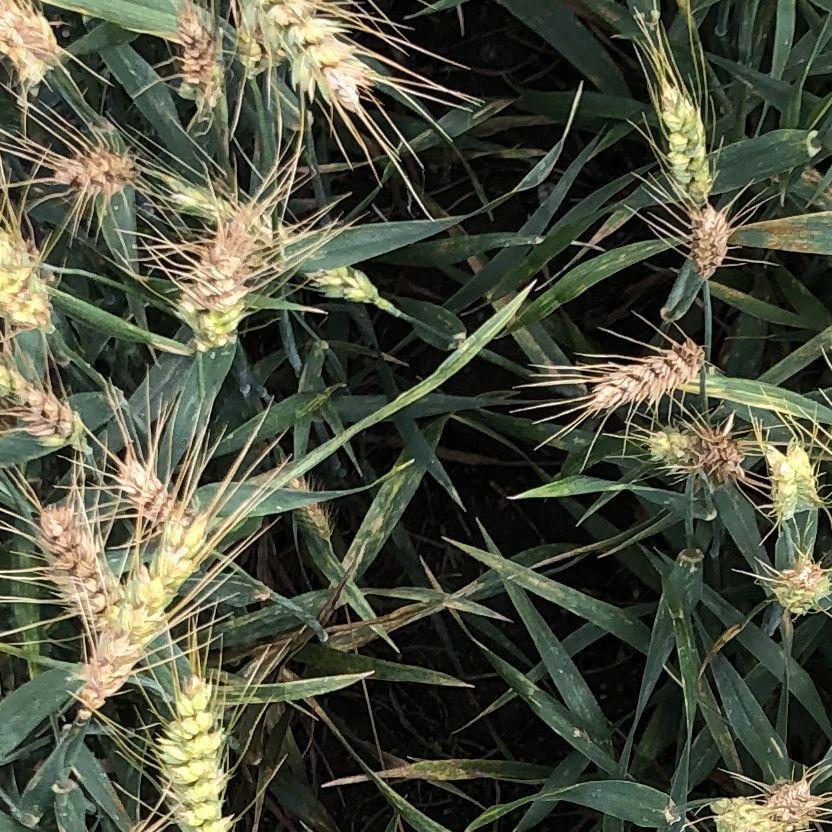

Supplement: Supplemental Information 3 [file peerj-cs-10-1948-s003.zip › data2/image0270.jpg]

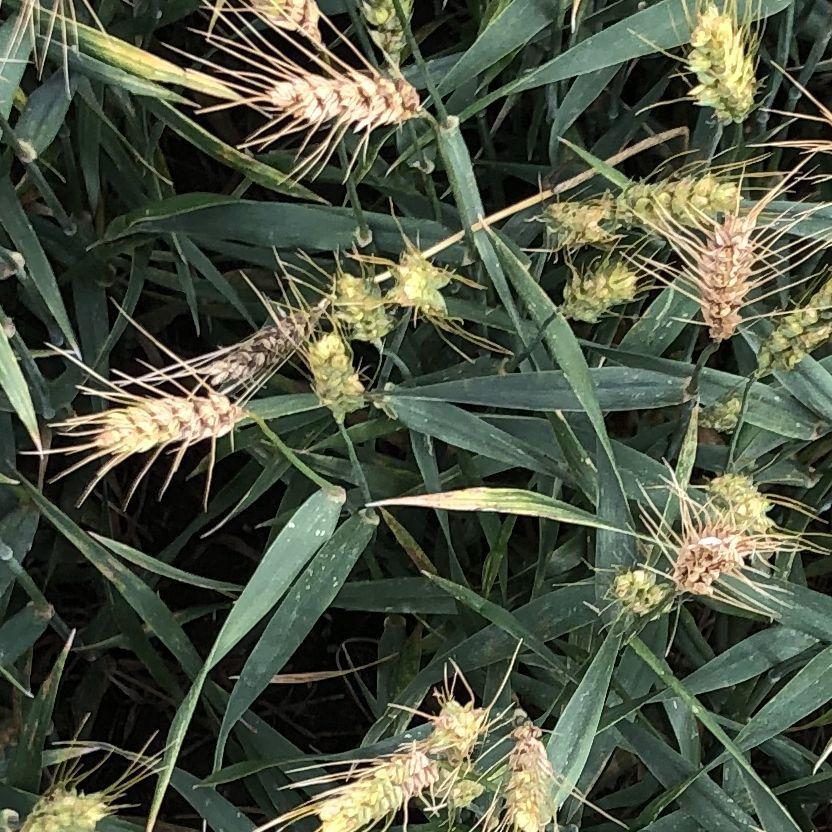

Supplement: Supplemental Information 3 [file peerj-cs-10-1948-s003.zip › data2/image0271.jpg]

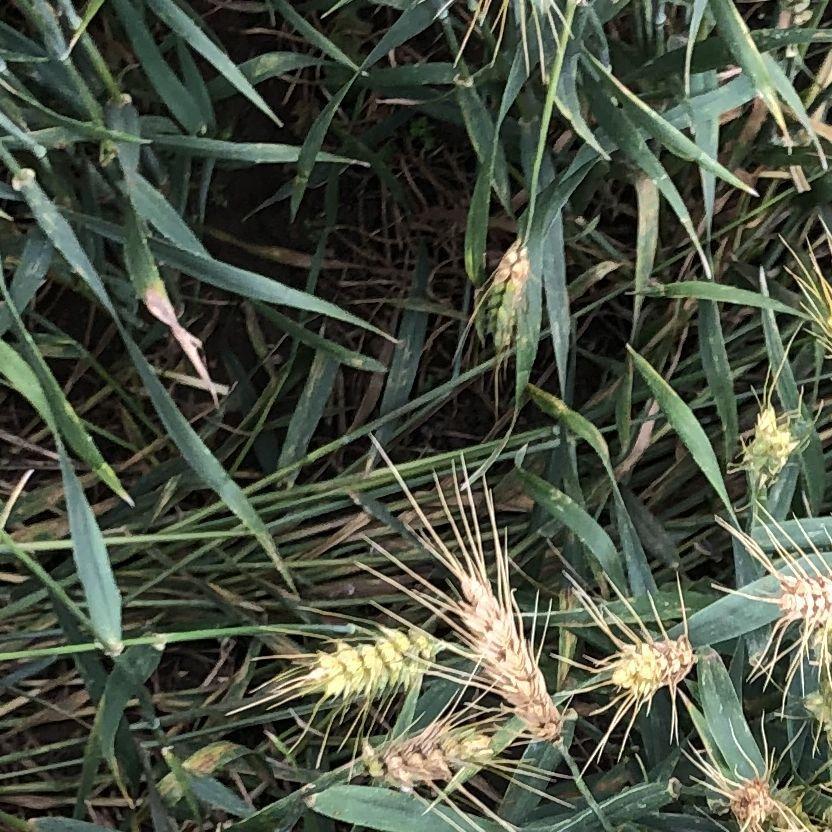

Supplement: Supplemental Information 3 [file peerj-cs-10-1948-s003.zip › data2/image0272.jpg]

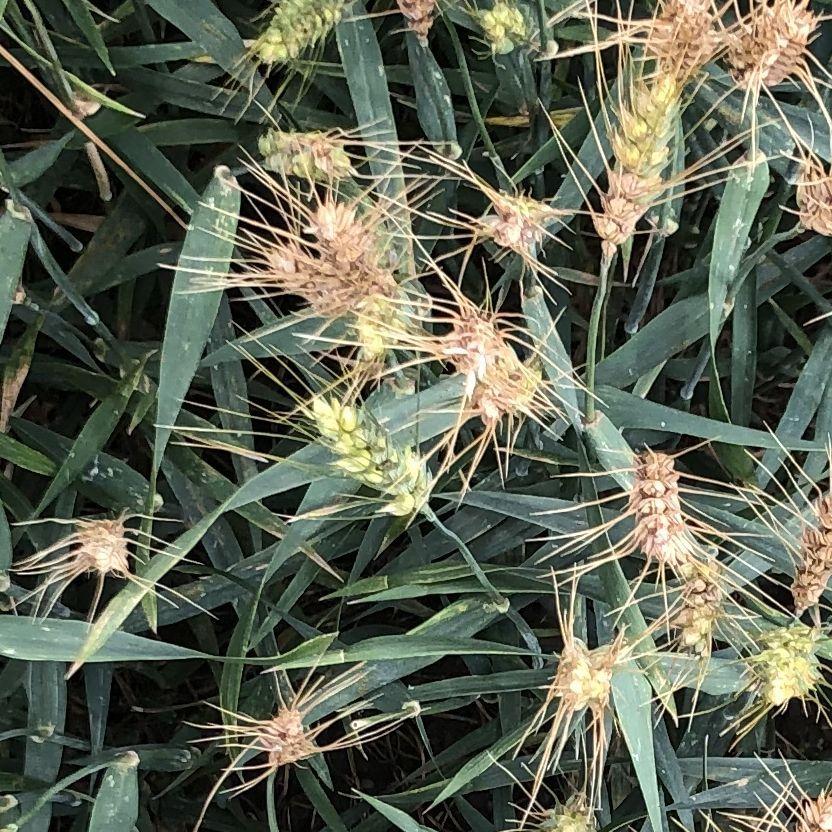

Supplement: Supplemental Information 3 [file peerj-cs-10-1948-s003.zip › data2/image0273.jpg]

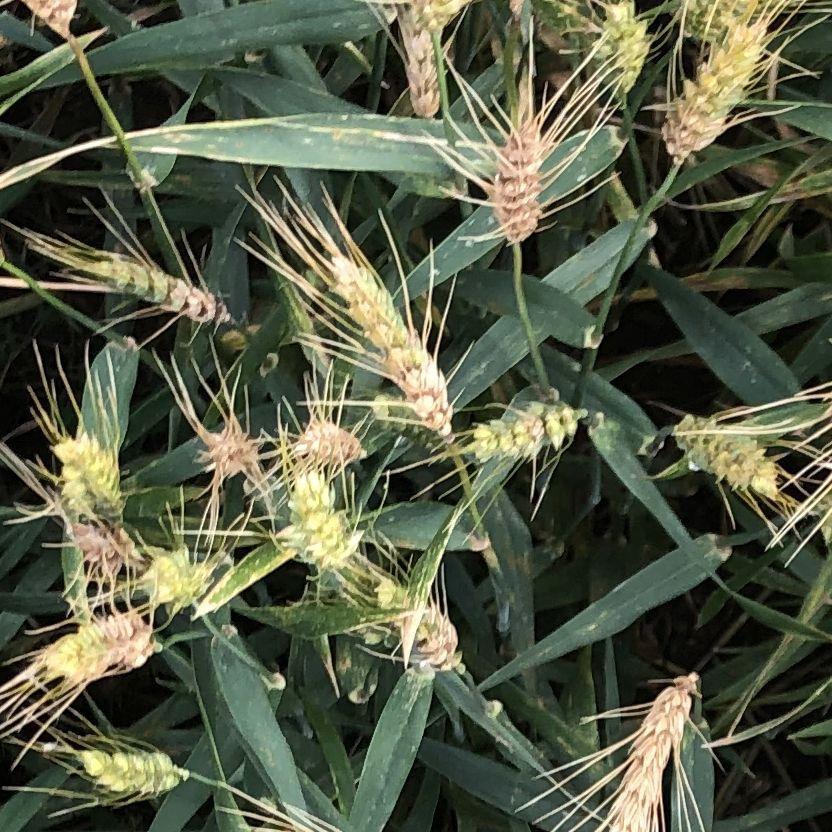

Supplement: Supplemental Information 3 [file peerj-cs-10-1948-s003.zip › data2/image0274.jpg]

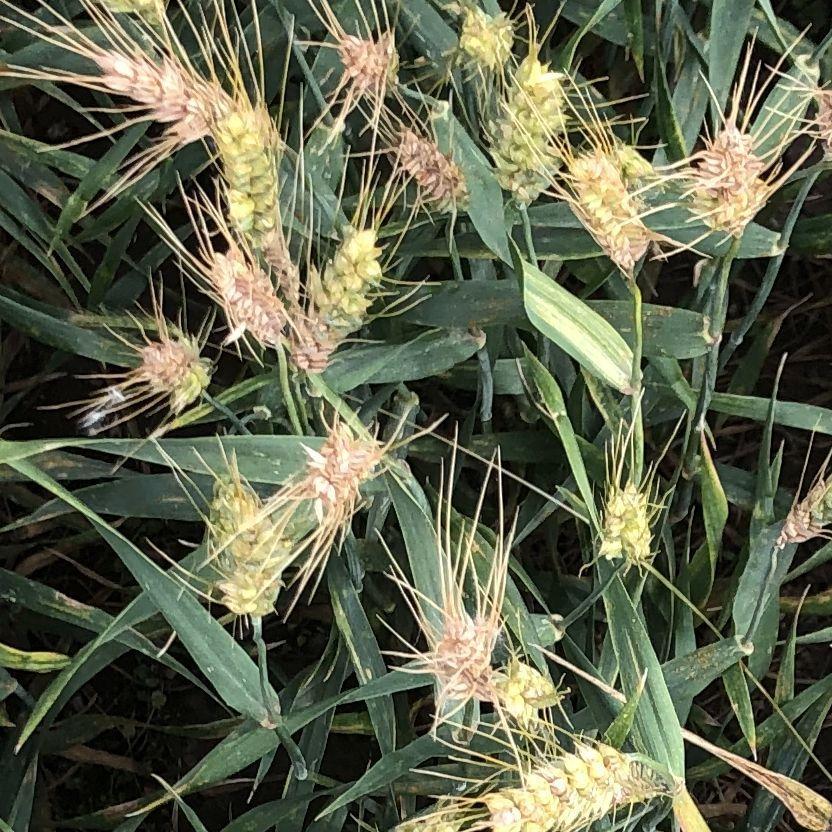

Supplement: Supplemental Information 3 [file peerj-cs-10-1948-s003.zip › data2/image0275.jpg]

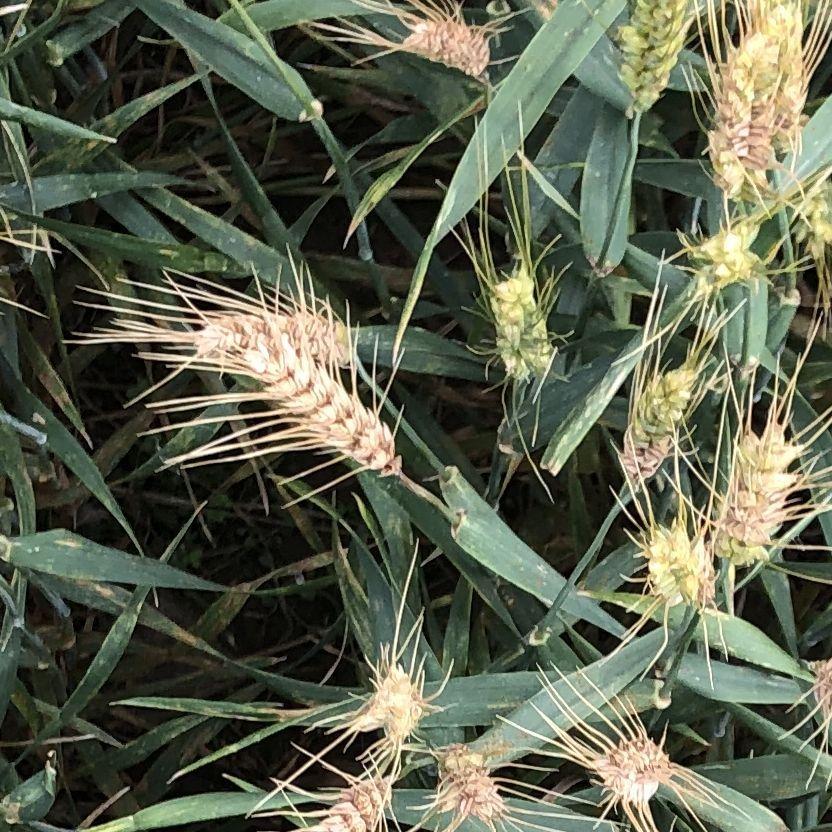

Supplement: Supplemental Information 3 [file peerj-cs-10-1948-s003.zip › data2/image0276.jpg]

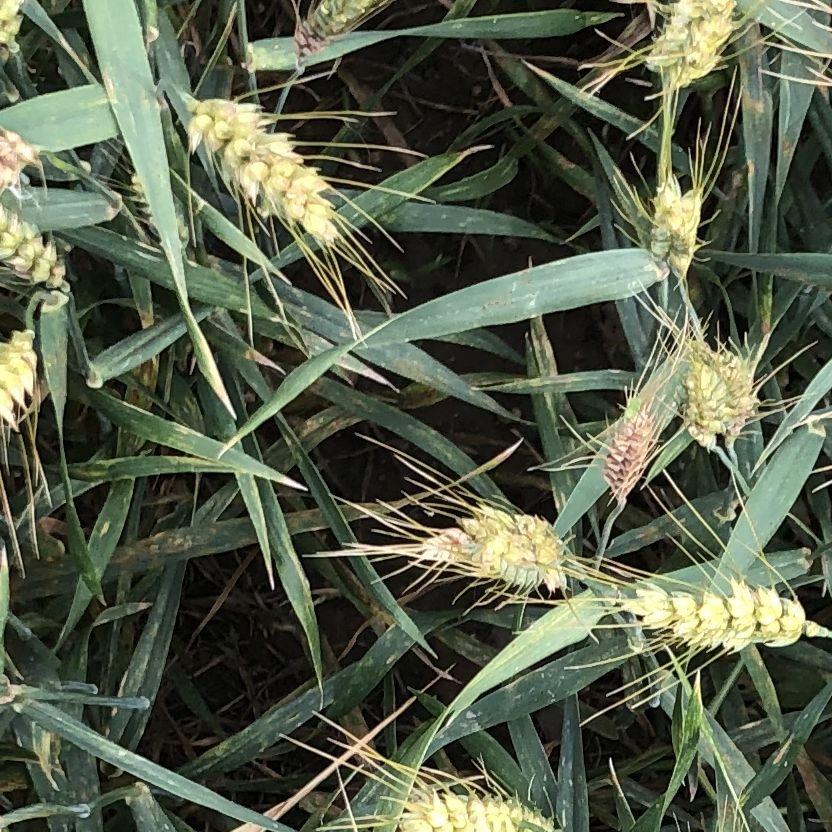

Supplement: Supplemental Information 3 [file peerj-cs-10-1948-s003.zip › data2/image0277.jpg]

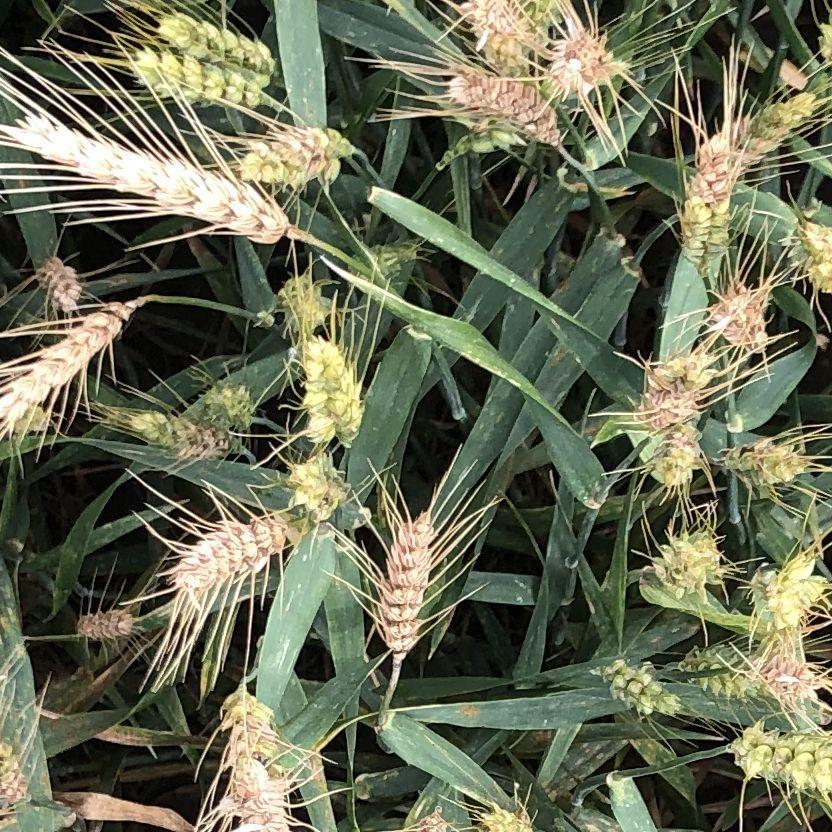

Supplement: Supplemental Information 3 [file peerj-cs-10-1948-s003.zip › data2/image0278.jpg]

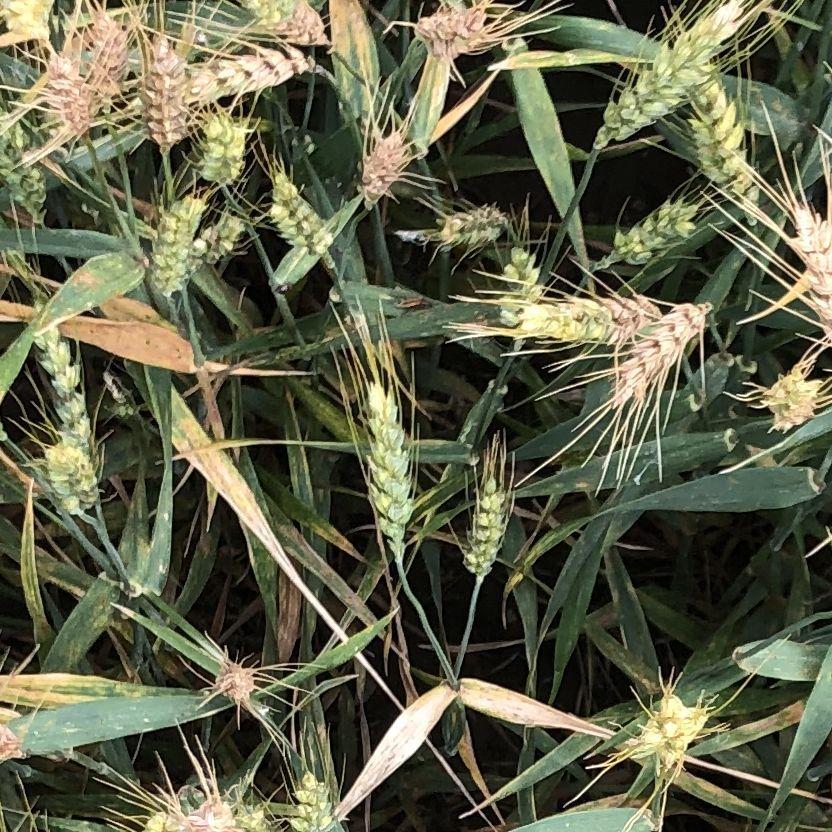

Supplement: Supplemental Information 3 [file peerj-cs-10-1948-s003.zip › data2/image0279.jpg]

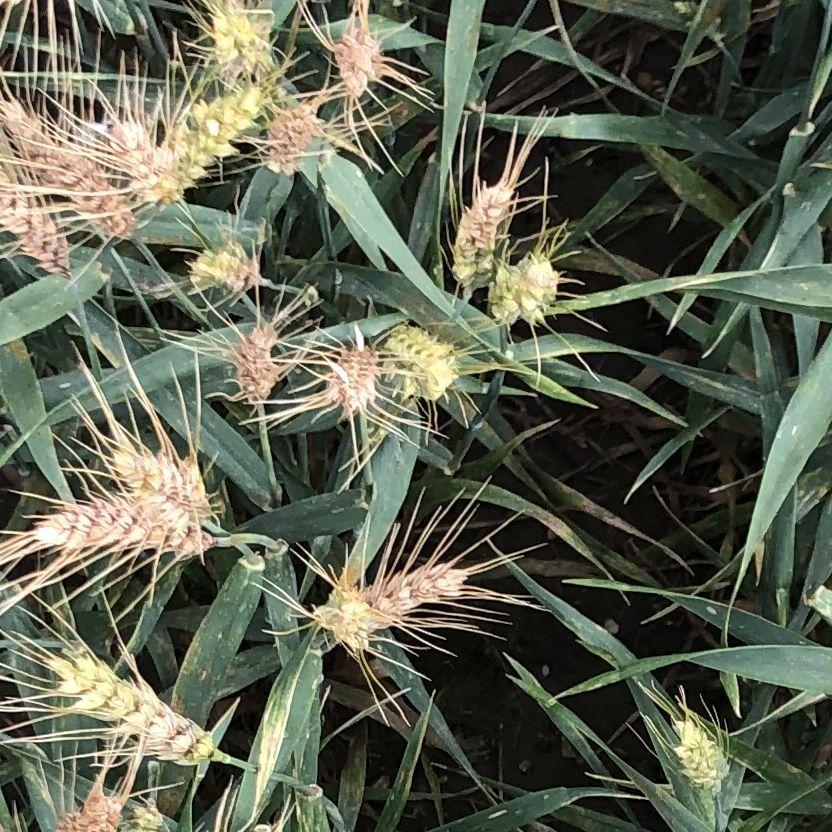

Supplement: Supplemental Information 3 [file peerj-cs-10-1948-s003.zip › data2/image0280.jpg]

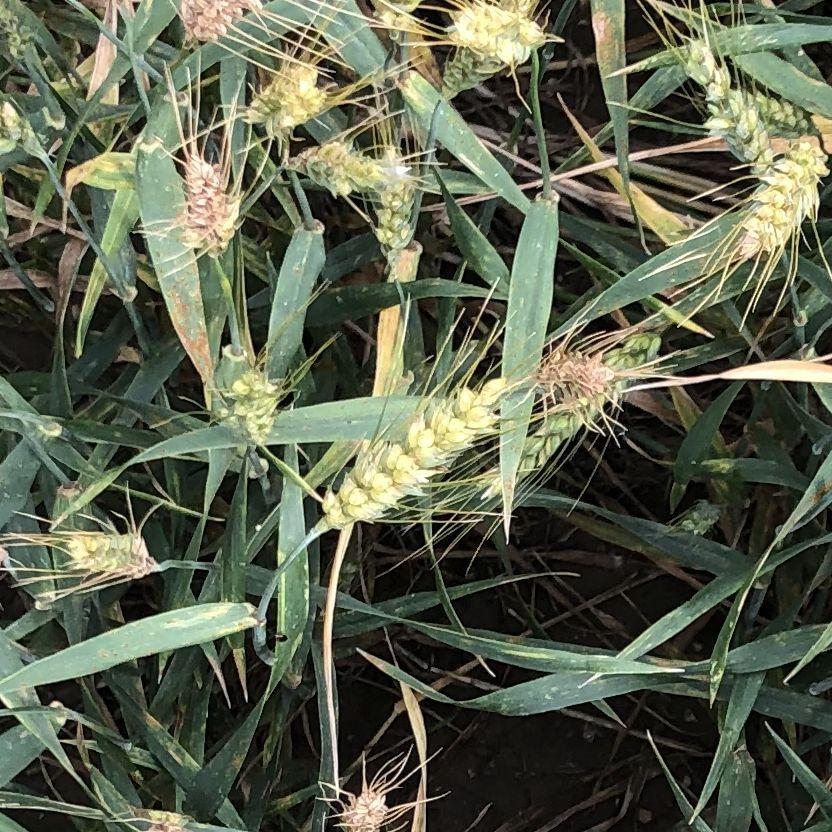

Supplement: Supplemental Information 3 [file peerj-cs-10-1948-s003.zip › data2/image0281.jpg]

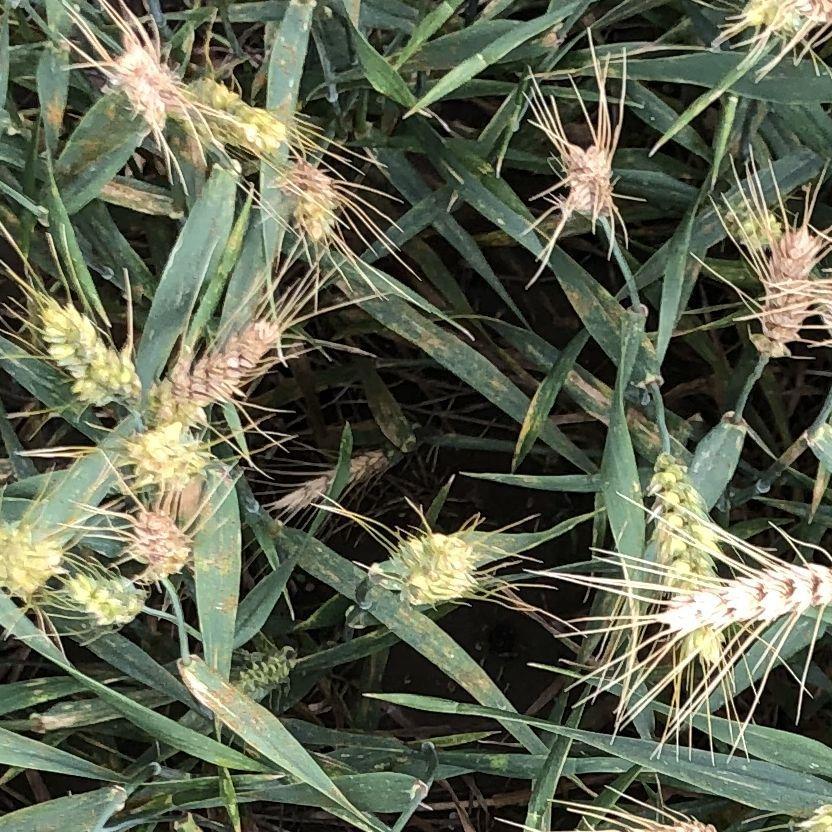

Supplement: Supplemental Information 3 [file peerj-cs-10-1948-s003.zip › data2/image0282.jpg]

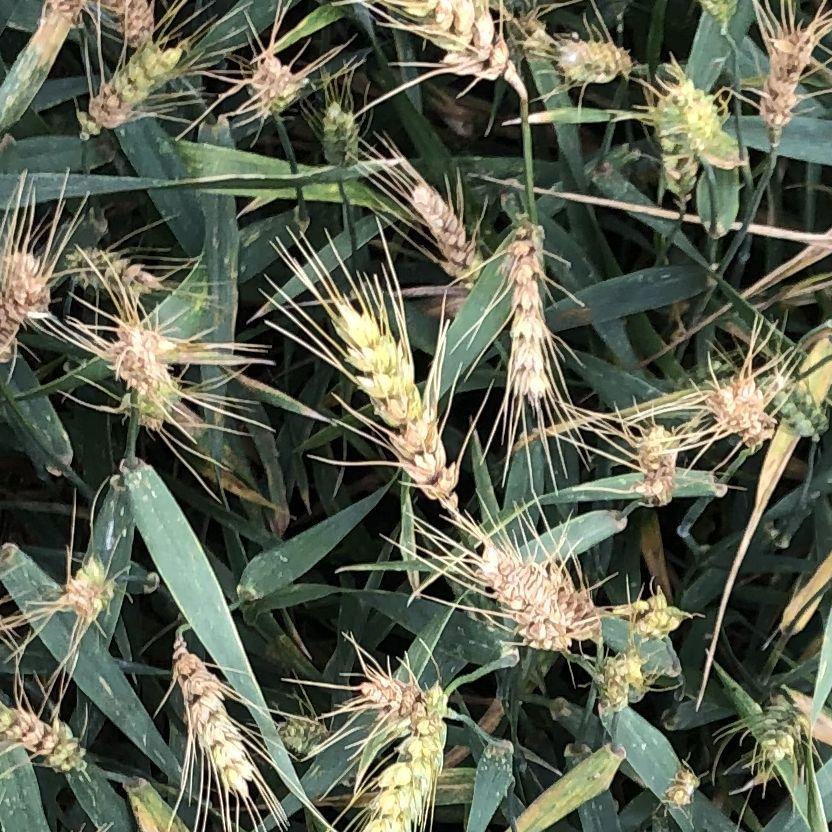

Supplement: Supplemental Information 3 [file peerj-cs-10-1948-s003.zip › data2/image0283.jpg]

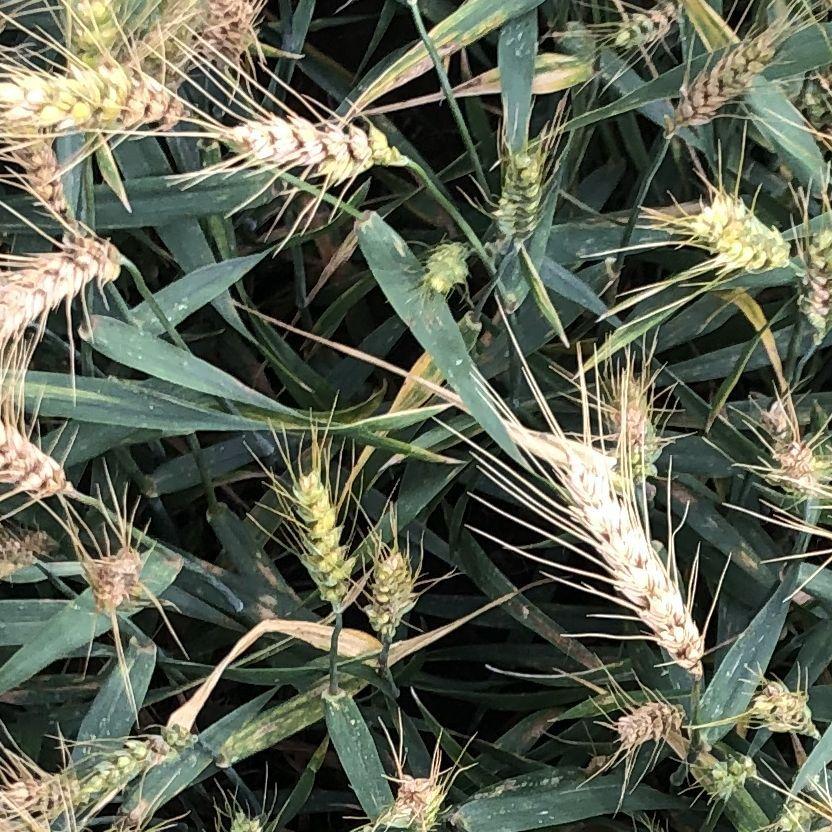

Supplement: Supplemental Information 3 [file peerj-cs-10-1948-s003.zip › data2/image0284.jpg]

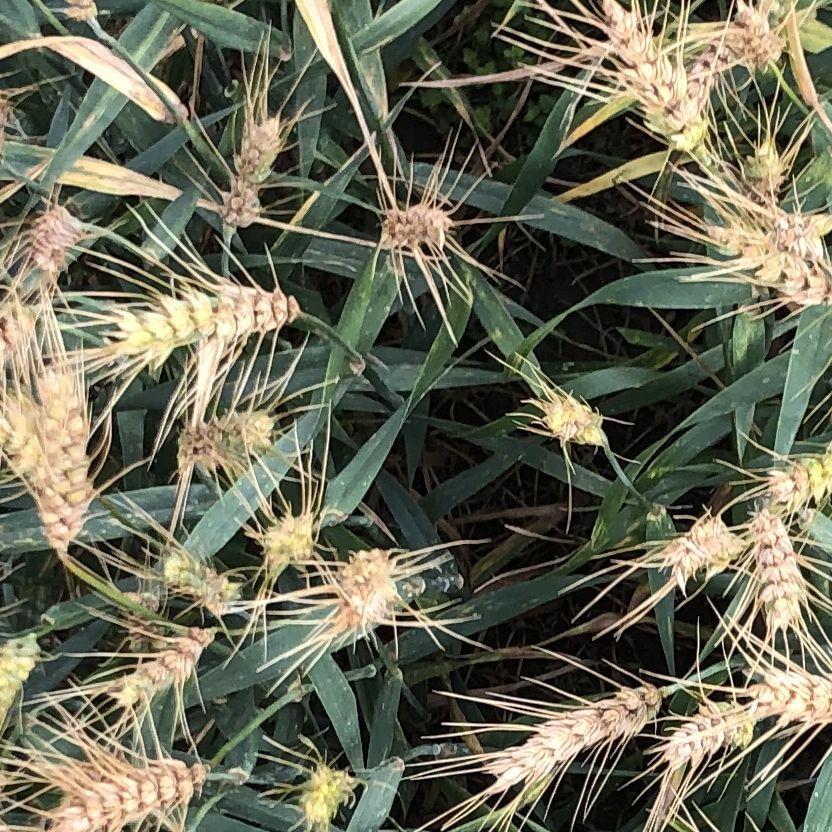

Supplement: Supplemental Information 3 [file peerj-cs-10-1948-s003.zip › data2/image0285.jpg]

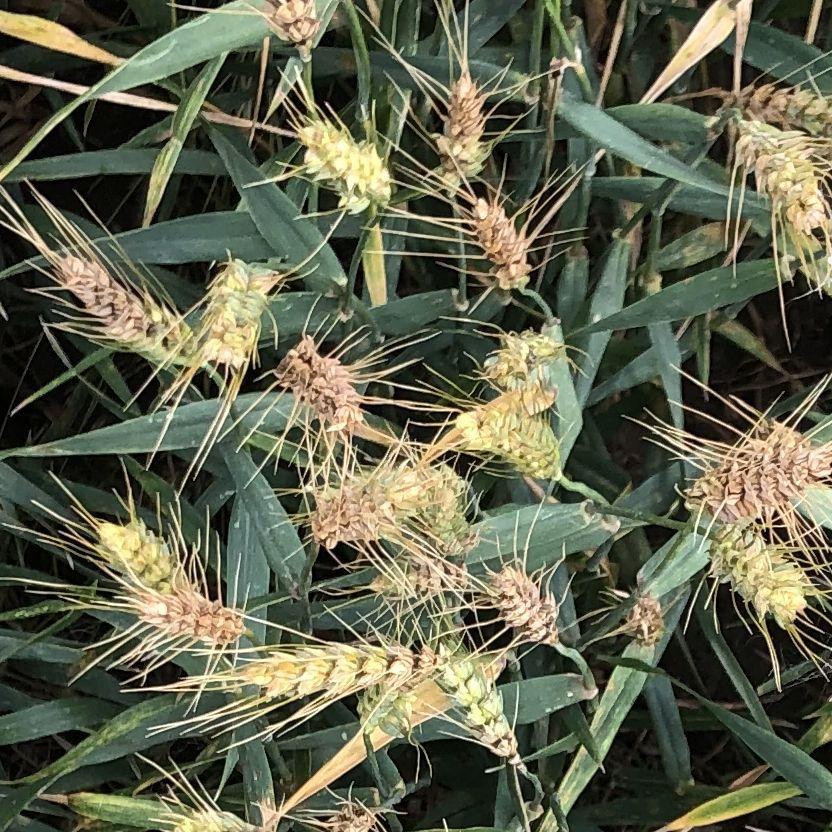

Supplement: Supplemental Information 3 [file peerj-cs-10-1948-s003.zip › data2/image0286.jpg]

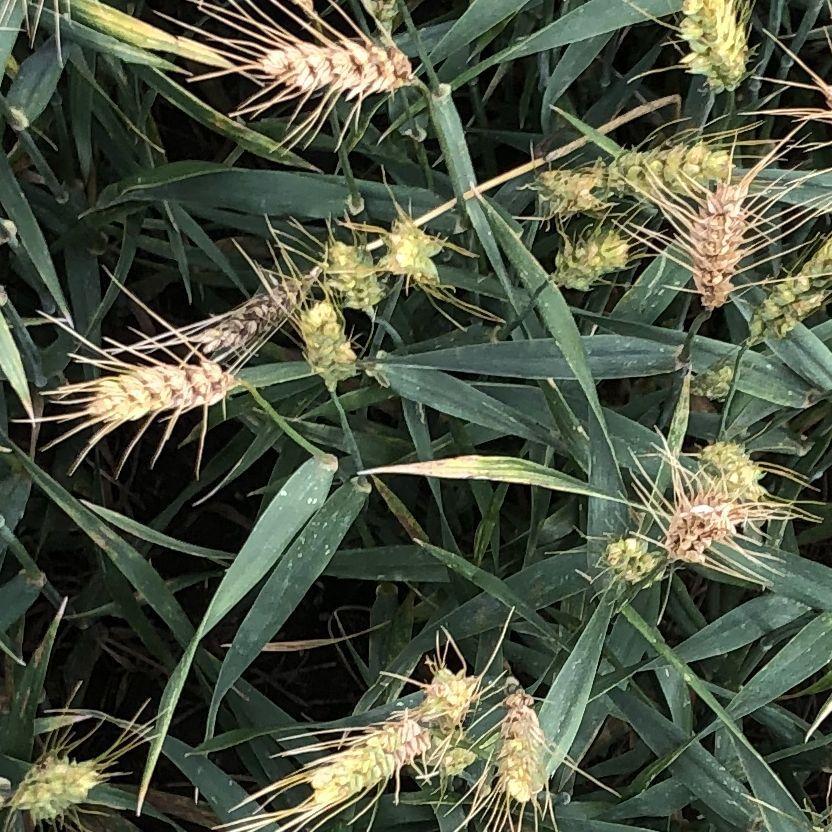

Supplement: Supplemental Information 3 [file peerj-cs-10-1948-s003.zip › data2/image0287.jpg]

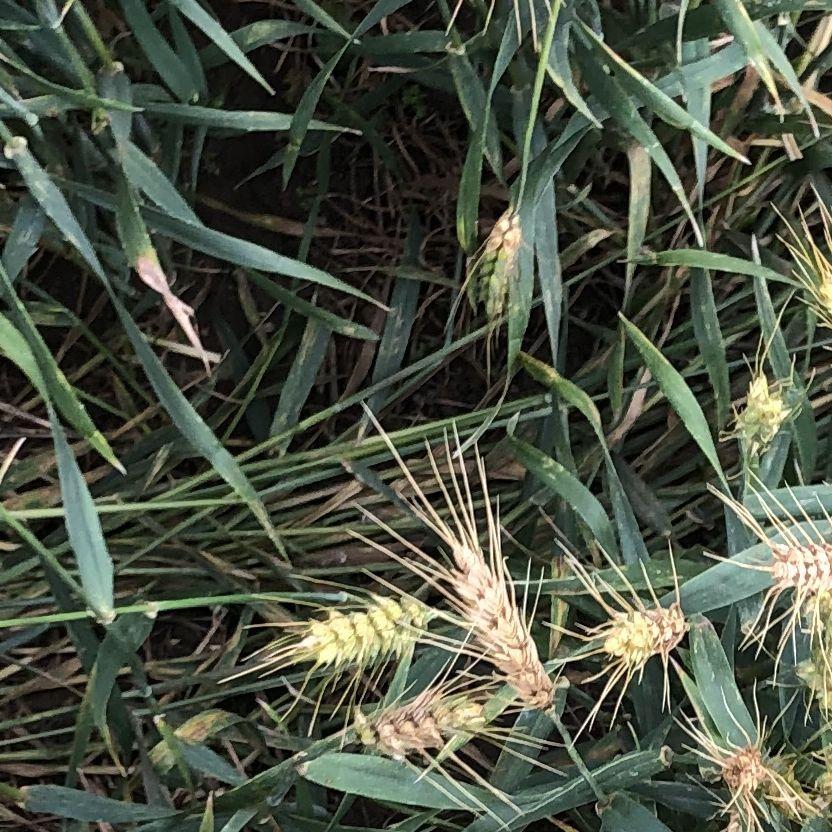

Supplement: Supplemental Information 3 [file peerj-cs-10-1948-s003.zip › data2/image0288.jpg]

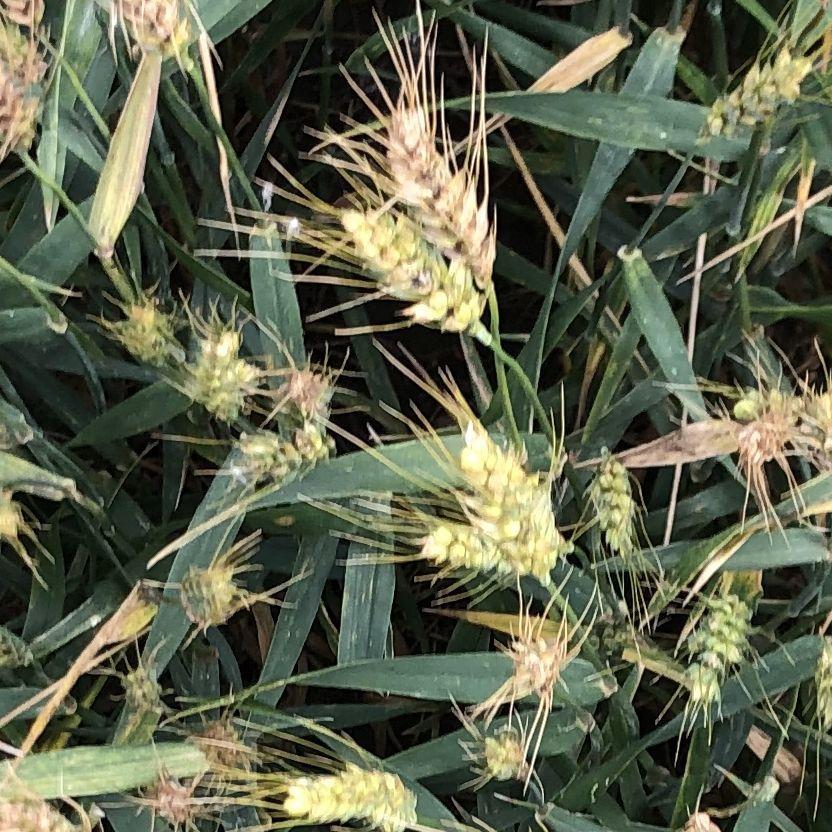

Supplement: Supplemental Information 3 [file peerj-cs-10-1948-s003.zip › data2/image0289.jpg]

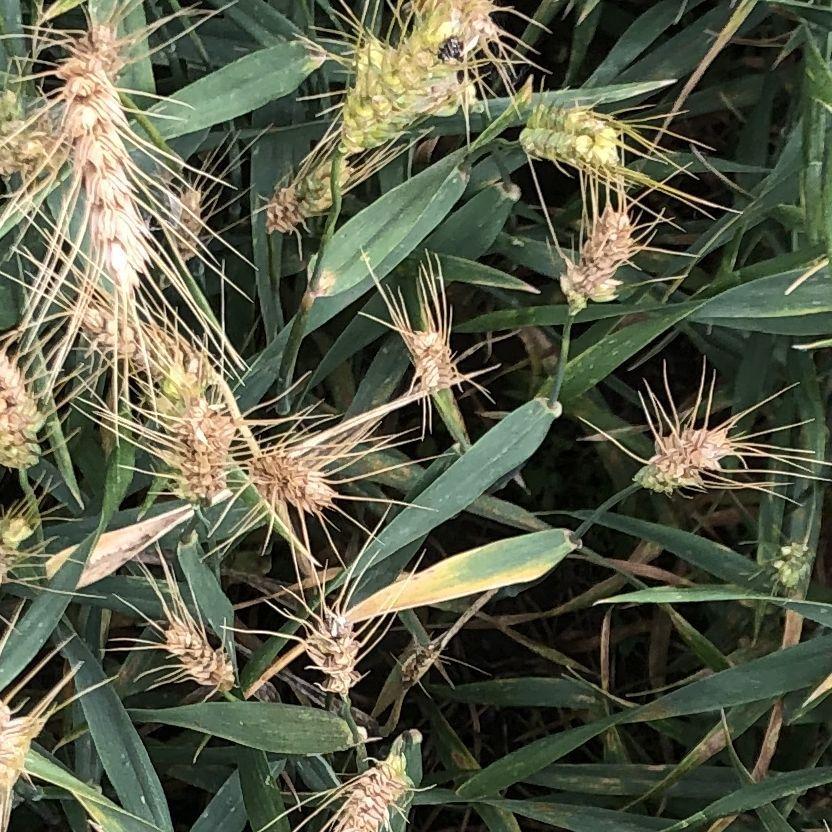

Supplement: Supplemental Information 3 [file peerj-cs-10-1948-s003.zip › data2/image0290.jpg]

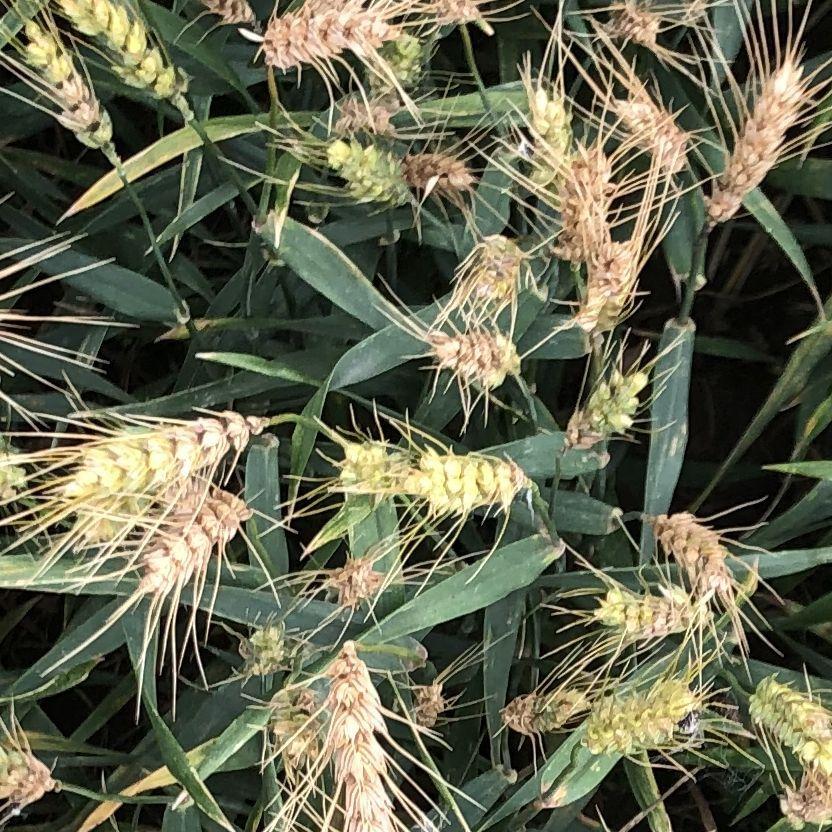

Supplement: Supplemental Information 3 [file peerj-cs-10-1948-s003.zip › data2/image0291.jpg]

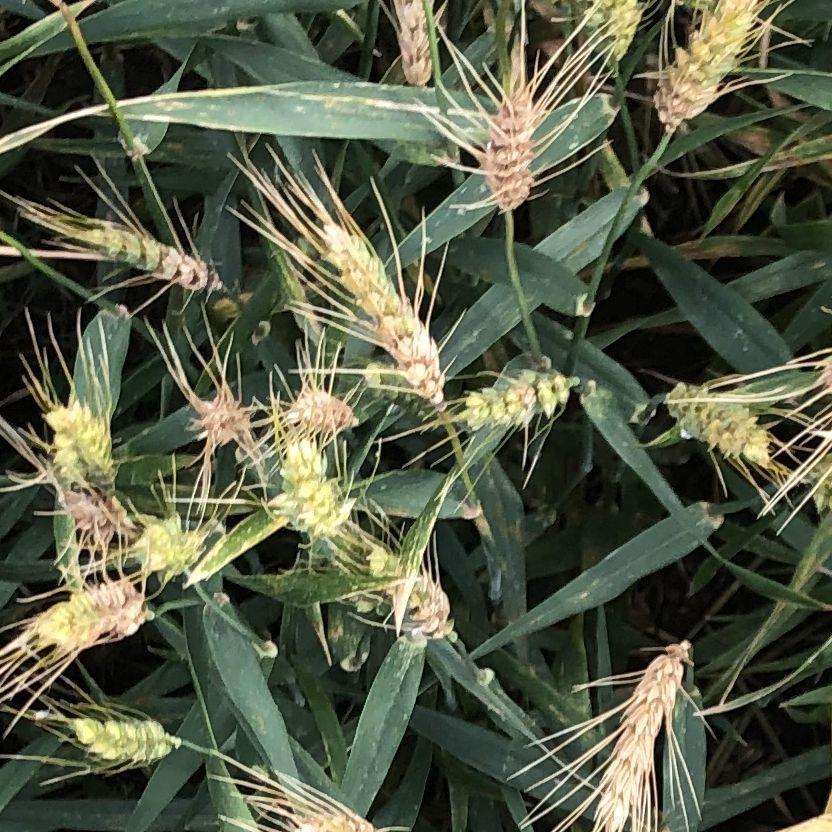

Supplement: Supplemental Information 3 [file peerj-cs-10-1948-s003.zip › data2/image0292.jpg]

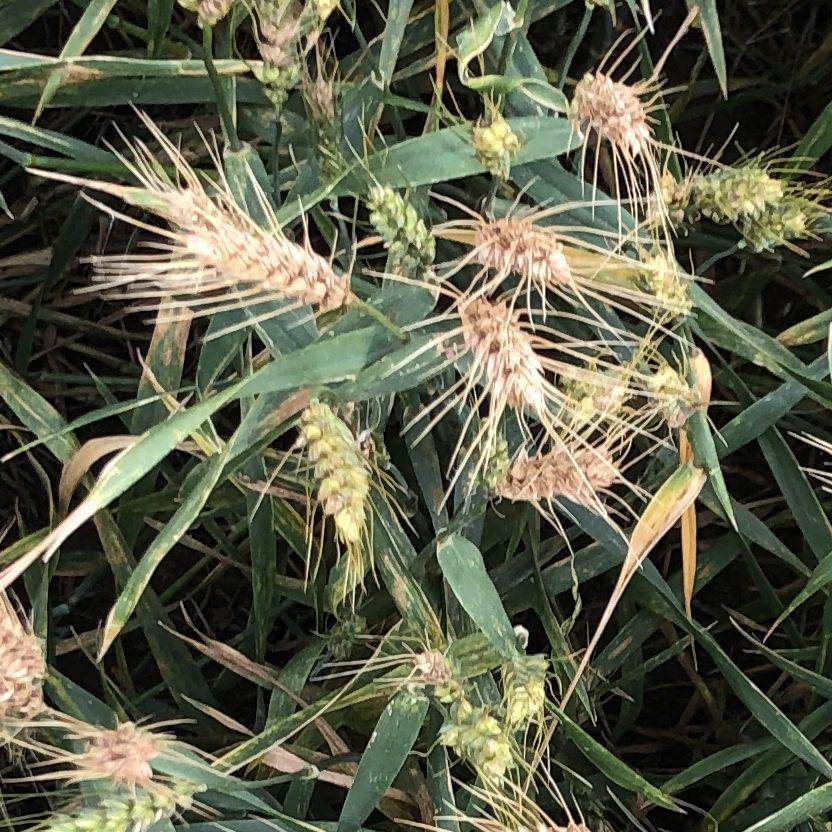

Supplement: Supplemental Information 3 [file peerj-cs-10-1948-s003.zip › data2/image0293.jpg]

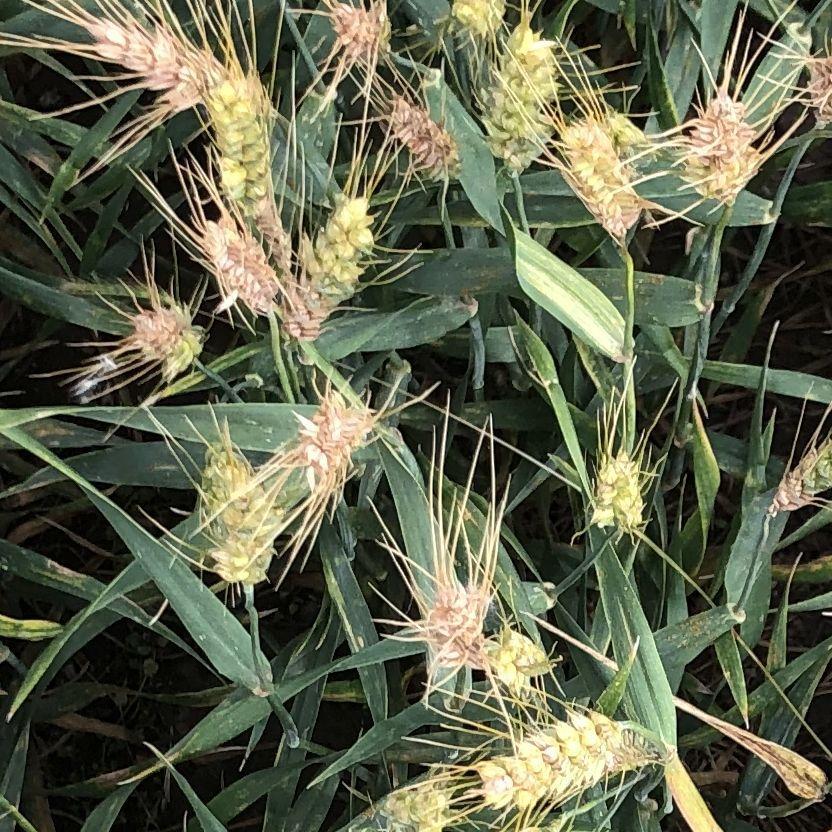

Supplement: Supplemental Information 3 [file peerj-cs-10-1948-s003.zip › data2/image0294.jpg]

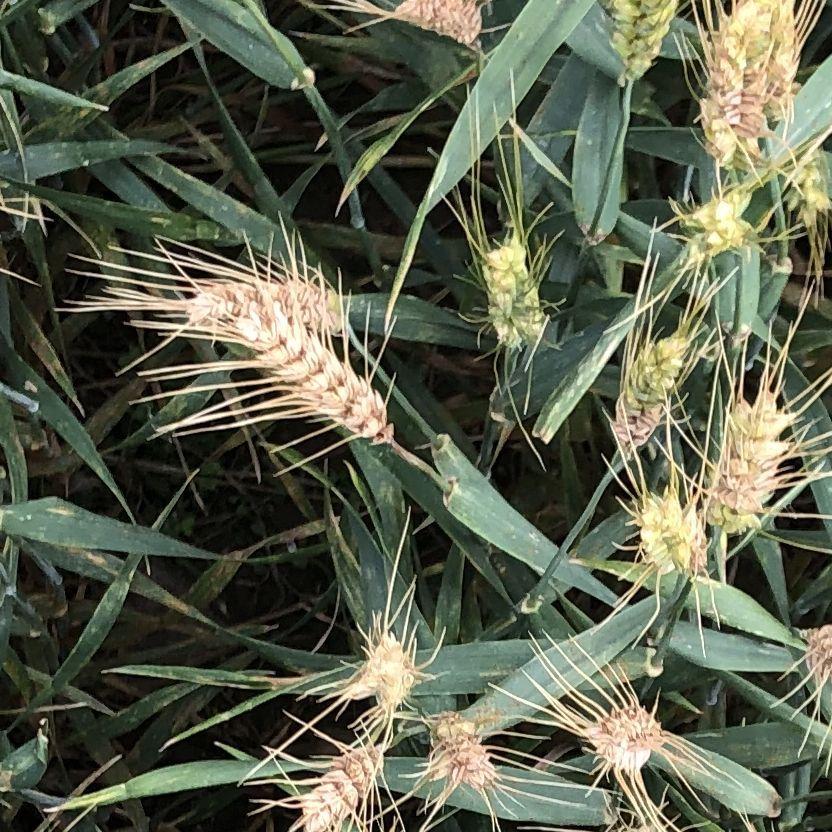

Supplement: Supplemental Information 3 [file peerj-cs-10-1948-s003.zip › data2/image0295.jpg]

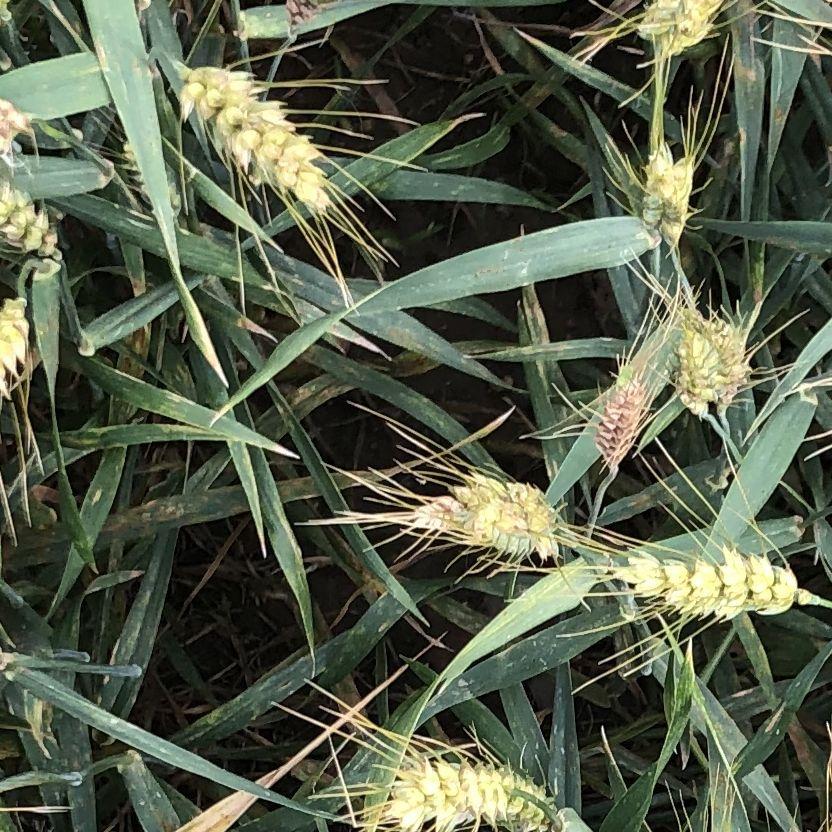

Supplement: Supplemental Information 3 [file peerj-cs-10-1948-s003.zip › data2/image0296.jpg]

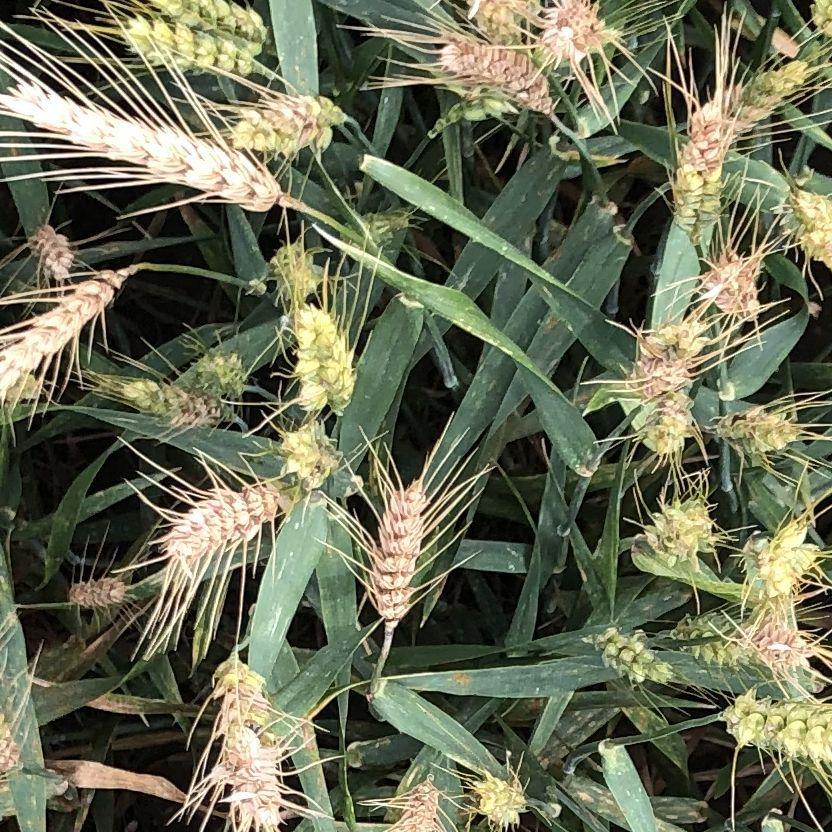

Supplement: Supplemental Information 3 [file peerj-cs-10-1948-s003.zip › data2/image0297.jpg]

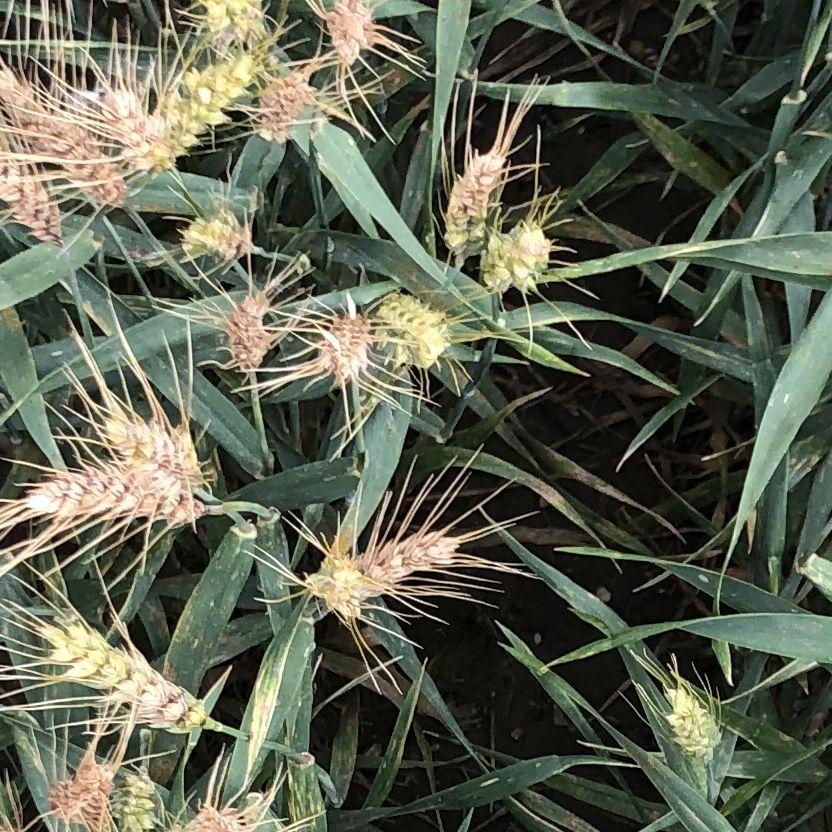

Supplement: Supplemental Information 3 [file peerj-cs-10-1948-s003.zip › data2/image0298.jpg]

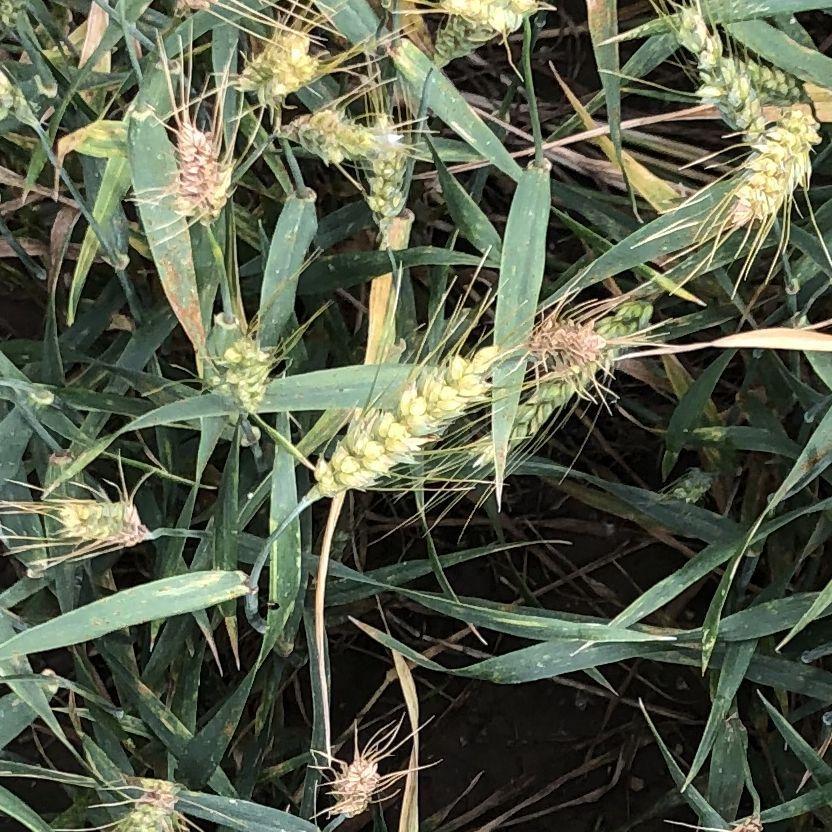

Supplement: Supplemental Information 3 [file peerj-cs-10-1948-s003.zip › data2/image0299.jpg]

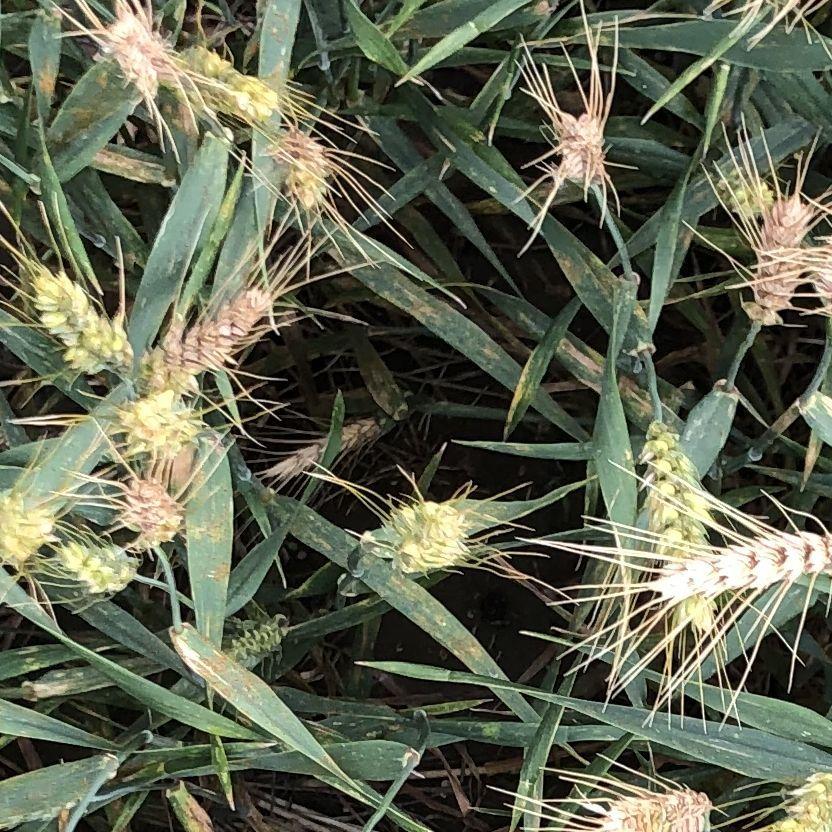

Supplement: Supplemental Information 3 [file peerj-cs-10-1948-s003.zip › data2/image0300.jpg]
